# Base-Mediated Nitrophenyl Reductive Cyclization for the Synthesis of Hexahydro-2,6-methano-1-benzazocines

**DOI:** 10.1021/acs.joc.2c02205

**Published:** 2022-11-04

**Authors:** Laura G. Rodríguez, Ana Delgado, Carlos J. Ciudad, Véronique Noé, Josep Bonjoch, Ben Bradshaw

**Affiliations:** † Laboratori de Química Orgànica, Facultat de Farmàcia, IBUB, Universitat de Barcelona, Avenida Joan XXIII 27-31, 08028 Barcelona, Spain; ‡ Department of Biochemistry and Physiology, Faculty of Pharmacy & IN2UB, University of Barcelona, Avenida Joan XXIII 27-31, 08028 Barcelona, Spain

## Abstract

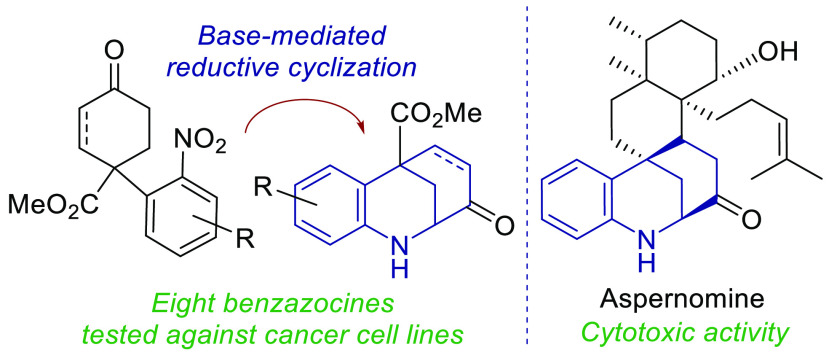

A Diels–Alder reaction leading to 4-nitrophenylcyclohexanones followed by a newly developed base-mediated reductive cyclization of the resulting ketone tethered to the nitrobenzene moiety gives access to the hexahydro-2,6-methano-1-benzazocine ring system present in several biologically interesting natural products such as aspernomine. The scope of the reaction was explored with eight substituted nitrobenzenes, obtaining yields of up to 87%. The highest cytotoxicity was observed in benzazocine **4h**, bearing an enone moiety, which was active against eight cancer cell lines.

A wide variety of fungi produce morphological structures known as sclerotia that are key for long-term species survival and propagation. A study of the sclerotia of *Aspergillus* by Gloer’s group led to the isolation of several biologically active secondary metabolites,^1^ including the complex indole diterpenoid anominine,^2^ most likely the parent structure from which the others are biogenetically derived.^3^ In a previous study on this natural product, our group achieved the first total synthesis of anominine and established its absolute configuration.^4^ Since then, a number of other products from this family have been synthesized.^5^

Among these fungal metabolites, some are reported to have antiinsectan properties, whereas the cytotoxic aspernomine,^6^ yet to be synthesized, has shown activity against the A549 lung carcinoma, MCF7 breast adenocarcinoma, and HT29 colon adenocarcinoma cell lines. Its anticancer properties could be attributable to the hexahydro-2,6-methano-1-benzazocine moiety, also present in the structurally related sespenine^3^ or strychnochromine^7^ (Figure 1).

**Figure 1:**
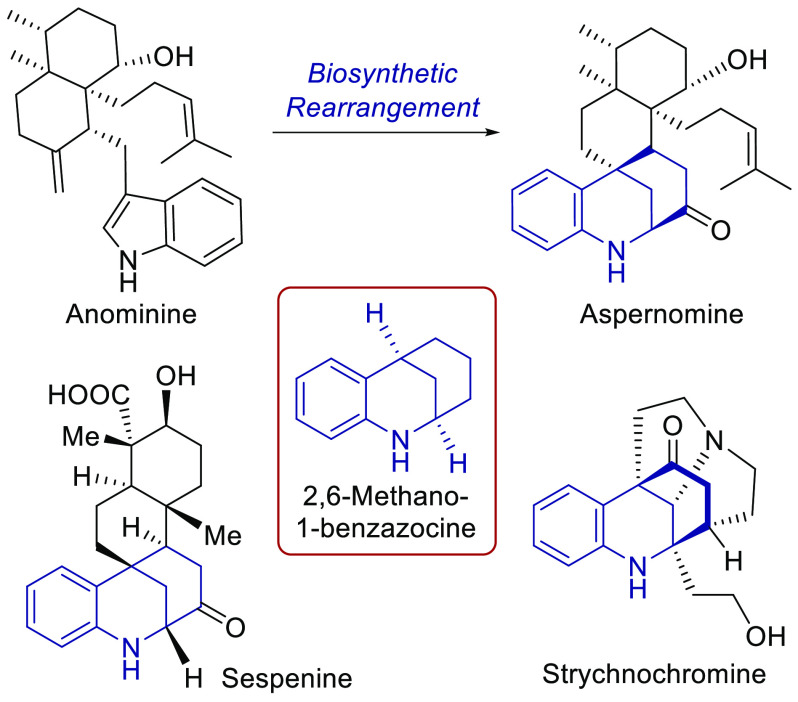
Structures of anominine and structurally related metabolites containing a 2,6-methano-1-benzazocine core.

Given the highly promising properties of these structures, the scarcity of precedents for their preparation indicates the synthetic challenge they pose.^8^ In the present study, we targeted the synthesis of the hexahydro-2,6-methano-1-benzazocine ring system through a base-mediated intramolecular nitrophenyl reductive cyclization based on precedents reported by our group (Scheme 1a). In studies of the total synthesis of *Strychnos* indole alkaloids, attempts at an α-formylation of a nitrophenylketone scaffold using tris(dimethylamino)methane resulted in the accidental discovery of a cyclized product bearing a pyrrolobenzazocine framework (Scheme 1b).^9^ In addition, in the context of the total synthesis of strychnine, endeavors to form a piperidine ring using propargylic and vinyl iodide precursors led to the unexpected formation of a bridged tetrahydroquinoline scaffold, albeit with low yields (Scheme 1c).^10^

**Scheme 1:**
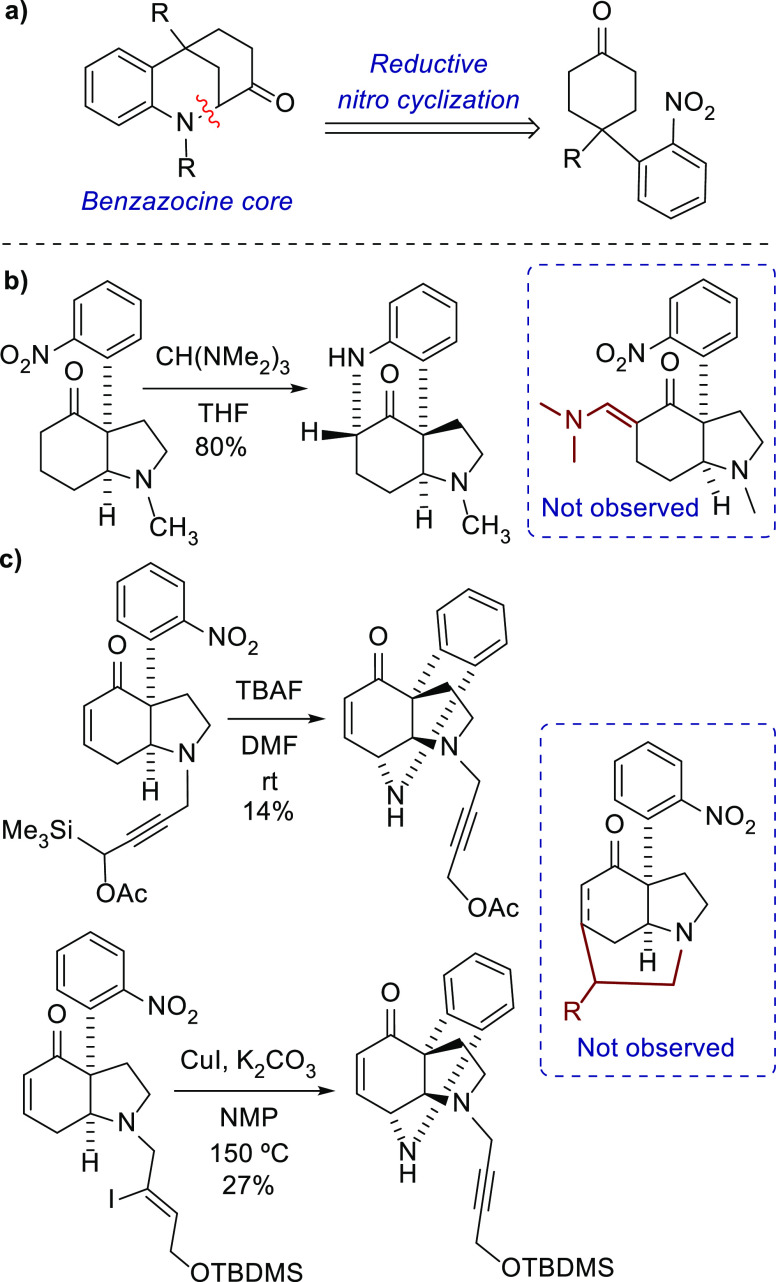
(a) Proposed Retrosynthesis of Aspernomine; (b, c) Synthetic Precedents for Bridged Benzazocines Reported by Our Group

With these precedents in mind, the cyclization of methyl-1-(2-nitrophenyl)-4-oxocyclohexane carboxylate (**3a**) was chosen as an initial model system for our study. The scope of the study was expanded to include other analogs bearing different substituents on the aromatic moiety (**3b**–**g**), and their reactivity and biological activity were compared with those of the model substrate and the natural product aspernomine.

To prepare the starting materials, a Diels–Alder coupling based on existing protocols in the literature was proposed.^11^ An effective two-step synthesis for the dienophile involving the aromatic nucleophilic substitution of dimethyl malonate on selected 2-fluoronitrobenzenes followed by treatment of nitromalonates **1a**–**g** with paraformaldehyde, Bu_4_NI, and K_2_CO_3_ afforded nitroaryl propenoates **2a**–**g** in high to very high yields.^12^ Subsequent coupling of the acrylates with the selected diene and hydrolysis of the silylated products furnished the precursors **3a**–**g**. Following an analogous protocol, acrylate **2a** was coupled with Danishefsky’s diene^13^ to provide the additional precursor **3h** in 76% yield with the alkene preinstalled, ready for further elaboration (Scheme 2).

**Scheme 2:**
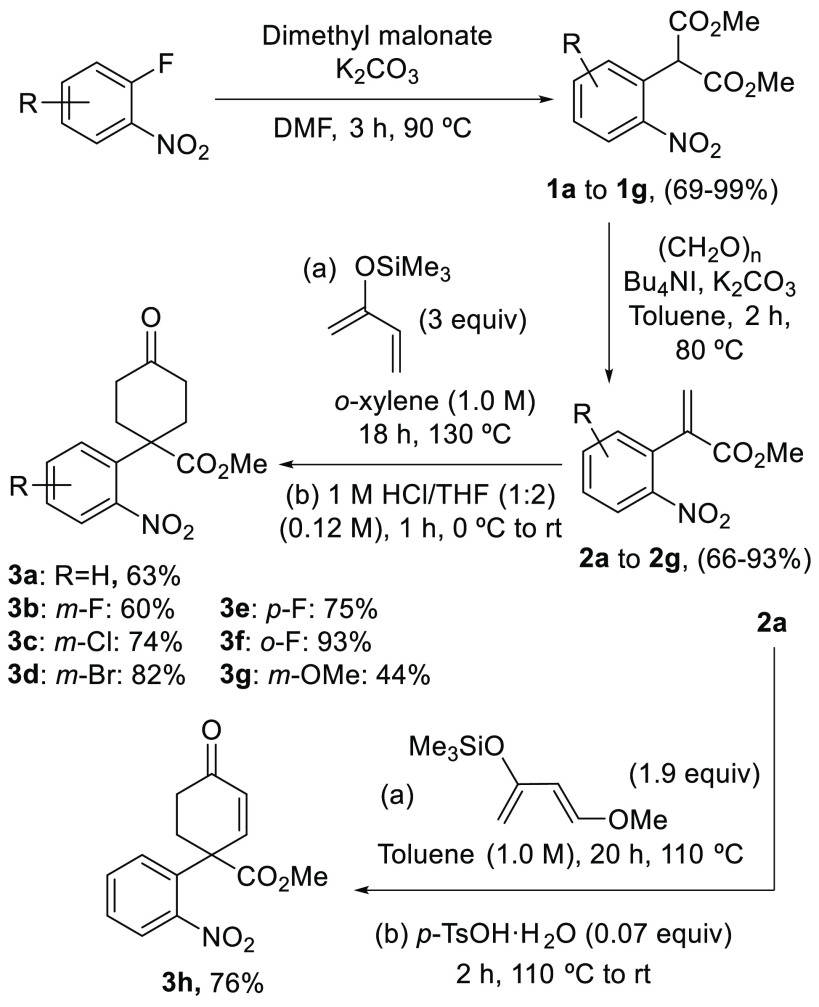
Synthesis of Substrates for the Base-Mediated Cyclization

With the precursors in hand, several studies using **3a** as the model substrate were carried out to achieve the cyclization product (Table 1). In early attempts employing Bredereck’s reagents, the unwanted formylation process^8b,9^ was clearly favored (Table 1, entries 1 and 2). When TBAF and HMPA in DMF were used instead (Table 1, entry 3), the reaction provided traces of the desired product and the starting material was largely unconsumed. However, the total isolated amount was poor and contained undetermined impurities. Promoting the cyclization with potassium carbonate and copper iodide proved to be a more efficient strategy, affording the product in 37% yield (Table 1, entry 4). However, despite the existing precedents,^10^ copper iodide was ineffective for the targeted intramolecular cyclization (Table 1, entry 5). At this point, the reaction time was significantly reduced by applying microwave irradiation, which also slightly improved the product yields (Table 1, entry 6);^14^ the yields were noticeably higher when the K_2_CO_3_ concentration was increased (Table 1, entry 7). The efficiency of the transformation was further improved by using NMP as the solvent (Table 1, entry 8). Experiments at 100 and 200 °C established 150 °C as the optimal temperature for the targeted cyclization (Table 1, entries 9 and 10). An additional increase in the amount of K_2_CO_3_ combined with the previously optimized conditions afforded product **4a** in 55% yield but without recovery of any starting material (Table 1, entry 11). At this point, we achieved a breakthrough by diluting the reaction mixture to a 0.01 M concentration, which enabled the product to be isolated in an excellent 82% yield (Table 1, entry 13). Finally, we scaled up the reaction to 1 mmol, which afforded the cyclized product in 87% yield (Table 1, entry 14).

**Table 1:**
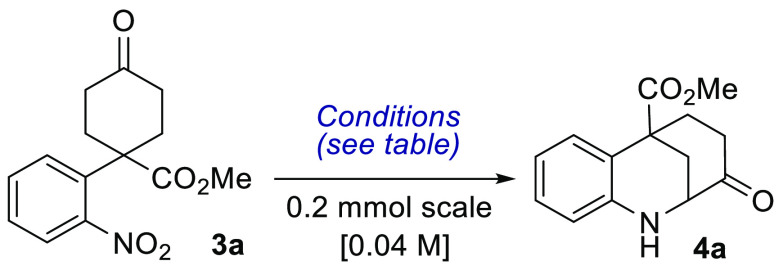
Optimization of the Reaction Conditions for the Reductive Cyclization

entry	solvent	*T* (°C)	time	base	yield (%)
1	THF	70	5 h	CH(NMe)_3_ (5 equiv)	0
2	THF	70	5 h	CH(NMe)_2_O*t*-Bu (3 equiv)	0
3	DMF	rt	18 h	TBAF (2 equiv) + HMPA (6 equiv)	13
4	DMF	150	4 h	K_2_CO_3_ (2 equiv) + CuI (1 equiv)	37
5	DMF	150	4 h	K_2_CO_3_ (2 equiv)	36
6	DMF	150^a^	15 min	K_2_CO_3_ (2 equiv)	40
7	DMF	150^a^	15 min	K_2_CO_3_ (4 equiv)	44
8	NMP	150^a^	15 min	K_2_CO_3_ (4 equiv)	50
9	NMP	100^a^	15 min	K_2_CO_3_ (4 equiv)	29
10	NMP	200^a^	15 min	K_2_CO_3_ (4 equiv)	34
11	NMP	150^a^	15 min	K_2_CO_3_ (10 equiv)	55
12	NMP	150	1 h	K_2_CO_3_ (10 equiv)	50
13	NMP^b^	150	1 h	K_2_CO_3_ (10 equiv)	82
14^c^	NMP^b^	150	1 h	K_2_CO_3_ (10 equiv)	87

a Use of microwaves.

b Concentration = 0.01 M.

c The reaction was carried out on a 1 mmol scale.

Once the reaction conditions for the cyclization of the model substrate **3a** had been optimized, we focused on the cyclization of the additional precursors prepared earlier (**3b**–**h**) (Figure 2). In general, modifications of the model substrate resulted in lower but still satisfactory yields. Notably, the presence of electron-withdrawing substituents at the meta position of the aromatic ring as well as the introduction of a double bond, which would be beneficial for further elaboration of the benzazocine framework, had slightly detrimental effects on the reaction. In contrast, the presence of an electron-donating group had a less significant impact on the isolated yields. Among all of the prepared compounds, product **4e** was the most crystalline and was submitted to X-ray diffraction to confirm its structure.

**Figure 2:**
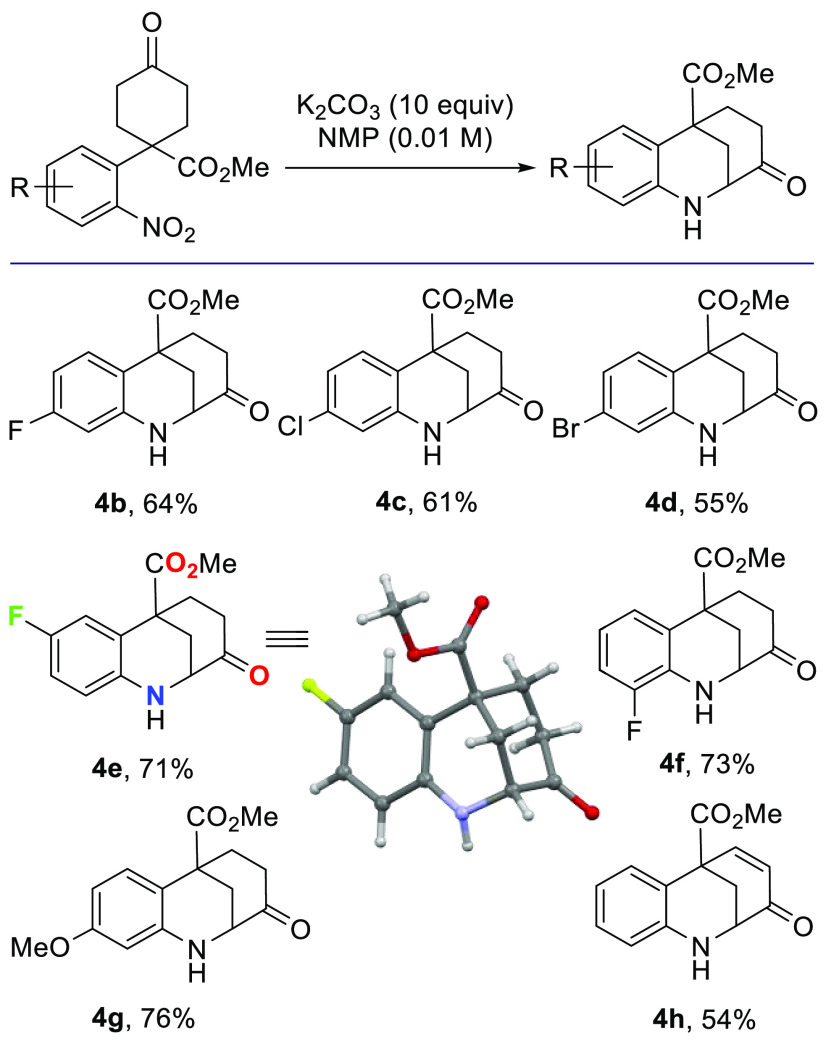
Exploring the scope of the base-mediated reductive cyclization, and X-ray structure of compound **4e**.

It was observed that the synthesized products were prone to partial degradation after prolonged storage. Therefore, with the aim of making these compounds easier to handle, we decided to protect the nitrogen atom (Scheme 3). However, using benzazocine **4a** as a test substrate, this task proved to be less straightforward than envisaged due to the poor nucleophilicity of the nitrogen atom caused by the neighboring electron-withdrawing groups. Attempted protection with a range of groups such as methyl chloroformate, *p*-TsCl, CbzCl, Ac_2_O, and Boc_2_O in the presence of various bases (TEA, DIPEA, K_2_CO_3_, NaOH, and NaH) did not result in any reaction. Interestingly, when the stronger base LiHMDS was used in combination with Boc anhydride,^15^ the protection of the nitrogen atom was accompanied by *O*-*tert*-butoxycarbonylation of the carbonyl group, providing benzazocine **5** along with recovered starting material. At this point, the formation of acetal **6** was performed to block the α carbon. Subsequent protection of the nitrogen atom under the conditions affording **5** gave compound **7** in excellent 91% yield.

**Scheme 3:**
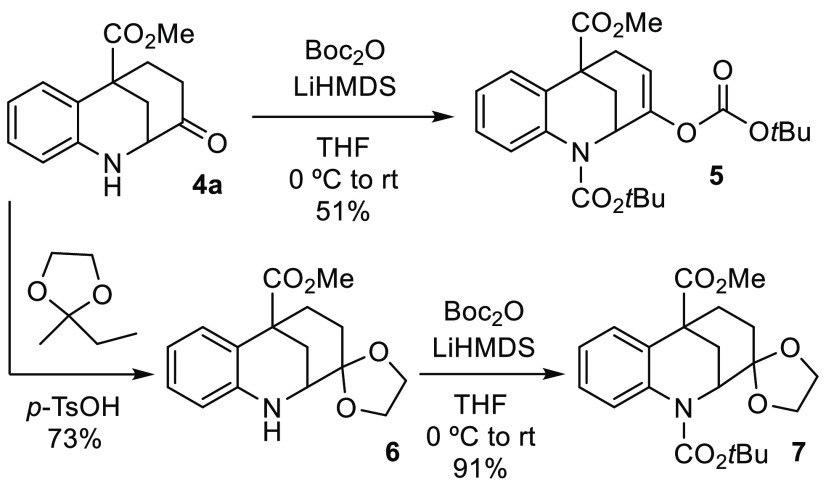
Protection of the Amino Group of the Benzazocine Framework

On the basis of the observed reactivity of the different products, a reaction mechanism involving a Grob fragmentation is proposed.^16^ After the initial enolate–nitrophenyl coupling, the overall process could imply a ring-opening nucleophilic attack on the carbonyl group of NMP, which would generate the five-atom scaffold required for the concerted fragmentation (Scheme 4). The cleavage of the indicated bonds would cause the reduction of the nitro to a deprotonated hydroxylamine **A**, which through an iterative sequence would enable a second reduction process, and the resulting amide **B** could render the targeted benzazocine product by protonation.^17^

**Scheme 4:**
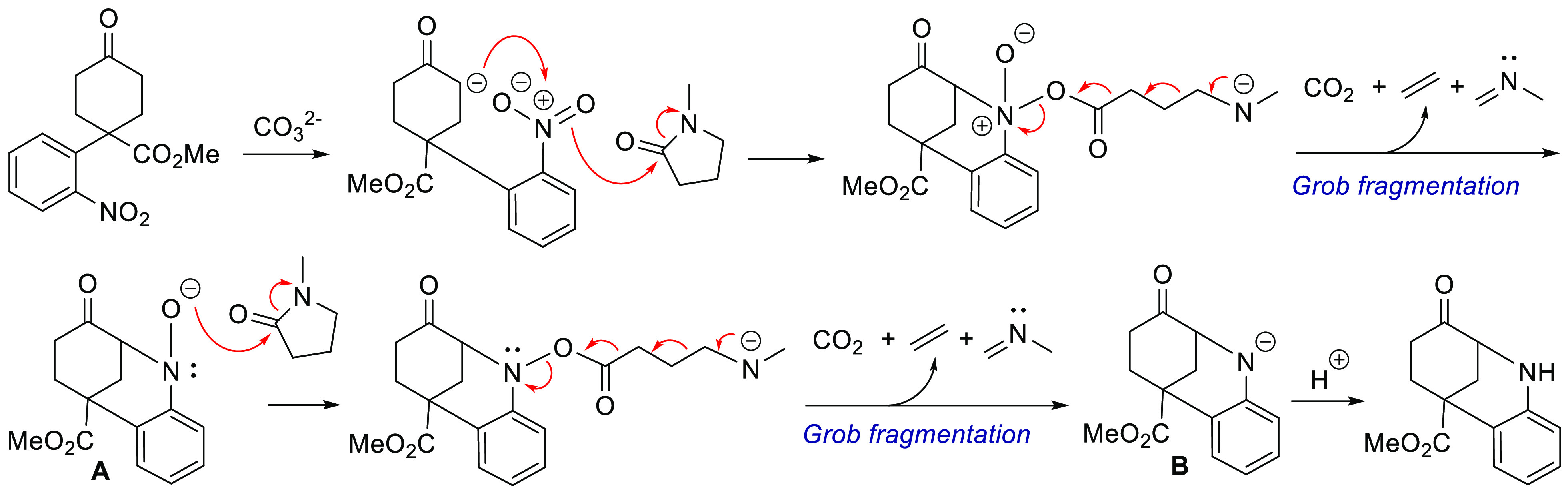
Proposed Mechanism for the Base-Mediated Reductive Cyclization involving a Grob Fragmentation

After optimizing the conditions for the synthesis of the hexahydro-2,6-methano-1-benzazocine scaffold, compounds **4a**–**h** were screened for their cytotoxic activity against the human breast cancer cell line MCF7. As shown in Table 2, the activity of the compounds was generally lower than that of the natural product aspernomine, which indicates that the cytotoxicity may not solely depend on the presence of the benzazocine moiety, and other structural factors in the natural product may also be important. Alternatively, the presence of an ester moiety or the racemic nature of our compounds may have contributed to the lower activities. However, the activity of substrate **4h**, which bears an enone fragment, was notably higher compared to the natural product and gave low IC_50_ values when tested against a variety of cancer cell lines. It is known that Michael acceptors can act as enzyme inhibitors by irreversibly alkylating cysteine residues via conjugate addition,^18^ and the toxicity of these compounds is likely attributable to nonspecific protein aggregation.^19^ However, it should also be pointed out that a number of Michael acceptor motif-containing drugs are cutting-edge treatments for several types of cancer,^20^ which endorses the biosynthetic interest of this structural framework and its further study.

**Table 2:**
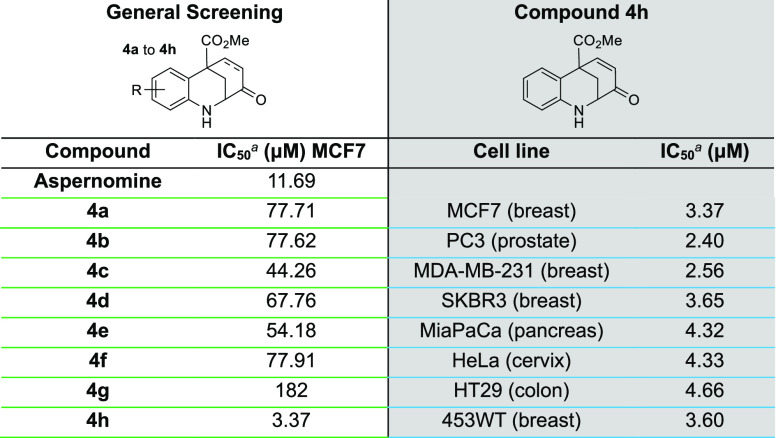
Activities of Compounds **4a–h** against the MCF7 Cell Line; Activities of Compound **4h** against Seven Additional Cell Lines

a IC_50_ values were determined after 5 days of incubation at increasing concentrations ranging from 300 nM to 300 μM.

In conclusion, we have developed efficient access to the important hexahydro-2,6-methano-1-benzazocine framework in only four steps using a Diels–Alder reaction and a novel base-mediated nitrophenyl reductive cyclization. The preparation of several analogs and their subsequent biological evaluation revealed that the heterocyclic core is less cytotoxic compared to aspernomine, whereas the introduction of an enone functionality notably increased the antiproliferative activity against human cancer cell lines. Future work will focus on the elaboration of the benzazocine framework toward the total synthesis of aspernomine.

## Experimental Section

### General Information

All reactions were carried out under an argon atmosphere with dry, freshly distilled solvents under anhydrous conditions. Analytical thin-layer chromatography was performed on SiO_2_ (Merck silica gel 60 F_254_), and the spots were located with 1% aqueous KMnO_4_ or 2% ethanolic anisaldehyde. Chromatography refers to flash chromatography and was carried out on SiO_2_ (SDS silica gel 60 ACC, 35–75 μm, 230–240 mesh ASTM). Drying of organic extracts during workup of reactions was performed over anhydrous Na_2_SO_4_. Evaporation of solvent was accomplished with a rotatory evaporator. NMR spectra were recorded in CDCl_3_ except where stated otherwise, and the chemical shifts of ^1^H and ^13^C NMR spectra are reported in ppm downfield (δ) from Me_4_Si or referenced at CDCl_3_. All NMR data assignments are supported by gCOSY and gHSQC experiments. Nitrobenzenes used for the reductive cyclization were purchased from Sigma-Aldrich (2,5-difluoronitrobenzene, 2,6-difluoronitrobenzene, and 4-fluoro-3-nitroanisole), Apollo Scientific (5-chloro-2-fluoronitrobenzene and 5-bromo-2-fluoronitrobenzene), Fluka (2-fluoronitrobenzene), and Alfa Aesar (2,4-difluoronitrobenzene). MCF7, SKBR3, MDA-MB-231, 453-WT, MiaPaCa, HeLa, HT29, and PC-3 cell lines were obtained from the cell bank resources from the University of Barcelona. Cells were grown in Ham’s F12 medium supplemented with 10% fetal bovine serum (GIBCO, Invitrogen, Barcelona, Spain) and incubated at 37 °C in a humidified 5% CO_2_ atmosphere. Subculture was performed using 0.05% Trypsin (Merck, Madrid, Spain). For IC_50_ determination, cells were plated at a density of 10 000 cells/35 mm diameter wells in 1 mL of medium and let to attach to the dish for 20 h before proceeding to cell incubation. The different compounds were first dissolved in 100% DMSO at 100 mM and then diluted appropriately so that the final concentration of DMSO in cell culture did not exceed 0.1%. Cells were incubated with the compounds at increasing concentrations ranging from 300 nM to 300 μM. After 5 days, viability was determined by the MTT assay. Briefly, culture medium was added with 100 μM sodium succinate plus 0.63 mM 3-(4,5-dimethylthyazol-2-yl)-2,5-diphenyltetrazolium bromide (both from Sigma-Aldrich, Madrid, Spain) and incubated for 3h at 37 °C. After incubation, culture medium was removed and lysis solution (0.57% of acetic acid and 10% of sodium dodecyl sulfate in dimethyl sulfoxide) (Sigma-Aldrich, Madrid, Spain) was added. Absorbance was measured at 570 nm in a Varioskan Lux from Thermoscientific using the SkanIt TM software v.6.0 to determine the percentage of cell survival relative to the untreated controls. IC_50_ was calculated using the specific application within GraphPath Prism v9.0.1 In addition, cell images for each condition were taken using a ZOE Fluorescent Cell Imager (Bio-Rad Laboratories, Inc., Spain) before the MTT assays.

### General Method for the Synthesis of Nitrophenyl Malonates

Dimethyl malonate (1 equiv), potassium carbonate (3 equiv), and the selected 2-fluoronitrobenzene (1 equiv) were charged in a round-bottom flask. Then, dimethylformamide (1.25 M) was added to the mixture. The resulting brown suspension was heated at 90 °C for 2 h. After cooling to room temperature, the mixture was diluted with ice–water (3 mL/mmol) and Et_2_O (3 mL/mmol). The aqueous layer was then extracted three times with Et_2_O. The combined organic layers were washed with brine, dried, and evaporated. The corresponding dimethyl malonate was purified by flash column chromatography on silica gel.

#### Dimethyl-(2-nitrophenyl)malonate (**1a**)^12a^

The title compound was prepared according to the general procedure using 2-fluoro-1-nitrobenzene (6.01 g, 4.50 mL, 42.6 mmol), dimethyl malonate (5.63 g, 4.90 mL, 42.6 mmol, 1 equiv), and potassium carbonate (17.69 g, 128.0 mmol, 3 equiv) in DMF (34 mL, 1.25 M). Purification by chromatography (CH_2_Cl_2_) gave **1a** (10.68 g, 99%) as a yellow oil. ^1^H NMR (500 MHz, CDCl_3_) δ 8.07 (dd, *J* = 8.2, 1.4 Hz, 1H), 7.65 (ddd, *J* = 7.6, 7.6, 1.4 Hz, 1H), 7.56–7.49 (m, 2H), 5.33 (s, 1H), 3.80 (s, 6H) ppm; ^13^C{^1^H} NMR (126 MHz, CDCl_3_) δ 167.8, 148.9, 133.7, 131.5, 129.5, 128.0, 125.4, 54.2, 53.3 ppm.

#### Dimethyl-2-(4-fluoro-2-nitrophenyl)malonate (**1b**)^21a^

The title compound was prepared according to the general procedure using 2,5-difluoronitrobenzene (1.59 g, 1.1 mL, 9.99 mmol), dimethyl malonate (1.32 g, 1.2 mL, 9.99 mmol, 1 equiv), and potassium carbonate (4.15 g, 30.0 mmol, 3 equiv) in DMF (8 mL, 1.25 M). Purification by chromatography (CH_2_Cl_2_) gave **1b** (2.50 g, 92%) as a pale-yellow solid; ^1^H NMR (400 MHz, CDCl_3_) δ 7.79 (dd, *J* = 8.2, 2.7 Hz, 1H), 7.55 (dd, *J* = 8.8, 5.3 Hz, 1H), 7.38 (ddd, *J* = 8.8, 7.2, 2.8 Hz, 1H), 5.31 (s, 1H), 3.80 (s, 6H) ppm; ^13^C{^1^H} NMR (101 MHz, CDCl_3_) δ 167.6, 163.2, 160.7, 149.5, 149.4, 133.4, 133.3, 124.1, 124.0, 121.1, 120.9, 113.2, 113.0, 53.5, 53.4 ppm; ^19^F NMR (376 MHz, CDCl_3_) δ −109.08 (td, *J* = 7.6, 5.1 Hz) ppm.

#### Dimethyl-2-(4-chloro-2-nitrophenyl)malonate (**1c**)^21a^

The title compound was prepared according to the general procedure using 4-chloro-1-fluoro-2-nitrobenzene (1.00 g, 0.67 mL, 5.70 mmol), dimethyl malonate (753 mg, 0.65 mL, 5.70 mmol, 1 equiv), and potassium carbonate (2.37 g, 17.2 mmol, 3 equiv) in DMF (4.6 mL, 1.25 M). Purification by chromatography (CH_2_Cl_2_) gave **1c** (1.47 g, 90%) as a yellow solid; ^1^H NMR (400 MHz, CDCl_3_) δ 8.05 (d, *J* = 2.2 Hz, 1H), 7.62 (dd, *J* = 8.4, 2.2 Hz, 1H), 7.49 (d, *J* = 8.4 Hz, 1H), 5.29 (s, 1H), 3.80 (s, 6H) ppm; ^13^C{^1^H} NMR (101 MHz, CDCl_3_) δ 167.4, 149.2, 135.5, 133.7, 132.8, 126.5, 125.5, 53.6, 53.5 ppm.

#### Dimethyl-2-(4-bromo-2-nitrophenyl)malonate (**1d**)^21b^

The title compound was prepared according to the general procedure using 5-bromo-2-fluoronitrobenzene (1.65 g, 0.92 mL, 7.50 mmol), dimethyl malonate (991 mg, 0.86 mL, 7.50 mmol, 1 equiv), and potassium carbonate (3.11 g, 22.5 mmol, 3 equiv) in DMF (6 mL, 1.25 M). Purification by chromatography (CH_2_Cl_2_) gave **1d** (2.36 g, 95%) as a pale-yellow solid; ^1^H NMR (400 MHz, CDCl_3_) δ 8.19 (d, *J* = 2.1 Hz, 1H), 7.77 (dd, *J* = 8.4, 2.1 Hz, 1H), 7.42 (d, *J* = 8.4 Hz, 1H), 5.27 (s, 1H), 3.79 (s, 6H) ppm; ^13^C{^1^H} NMR (101 MHz, CDCl_3_) δ 167.3, 149.2, 136.7, 133.0, 128.3, 126.9, 122.9, 53.7, 53.5 ppm.

#### Dimethyl 2-(5-fluoro-2-nitrophenyl)malonate (**1e**)^21c^

The title compound was prepared according to the general procedure using 2,4-difluoronitrobenzene (1.61 g, 1.1 mL, 10.1 mmol), dimethyl malonate (1.33 g, 1.2 mL, 10.1 mmol, 1 equiv), and potassium carbonate (4.19 g, 30.3 mmol, 3 equiv) in DMF (8 mL, 1.26 M). Purification by chromatography (hexane/EtOAc 97.5:2.5 → hexane/EtOAc 50:50) gave **1e** (2.05 g, 75%) as a white solid; ^1^H NMR (400 MHz, CDCl_3_) δ 8.15 (dd, *J* = 9.1, 5.1 Hz, 1H), 7.28–7.18 (m, 2H), 5.40 (s, 1H), 3.82 (s, 6H) ppm; ^13^C{^1^H} NMR (101 MHz, CDCl_3_) δ 167.3, 166.2, 163.6, 144.9, 131.4, 131.3, 128.3, 128.2, 118.9, 118.7, 116.6, 116.4, 54.1, 53.5 ppm; ^19^F NMR (376 MHz, CDCl_3_) δ −101.88 to −102.02 (m) ppm.

#### Dimethyl-2-(3-fluoro-2-nitrophenyl)malonate (**1f**)^21d^

The title compound was prepared according to the general procedure using 2,6-difluoronitrobenzene (1.00 g, 0.67 mL, 6.29 mmol), dimethyl malonate (832 mg, 0.72 mL, 6.30 mmol, 1 equiv), and potassium carbonate (2.62 g, 19.0 mmol, 3 equiv) in DMF (5 mL, 1.25 M). Purification by chromatography (CH_2_Cl_2_) gave **1f** (1.48 g, 87%) as a pale-yellow solid; ^1^H NMR (400 MHz, CDCl_3_) δ 7.56 (ddd, *J* = 8.2, 8.2, 5.3 Hz, 1H), 7.47–7.39 (m, 1H), 7.29 (ddd, *J* = 9.3, 8.2, 1.0 Hz, 1H), 4.86 (s, 1H), 3.80 (s, 6H) ppm; ^13^C{^1^H} NMR (101 MHz, CDCl_3_) δ 167.0, 155.6, 153.0, 139.8, 139.6, 132.7, 132.6, 128.0, 126.38, 126.35, 117.7, 117.5, 53.5, 52.6, 52.5 ppm; ^19^F NMR (376 MHz, CDCl_3_) δ −121.26 (dd, *J* = 9.5, 5.2 Hz) ppm.

#### Dimethyl-2-(4-methoxy-2-nitrophenyl)malonate (**1g**)^12a^

The title compound was prepared according to the general procedure using 4-fluoro-3-nitroanisole (993 mg, 5.80 mmol), dimethyl malonate (766 mg, 0.67 mL, 5.80 mmol, 1 equiv), and potassium carbonate (2.41 g, 17.4 mmol, 3 equiv) in DMF (4.6 mL, 1.25 M). Purification by chromatography (CH_2_Cl_2_) gave **1g** (1.13 g, 69%) as a white solid; ^1^H NMR (400 MHz, CDCl_3_) δ 7.56 (d, *J* = 2.7 Hz, 1H), 7.41 (d, *J* = 8.7 Hz, 1H), 7.17 (dd, *J* = 8.7, 2.8 Hz, 1H), 5.25 (s, 1H), 3.88 (s, 3H), 3.79 (s, 6H) ppm; ^13^C{^1^H} NMR (101 MHz, CDCl_3_) δ 168.1, 159.9, 149.5, 132.4, 120.0, 119.9, 110.3, 56.1, 53.5, 53.2 ppm.

### General Method for the Synthesis of Acrylates

To a solution of the selected diester (1 equiv) in toluene (0.4 M) at room temperature were added paraformaldehyde (3 equiv), tetrabutylammonium iodide (0.04 equiv), and potassium carbonate (3 equiv). The resulting mixture was stirred at 80 °C for 2 h. After cooling to room temperature, water (2.5 mL/mmol) was added and the aqueous phase was extracted three times with toluene. The combined organic layers were washed with brine, dried, and evaporated. The corresponding acrylate was purified by flash column chromatography on silica gel.

#### Methyl 2-(2-Nitrophenyl)acrylate (**2a**)^12b^

The title compound was prepared according to the general procedure using dimethyl (2-nitrophenyl)malonate (10.68 g, 42.2 mmol), paraformaldehyde (3.80 g, 126.5 mmol, 3 equiv), tetrabutylammonium iodide (624 mg, 1.69 mmol, 0.04 equiv), and potassium carbonate (17.5 g, 126.6 mmol, 3 equiv) in toluene (100 mL, 0.4 M). Purification by chromatography (CH_2_Cl_2_) gave **2a** (6.85 g, 78%) as a yellow oil. Spectral data were identical to those previously reported;^12b^^1^H NMR (400 MHz, CDCl_3_) δ 8.12 (ddd, *J* = 8.1, 1.3, 0.4 Hz, 1H), 7.65 (td, *J* = 7.5, 1.3 Hz, 1H), 7.54 (ddd, *J* = 8.2, 7.5, 1.5 Hz, 1H), 7.39 (ddd, *J* = 7.6, 1.5, 0.4 Hz, 1H), 6.55 (d, *J* = 0.9 Hz, 1H), 5.88 (d, *J* = 0.9 Hz, 1H), 3.73 (s, 3H) ppm; ^13^C{^1^H} NMR (101 MHz, CDCl_3_) δ 165.4, 148.0, 139.9, 133.8, 133.1, 132.3, 129.5, 127.6, 124.7, 52.4 ppm.

#### Methyl 2-(4-Fluoro-2-nitrophenyl)acrylate (**2b**)

The title compound was prepared according to the general procedure using dimethyl 2-(4-fluoro-2-nitrophenyl) malonate (2.39 g, 8.81 mmol), paraformaldehyde (793 mg, 26.4 mmol, 3 equiv), tetrabutylammonium iodide (129 mg, 0.35 mmol, 0.04 equiv), and potassium carbonate (3.65 g, 26.4 mmol, 3 equiv) in toluene (22 mL, 0.4 M). Purification by chromatography (CH_2_Cl_2_) gave **2b** (1.74 g, 88%) as a yellow oil; ^1^H NMR (400 MHz, CDCl_3_) δ 7.84 (ddd, *J* = 8.3, 2.2, 0.7 Hz, 1H), 7.42–7.33 (m, 2H), 6.54 (d, *J* = 0.7 Hz, 1H), 5.86 (d, *J* = 0.7 Hz, 1H), 3.72 (s, 3H) ppm; ^13^C{^1^H} NMR (101 MHz, CDCl_3_) δ 165.2, 163.4, 160.9, 148.5, 148.4, 139.0, 133.8, 133.7, 129.23, 129.20, 128.0, 121.0, 120.8, 112.7, 112.4, 52.5 ppm; ^19^F NMR (376 MHz, CDCl_3_) δ −109.31 to −109.43 (m) ppm. HRMS (ESI) *m*/*z* [M + H]^+^: calcd for C_10_H_9_FNO_4_^+^, 226.0510; found, 226.0519.

#### Methyl 2-(4-Chloro-2-nitrophenyl)acrylate (**2c**)^12b^

The title compound was prepared according to the general procedure using dimethyl 2-(4-chloro-2-nitrophenyl) malonate (1.41 g, 4.90 mmol), paraformaldehyde (441 mg, 14.7 mmol, 3 equiv), tetrabutylammonium iodide (74 mg, 0.20 mmol, 0.04 equiv), and potassium carbonate (2.03 g, 14.7 mmol, 3 equiv) in toluene (12 mL, 0.4 M). Purification by chromatography (CH_2_Cl_2_) gave **2c** (1.09 g, 92%) as a yellow oil. Spectral data were identical to those previously reported;^12b^^1^H NMR (400 MHz, CDCl_3_) δ 8.11 (d, *J* = 2.1 Hz, 1H), 7.62 (dd, *J* = 8.2, 2.1 Hz, 1H), 7.34 (d, *J* = 8.2 Hz, 1H), 6.56 (d, *J* = 0.8 Hz, 1H), 5.88 (d, *J* = 0.8 Hz, 1H), 3.72 (s, 3H) ppm; ^13^C{^1^H} NMR (101 MHz, CDCl_3_) δ 165.1, 148.3, 139.0, 135.3, 133.8, 133.3, 131.5, 128.2, 125.0, 52.5 ppm.

#### Methyl 2-(4-Bromo-2-nitrophenyl)acrylate (**2d**)^12b^

The title compound was prepared according to the general procedure using dimethyl 2-(4-bromo-2-nitrophenyl) malonate (2.19 g, 6.59 mmol), paraformaldehyde (595 mg, 19.8 mmol, 3 equiv), tetrabutylammonium iodide (103 mg, 0.28 mmol, 0.04 equiv), and potassium carbonate (2.74 g, 19.8 mmol, 3 equiv) in toluene (17 mL, 0.4 M). Purification by chromatography (CH_2_Cl_2_) gave **2d** (1.76 g, 93%) as a yellow solid. Spectral data were identical to those previously reported;^12b^^1^H NMR (400 MHz, CDCl_3_) δ 8.25 (d, *J* = 2.1 Hz, 1H), 7.78 (dd, *J* = 8.2, 2.0 Hz, 1H), 7.28 (d, *J* = 8.2 Hz, 1H), 6.56 (d, *J* = 0.7 Hz, 1H), 5.89 (d, *J* = 0.8 Hz, 1H), 3.73 (s, 3H) ppm; ^13^C{^1^H} NMR (101 MHz, CDCl_3_) δ 165.0, 148.3, 139.0, 136.7, 133.4, 131.9, 128.1, 127.8, 122.7, 52.5 ppm.

#### Methyl 2-(5-Fluoro-2-nitrophenyl)acrylate (**2e**)^12b^

The title compound was prepared according to the general procedure using dimethyl 2-(5-fluoro-2-nitrophenyl) malonate (1.95 g, 7.19 mmol), paraformaldehyde (649 mg, 21.6 mmol, 3 equiv), tetrabutylammonium iodide (107 mg, 0.29 mmol, 0.04 equiv), and potassium carbonate (2.99 g, 21.6 mmol, 3 equiv) in toluene (18 mL, 0.4 M). Purification by chromatography (CH_2_Cl_2_) gave **2e** (1.40 g, 87%) as a pale-yellow solid. Spectral data were identical to those previously reported;^12b^^1^H NMR (400 MHz, CDCl_3_) δ 8.18 (dd, *J* = 9.0, 5.0 Hz, 1H), 7.20 (ddd, *J* = 9.1, 7.2, 2.8 Hz, 1H), 7.08 (dd, *J* = 8.4, 2.8 Hz, 1H), 6.56 (d, *J* = 0.6 Hz, 1H), 5.88 (d, *J* = 0.6 Hz, 1H), 3.73 (s, 3H) ppm; ^13^C{^1^H} NMR (101 MHz, CDCl_3_) δ 166.3, 164.9, 163.7, 144.1, 139.3, 136.3, 136.2, 128.1, 127.7, 127.6, 119.4, 119.2, 116.4, 116.2, 52.6 ppm; ^19^F NMR (376 MHz, CDCl_3_) δ −103.04 to −103.14 (m) ppm.

#### Methyl 2-(3-Fluoro-2-nitrophenyl)acrylate (**2f**)^22^

The title compound was prepared according to the general procedure using dimethyl 2-(3-fluoro-2-nitrophenyl) malonate (1.46 g, 5.38 mmol), paraformaldehyde (486 mg, 16.2 mmol, 3 equiv), tetrabutylammonium iodide (81 mg, 0.22 mmol, 0.04 equiv), and potassium carbonate (2.24 g, 16.2 mmol, 3 equiv) in toluene (14 mL, 0.4 M). Purification by chromatography (CH_2_Cl_2_) gave **2f** (970 mg, 80%) as a yellow oil; ^1^H NMR (400 MHz, CDCl_3_) δ 7.53 (ddd, *J* = 8.5, 7.8, 5.1 Hz, 1H), 7.29 (ddd, *J* = 9.8, 8.5, 1.3 Hz, 1H), 7.20–7.15 (m, 1H), 6.61 (s, 1H), 5.96 (s, 1H), 3.76 (s, 3H) ppm; ^13^C{^1^H} NMR (101 MHz, CDCl_3_) δ 164.8, 155.8, 153.3, 137.2, 137.1, 133.6, 132.83, 132.75, 130.5, 126.8, 126.8, 117.8, 117.6, 52.6 ppm; ^19^F NMR (376 MHz, CDCl_3_) δ −121.03 (dd, *J* = 9.9, 4.9 Hz) ppm. HRMS (ESI) *m*/*z* [M + Na]^+^: calcd for C_10_H_8_FNNaO_4_^+^, 248.0335; found, 248.0328.

#### Methyl 2-(4-Methoxy-2-nitrophenyl)acrylate (**2g**)^12b^

The title compound was prepared according to the general procedure using dimethyl 2-(4-methoxy-2-nitrophenyl) malonate (0.99 g, 3.50 mmol), paraformaldehyde (315 mg, 10.5 mmol, 3 equiv), tetrabutylammonium iodide (52 mg, 0.14 mmol, 0.04 equiv), and potassium carbonate (1.45 g, 10.5 mmol, 3 equiv) in toluene (9 mL, 0.4 M). Purification by chromatography (hexane/EtOAc 95:5 → hexane/EtOAc 75:25) gave **2g** (554 mg, 66%) as a pale-yellow solid. Spectral data were identical to those previously reported;^12b^^1^H NMR (400 MHz, CDCl_3_) δ 7.63 (d, *J* = 2.6 Hz, 1H), 7.29 (d, *J* = 8.5 Hz, 1H), 7.16 (dd, *J* = 8.5, 2.6 Hz, 1H), 6.48 (d, *J* = 1.0 Hz, 1H), 5.82 (d, *J* = 1.0 Hz, 1H), 3.90 (s, 3H), 3.71 (s, 3H) ppm; ^13^C{^1^H} NMR (101 MHz, CDCl_3_) δ 165.8, 160.2, 148.6, 139.6, 133.1, 127.0, 125.3, 120.0, 109.6, 56.1, 52.4 ppm.

### General Method for the Synthesis of the Cyclization Precursors

To a solution of the selected acrylate (1 equiv) in *o*-xylene (1 M) was added 2-trimethylsiloxy-1,3-butadiene (3 equiv) under an argon atmosphere. The reaction tube was sealed, and the mixture was heated at 130 °C for 18 h. After cooling to room temperature, the solvent was evaporated and the crude product was dissolved in tetrahydrofuran (0.18 M). The mixture was cooled to 0 °C, and 1 M hydrochloric acid (2.8 mL/mmol) was added dropwise. After stirring the solution for 2 h, tetrahydrofuran was evaporated and the aqueous layer was diluted and extracted three times with ethyl acetate. The combined organic layers were washed with brine, dried, and concentrated. The corresponding product was purified by flash column chromatography on silica gel.

#### Methyl 1-(2-Nitrophenyl)-4-oxocyclohexanecarboxylate (**3a**)

The title compound was prepared according to the general procedure using methyl-2-(2-nitrophenyl)acrylate (1.49 g, 7.19 mmol) and 2-trimethylsiloxy-1,3-butadiene (3.07 g, 3.8 mL, 21.6 mmol, 3 equiv) in *o*-xylene (7.2 mL, 1 M). The residue was hydrolyzed in a 1:2 v/v 1 M hydrochloric acid/tetrahydrofuran mixture (0.12 M). Purification by chromatography (CH_2_Cl_2_ → CH_2_Cl_2_/MeOH 0.5%) gave **3a** (1.26 g, 63%) as a pale-brown solid; ^1^H NMR (400 MHz, CDCl_3_) δ 7.85–7.80 (m, 1H), 7.66–7.62 (m, 2H), 7.50–7.44 (m, 1H), 3.71 (s, 3H), 2.78–2.59 (m, 4H), 2.42–2.24 (m, 4H) ppm; ^13^C{^1^H} NMR (101 MHz, CDCl_3_) δ 209.5, 174.1, 149.6, 135.8, 133.1, 128.6, 128.5, 125.9, 52.6, 48.7, 37.7, 33.2 ppm. HRMS (ESI) *m*/*z* [M + H]^+^: calcd for C_14_H_16_NO_5_^+^, 278.1023; found, 278.1019.

#### Methyl 1-(4-Fluoro-2-nitrophenyl)-4-oxocyclohexane-1-carboxylate (**3b**)

The title compound was prepared according to the general procedure using methyl-2-(4-fluoro-2-nitrophenyl)acrylate (1.51 g, 6.71 mmol) and 2-trimethylsiloxy-1,3-butadiene (2.86 g, 3.5 mL, 20.1 mmol, 3 equiv) in *o*-xylene (6.7 mL, 1 M). The residue was hydrolyzed in a 1:2 v/v 1 M hydrochloric acid/tetrahydrofuran mixture (0.12 M). Purification by chromatography (CH_2_Cl_2_ → CH_2_Cl_2_/MeOH 0.3%) gave **3b** (1.18 g, 60%) as a pale-brown solid; ^1^H NMR (400 MHz, CDCl_3_) δ 7.64 (dd, *J* = 9.0, 5.3 Hz, 1H), 7.57 (dd, *J* = 7.9, 2.8 Hz, 1H), 7.36 (ddd, *J* = 8.9, 7.0, 2.9 Hz, 1H), 3.71 (s, 3H), 2.81–2.58 (m, 4H), 2.42–2.20 (m, 4H) ppm; ^13^C{^1^H} NMR (101 MHz, CDCl_3_) δ 209.2, 173.9, 162.4, 159.9, 150.1, 150.0, 132.1, 132.0, 130.2, 130.1, 120.2, 119.9, 113.8, 113.6, 52.7, 48.4, 37.6, 33.4 ppm; ^19^F NMR (471 MHz, CDCl_3_) δ −110.94 (td, *J* = 7.4, 5.2 Hz) ppm. HRMS (ESI) *m*/*z* [M + H]^+^: calcd for C_14_H_15_FNO_5_^+^, 296.0929; found, 296.0936.

#### Methyl 1-(4-Chloro-2-nitrophenyl)-4-oxocyclohexane-1-carboxylate (**3c**)

The title compound was prepared according to the general procedure using methyl-2-(4-chloro-2-nitrophenyl)acrylate (1.04 g, 4.30 mmol) and 2-trimethylsiloxy-1,3-butadiene (1.84 g, 2.2 mL, 12.9 mmol, 3 equiv) in *o*-xylene (4.3 mL, 1 M). The residue was hydrolyzed in a 1:2 v/v 1 M hydrochloric acid/tetrahydrofuran mixture (0.12 M). Purification by chromatography (CH_2_Cl_2_ → CH_2_Cl_2_/MeOH 1.0%) gave **3c** (990 mg, 74%) as a white solid; ^1^H NMR (400 MHz, CDCl_3_) δ 7.82 (dd, *J* = 2.1, 0.6 Hz, 1H), 7.63–7.56 (m, 2H), 3.71 (s, 3H), 2.80–2.58 (m, 4H), 2.41–2.20 (m, 4H) ppm; ^13^C{^1^H} NMR (101 MHz, CDCl_3_) δ 209.1, 173.7, 149.9, 134.48, 134.47, 133.0, 129.7, 126.0, 52.7, 48.5, 37.6, 33.2 ppm. HRMS (ESI) *m*/*z* [M + H]^+^: calcd for C_14_H_15_ClNO_5_^+^, 312.0633; found, 312.0640.

#### Methyl 1-(4-Bromo-2-nitrophenyl)-4-oxocyclohexane-1-carboxylate (**3d**)^11a^

The title compound was prepared according to the general procedure using methyl-2-(4-bromo-2-nitrophenyl)acrylate (1.66 g, 5.80 mmol) and 2-trimethylsiloxy-1,3-butadiene (2.48 g, 3.1 mL, 17.4 mmol, 3 equiv) in *o*-xylene (5.8 mL, 1 M). The residue was hydrolyzed in a 1:2 v/v 1 M hydrochloric acid/tetrahydrofuran mixture (0.12 M). Purification by chromatography (CH_2_Cl_2_ → CH_2_Cl_2_/MeOH 0.3%) gave **3d** (1.68 g, 82%) as a pale-yellow solid. Spectral data were identical to those previously reported;^11a^^1^H NMR (400 MHz, CDCl_3_) δ 7.96 (d, *J* = 2.2 Hz, 1H), 7.76 (dd, *J* = 8.6, 2.2 Hz, 1H), 7.51 (d, *J* = 8.6 Hz, 1H), 3.71 (s, 3H), 2.80–2.68 (m, 2H), 2.68–2.58 (m, 2H), 2.41–2.30 (m, 2H), 2.30–2.20 (m, 2H); ^13^C{^1^H} NMR (101 MHz, CDCl_3_) δ 209.1, 173.6, 150.0, 136.0, 135.0, 129.9, 128.8, 121.9, 52.7, 48.6, 37.6, 33.2 ppm.

#### Methyl 1-(5-Fluoro-2-nitrophenyl)-4-oxocyclohexane-1-carboxylate (**3e**)

The title compound was prepared according to the general procedure using methyl-2-(5-fluoro-2-nitrophenyl)acrylate (1.37 g, 6.08 mmol) and 2-trimethylsiloxy-1,3-butadiene (2.60 g, 3.2 mL, 18.3 mmol, 3 equiv) in *o*-xylene (6.1 mL, 1 M). The residue was hydrolyzed in a 1:2 v/v 1 M hydrochloric acid/tetrahydrofuran mixture (0.12 M). Purification by chromatography (CH_2_Cl_2_ → CH_2_Cl_2_/MeOH 0.3%) gave **3e** (1.35 g, 75%) as a white solid; ^1^H NMR (400 MHz, CDCl_3_) δ 7.95 (dd, *J* = 9.0, 5.4 Hz, 1H), 7.33 (dd, *J* = 10.4, 2.7 Hz, 1H), 7.16 (ddd, *J* = 8.9, 6.6, 2.7 Hz, 1H), 3.71 (s, 3H), 2.84–2.71 (m, 2H), 2.71–2.60 (m, 2H), 2.43–2.32 (m, 2H), 2.30–2.18 (m, 2H); ^13^C{^1^H} NMR (101 MHz, CDCl_3_) δ 209.1, 173.5, 166.0, 163.4, 145.5, 140.2, 140.1, 128.84, 128.75, 116.2, 116.0, 115.5, 115.2, 52.7, 48.7, 37.5, 33.1 ppm; ^19^F NMR (376 MHz, CDCl_3_) δ −102.61 (dt, *J* = 10.6, 6.1 Hz) ppm. HRMS (ESI) *m*/*z* [M + H]^+^: calcd for C_14_H_15_FNO_5_^+^, 296.0929; found, 296.0932.

#### Methyl 1-(3-Fluoro-2-nitrophenyl)-4-oxocyclohexane-1-carboxylate (**3f**)

The title compound was prepared according to the general procedure using methyl-2-(3-fluoro-2-nitrophenyl)acrylate (1.40 g, 6.22 mmol) and 2-trimethylsiloxy-1,3-butadiene (2.65 g, 3.2 mL, 18.6 mmol, 3 equiv) in *o*-xylene (6.2 mL, 1 M). The residue was hydrolyzed in a 1:2 v/v 1 M hydrochloric acid/tetrahydrofuran mixture (0.12 M). Purification by chromatography (CH_2_Cl_2_ → CH_2_Cl_2_/MeOH 1.5%) gave **3f** (1.70 g, 93%) as a white solid; ^1^H NMR (400 MHz, CDCl_3_) δ 7.54 (td, *J* = 8.3, 5.7 Hz, 1H), 7.38 (dt, *J* = 8.3, 1.2 Hz, 1H), 7.27 (td, *J* = 8.6, 1.1 Hz, 1H), 3.76 (s, 3H), 2.72–2.59 (m, 4H), 2.46–2.34 (m, 2H), 2.34–2.19 (m, 2H) ppm; ^13^C{^1^H} NMR (126 MHz, CDCl_3_) δ 208.7, 173.2, 155.7, 153.7, 139.7, 139.6, 135.2, 131.94, 131.88, 123.29, 123.27, 116.8, 116.6, 53.1, 49.29, 49.28, 37.8, 33.2 ppm; ^19^F NMR (471 MHz, CDCl_3_) δ −122.49 (ddd, *J* = 8.9, 5.7, 1.3 Hz) ppm. HRMS (ESI) *m*/*z* [M + H]^+^: calcd for C_14_H_15_FNO_5_^+^, 296.0929; found, 296.0933.

#### Methyl 1-(4-Methoxy-2-nitrophenyl)-4-oxocyclohexane-1-carboxylate (**3g**)

The title compound was prepared according to a modification of the general procedure using methyl-2-(4-methoxy-2-nitrophenyl)acrylate (1.07 g, 4.51 mmol) and 2-trimethylsiloxy-1,3-butadiene (2.56 g, 3.2 mL, 18.0 mmol, 4 equiv) in *o*-xylene (4.5 mL, 1 M). The residue was hydrolyzed in a 1:2 v/v 1 M hydrochloric acid/tetrahydrofuran mixture (0.12 M). Purification by chromatography (CH_2_Cl_2_ → CH_2_Cl_2_/MeOH 1.0%) gave **3g** (616 mg, 44%) as a green solid; ^1^H NMR (400 MHz, CDCl_3_) δ 7.52 (d, *J* = 8.9 Hz, 1H), 7.33 (d, *J* = 2.8 Hz, 1H), 7.14 (dd, *J* = 8.9, 2.9 Hz, 1H), 3.87 (s, 3H), 3.70 (s, 3H), 2.77–2.55 (m, 4H), 2.40–2.21 (m, 4H) ppm; ^13^C{^1^H} NMR (101 MHz, CDCl_3_) δ 209.7, 174.4, 159.1, 150.1, 129.5, 127.4, 118.7, 111.2, 56.0, 52.6, 48.2, 37.8, 33.4 ppm. HRMS (ESI) *m*/*z* [M + H]^+^: calcd for C_15_H_18_NO_6_^+^, 308.1129; found, 308.1128.

### Synthesis of Methyl 1-(2-Nitrophenyl)-4-oxo-2-cyclohexene-1-carboxylate (**3h**)^11b^

A solution of methyl 2-(2-nitrophenyl)acrylate (1.26 g, 6.08 mmol) and 1-methoxy-3-trimethylsilyloxy-1,3-butadiene (2.00 g, 11.6 mmol, 1.9 equiv) in toluene (12 mL, 0.5 M) was heated at reflux in a sealed tube for 20 h. Solid *p*-toluenesulfonic acid monohydrate (228 mg, 1.20 mmol, 0.2 equiv) was added to the mixture, and heating was discontinued. The resulting solution was stirred for 1 h, diluted with ethyl acetate (60 mL), washed with water (25 mL) and brine (25 mL), dried with sodium sulfate, and concentrated. Purification by chromatography (hexane/EtOAc 97.5:2.5 → hexane/EtOAc 50:50) gave **3h** (1.28 g, 76%) as a yellow oil. Spectral data were identical to those previously reported;^11b^^1^H NMR (400 MHz, CDCl_3_) δ 8.00 (ddd, *J* = 8.1, 1.5, 0.4 Hz, 1H), 7.64 (ddd, *J* = 7.9, 7.4, 1.5 Hz, 1H), 7.51 (ddd, *J* = 8.1, 7.5, 1.4 Hz, 1H), 7.46 (ddd, *J* = 7.8, 1.4, 0.4 Hz, 1H), 6.79 (d, *J* = 10.2 Hz, 1H), 6.32 (d, *J* = 10.2 Hz, 1H), 3.69 (s, 3H), 3.30–3.18 (m, 1H), 3.00–2.89 (m, 1H), 2.46–2.30 (m, 2H) ppm; ^13^C{^1^H} NMR (101 MHz, CDCl_3_) δ 198.0, 171.0, 148.7, 147.5, 135.8, 133.5, 132.3, 130.4, 129.0, 126.2, 52.9, 34.4, 32.3 ppm.

### General Method for the Synthesis of the Cyclized Products

A solution of potassium carbonate (10 equiv) in *N*-methylpyrrolidinone (90 mL/mmol) under an argon atmosphere was heated to 150 °C in a preheated oil bath. Then, a solution of the selected Diels–Alder product (1 equiv) in NMP (10 mL/mmol) was added dropwise to the reaction mixture over 15 min. After this time, the solution was vigorously stirred for 1 h. The resulting dark brown mixture was filtered through Celite to retain the salts and subsequently evaporated to dryness. The corresponding product was purified by flash column chromatography on silica gel.

#### Methyl 3-Oxo-2,3,4,5-tetrahydro-2,6-methanobenzo[*b*]azocine-6(1*H*)-carboxylate (**4a**)

The title compound was prepared according to the general procedure using methyl 1-(2-nitrophenyl)-4-oxocyclohexane-1-carboxylate (277 mg, 1.00 mmol) and potassium carbonate (1.38 g, 9.98 mmol, 10 equiv) in *N*-methylpyrrolidinone (100 mL, 0.01 M). Purification by chromatography (CH_2_Cl_2_) gave **4a** (213 mg, 87%) as a yellow oil; ^1^H NMR (400 MHz, CDCl_3_) δ 7.12 (ddd, *J* = 8.2, 7.3, 1.5 Hz, 1H), 6.97 (dd, *J* = 7.7, 1.5 Hz, 1H), 6.72 (ddd, *J* = 7.8, 7.3, 1.2 Hz, 1H), 6.59 (dd, *J* = 8.0, 1.2 Hz, 1H), 4.49 (s, 1H), 3.86–3.82 (m, 1H), 3.76 (s, 3H), 2.63 (dt, *J* = 13.2, 3.7 Hz, 1H), 2.47–2.41 (m, 1H), 2.35–2.23 (m, 3H), 2.17 (ddd, *J* = 13.2, 2.9, 1.5 Hz, 1H) ppm; ^13^C{^1^H} NMR (101 MHz, CDCl_3_) δ 208.3, 175.3, 141.7, 128.9, 126.9, 121.1, 118.3, 114.4, 57.3, 52.6, 44.7, 38.8, 34.7, 32.8 ppm. HRMS (ESI) *m*/*z* [M + H]^+^: calcd for C_14_H_16_NO_3_^+^, 246.1125; found, 246.1125.

#### Methyl 9-Fluoro-3-oxo-2,3,4,5-tetrahydro-2,6-methanobenzo[*b*]azocine-6(1*H*)-carboxylate (**4b**)

The title compound was prepared according to the general procedure using methyl 1-(4-fluoro-2-nitrophenyl)-4-oxocyclohexane-1-carboxylate (89 mg, 0.30 mmol) and potassium carbonate (415 mg, 3.00 mmol, 10 equiv) in *N*-methylpyrrolidinone (30 mL, 0.01 M). Purification by chromatography (CH_2_Cl_2_) gave **4b** (51 mg, 64%) as a yellow oil; ^1^H NMR (400 MHz, CDCl_3_) δ 6.93 (dd, *J* = 8.6, 6.1 Hz, 1H), 6.42 (td, *J* = 8.5, 2.5 Hz, 1H), 6.30 (dd, *J* = 10.3, 2.6 Hz, 1H), 4.61 (s, 1H), 3.85–3.81 (m, 1H), 3.76 (s, 3H), 2.59 (dt, *J* = 13.3, 3.7 Hz, 1H), 2.45–2.37 (m, 1H), 2.35–2.22 (m, 3H), 2.17 (dd, *J* = 13.3, 2.9 Hz, 1H) ppm; ^13^C{^1^H} NMR (101 MHz, CDCl_3_) δ 207.9, 175.0, 164.4, 162.0, 143.3, 143.2, 128.5, 128.4, 116.91, 116.88, 105.5, 105.2, 101.0, 100.7, 57.1, 52.7, 44.3, 38.8, 34.5, 32.9 ppm; ^19^F NMR (471 MHz, CDCl_3_) δ −113.62 (ddd, *J* = 10.3, 8.3, 6.1 Hz) ppm. HRMS (ESI) *m*/*z* [M + H]^+^: calcd for C_14_H_15_FNO_3_^+^, 264.1036; found, 264.1032.

#### Methyl 9-Chloro-3-oxo-2,3,4,5-tetrahydro-2,6-methanobenzo[*b*]azocine-6(1*H*)-carboxylate (**4c**)

The title compound was prepared according to the general procedure using methyl 1-(4-chloro-2-nitrophenyl)-4-oxocyclohexane-1-carboxylate (56 mg, 0.18 mmol) and potassium carbonate (249 mg, 1.80 mmol, 10 equiv) in *N*-methylpyrrolidinone (18 mL, 0.01 M). Purification by chromatography (CH_2_Cl_2_) gave **4c** (31 mg, 61%) as a yellow solid; ^1^H NMR (400 MHz, CDCl_3_) δ 6.91 (d, J = 8.3 Hz, 1H), 6.69 (dd, J = 8.3, 2.1 Hz, 1H), 6.60 (d, J = 2.1 Hz, 1H), 4.56 (s, 1H), 3.86–3.81 (m, 1H), 3.76 (s, 3H), 2.58 (dt, J = 13.3, 3.7 Hz, 1H), 2.45–2.37 (m, 1H), 2.37–2.23 (m, 3H), 2.18 (dd, J = 13.3, 2.9 Hz, 1H) ppm; ^13^C{^1^H} NMR (101 MHz, CDCl_3_) δ 207.7, 174.8, 142.8, 134.4, 128.2, 119.6, 118.4, 114.0, 57.1, 52.7, 44.4, 38.6, 34.5, 32.7 ppm. HRMS (ESI) *m*/*z* [M + H]^+^: calcd for C_14_H_15_ClNO_3_^+^, 280.0740; found, 280.0739.

#### Methyl 9-Bromo-3-oxo-2,3,4,5-tetrahydro-2,6-methanobenzo[*b*]azocine-6(1*H*)-carboxylate (**4d**)

The title compound was prepared according to the general procedure using methyl 1-(4-bromo-2-nitrophenyl)-4-oxocyclohexane-1-carboxylate (64 mg, 0.18 mmol) and potassium carbonate (249 mg, 1.80 mmol, 10 equiv) in *N*-methylpyrrolidinone (18 mL, 0.01 M). Purification by chromatography (CH_2_Cl_2_) gave **4d** (32 mg, 55%) as a yellow solid; ^1^H NMR (400 MHz, CDCl_3_) δ 6.88–6.79 (m, 2H), 6.75 (dd, *J* = 1.6, 0.6 Hz, 1H), 4.58 (s, 1H), 3.87–3.80 (m, 1H), 3.76 (s, 3H), 2.57 (dt, *J* = 13.3, 3.7 Hz, 1H), 2.44–2.36 (m, 1H), 2.36–2.23 (m, 3H), 2.17 (dd, *J* = 13.3, 2.9 Hz, 1H) ppm; ^13^C{^1^H} NMR (101 MHz, CDCl_3_) δ 207.7, 174.8, 143.1, 128.4, 122.4, 121.2, 120.0, 116.9, 57.0, 52.7, 44.5, 38.6, 34.5, 32.6 ppm. HRMS (ESI) *m*/*z* [M + H]^+^: calcd for C_14_H_15_BrNO_3_^+^ 324.0230; found, 324.0215.

#### Methyl 8-Fluoro-3-oxo-2,3,4,5-tetrahydro-2,6-methanobenzo[*b*]azocine-6(1*H*)-carboxylate (**4e**)

The title compound was prepared according to the general procedure using methyl 1-(5-fluoro-2-nitrophenyl)-4-oxocyclohexane-1-carboxylate (53 mg, 0.18 mmol) and potassium carbonate (249 mg, 1.80 mmol, 10 equiv) in *N*-methylpyrrolidinone (18 mL, 0.01 M). Purification by chromatography (CH_2_Cl_2_) gave **4e** (34 mg, 71%) as a yellow solid; ^1^H NMR (400 MHz, CDCl_3_) δ 6.85 (ddd, *J* = 8.8, 8.0, 2.9 Hz, 1H), 6.75 (dd, *J* = 9.5, 2.8 Hz, 1H), 6.53 (dd, *J* = 8.8, 4.7 Hz, 1H), 3.82 (ddt, *J* = 3.6, 2.9, 1.3 Hz, 1H), 3.78 (s, 3H), 2.59 (dt, *J* = 13.3, 3.7 Hz, 1H), 2.47–2.40 (m, 1H), 2.38–2.21 (m, 3H), 2.18 (dd, *J* = 13.3, 2.9 Hz, 1H) ppm; ^13^C{^1^H} NMR (101 MHz, CDCl_3_) δ 208.0, 174.7, 157.1, 154.7, 138.1, 121.8, 116.1, 115.8, 115.34, 115.26, 113.4, 113.2, 57.2, 52.8, 44.5, 38.6, 34.7, 32.6 ppm; ^19^F NMR (376 MHz, CDCl_3_) δ −126.05 (td, *J* = 8.8, 4.7 Hz) ppm. HRMS (ESI) *m*/*z* [M + H]^+^: calcd for C_14_H_15_FNO_3_^+^, 264.1030; found, 264.1031.

#### Methyl 10-Fluoro-3-oxo-2,3,4,5-tetrahydro-2,6-methanobenzo[*b*]azocine-6(1*H*)-carboxylate (**4f**)

The title compound was prepared according to the general procedure using methyl 1-(3-fluoro-2-nitrophenyl)-4-oxocyclohexane-1-carboxylate (53 mg, 0.18 mmol) and potassium carbonate (249 mg, 1.80 mmol, 10 equiv) in *N*-methylpyrrolidinone (18 mL, 0.01 M). Purification by chromatography (CH_2_Cl_2_) gave **4f** (35 mg, 73%) as a yellow solid; ^1^H NMR (400 MHz, CDCl_3_) δ 6.95 (ddd, *J* = 11.0, 8.1, 1.3 Hz, 1H), 6.78 (dt, *J* = 7.8, 1.1 Hz, 1H), 6.64 (td, *J* = 8.0, 5.3 Hz, 1H), 4.68 (s, 1H), 3.97–3.92 (m, 1H), 3.77 (s, 3H), 2.62 (dt, *J* = 13.4, 3.8 Hz, 1H), 2.49–2.37 (m, 1H), 2.38–2.24 (m, 3H), 2.24–2.17 (m, 1H) ppm; ^13^C{^1^H} NMR (126 MHz, CDCl_3_) δ 207.6, 174.8, 151.5, 149.5, 130.8, 130.7, 123.21, 123.19, 122.03, 122.01, 117.3, 117.2, 114.3, 114.1, 56.5, 52.7, 44.6, 44.5, 38.7, 34.6, 32.7 ppm; ^19^F NMR (471 MHz, CDCl_3_) δ −137.17 (dd, *J* = 11.1, 5.3 Hz) ppm. HRMS (ESI) *m*/*z* [M + H]^+^: calcd for C_14_H_15_FNO_3_^+^, 264.1036; found, 264.1031.

#### Methyl 9-Methoxy-3-oxo-2,3,4,5-tetrahydro-2,6-methanobenzo[*b*]azocine-6(1*H*)-carboxylate (**4g**)

The title compound was prepared according to the general procedure using methyl 1-(4-methoxy-2-nitrophenyl)-4-oxocyclohexane-1-carboxylate (55 mg, 0.18 mmol) and potassium carbonate (249 mg, 1.80 mmol, 10 equiv) in *N*-methylpyrrolidinone (18 mL, 0.01 M). Purification by chromatography (CH_2_Cl_2_) gave **4g** (38 mg, 76%) as a yellow solid; ^1^H NMR (400 MHz, CDCl_3_) δ 6.88 (d, *J* = 8.6 Hz, 1H), 6.31 (dd, *J* = 8.6, 2.5 Hz, 1H), 6.12 (d, *J* = 2.5 Hz, 1H), 3.81 (t, *J* = 3.3 Hz, 1H), 3.76 (s, 3H), 3.75 (s, 3H), 2.60 (dt, *J* = 13.2, 3.7 Hz, 1H), 2.46–2.35 (m, 1H), 2.36–2.19 (m, 3H), 2.16 (dd, *J* = 13.2, 2.9 Hz, 1H); ^13^C{^1^H} NMR (126 MHz, CDCl_3_) δ 208.4, 175.4, 160.2, 142.8, 127.9, 113.9, 104.7, 99.3, 57.3, 55.3, 52.6, 44.2, 38.8, 34.7, 33.2 ppm. HRMS (ESI) *m*/*z* [M + H]^+^: calcd for C_15_H_18_NO_4_^+^, 276.1236; found, 276.1236.

#### Methyl 3-Oxo-2,3-dihydro-2,6-methanobenzo[*b*]azocine-6(1*H*)-carboxylate (**4h**)

The title compound was prepared according to the general procedure using methyl 1-(2-nitrophenyl)-4-oxocyclohex-2-ene-1-carboxylate (275 mg, 1.00 mmol) and potassium carbonate (1.38 g, 9.98 mmol, 10 equiv) in *N*-methylpyrrolidinone (100 mL, 0.01 M). Purification by chromatography (CH_2_Cl_2_) gave **4h** (131 mg, 54%) as a yellow oil; ^1^H NMR (400 MHz, CDCl_3_) δ 7.45 (dd, *J* = 10.1, 2.1 Hz, 1H), 7.12 (ddd, *J* = 8.2, 7.3, 1.5 Hz, 1H), 6.91 (dd, *J* = 7.8, 1.5 Hz, 1H), 6.69 (ddd, *J* = 7.9, 7.3, 1.2 Hz, 1H), 6.61 (ddd, *J* = 8.1, 1.3, 0.5 Hz, 1H), 6.22 (dd, *J* = 10.1, 1.2 Hz, 1H), 4.74 (s, 1H), 4.03–3.96 (m, 1H), 3.87 (s, 3H), 2.51–2.42 (m, 1H), 2.42–2.33 (m, 1H) ppm; ^13^C{^1^H} NMR (101 MHz, CDCl_3_) δ 193.8, 173.0, 151.2, 140.1, 129.4, 128.1, 125.8, 117.9, 117.6, 115.2, 53.8, 53.0, 46.5, 29.9 ppm. HRMS (ESI) *m*/*z* [M + H]^+^: calcd for C_14_H_14_NO_3_^+^, 244.0968; found, 244.0965.

### Synthesis of 1-(*tert*-Butyl) 6-Methyl 3-((*tert*-Butoxycarbonyl)oxy)-2,5-dihydro-2,6-methanobenzo[*b*]azocine-1,6-dicarboxylate (**5**)

A solution of benzazocine **4a** (49 mg, 0.20 mmol) and di-*tert*-butyl dicarbonate (131 mg, 0.60 mmol, 3 equiv) in anhydrous tetrahydrofuran (3 mL, 0.07 M) was cooled to 0 °C. Then, a 1.0 M solution of LiHMDS in THF (0.4 mL, 0.40 mmol, 2 equiv) was added dropwise, and the mixture was stirred at room temperature for 2 h. After this time, the solution was quenched with water and subsequently dried with sodium sulfate. The resulting mixture was filtered and concentrated in vacuo. Purification by chromatography (hexane/EtOAc 95:5 → hexane/EtOAc 75:25) gave **5** as a yellow oil (45 mg, 51%); ^1^H NMR (400 MHz, CDCl_3_) δ 7.87 (d, *J* = 8.5 Hz, 1H), 7.19 (ddd, *J* = 8.6, 7.1, 1.7 Hz, 1H), 7.08 (dd, *J* = 7.8, 1.7 Hz, 1H), 6.99 (ddd, *J* = 8.1, 7.1, 1.3 Hz, 1H), 5.50–5.43 (m, 2H), 3.72 (s, 3H), 2.86 (dt, *J* = 17.8, 1.6 Hz, 1H), 2.58–2.46 (m, 2H), 2.16 (dd, *J* = 12.7, 3.7 Hz, 1H), 1.55 (s, 9H), 1.51 (s, 9H) ppm; ^13^C{^1^H} NMR (101 MHz, CDCl_3_) δ 175.5, 152.9, 151.9, 145.2, 136.5, 128.3, 127.5, 126.6, 124.2, 123.8, 116.0, 83.4, 81.8, 52.7, 49.0, 45.1, 37.4, 32.4, 28.5, 27.9 ppm. HRMS (ESI) *m*/*z* [M + H]^+^: calcd for C_24_H_32_NO_7_^+^, 446.2173; found, 446.2175.

### Synthesis of Methyl 1′,2′,4′,5′-Tetrahydro-6′*H*-spiro[[1,3]dioxolane-2,3′-[2,6]methanobenzo[*b*]azocine]-6′-carboxylate (**6**)

A solution of benzazocine **4a** (123 mg, 0.50 mmol) and *p*-toluenesulfonic acid monohydrate (10 mg, 0.05 mmol, 0.1 equiv) in 2-ethyl-2-methyl-1,3-dioxolane (3 mL, 0.17 M) was stirred at room temperature overnight. Afterward, the reaction was quenched with solid potassium carbonate, filtered, and concentrated in vacuo. Purification by chromatography (hexane/EtOAc 99:1 → hexane/EtOAc 50:50) gave **6** as a yellow oil (106 mg, 73%); ^1^H NMR (400 MHz, CDCl_3_) δ 7.04 (ddd, *J* = 8.0, 7.2, 1.5 Hz, 1H), 6.84 (dd, *J* = 7.7, 1.5 Hz, 1H), 6.61 (ddd, *J* = 7.5, 7.4, 1.2 Hz, 1H), 6.55 (dd, *J* = 8.1, 1.2 Hz, 1H), 4.05–3.88 (m, 4H), 3.74 (s, 3H), 3.31–3.25 (m, 1H), 2.34 (ddd, *J* = 12.8, 3.8, 2.8 Hz, 1H), 2.22 (dd, *J* = 12.8, 2.8 Hz, 1H), 2.17–1.98 (m, 2H), 1.57–1.48 (m, 2H) ppm; ^13^C{^1^H} NMR (101 MHz, CDCl_3_) δ 176.2, 143.5, 128.2, 126.5, 122.3, 117.3, 114.2, 110.2, 65.3, 64.6, 52.24, 52.16, 44.7, 35.3, 30.2, 28.0 ppm. HRMS (ESI) *m*/*z* [M + H]^+^: calcd for C_16_H_20_NO_4_^+^, 290.1387; found, 290.1389.

### Synthesis of 1′-(*tert*-Butyl) 6′-Methyl 4′,5′-Dihydro-2′*H*-spiro[[1,3]dioxolane-2,3′-[2,6]methanobenzo[*b*]azocine]-1′,6′-dicarboxylate (**7**)

A solution of benzazocine **6** (58 mg, 0.20 mmol) and di-*tert*-butyl dicarbonate (131 mg, 0.60 mmol, 3 equiv) in anhydrous tetrahydrofuran (3 mL, 0.07 M) was cooled to 0 °C. Then, a 1.0 M solution of LiHMDS in THF (0.4 mL, 0.40 mmol, 2 equiv) was added dropwise, and the mixture was stirred at room temperature for 2 h. After this time, the solution was quenched with water and subsequently dried with sodium sulfate. The resulting mixture was filtered and concentrated in vacuo. Purification by chromatography (hexane → hexane/EtOAc 75:25) gave **7** as a colorless oil (71 mg, 91%); ^1^H NMR (400 MHz, CDCl_3_) δ 8.11 (dd, *J* = 8.6, 1.1 Hz, 1H), 7.19 (ddd, *J* = 8.7, 6.6, 2.3 Hz, 1H), 7.00–6.89 (m, 2H), 4.70–4.65 (m, 1H), 4.13–3.98 (m, 3H), 3.98–3.90 (m, 1H), 3.73 (s, 3H), 2.38 (dt, *J* = 13.0, 3.4 Hz, 1H), 2.20 (dd, *J* = 13.0, 3.2 Hz, 1H), 2.11 (td, *J* = 13.6, 4.3 Hz, 1H), 1.95 (ddt, *J* = 13.3, 5.3, 2.6 Hz, 1H), 1.56 (s, 9H), 1.54–1.47 (m, 1H), 1.24 (td, *J* = 14.0, 5.2 Hz, 1H) ppm; ^13^C{^1^H} NMR (101 MHz, CDCl_3_) δ 176.0, 154.0, 137.9, 127.4, 126.9, 126.2, 123.0, 122.4, 108.2, 81.8, 65.2, 65.1, 53.1, 52.4, 45.8, 35.1, 30.5, 29.2, 28.5 ppm. HRMS (ESI) *m*/*z* [M + H]^+^: calcd for C_21_H_28_NO_6_^+^, 390.1911; found, 390.1910.

## Supporting Information Available

X-ray crystallographic data for compound **4e**; copies of NMR spectra, (PDF)
FAIR data, including the primary NMR FID files, for compounds **1a**–**4h** (ZIP)

## References

[ref1] Gloer J. B. Antiinsectan natural products from fungal sclerotia. Acc. Chem. Res. 1995, 28, 343–350. 10.1021/ar00056a004.

[ref2] Gloer J. B.; Rinderknecht B. L.; Wicklow D. T.; Dowd P. F. Nominine: a new insecticidal indole diterpene from the sclerotia of *Aspergillus nomius*. J. Org. Chem. 1989, 54, 2530–2532. 10.1021/jo00272a012.

[ref3] Sun Y.; Chen P.; Zhang D.; Baunach M.; Hertweck C.; Li A. Bioinspired Total Synthesis of Sespenine. Angew. Chem., Int. Ed. 2014, 53, 9012–9016. 10.1002/anie.201404191. 24962149

[ref4] Bradshaw B.; Etxebarría-Jardí G.; Bonjoch J. Total Synthesis of (−)-Anominine. J. Am. Chem. Soc. 2010, 132, 5966–5967. 10.1021/ja101994q. 20384301

[ref5] aBian M.; Wang Z.; Xiong X.; Sun Y.; Matera C.; Nicolaou K. C.; Li A. Total Syntheses of Anominine and Tubingensin A. J. Am. Chem. Soc. 2012, 134, 8078–8081. 10.1021/ja302765m. 22537293

[ref6] Staub G. M.; Gloer J. B.; Wicklow D. T.; Dowd P. F. Aspernomine: A Cytotoxic Antiinsectan Metabolite with a Novel Ring System from the sclerotia of *Aspergillus Nomius*. J. Am. Chem. Soc. 1992, 114, 1015–1017. 10.1021/ja00029a033.

[ref7] Quetin-Leclercq J.; Angenot L.; Dupont L.; Dideberg O.; Warin R.; Delaude C.; Coune C. Revision of the Structure of Strychnochromine. Tetrahedron Lett. 1991, 32, 4295–4298. 10.1016/S0040-4039(00)92152-X.

[ref8] aAlthough the benzazocine core ring has been synthesized, the strategies employed would be difficult to extrapolate to the total synthesis of complex natural products (see Figure 1). For examples, see: Solé D.; Vallverdú L.; Bonjoch J. Palladium-Catalyzed Intramolecular Coupling of Aryl Halides and Ketone Enolates: Synthesis of Hexahydro-2,6-methano-1-benzazocines. Adv. Synth. Catal. 2001, 343, 439–442. 10.1002/1615-4169(200107)343:5<439::AID-ADSC439>3.0.CO;2-G.

[ref9] Solé D.; Parés A.; Bonjoch J. An Unexpected Transformation by Reaction of Congested α-(*o*-Nitrophenyl)ketones with Tris(dimethylamino)methane. A New Heterocyclic System: 6,11b-Methanopyrrolo [2,3-*e*][1]benzazocine. Tetrahedron. 1994, 50, 9769–9774. 10.1016/S0040-4020(01)85542-3.

[ref10] Solé D.; García-Rubio S.; Vallverdú L.; Bonjoch J. A Straightforward Synthetic Entry to the 4,9b-Propanopyrrolo[2,3-c]quinoline System by a New Reductive Cyclization of α’-(2-Nitrophenyl) Enones. J. Org. Chem. 2001, 66, 5266–5268. 10.1021/jo015659h. 11463287

[ref11] aLotesta S. D.; Marcus A. P.; Zheng Y.; Leftheris K.; Noto P. B.; Meng S.; Kandpal G.; Chen G.; Zhou J.; McKeever B.; Bukhtiyarov Y.; Zhao Y.; Lala D. S.; Singh S. B.; McGeehan G. M. Identification of Spirooxindole and Dibenzoxazepine Motifs as Potent Mineralocorticoid Receptor Antagonists. Bioorg. Med. Chem. 2016, 24, 1384–1391. 10.1016/j.bmc.2016.02.014. 26897089

[ref12] aYoshida M.; Maeyama Y.; Shishido K. Regio- and enantioselective synthesis of functionalized tetrahydroquinolines by palladium-catalyzed cyclization of 2-amidophenylmalonates with allylic bisacetates. Tetrahedron. 2012, 68, 9962–9972. 10.1016/j.tet.2012.09.075.

[ref13] Danishefsky S.; Kitahara T. A Useful Diene for the Diels-Alder Reaction. J. Am. Chem. Soc. 1974, 96, 7807–7808. 10.1021/ja00832a031.

[ref14] aParvatkar P. T.; Majik M. S. Microwave-assisted reductive cyclization: an easy entry to the indoquinolines and spiro[2H-indole-2,3′-oxindole]. RSC Adv. 2014, 4, 22481–22486. 10.1039/c4ra02814g.

[ref15] Zuo Z.; Xie W.; Ma D. Total Synthesis and Absolute Stereochemical Assignment of (−)-Communesin F. J. Am. Chem. Soc. 2010, 132, 13226–13228. 10.1021/ja106739g. 20812683

[ref16] aHu W. P.; Wang J. J.; Tsai P. C. Novel Examples of 3-Aza-Grob Fragmentation. J. Org. Chem. 2000, 65, 4208–4209. 10.1021/jo000252i. 10866646

[ref17] aKarnik A. V.; Hasan M. Stereochemistry: A Three-Dimensional Insight 2021, 273–275. 10.1016/B978-0-12-821062-8.00010-7.

[ref18] Santos M. M. M.; Moreira R. Michael Acceptors as Cysteine Protease Inhibitors. Mini-Rev. Med. Chem. 2007, 7, 1040–1050. 10.2174/138955707782110105. 17979807

[ref19] Ward J. A.; Pinto-Fernandez A.; Cornelissen L.; Bonham S.; Díaz-Sáez L.; Riant O.; Huber K. V. M.; Kessler B. M.; Feron O.; Tate E. W. Re-Evaluating the Mechanism of Action of α,β-Unsaturated Carbonyl DUB Inhibitors b-AP15 and VLX1570: A Paradigmatic Example of Unspecific Protein Cross-linking with Michael Acceptor Motif-Containing Drugs. J. Med. Chem. 2020, 63, 3756–3762. 10.1021/acs.jmedchem.0c00144. 32109059 PMC7152998

[ref20] aHarvey R. D.; Adams V. R.; Beardslee T.; Medina P. Afatinib for the treatment of *EGFR* mutation-positive NSCLC: A review of clinical findings. J. Oncol. Pharm. Pract. 2020, 26, 1461–1474. 10.1177/1078155220931926. 32567494 PMC7448811

[ref21] aFor experimental procedures to prepare **1b**–**f**, see: Gao M.; Luo Y.; Xu Q.; Zhao Y.; Gong X.; Xia Y.; Hu L. A Unified Catalytic Asymmetric (4 + 1) and (5 + 1) Annulation Strategy to Access Chiral Spirooxindole-Fused Oxacycles. Angew. Chem., Int. Ed. 2021, 60, 19813–19820. 10.1002/anie.202105282. 34160121

[ref22] Shevlin M.; Strotman N. A.; Anderson L. L. Concise Synthesis of Furo[2,3,*b*]indolines via [3,3]-Sigmatropic Rearrangement of *N*-Alkenyloxyindoles. Synlett. 2021, 32, 197–201. 10.1055/s-0040-1707250.

