# Supplementary material for: Base-Mediated Nitrophenyl Reductive Cyclization for the Synthesis of Hexahydro-2,6-methano-1-benzazocines
Source: J Org Chem. 2022 Nov 4;87(22):15693–702. doi: 10.1021/acs.joc.2c02205 (PMC9680033; doi:10.1021/acs.joc.2c02205)
Supplement: Supplementary file 1 — jo2c02205_si_001.pdf [file jo2c02205_si_001.pdf]

Supporting Information

Base-mediated Nitrophenyl Reductive Cyclization for the Synthesis of Hexahydro-2,6-methano-1-benzazocines

Laura G. Rodríguez,[†] Ana Delgado,[‡] Carlos J. Ciudad,[‡] Véronique Noé,[‡] Josep Bonjoch,[*,†] and Ben Bradshaw[*,†]

[†]Laboratori de Química Orgànica, Facultat de Farmàcia, IBUB, Universitat de Barcelona, Av. Joan XXIII 27-31, 08028, Barcelona, Spain

[‡]Department of Biochemistry and Physiology, Faculty of Pharmacy & IN2UB, University of Barcelona, Av. Joan XXIII 27-31, 08028, Barcelona, Spain

Contents

X-Ray Crystallographic Data for compound **4e** S2

Copies of NMR spectra S5

X-Ray Crystallographic Data for Compound 4e

Compound **4e** was crystallized from a mixture of dichloromethane and hexane through vapor diffusion.

A yellow prism-like specimen of $C_{14}H_{14}FNO_3$, approximate dimensions 0.060 mm x 0.200 mm x 0.250 mm, was used for the X-ray crystallographic analysis. The X-ray intensity data were measured on a D8 Venture system equipped with a multilayer monochromator and a Mo microfocus (λ = 0.71073 Å).

The frames were integrated with the Bruker SAINT software package using a narrow-frame algorithm. The integration of the data using a monoclinic unit cell yielded a total of 47049 reflections to a maximum θ angle of 26.54° (0.80 Å resolution), of which 10273 were independent (average redundancy 4.580, completeness = 99.7%, R_{int} = 5.62%, R_{sig} = 4.53%) and 8314 (80.93%) were greater than 2σ(F^2). The final cell constants of a = 9.0069(16) Å, b = 9.7444(17) Å, c = 56.483(10) Å, β = 91.350(3)°, volume = 4956.0(15) $Å^3$, are based upon the refinement of the XYZ-centroids of reflections above 20 σ(I). Data were corrected for absorption effects using the Multi-Scan method (SADABS). The calculated minimum and maximum transmission coefficients (based on crystal size) are 0.6152 and 0.7454.

The structure was solved and refined using the Bruker SHELXTL Software Package, using the space group P 1 21/n 1, with Z = 16 for the formula unit, $C_{14}H_{14}FNO_3$. The final anisotropic full-matrix least-squares refinement on F^2 with 698 variables converged at R1 = 8.23%, for the observed data and wR2 = 16.61% for all data. The goodness-of-fit was 1.186. The largest peak in the final difference electron density synthesis was 0.320 $e^-/Å^3$ and the largest hole was -0.327 $e^-/Å^3$ with an RMS deviation of 0.067 $e^-/Å^3$. On the basis of the final model, the calculated density was 1.411 g/cm^3 and F(000), 2208 e^-.

Datablock D43ZB157_0m_a - ellipsoid plot

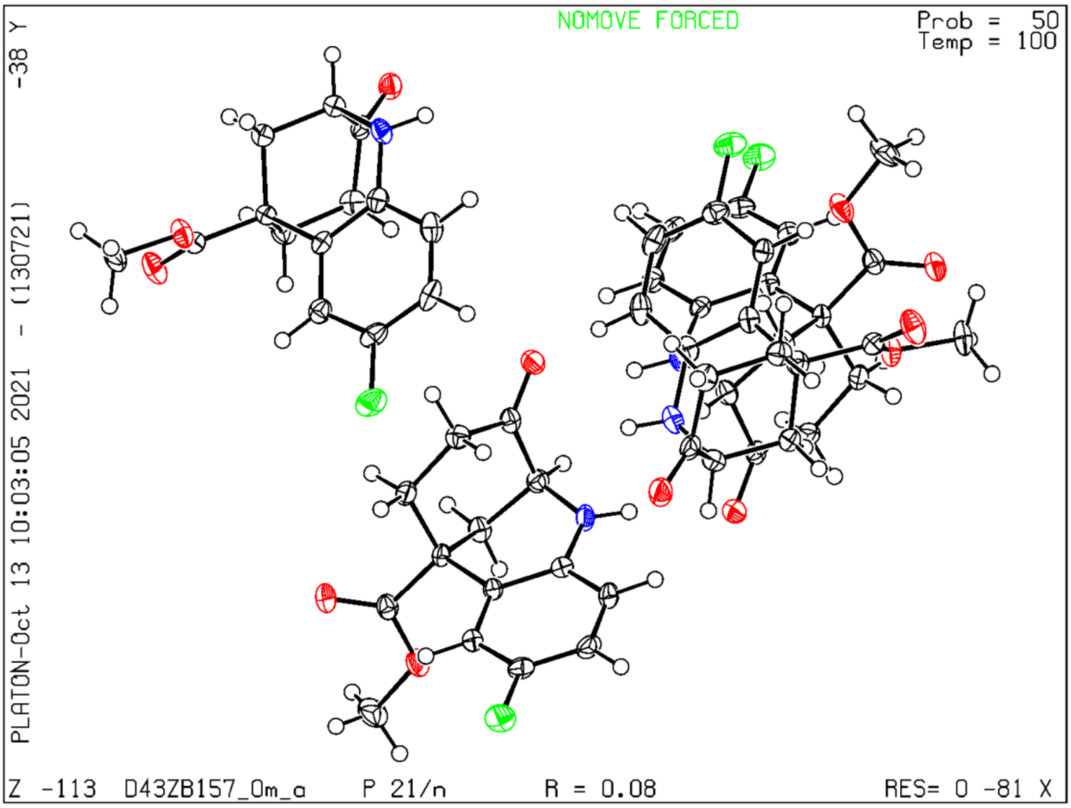

CCDC 2145263 contains the supplementary data for this paper. These data can be obtained free of charge from the Cambridge Crystallographic Data Centre via www.ccdc.cam.ac.uk/data_request/cif

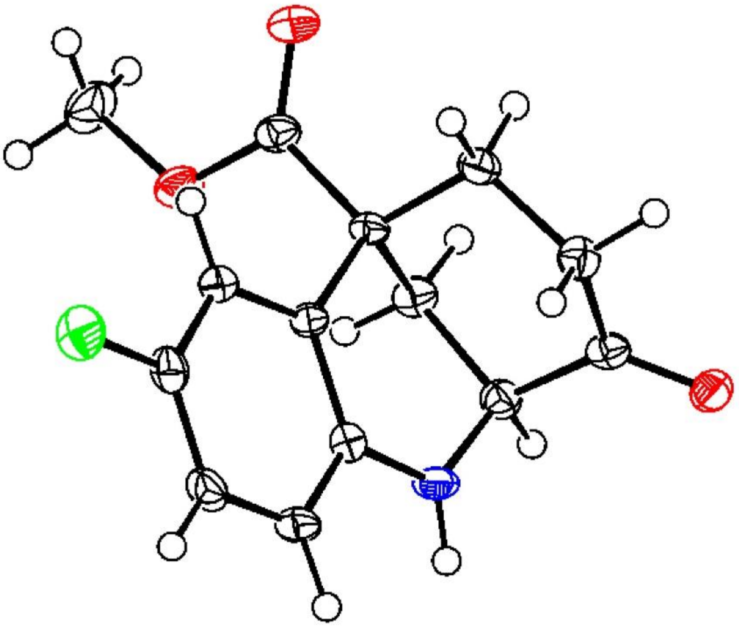

Identification code	D43ZB157_0m_a	
Empirical formula	C14 H14 F N O3	
Formula weight	263.26	
Temperature	100(2) K	
Wavelength	0.71073 Å	
Crystal system	Monoclinic	
Space group	P 21/n	
Unit cell dimensions	a = 9.0069(16) Å	α= 90°.
	b = 9.7444(17) Å	β= 91.350(3)°.
	c = 56.483(10) Å	γ = 90°.
Volume	4956.0(15) $Å^3$	
Z	16	
Density (calculated)	1.411 Mg/m^3	
Absorption coefficient	0.109 mm^{-1}	
F(000)	2208	
Crystal size	0.250 x 0.200 x 0.060 mm^3	
Theta range for data collection	2.121 to 26.539°.	
Index ranges	-11<=h<=11, -12<=k<=12, -70<=l<=70	
Reflections collected	47049	
Independent reflections	10273 [R(int) = 0.0562]	
Completeness to theta = 25.242°	99.9 %	
Absorption correction	Semi-empirical from equivalents	
Max. and min. transmission	0.7454 and 0.6152	
Refinement method	Full-matrix least-squares on F^2	
Data / restraints / parameters	10273 / 0 / 698	
Goodness-of-fit on F^2	1.186	
Final R indices [I>2sigma(I)]	R1 = 0.0823, wR2 = 0.1588	
R indices (all data)	R1 = 0.1012, wR2 = 0.1661	
Extinction coefficient	n/a	
Largest diff. peak and hole	0.320 and -0.327 e.$Å^{-3}$	

Copies of NMR spectra

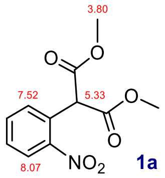

LRG265CH.10.fid 500 MHz
Register 11221/2022
Operator AGUSTIN MARTINEZ

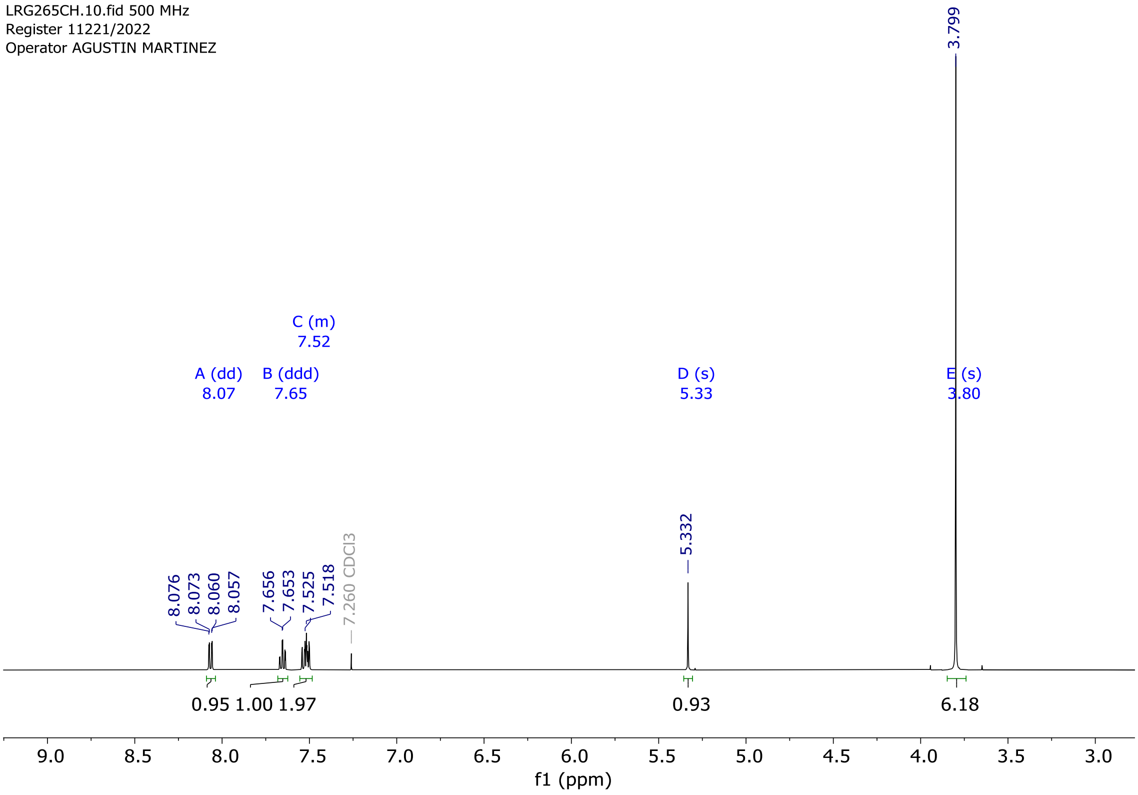

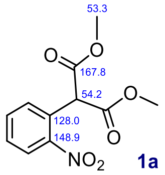

LRG265CH.11.fid 13C{1H} 126 MHz
Register 11221/2022
Operator AGUSTIN MARTINEZ

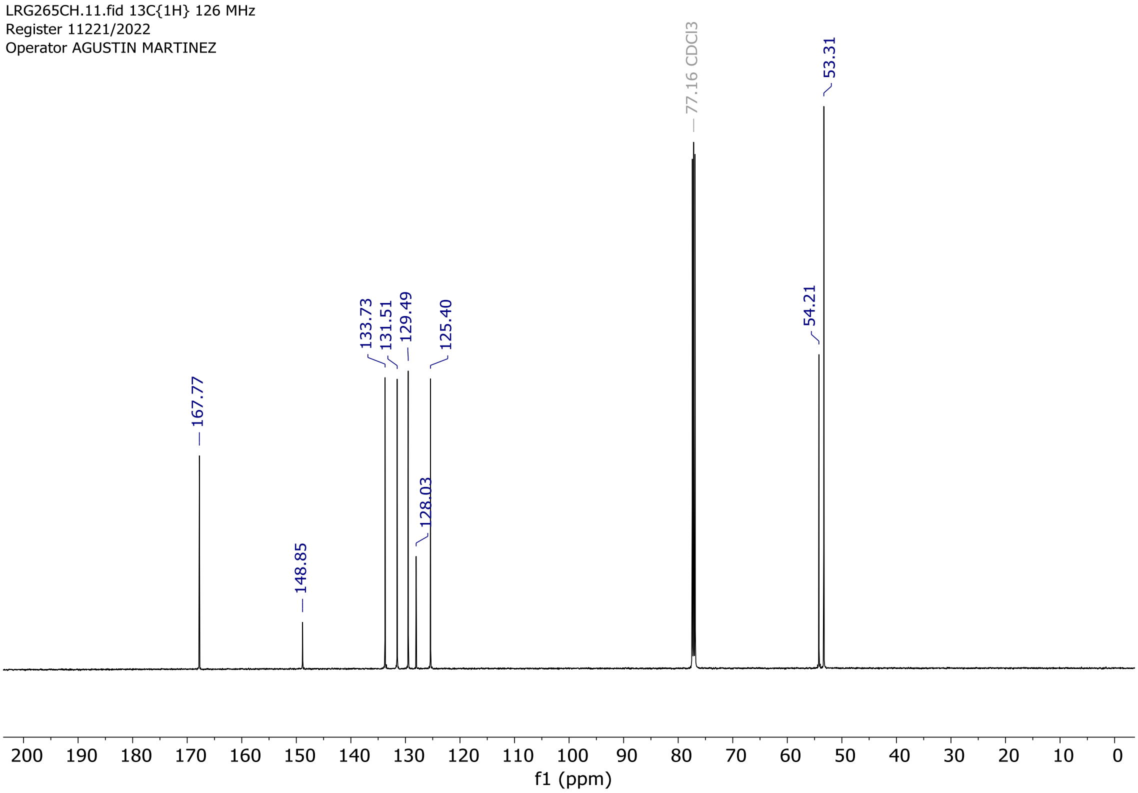

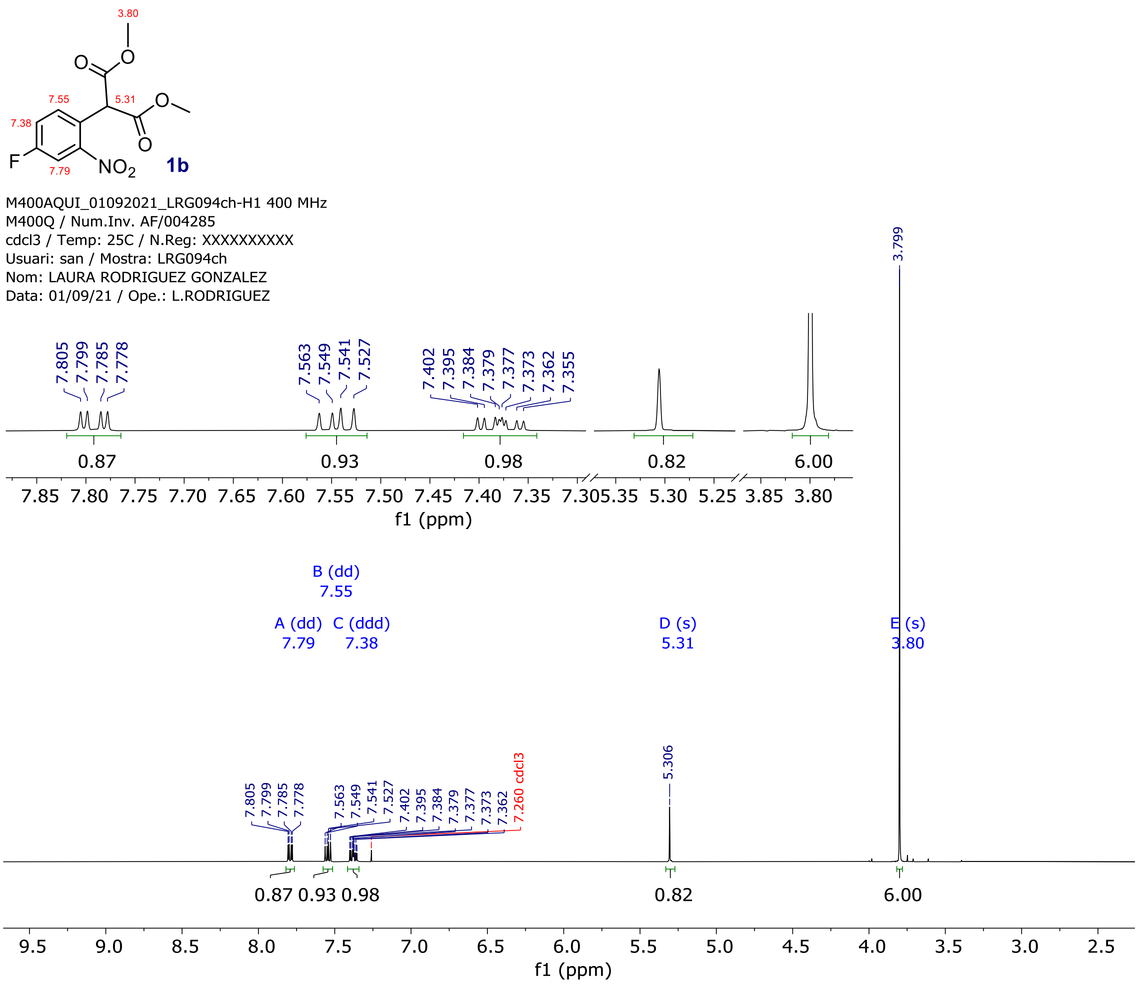
3.80
7.55
5.31
7.38
F
7.79
NO_2
1b
M400AQUI_01092021_LRG094ch-H1 400 MHz
M400Q / Num.Inv. AF/004285
cdcl3 / Temp: 25C / N.Reg: XXXXXXXXXX
Usuari: san / Mostra: LRG094ch
Nom: LAURA RODRIGUEZ GONZALEZ
Data: 01/09/21 / Ope.: L.RODRIGUEZ
3.799
7.805 7.799 7.785 7.778
7.563 7.549 7.541 7.527
7.402 7.395 7.384 7.379 7.377 7.373 7.362 7.355
0.87 0.93 0.98 0.82 6.00
f1 (ppm)
B (dd) 7.55
A (dd) 7.79
C (ddd) 7.38
D (s) 5.31
E (s) 3.80
5.306
7.260 cdcl3
0.87 0.93 0.98 0.82 6.00
f1 (ppm)

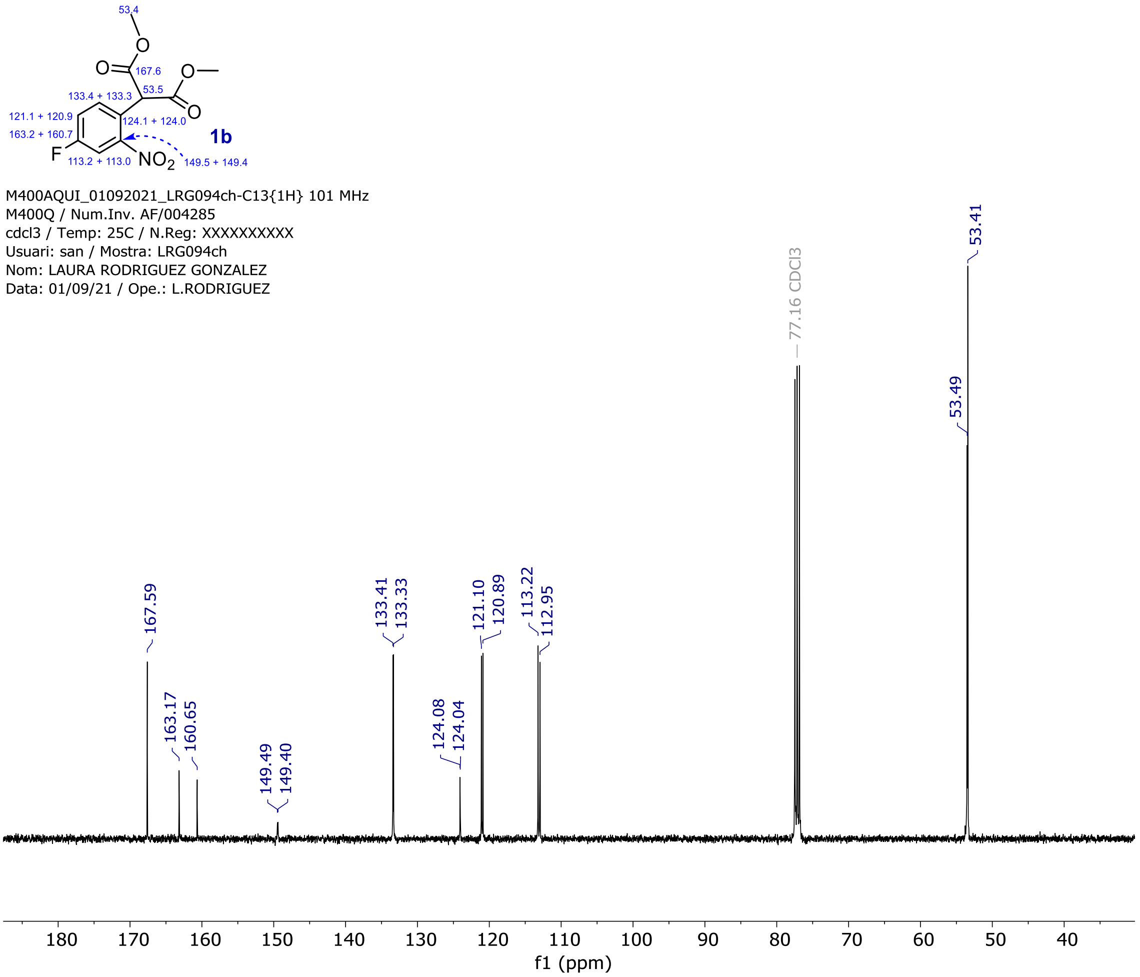
53.4
167.6
133.4 + 133.3
53.5
121.1 + 120.9
124.1 + 124.0
163.2 + 160.7
F
113.2 + 113.0
NO_2
149.5 + 149.4
1b
M400AQUI_01092021_LRG094ch-C13{1H} 101 MHz
M400Q / Num.Inv. AF/004285
cdcl3 / Temp: 25C / N.Reg: XXXXXXXXXX
Usuari: san / Mostra: LRG094ch
Nom: LAURA RODRIGUEZ GONZALEZ
Data: 01/09/21 / Ope.: L.RODRIGUEZ
77.16 CDCl3
53.41
53.49
167.59
163.17
160.65
149.49
149.40
133.41
133.33
124.08
124.04
121.10
120.89
113.22
112.95
f1 (ppm)

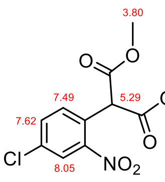

1c

M400AQUI_03092021_LRG096ch-H1 400 MHz
M400Q / Num.Inv. AF/004285
cdcl3 / Temp: 25C / N.Reg: XXXXXXXXXX
Usuari: san / Mostra: LRG096ch
Nom: LAURA RODRIGUEZ GONZALEZ
Data: 03/09/21 / Ope.: L.RODRIGUEZ

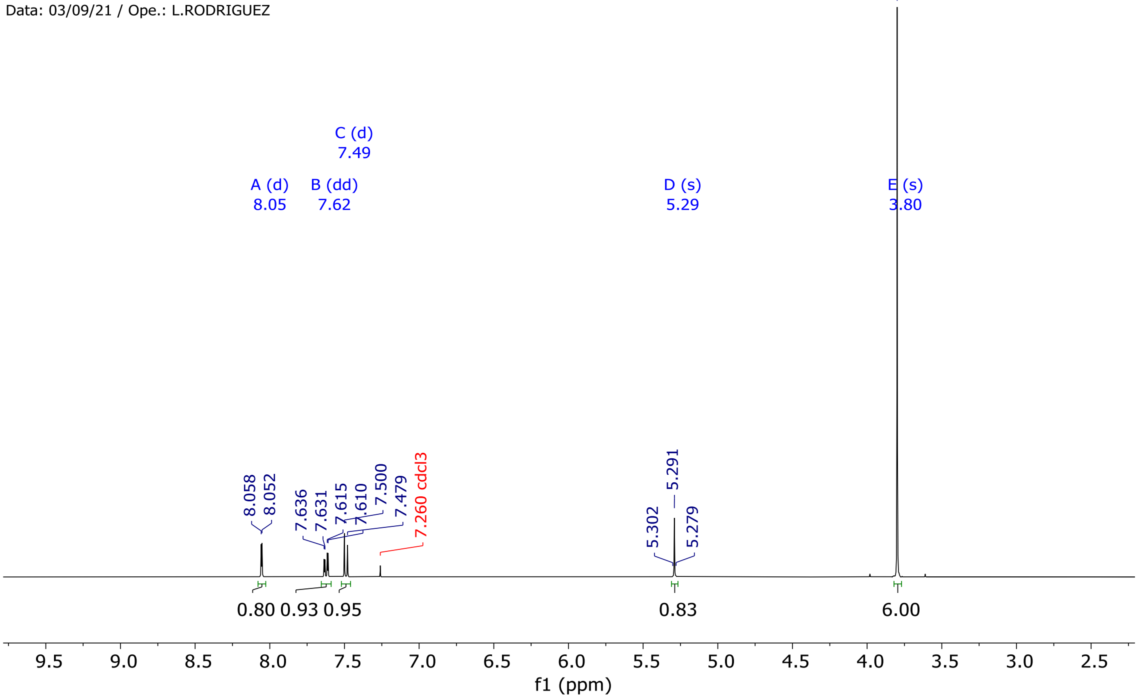

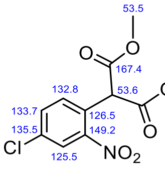

1c

M400AQUI_03092021_LRG096ch-C13{1H} 101 MHz
M400Q / Num.Inv. AF/004285
cdcl3 / Temp: 25C / N.Reg: XXXXXXXXXX
Usuari: san / Mostra: LRG096ch
Nom: LAURA RODRIGUEZ GONZALEZ
Data: 03/09/21 / Ope.: L.RODRIGUEZ

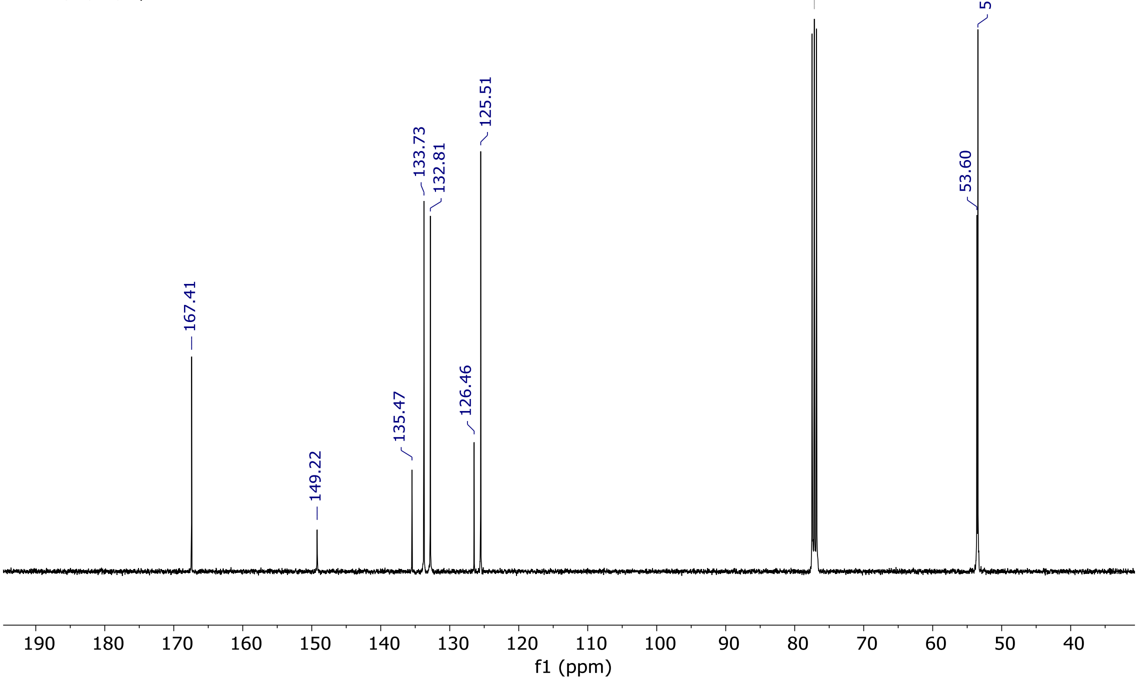

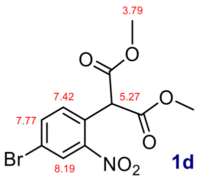

1d

M400AQUI_12072021_LRG085CHARAC-H1 400 MHz
M400Q / Num.Inv. AF/004285
cdcl3 / Temp: 25C / N.Reg: XXXXXXXXXX
Usuari: san / Mostra: LRG085CHARAC
Nom: LAURA RODRIGUEZ GONZALEZ
Data: 12/07/21 / Ope.: L.RODRIGUEZ

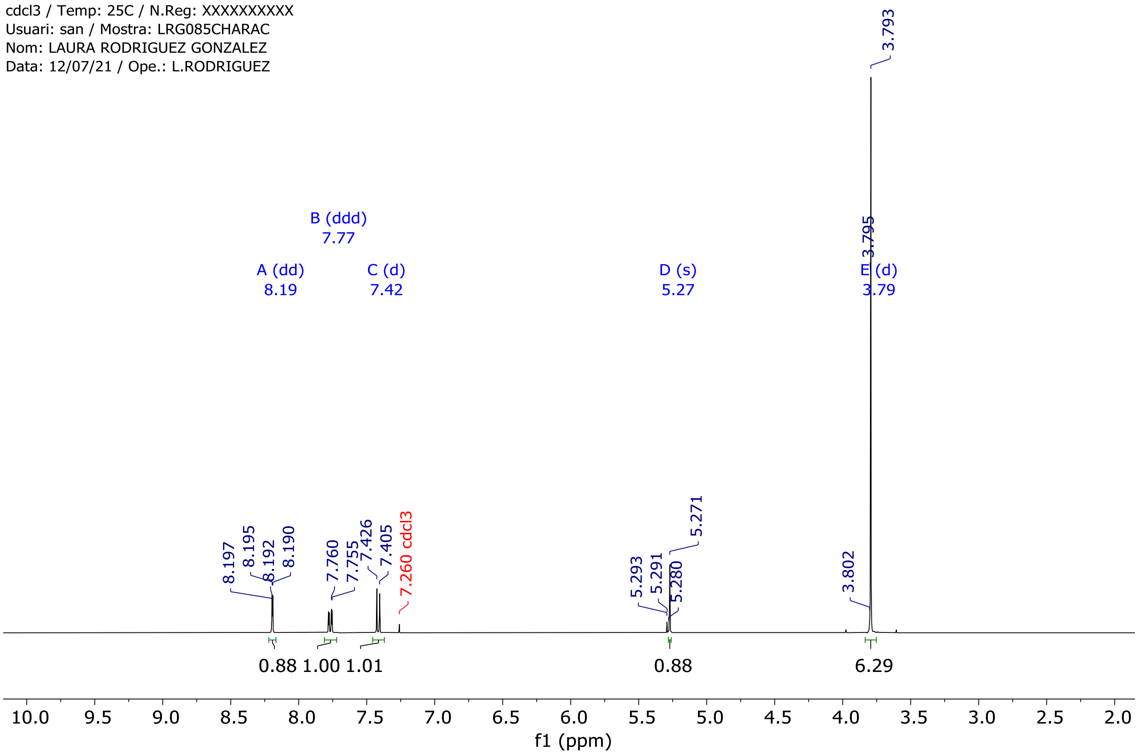

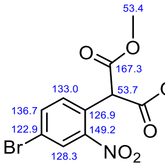

1d

M400AQUI_12072021_LRG085CHARAC-C13{1H} 101 MHz
M400Q / Num.Inv. AF/004285
cdcl3 / Temp: 25C / N.Reg: XXXXXXXXXX
Usuari: san / Mostra: LRG085CHARAC
Nom: LAURA RODRIGUEZ GONZALEZ
Data: 12/07/21 / Ope.: L.RODRIGUEZ

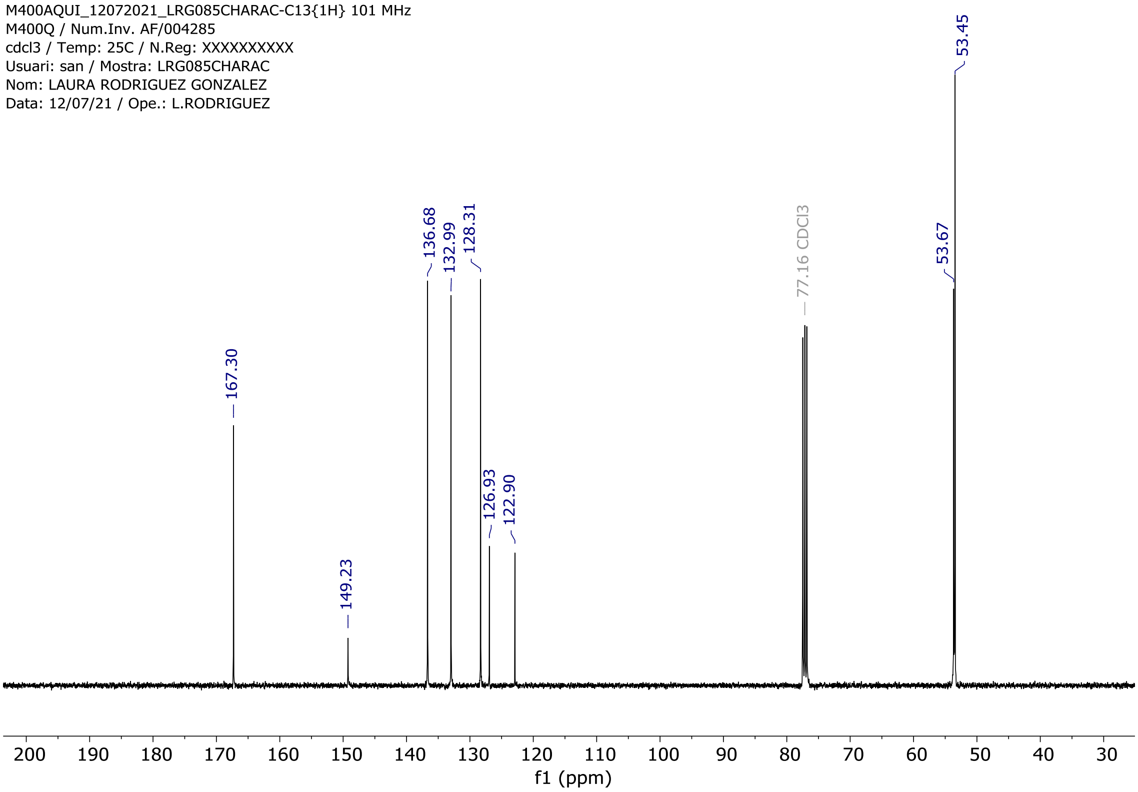

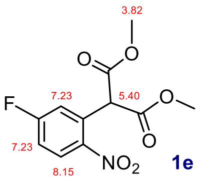

M400AFAR_08072021_LRG081PRODA-H1 400 MHz
M400F / Num.Inv. 1009191
cdcl3 / Temp: 25C / N.Reg: XXXXXXXXXX
Usuari: san / Mostra: LRG081PRODA
Nom: LAURA RODRIGUEZ GONZALEZ
Data: 08/07/21 / Ope.: L.RODRIGUEZ

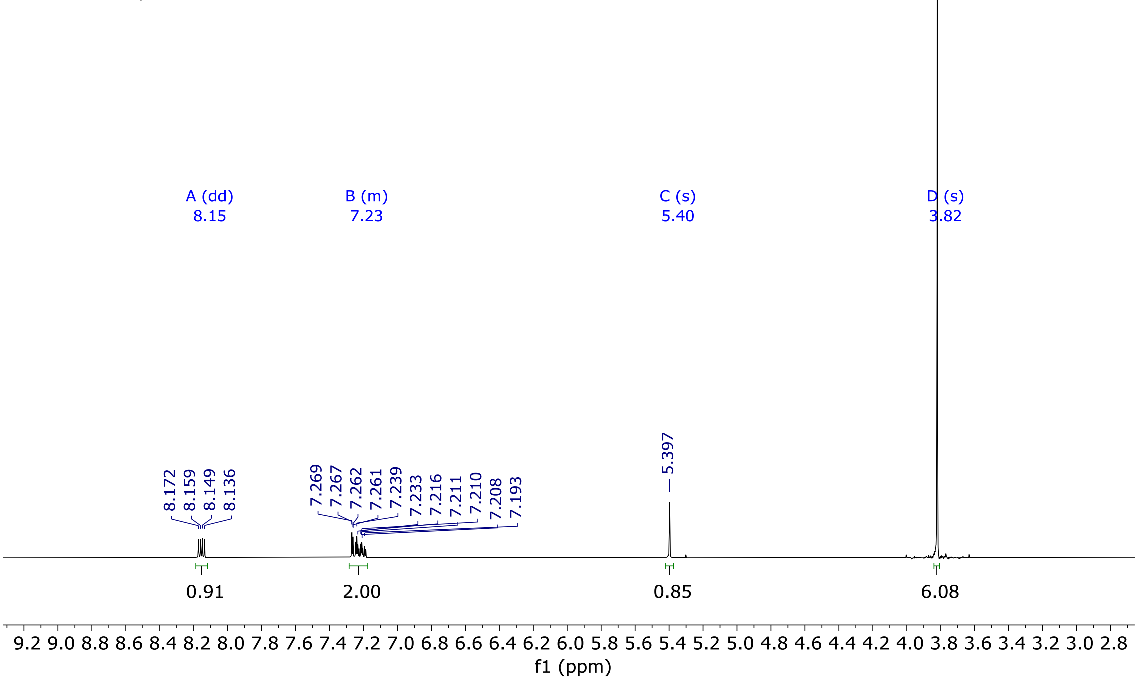

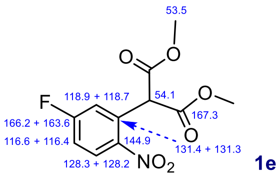

M400AQUI_08072021_LRG081CHARAC-C13{1H} 101 MHz
M400Q / Num.Inv. AF/004285
cdcl3 / Temp: 25C / N.Reg: XXXXXXXXXX
Usuari: san / Mostra: LRG081CHARAC
Nom: LAURA RODRIGUEZ GONZALEZ
Data: 08/07/21 / Ope.: L.RODRIGUEZ

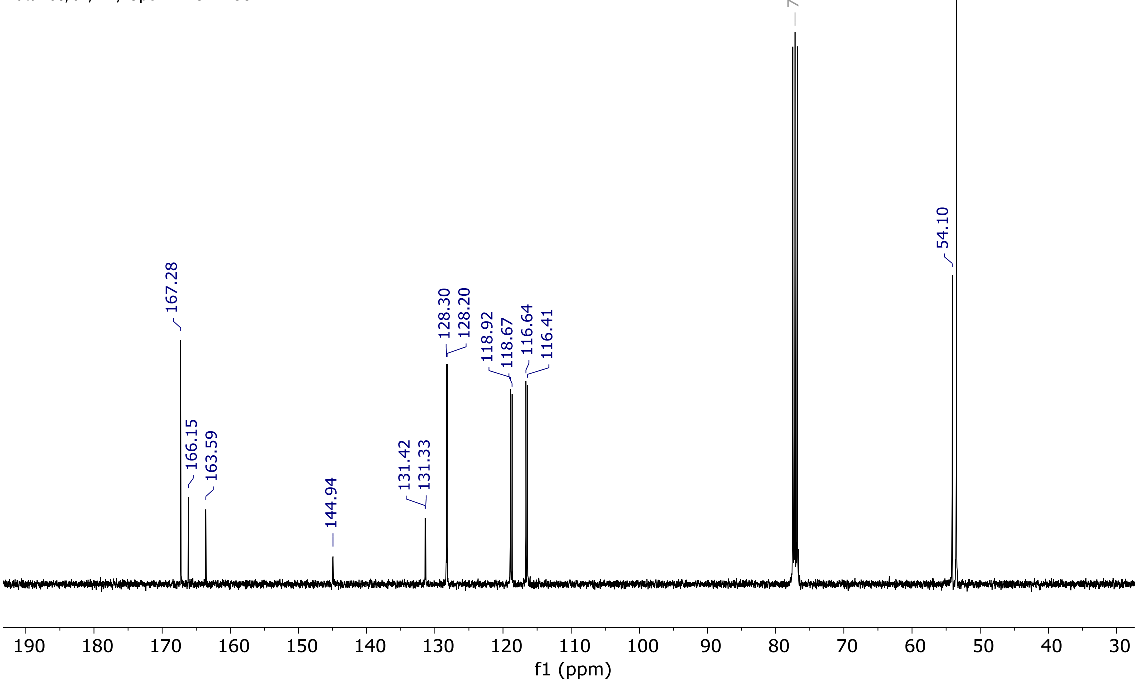

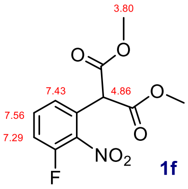

1f

M400AQUI_08092021_LRG101ch-H1 400 MHz
M400Q / Num.Inv. AF/004285
cdcl3 / Temp: 25C / N.Reg: XXXXXXXXXX
Usuari: san / Mostra: LRG101ch
Nom: LAURA RODRIGUEZ GONZALEZ
Data: 08/09/21 / Ope.: L.RODRIGUEZ

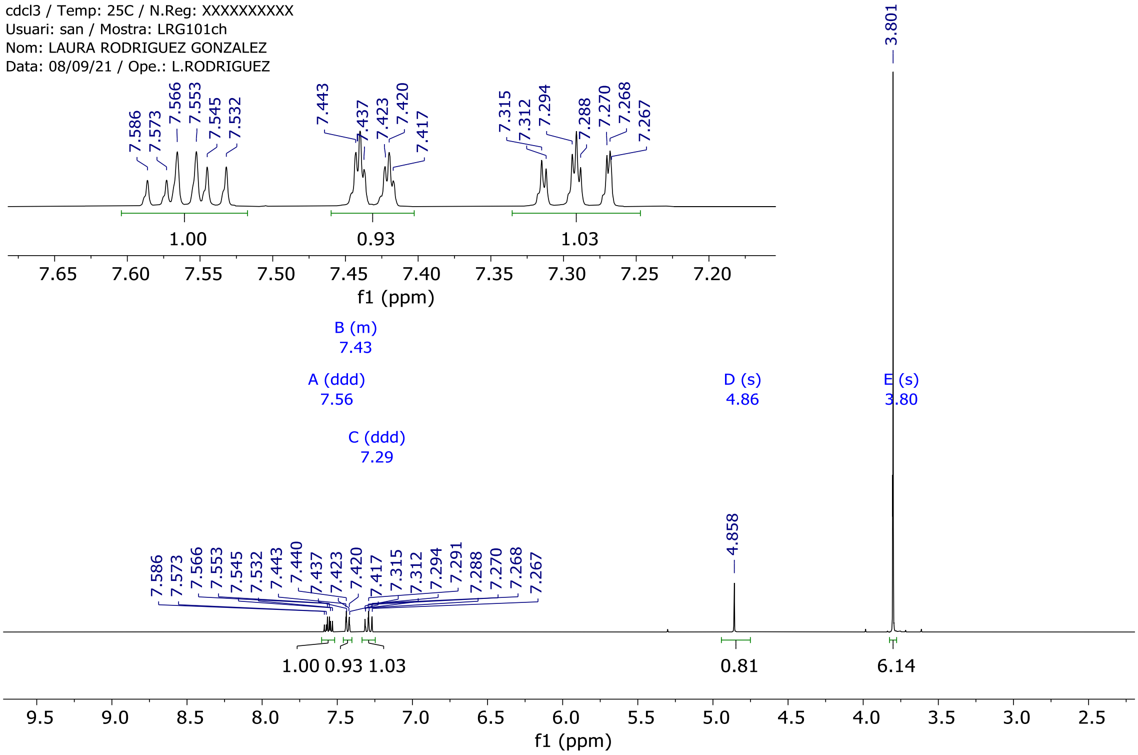

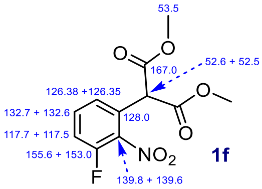

1f

M400AQUI_08092021_LRG101ch-C13{1H} 101 MHz
M400Q / Num.Inv. AF/004285
cdcl3 / Temp: 25C / N.Reg: XXXXXXXXXX
Usuari: san / Mostra: LRG101ch
Nom: LAURA RODRIGUEZ GONZALEZ
Data: 08/09/21 / Ope.: L.RODRIGUEZ

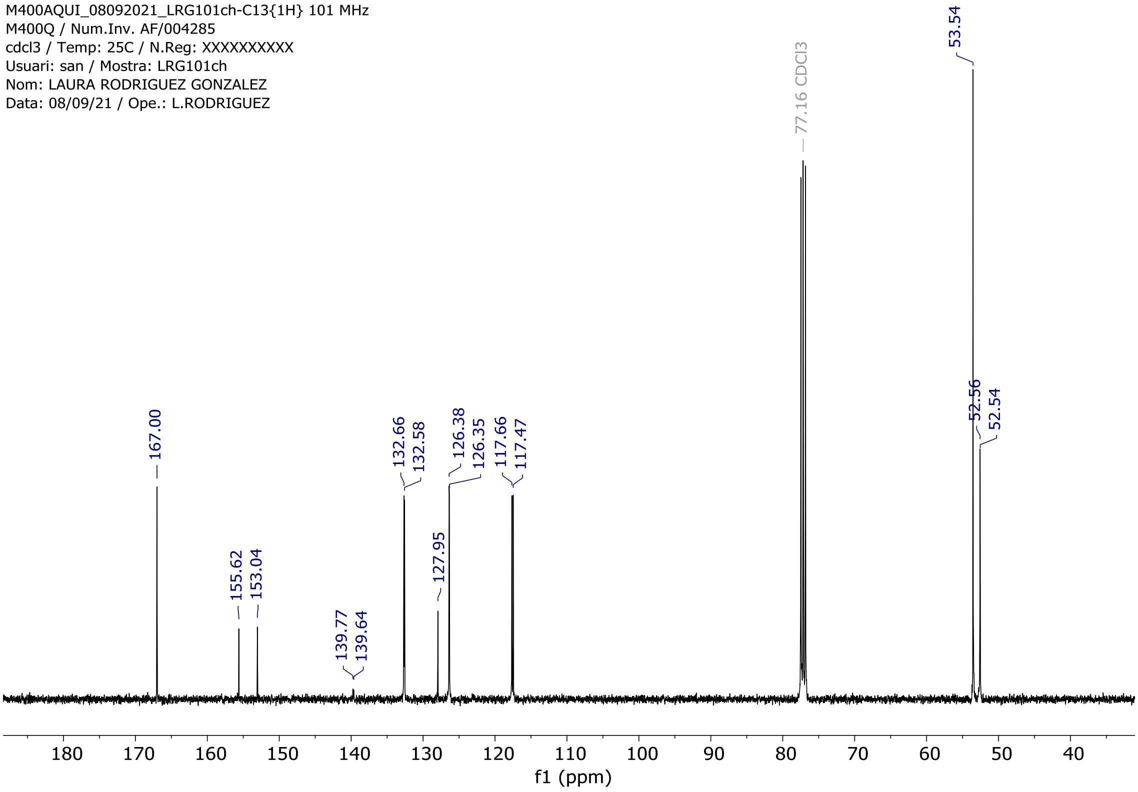

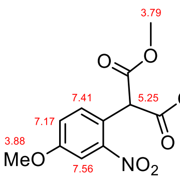

1g

M400AQUI_06092021_LRG098ch-H1 400 MHz
M400Q / Num.Inv. AF/004285
cdcl3 / Temp: 25C / N.Reg: XXXXXXXXXX
Usuari: san / Mostra: LRG098ch
Nom: LAURA RODRIGUEZ GONZALEZ
Data: 06/09/21 / Ope.: L.RODRIGUEZ

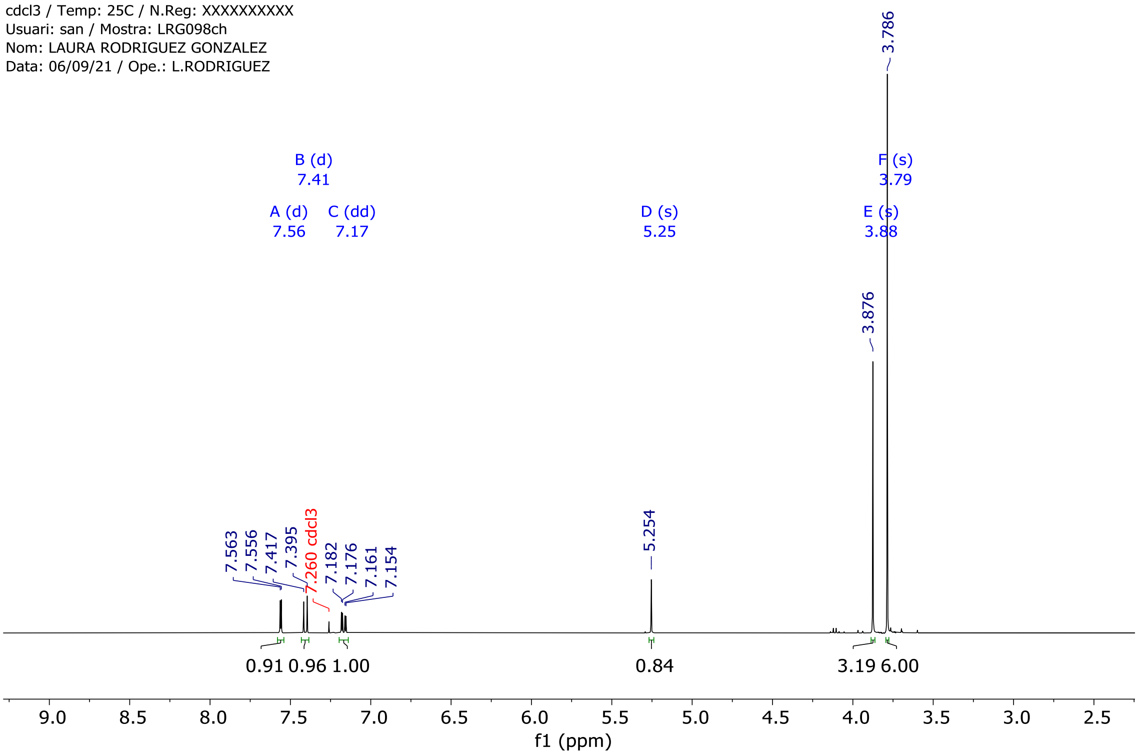

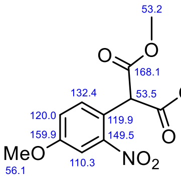

1g

M400AQUI_06092021_LRG098ch-C13{1H} 101 MHz
M400Q / Num.Inv. AF/004285
cdcl3 / Temp: 25C / N.Reg: XXXXXXXXXX
Usuari: san / Mostra: LRG098ch
Nom: LAURA RODRIGUEZ GONZALEZ
Data: 06/09/21 / Ope.: L.RODRIGUEZ

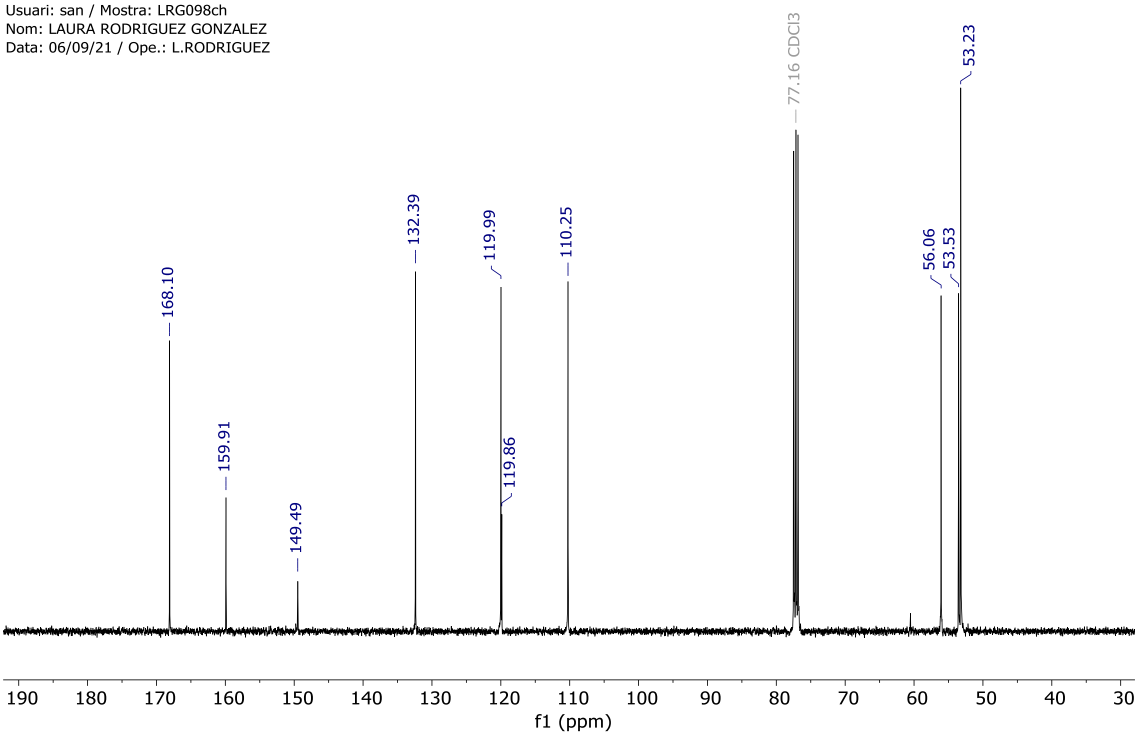

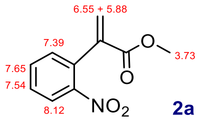

2a

M400APCB_01032021_LRG004CRUDE-H1 400 MHz
M400F / Num.Inv. 1009191
cdcl3 / Temp: 25C / N.Reg: XXXXXXXXXX
Usuari: san / Mostra: LRG004CRUDE
Nom: JORDI PUIG BOSCH
Data: 01/03/21 / Ope.: J.PUIG

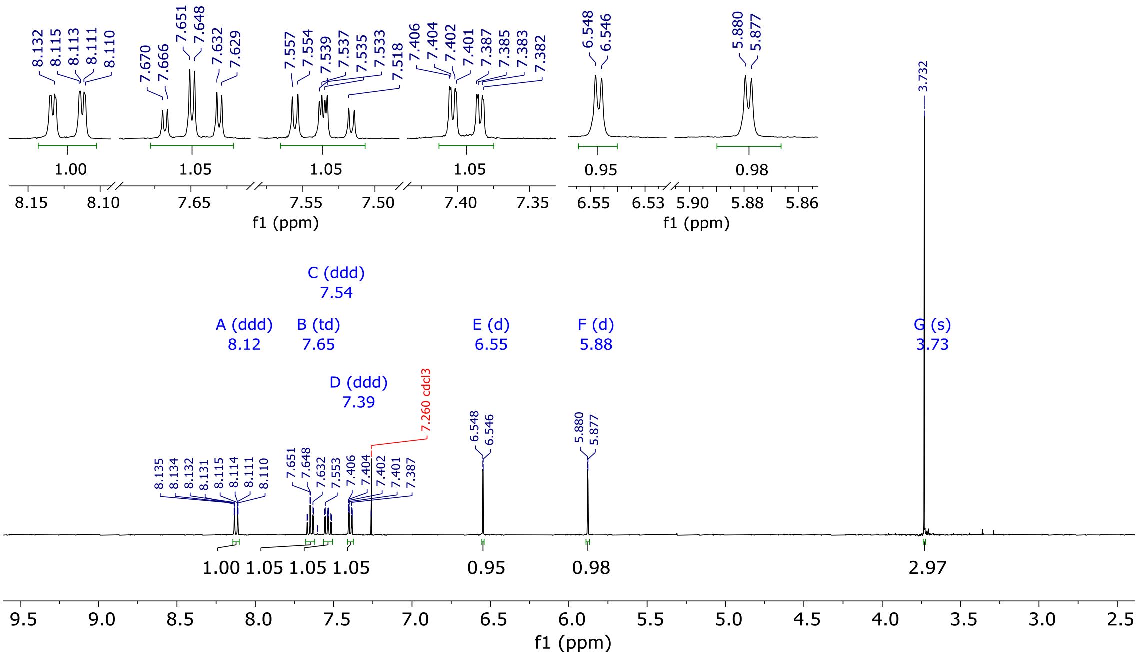

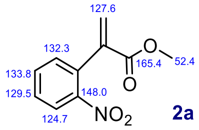

2a

VNMRS400A_14042021_LRG023CH-C13{1H} 101 MHz
VNMRS400F / Num.Inv. 205984
cdcl3 / Temp: 25C / N.Reg: XXXXXXXXXX
Usuari: san / Mostra: LRG023CH
Nom: LAURA RODRIGUEZ GONZALEZ
Data: 14/04/21 / Ope.: L.RODRIGUEZ

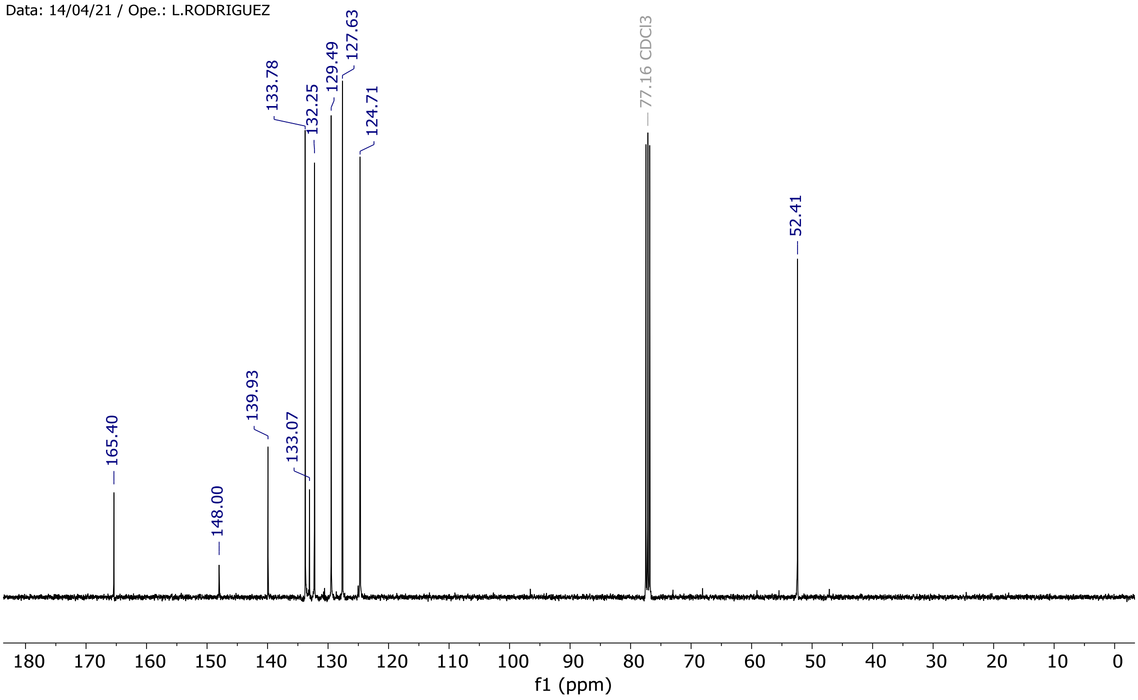

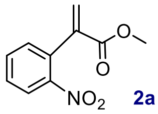
NO2

2a

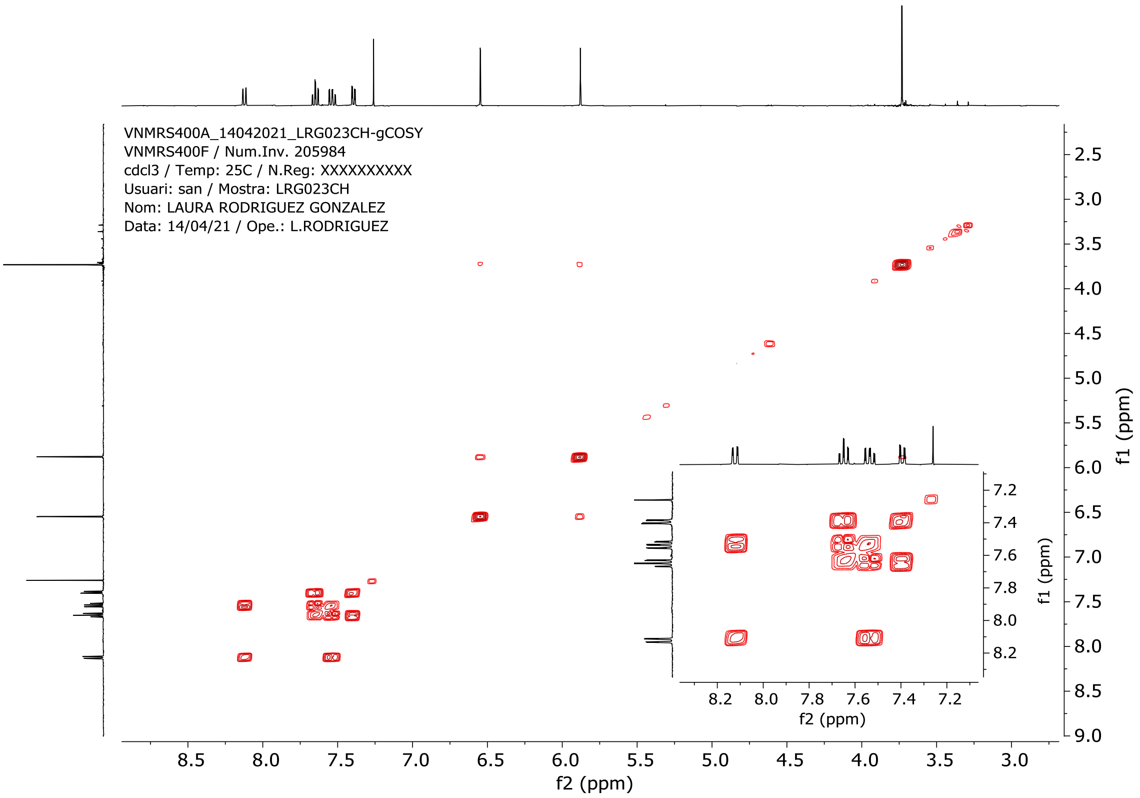
VNMRS400A_14042021_LRG023CH-gCOSY
VNMRS400F / Num.Inv. 205984
cdcl3 / Temp: 25C / N.Reg: XXXXXXXXXX
Usuari: san / Mostra: LRG023CH
Nom: LAURA RODRIGUEZ GONZALEZ
Data: 14/04/21 / Ope.: L.RODRIGUEZ
f2 (ppm)
f1 (ppm)

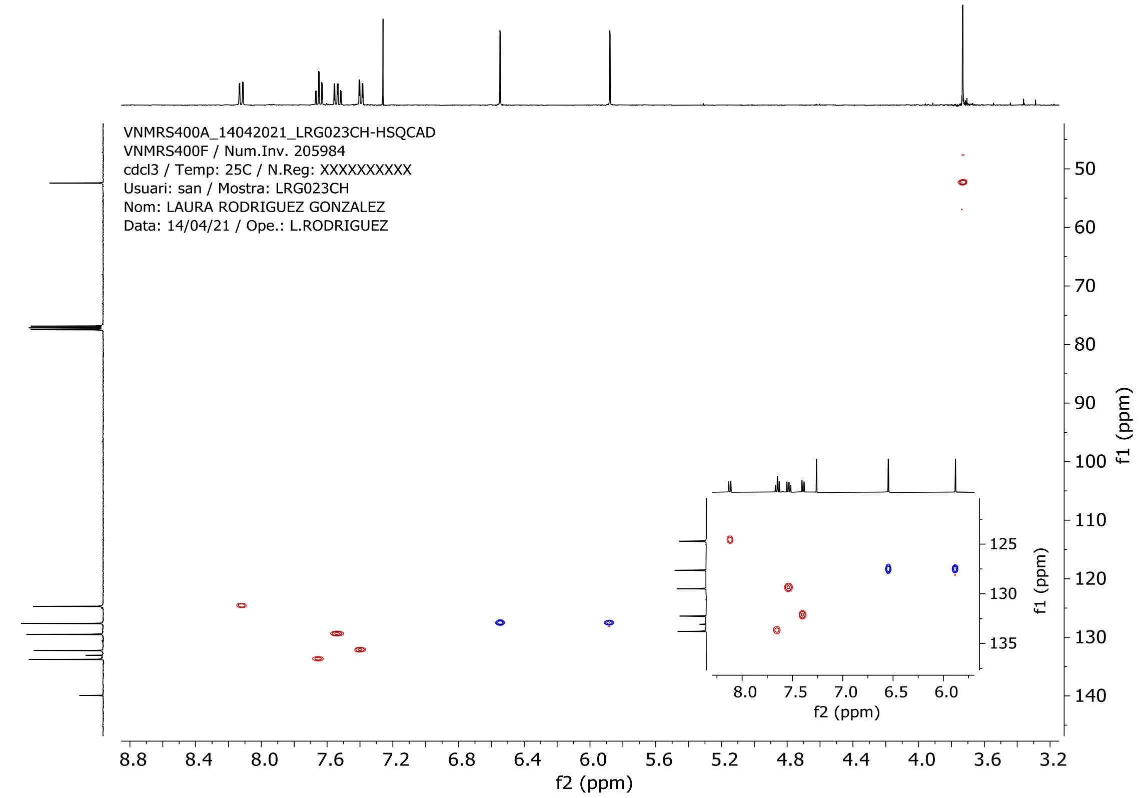
VNMRS400A_14042021_LRG023CH-HSQCAD
VNMRS400F / Num.Inv. 205984
cdcl3 / Temp: 25C / N.Reg: XXXXXXXXXX
Usuari: san / Mostra: LRG023CH
Nom: LAURA RODRIGUEZ GONZALEZ
Data: 14/04/21 / Ope.: L.RODRIGUEZ
f2 (ppm)
f1 (ppm)

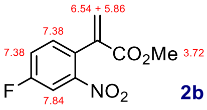

M400AQUI_02092021_LRG095ch-H1 400 MHz
M400Q / Num.Inv. AF/004285
cdcl3 / Temp: 25C / N.Reg: XXXXXXXXXX
Usuari: san / Mostra: LRG095ch
Nom: LAURA RODRIGUEZ GONZALEZ
Data: 02/09/21 / Ope.: L.RODRIGUEZ

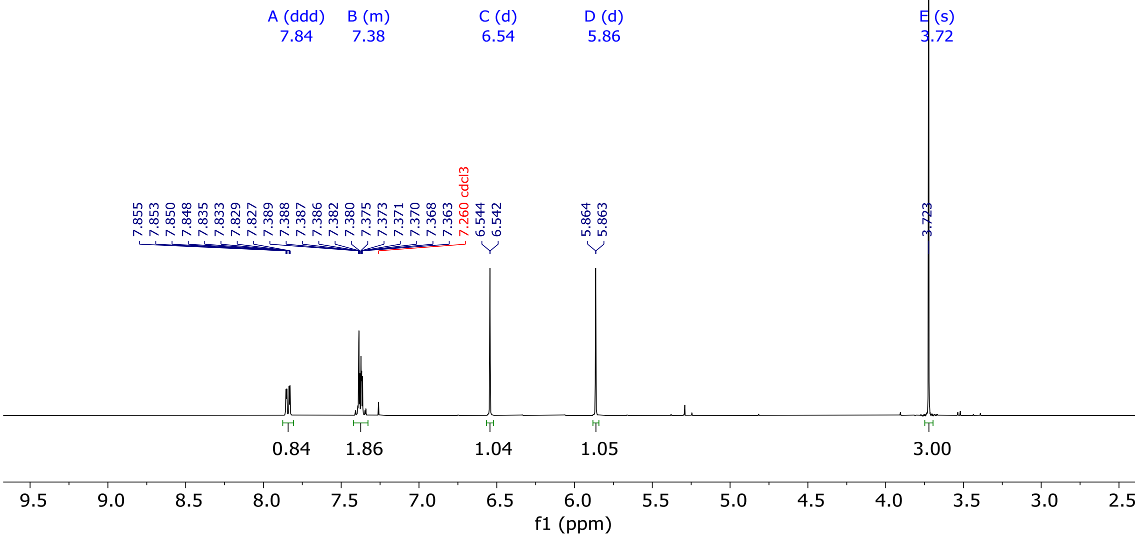

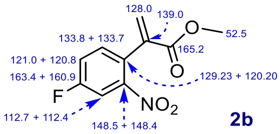

M400AQUI_02092021_LRG095ch-C13{1H} 101 MHz
M400Q / Num.Inv. AF/004285
cdcl3 / Temp: 25C / N.Reg: XXXXXXXXXX
Usuari: san / Mostra: LRG095ch
Nom: LAURA RODRIGUEZ GONZALEZ
Data: 02/09/21 / Ope.: L.RODRIGUEZ

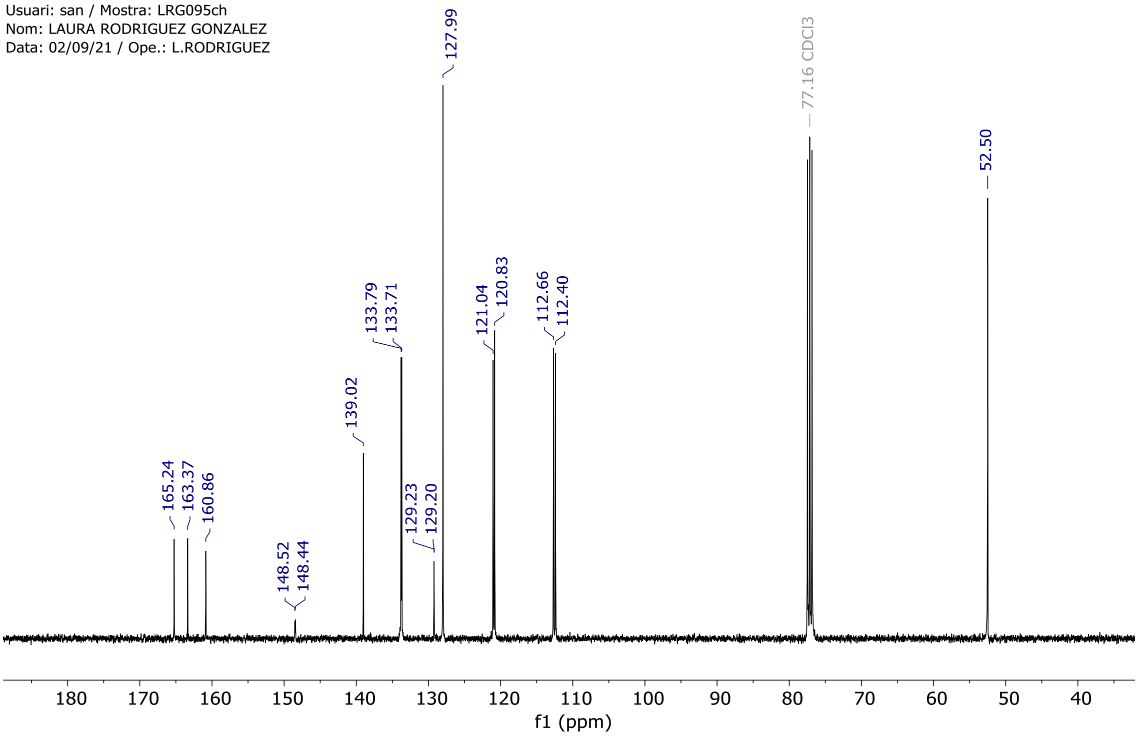

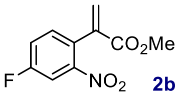
CO2Me
F
NO2

2b

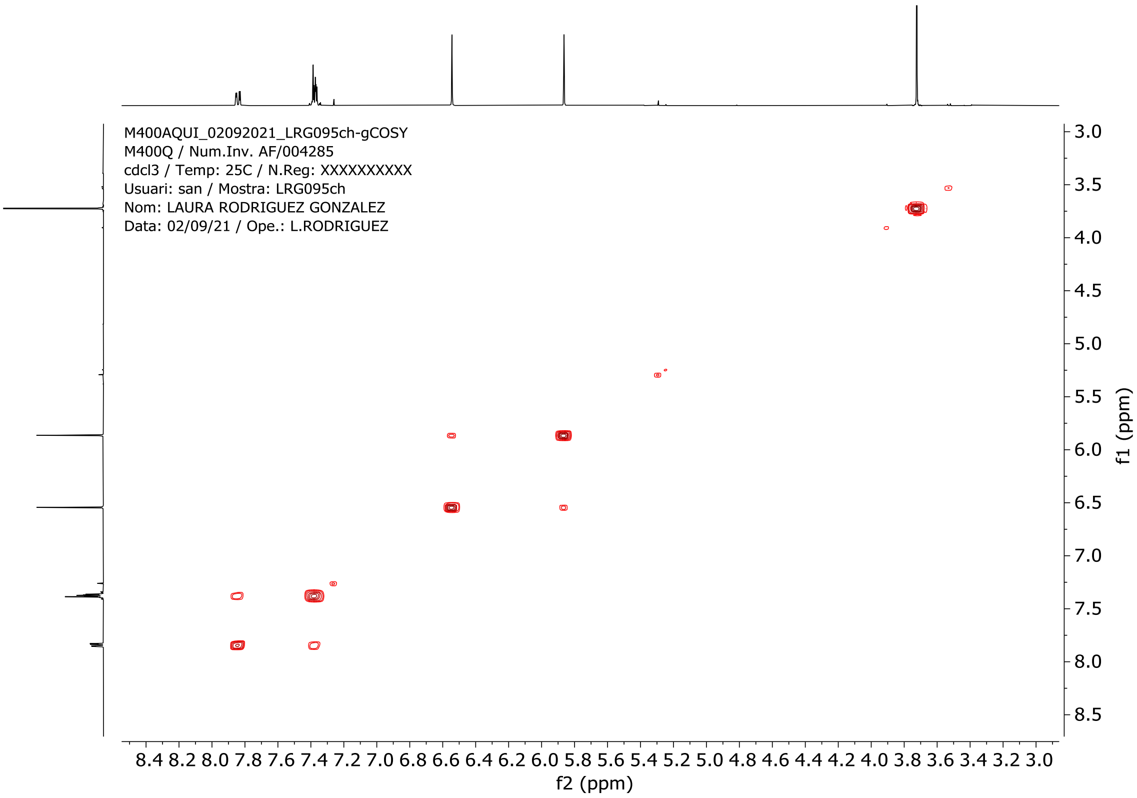
M400AQUI_02092021_LRG095ch-gCOSY
M400Q / Num.Inv. AF/004285
cdcl3 / Temp: 25C / N.Reg: XXXXXXXXXX
Usuari: san / Mostra: LRG095ch
Nom: LAURA RODRIGUEZ GONZALEZ
Data: 02/09/21 / Ope.: L.RODRIGUEZ
f1 (ppm)
f2 (ppm)

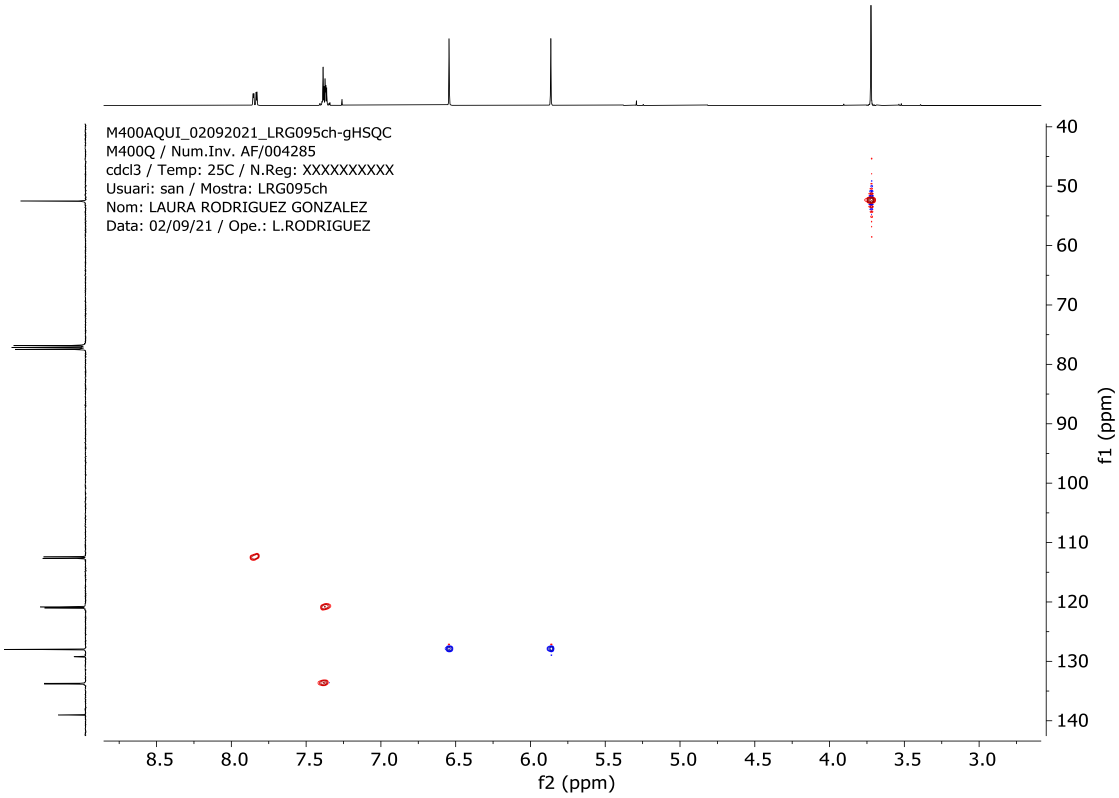
M400AQUI_02092021_LRG095ch-gHSQC
M400Q / Num.Inv. AF/004285
cdcl3 / Temp: 25C / N.Reg: XXXXXXXXXX
Usuari: san / Mostra: LRG095ch
Nom: LAURA RODRIGUEZ GONZALEZ
Data: 02/09/21 / Ope.: L.RODRIGUEZ
f1 (ppm)
f2 (ppm)

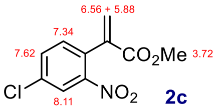

2c

M400AQUI_04092021_LRG097-H1 400 MHz
M400Q / Num.Inv. AF/004285
cdcl3 / Temp: 25C / N.Reg: XXXXXXXXXX
Usuari: san / Mostra: LRG097
Nom: LAURA RODRIGUEZ GONZALEZ
Data: 03/09/21 / Ope.: L.RODRIGUEZ

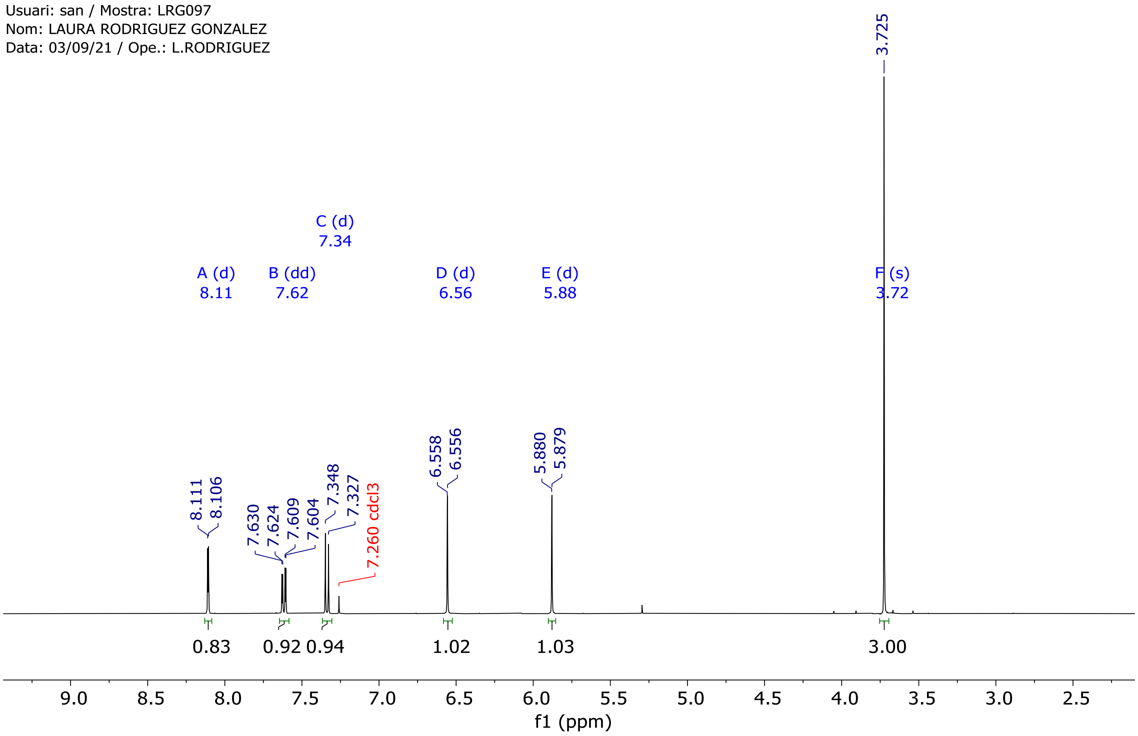

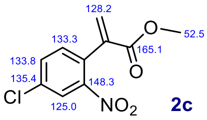

2c

M400AQUI_04092021_LRG097-C13{1H} 101 MHz
M400Q / Num.Inv. AF/004285
cdcl3 / Temp: 25C / N.Reg: XXXXXXXXXX
Usuari: san / Mostra: LRG097
Nom: LAURA RODRIGUEZ GONZALEZ
Data: 03/09/21 / Ope.: L.RODRIGUEZ

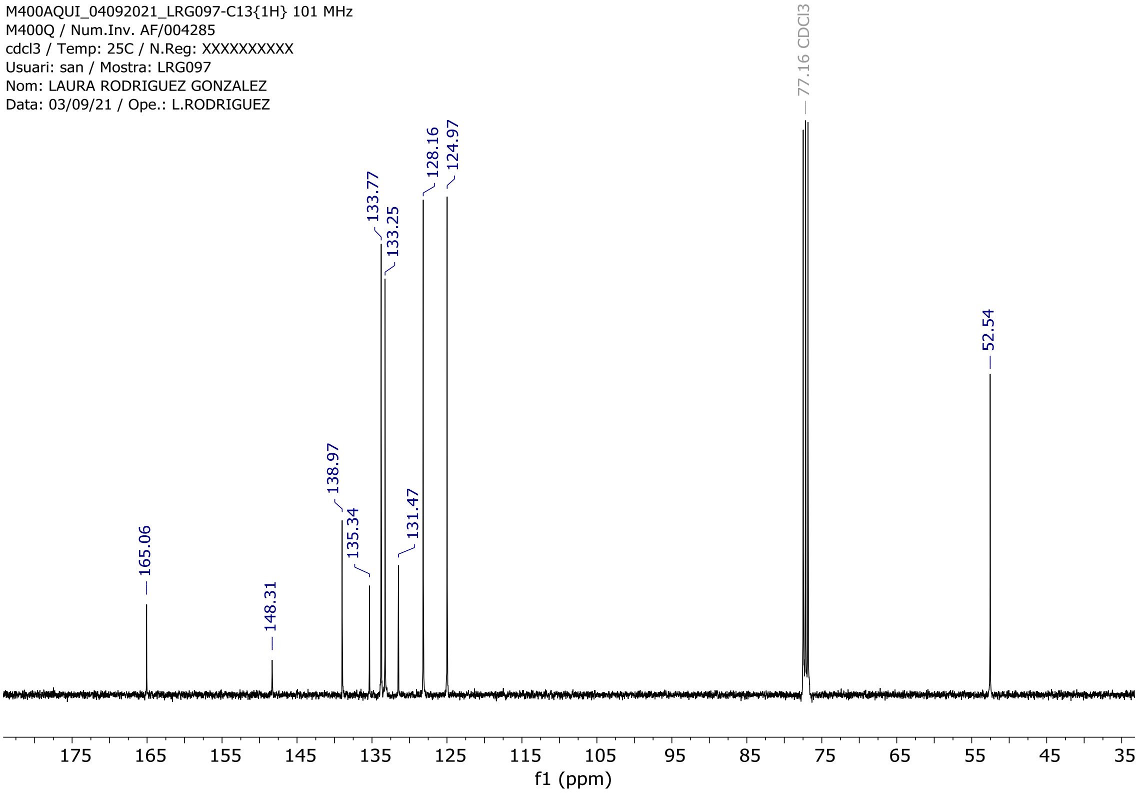

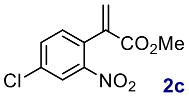
CO2Me
Cl
NO2

2c

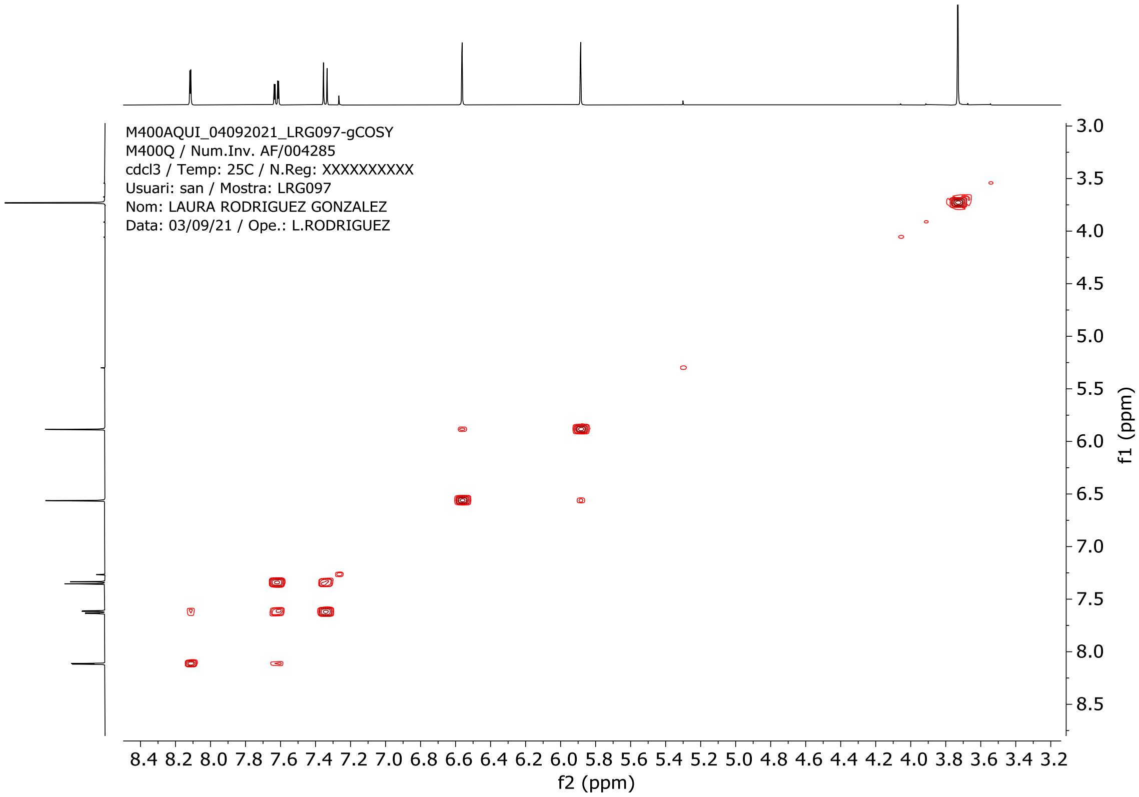
M400AQUI_04092021_LRG097-gCOSY
M400Q / Num.Inv. AF/004285
cdcl3 / Temp: 25C / N.Reg: XXXXXXXXXX
Usuari: san / Mostra: LRG097
Nom: LAURA RODRIGUEZ GONZALEZ
Data: 03/09/21 / Ope.: L.RODRIGUEZ
f1 (ppm)
f2 (ppm)
8.4 8.2 8.0 7.8 7.6 7.4 7.2 7.0 6.8 6.6 6.4 6.2 6.0 5.8 5.6 5.4 5.2 5.0 4.8 4.6 4.4 4.2 4.0 3.8 3.6 3.4 3.2
3.0 3.5 4.0 4.5 5.0 5.5 6.0 6.5 7.0 7.5 8.0 8.5

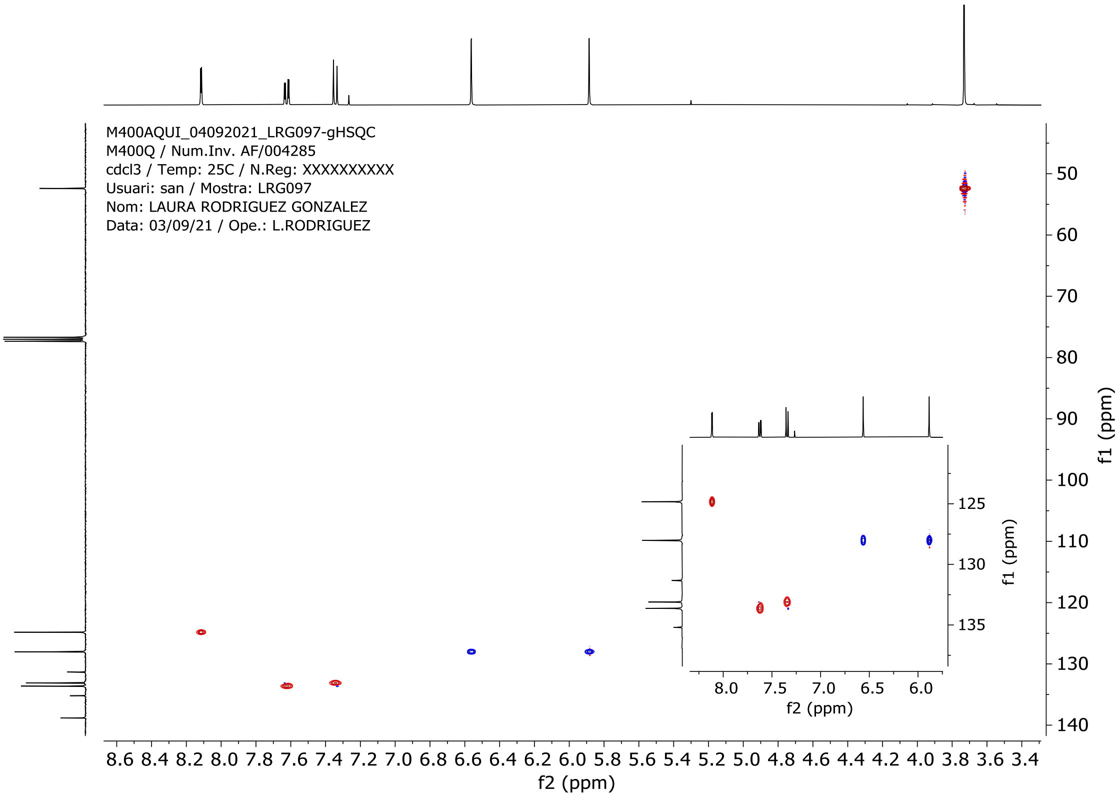
M400AQUI_04092021_LRG097-gHSQC
M400Q / Num.Inv. AF/004285
cdcl3 / Temp: 25C / N.Reg: XXXXXXXXXX
Usuari: san / Mostra: LRG097
Nom: LAURA RODRIGUEZ GONZALEZ
Data: 03/09/21 / Ope.: L.RODRIGUEZ
f1 (ppm)
f2 (ppm)
8.6 8.4 8.2 8.0 7.8 7.6 7.4 7.2 7.0 6.8 6.6 6.4 6.2 6.0 5.8 5.6 5.4 5.2 5.0 4.8 4.6 4.4 4.2 4.0 3.8 3.6 3.4
50 60 70 80 90 100 110 120 130 140
8.0 7.5 7.0 6.5 6.0
125 130 135

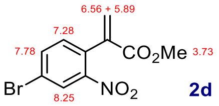

2d

M400AFAR_13072021_LRG086-H1 400 MHz
M400F / Num.Inv. 1009191
cdcl3 / Temp: 25C / N.Reg: XXXXXXXXXX
Usuari: san / Mostra: LRG086
Nom: LAURA RODRIGUEZ GONZALEZ
Data: 13/07/21 / Ope.: L.RODRIGUEZ

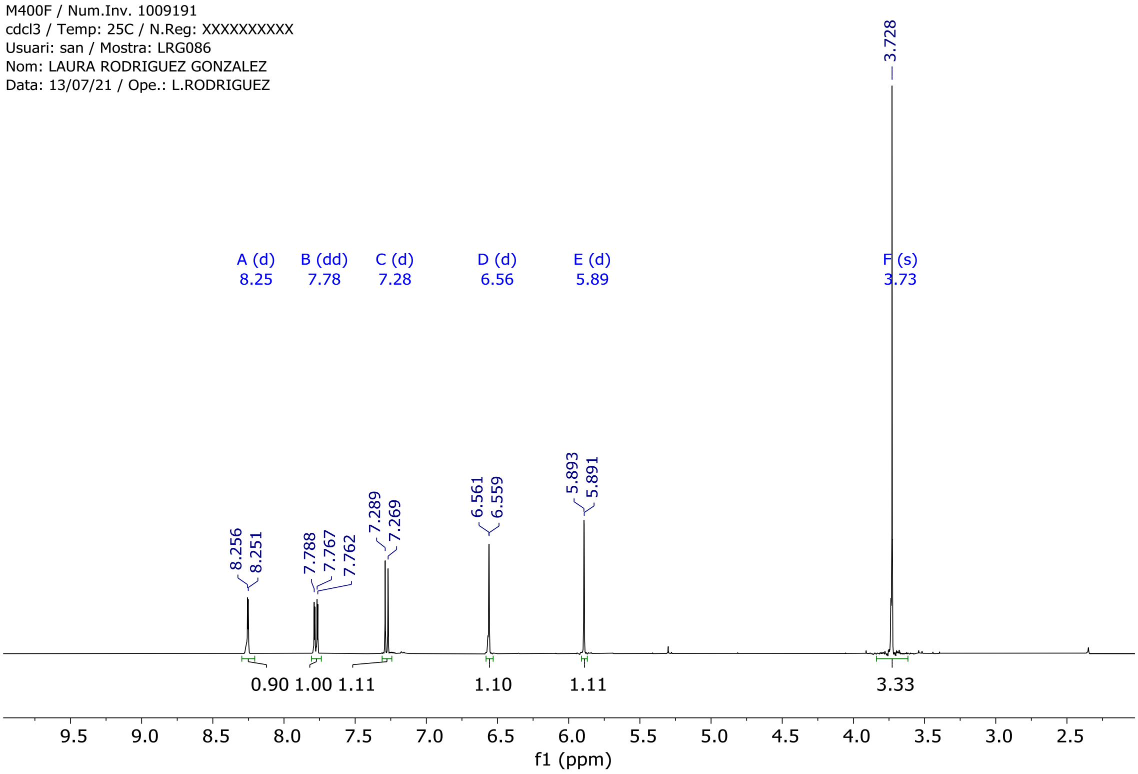

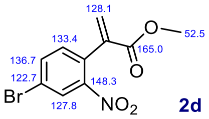

2d

M400AQUI_13072021_LRG086CHARAC-C13{1H} 101 MHz
M400Q / Num.Inv. AF/004285
cdcl3 / Temp: 25C / N.Reg: XXXXXXXXXX
Usuari: san / Mostra: LRG086CHARAC
Nom: LAURA RODRIGUEZ GONZALEZ
Data: 13/07/21 / Ope.: L.RODRIGUEZ

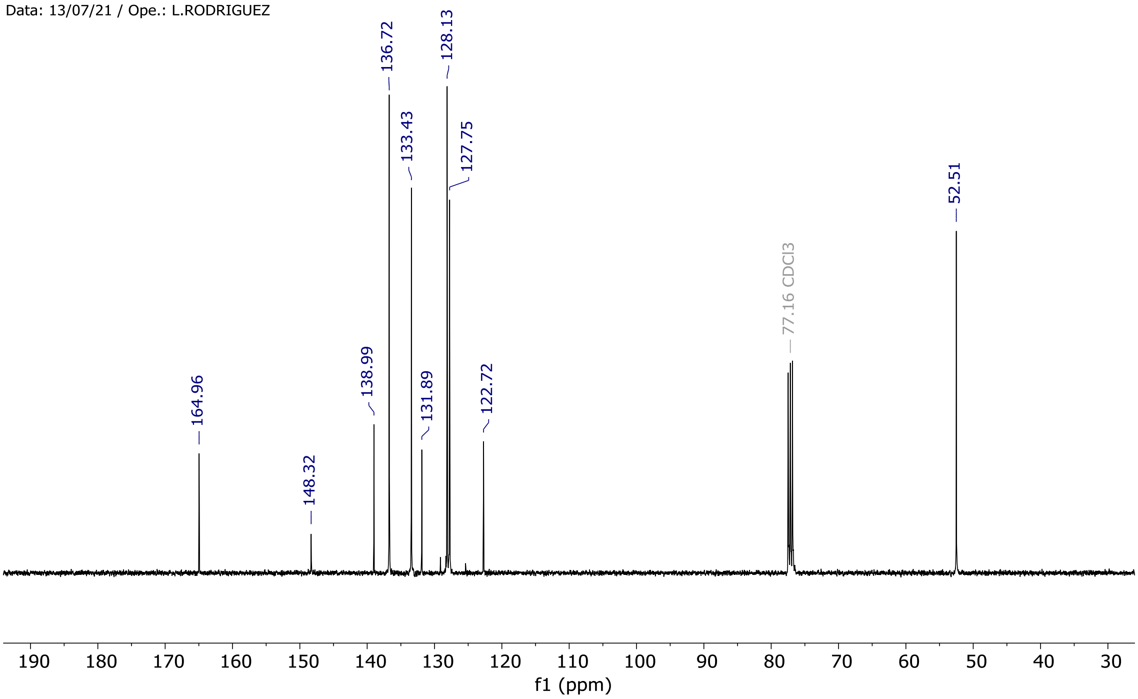

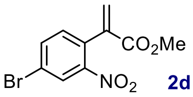
CO2Me
Br
NO2

2d

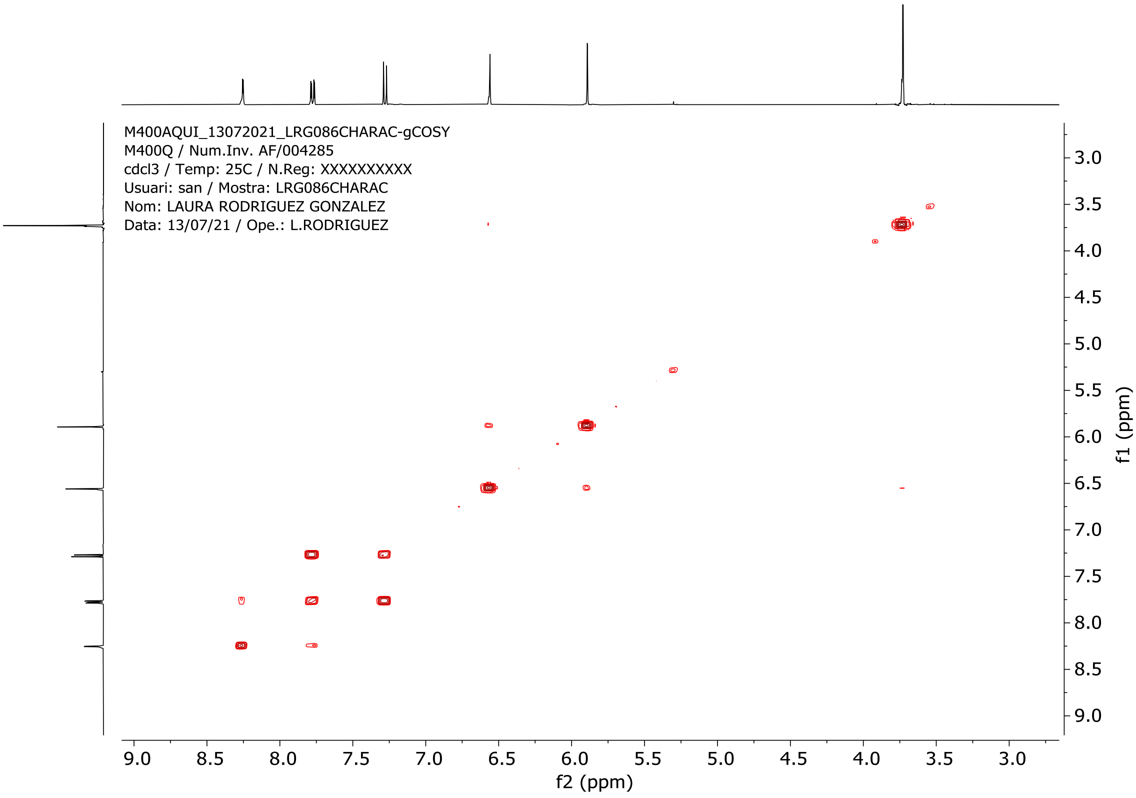
M400AQUI_13072021_LRG086CHARAC-gCOSY
M400Q / Num.Inv. AF/004285
cdcl3 / Temp: 25C / N.Reg: XXXXXXXXXX
Usuari: san / Mostra: LRG086CHARAC
Nom: LAURA RODRIGUEZ GONZALEZ
Data: 13/07/21 / Ope.: L.RODRIGUEZ
f1 (ppm)
f2 (ppm)

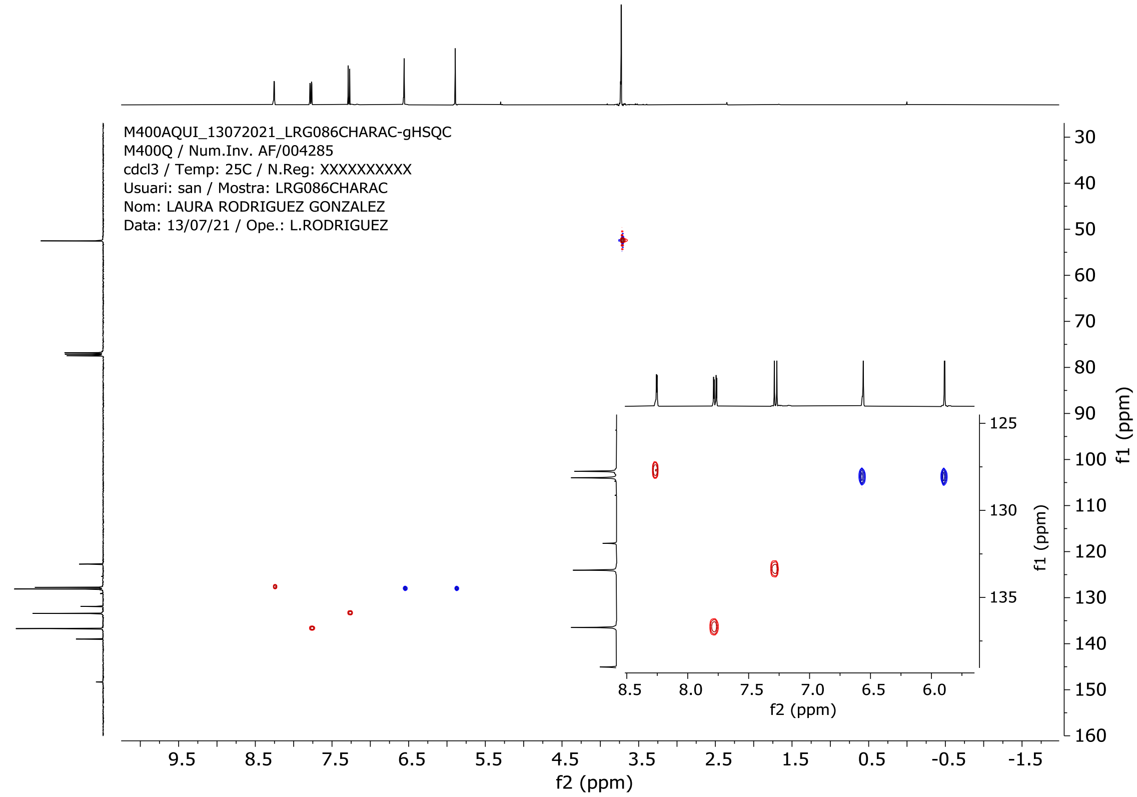
M400AQUI_13072021_LRG086CHARAC-gHSQC
M400Q / Num.Inv. AF/004285
cdcl3 / Temp: 25C / N.Reg: XXXXXXXXXX
Usuari: san / Mostra: LRG086CHARAC
Nom: LAURA RODRIGUEZ GONZALEZ
Data: 13/07/21 / Ope.: L.RODRIGUEZ
f1 (ppm)
f2 (ppm)

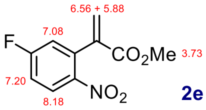

2e

M400AQUI_09072021_LRG083CHARAC-H1 400 MHz
M400Q / Num.Inv. AF/004285
cdcl3 / Temp: 25C / N.Reg: XXXXXXXXXX
Usuari: san / Mostra: LRG083CHARAC
Nom: LAURA RODRIGUEZ GONZALEZ
Data: 09/07/21 / Ope.: L.RODRIGUEZ

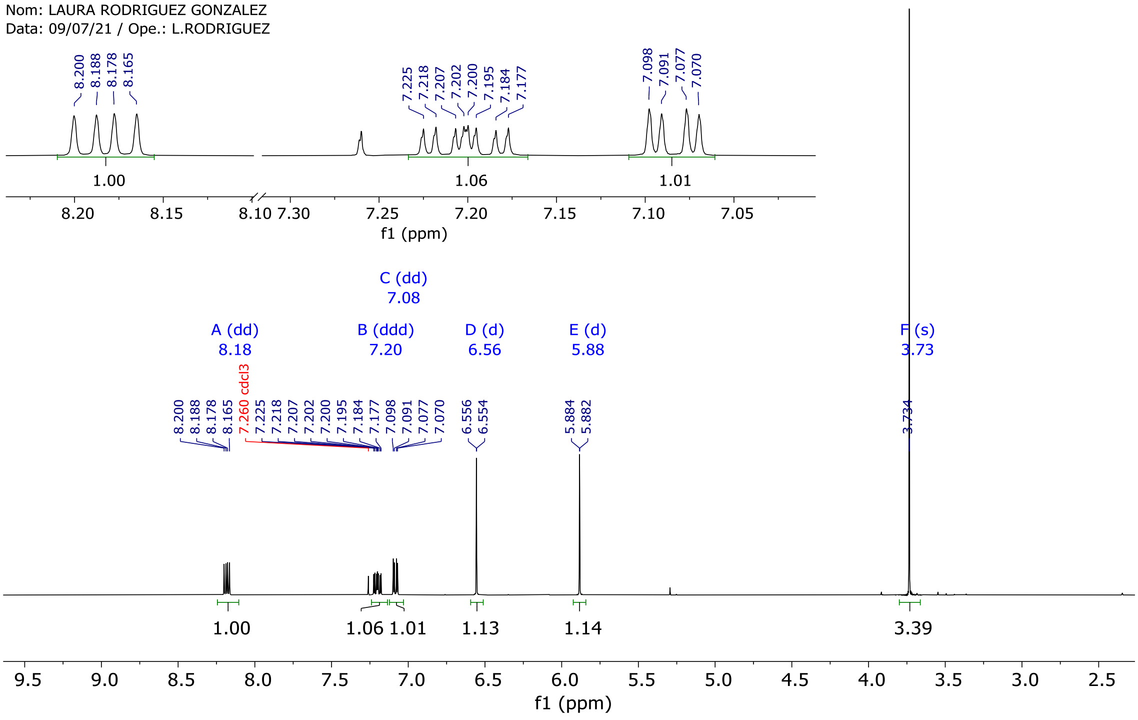

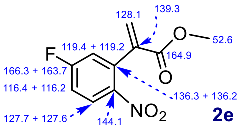

2e

M400AQUI_09072021_LRG083CHARAC-C13{1H} 101 MHz
M400Q / Num.Inv. AF/004285
cdcl3 / Temp: 25C / N.Reg: XXXXXXXXXX
Usuari: san / Mostra: LRG083CHARAC
Nom: LAURA RODRIGUEZ GONZALEZ
Data: 09/07/21 / Ope.: L.RODRIGUEZ

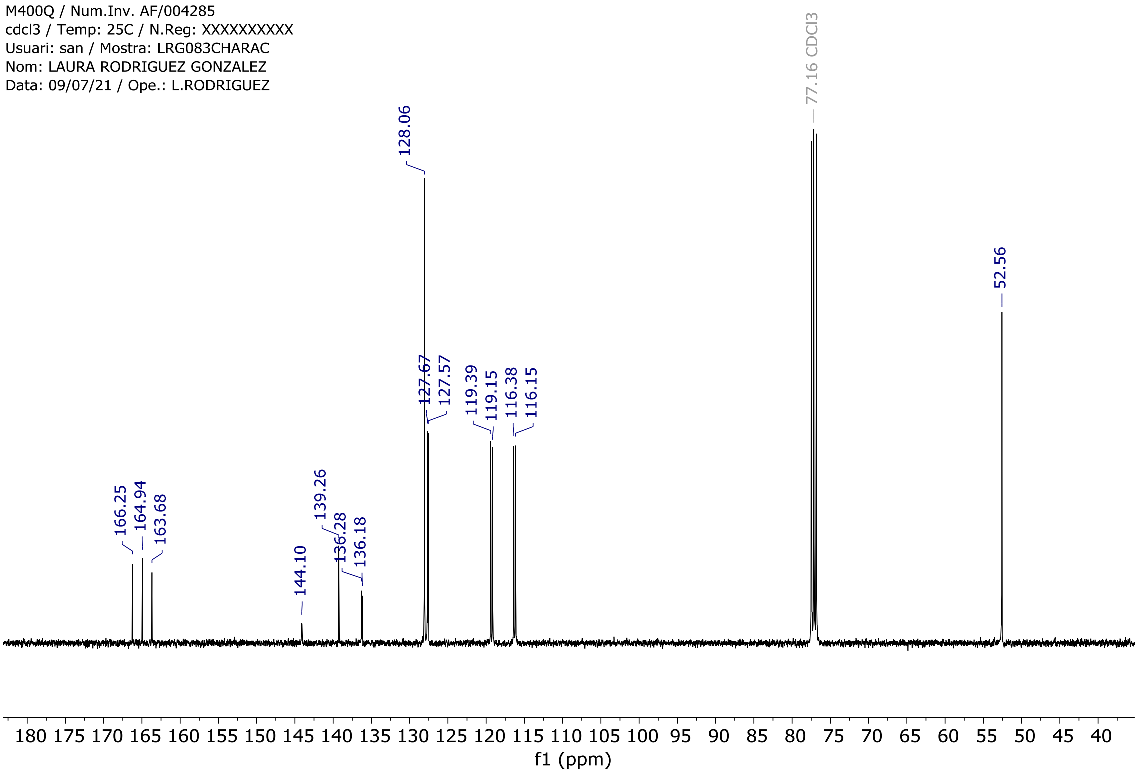

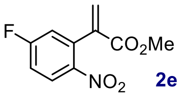

2e

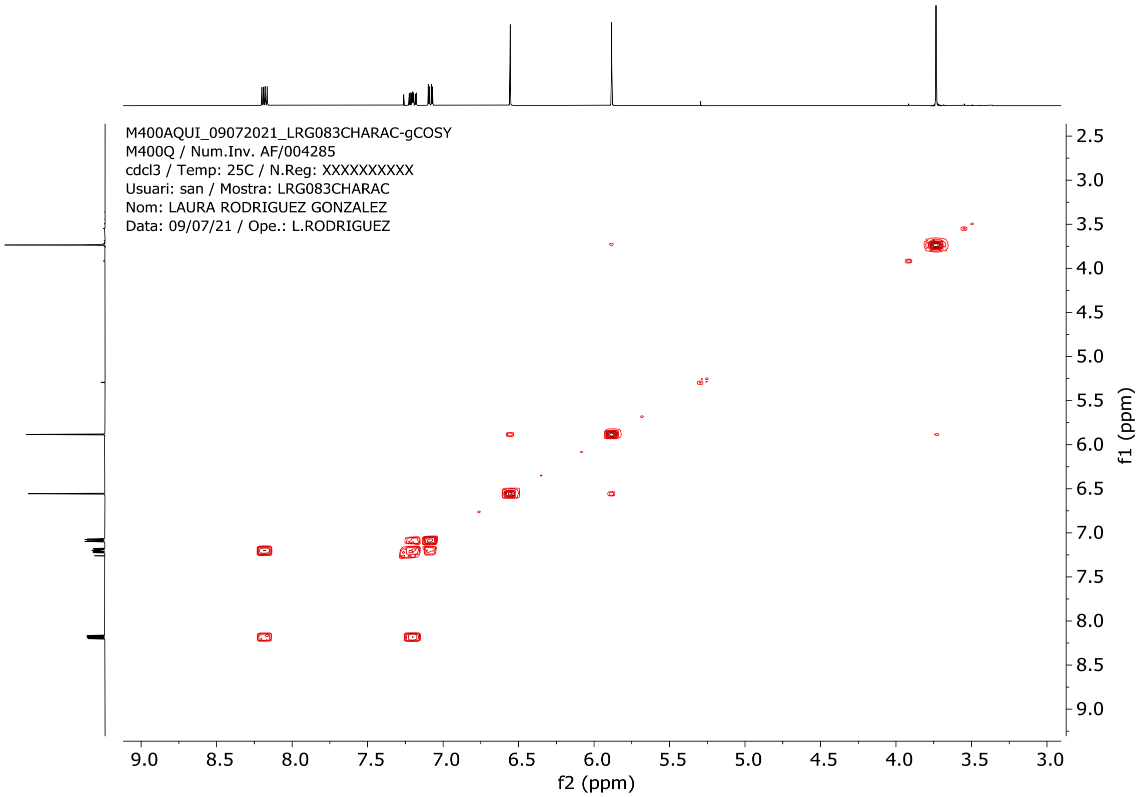
M400AQUI_09072021_LRG083CHARAC-gCOSY
M400Q / Num.Inv. AF/004285
cdcl3 / Temp: 25C / N.Reg: XXXXXXXXXX
Usuari: san / Mostra: LRG083CHARAC
Nom: LAURA RODRIGUEZ GONZALEZ
Data: 09/07/21 / Ope.: L.RODRIGUEZ
f2 (ppm)
f1 (ppm)

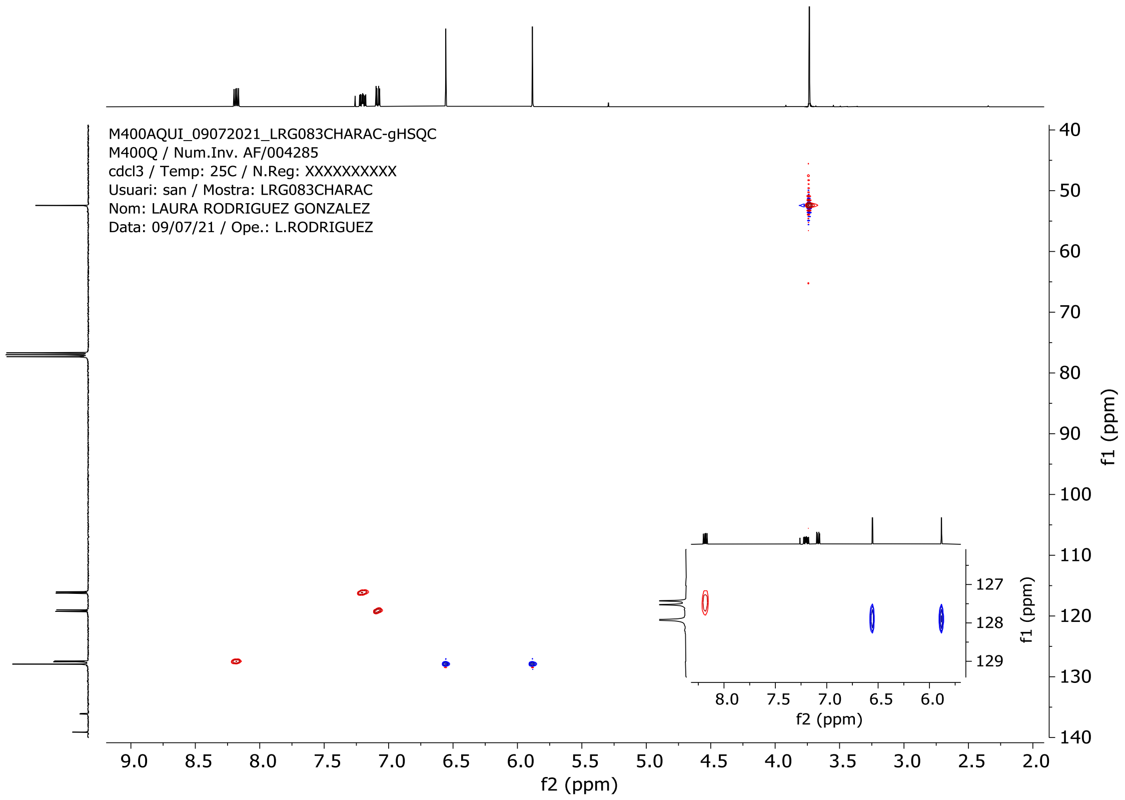
M400AQUI_09072021_LRG083CHARAC-gHSQC
M400Q / Num.Inv. AF/004285
cdcl3 / Temp: 25C / N.Reg: XXXXXXXXXX
Usuari: san / Mostra: LRG083CHARAC
Nom: LAURA RODRIGUEZ GONZALEZ
Data: 09/07/21 / Ope.: L.RODRIGUEZ
f2 (ppm)
f1 (ppm)

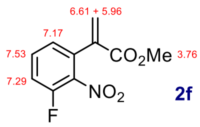

2f

M400AQUI_09092021_LRG103ch-H1 400 MHz
M400Q / Num.Inv. AF/004285
cdcl3 / Temp: 25C / N.Reg: XXXXXXXXXX
Usuari: san / Mostra: LRG103ch
Nom: LAURA RODRIGUEZ GONZALEZ
Data: 09/09/21 / Ope.: L.RODRIGUEZ

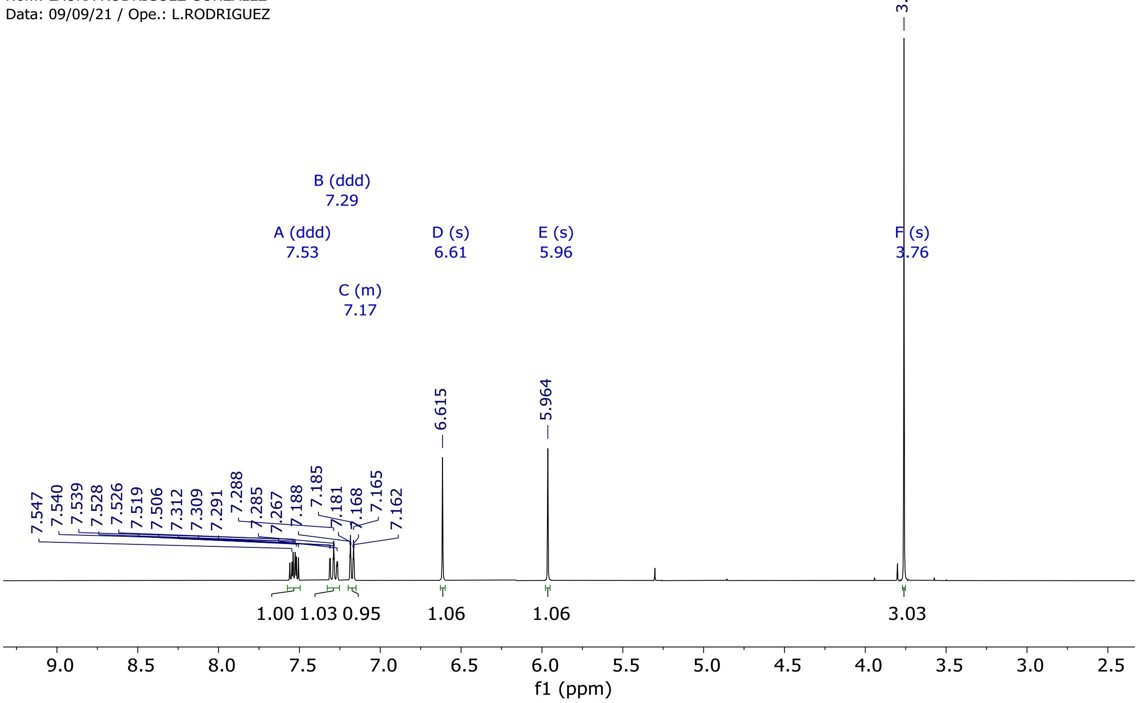

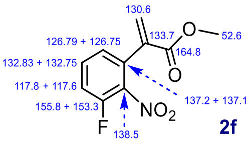

2f

M400AQUI_09092021_LRG103ch-C13{1H} 101 MHz
M400Q / Num.Inv. AF/004285
cdcl3 / Temp: 25C / N.Reg: XXXXXXXXXX
Usuari: san / Mostra: LRG103ch
Nom: LAURA RODRIGUEZ GONZALEZ
Data: 09/09/21 / Ope.: L.RODRIGUEZ

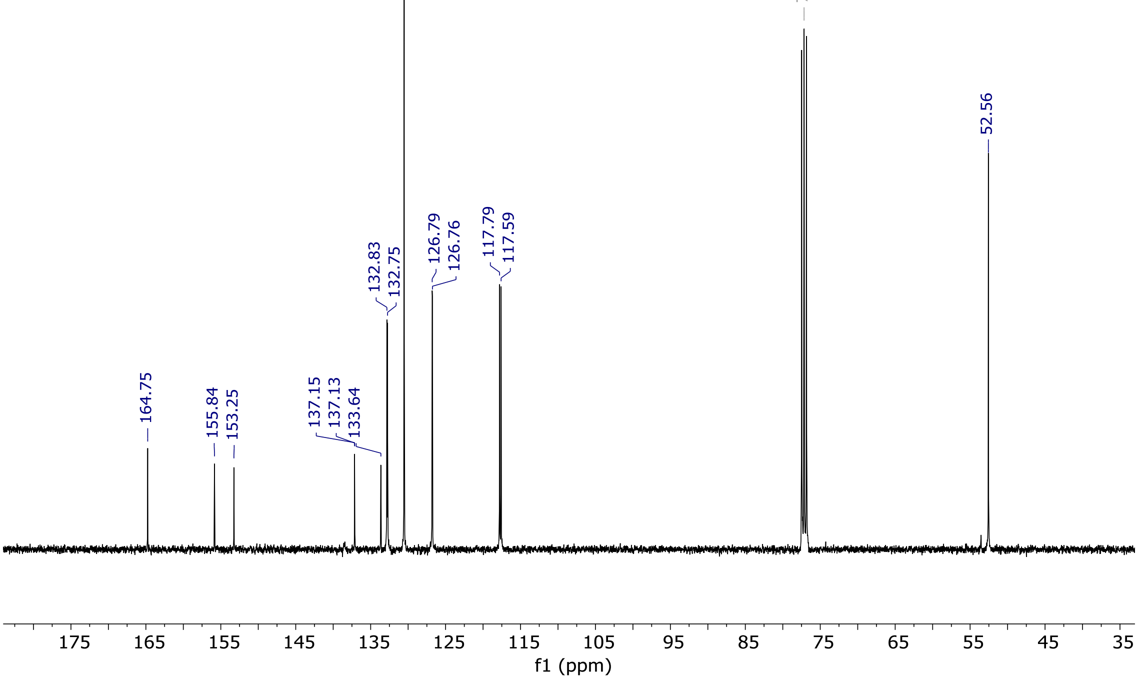

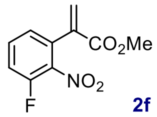
CO2Me
NO2
F

2f

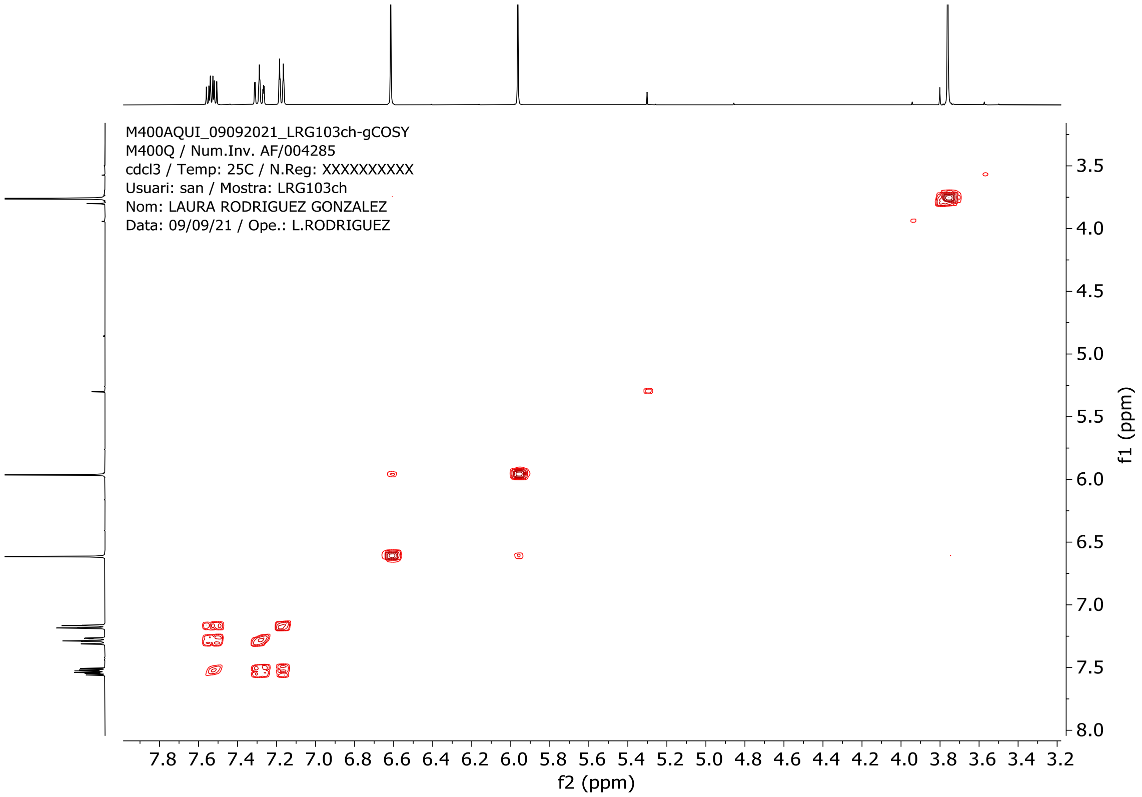
M400AQUI_09092021_LRG103ch-gCOSY
M400Q / Num.Inv. AF/004285
cdcl3 / Temp: 25C / N.Reg: XXXXXXXXXX
Usuari: san / Mostra: LRG103ch
Nom: LAURA RODRIGUEZ GONZALEZ
Data: 09/09/21 / Ope.: L.RODRIGUEZ
f1 (ppm)
f2 (ppm)

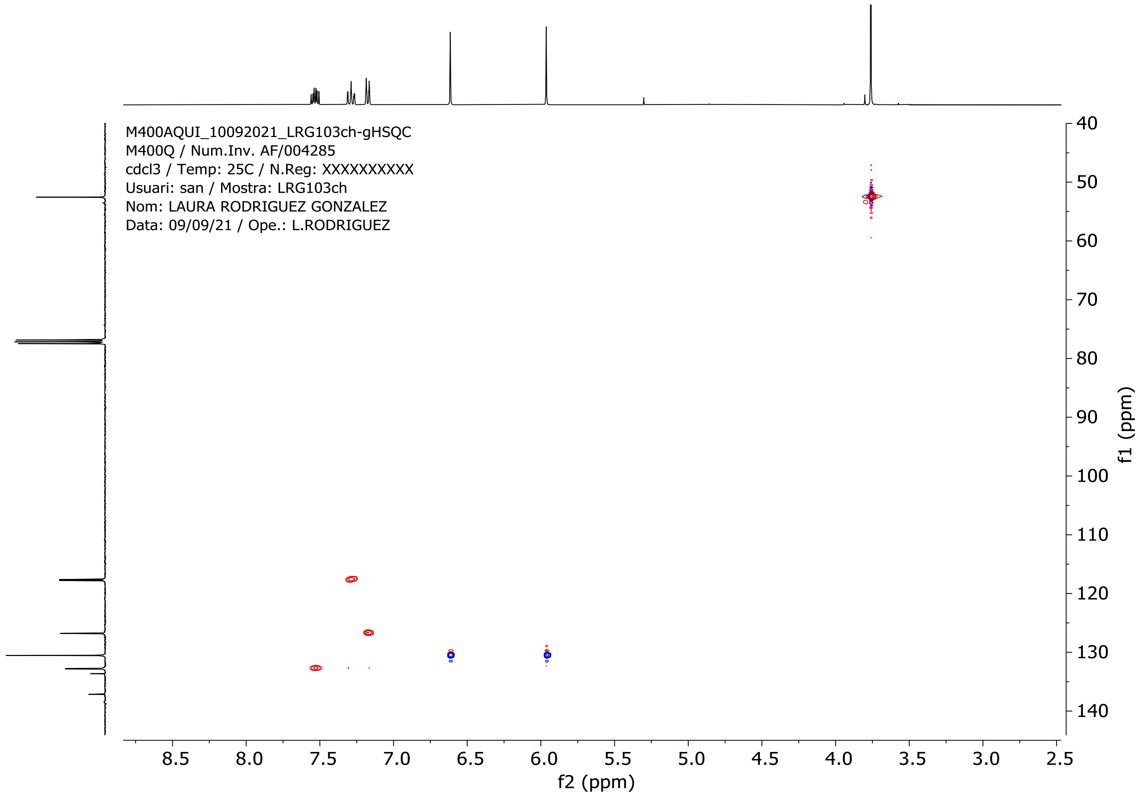
M400AQUI_10092021_LRG103ch-gHSQC
M400Q / Num.Inv. AF/004285
cdcl3 / Temp: 25C / N.Reg: XXXXXXXXXX
Usuari: san / Mostra: LRG103ch
Nom: LAURA RODRIGUEZ GONZALEZ
Data: 09/09/21 / Ope.: L.RODRIGUEZ
f1 (ppm)
f2 (ppm)

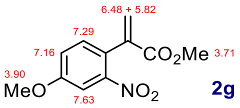

2g

M400AQUI_07092021_LRG099ch-H1 400 MHz
M400Q / Num.Inv. AF/004285
cdcl3 / Temp: 25C / N.Reg: XXXXXXXXXX
Usuari: san / Mostra: LRG099ch
Nom: LAURA RODRIGUEZ GONZALEZ
Data: 07/09/21 / Ope.: L.RODRIGUEZ

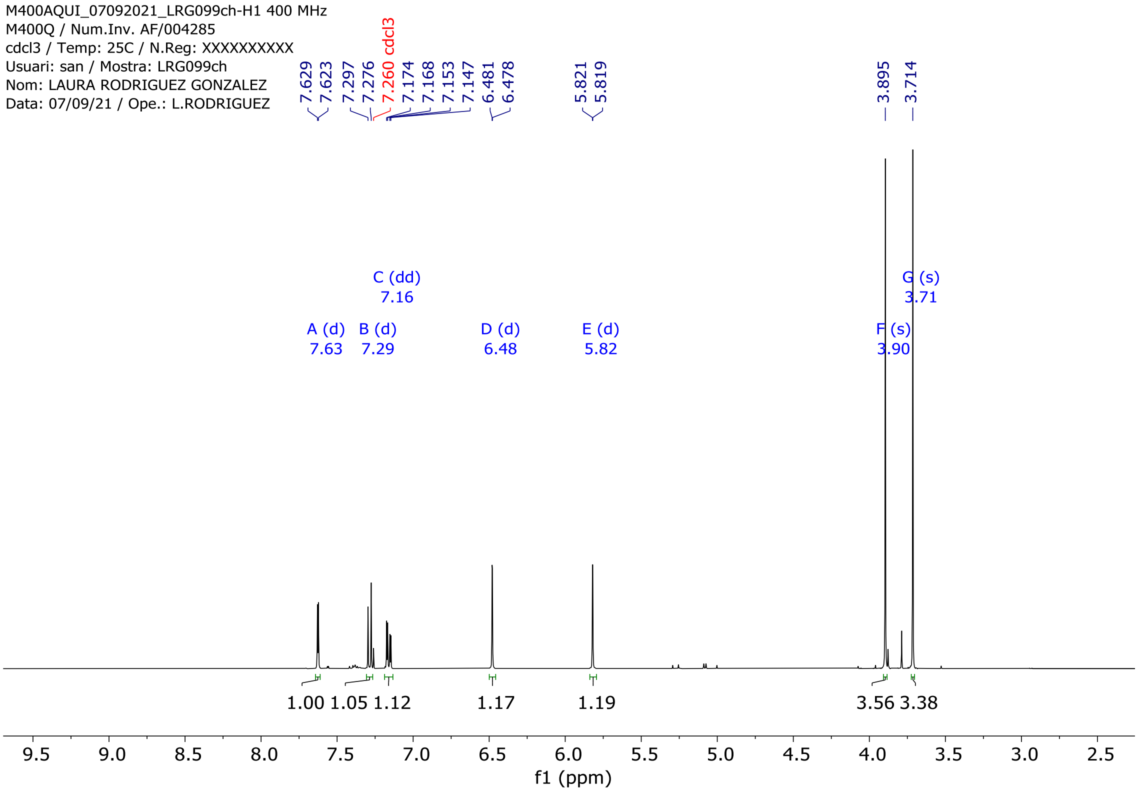

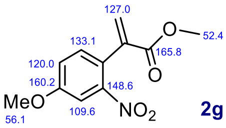

2g

M400AQUI_07092021_LRG099ch-C13{1H} 101 MHz
M400Q / Num.Inv. AF/004285
cdcl3 / Temp: 25C / N.Reg: XXXXXXXXXX
Usuari: san / Mostra: LRG099ch
Nom: LAURA RODRIGUEZ GONZALEZ
Data: 07/09/21 / Ope.: L.RODRIGUEZ

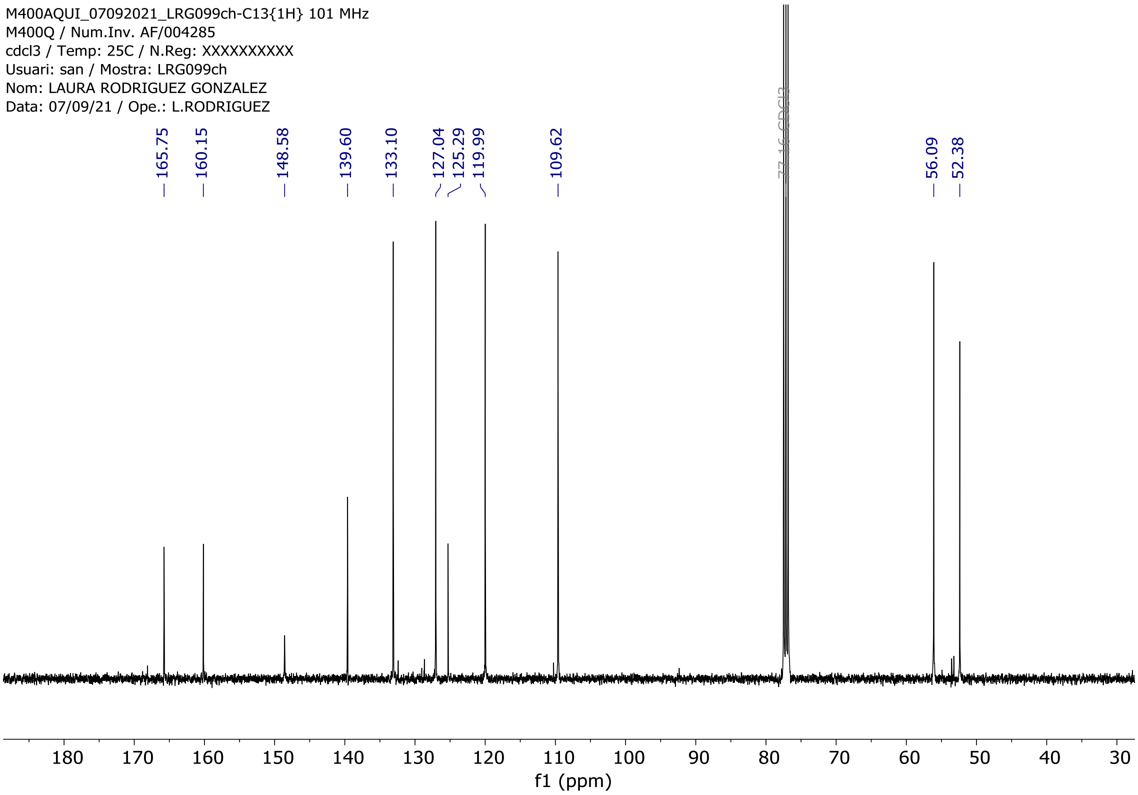

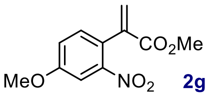
CO2Me
MeO
NO2

2g

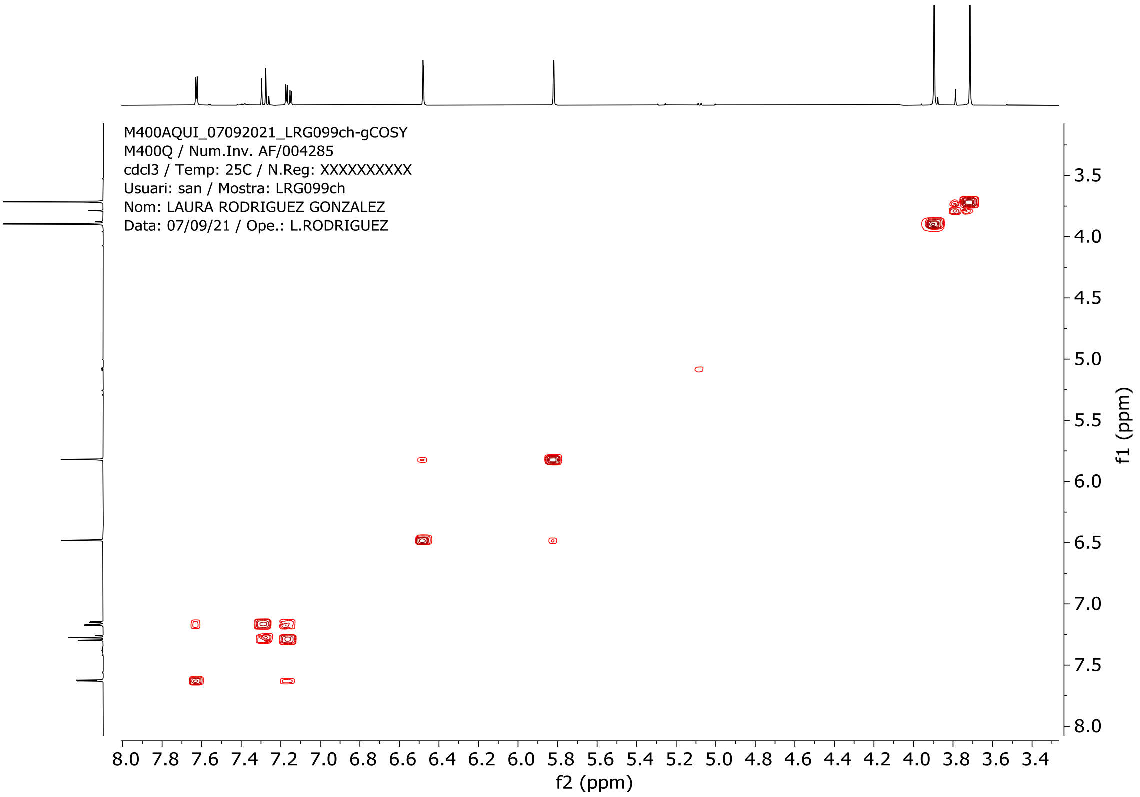
M400AQUI_07092021_LRG099ch-gCOSY
M400Q / Num.Inv. AF/004285
cdcl3 / Temp: 25C / N.Reg: XXXXXXXXXX
Usuari: san / Mostra: LRG099ch
Nom: LAURA RODRIGUEZ GONZALEZ
Data: 07/09/21 / Ope.: L.RODRIGUEZ
f2 (ppm)
f1 (ppm)

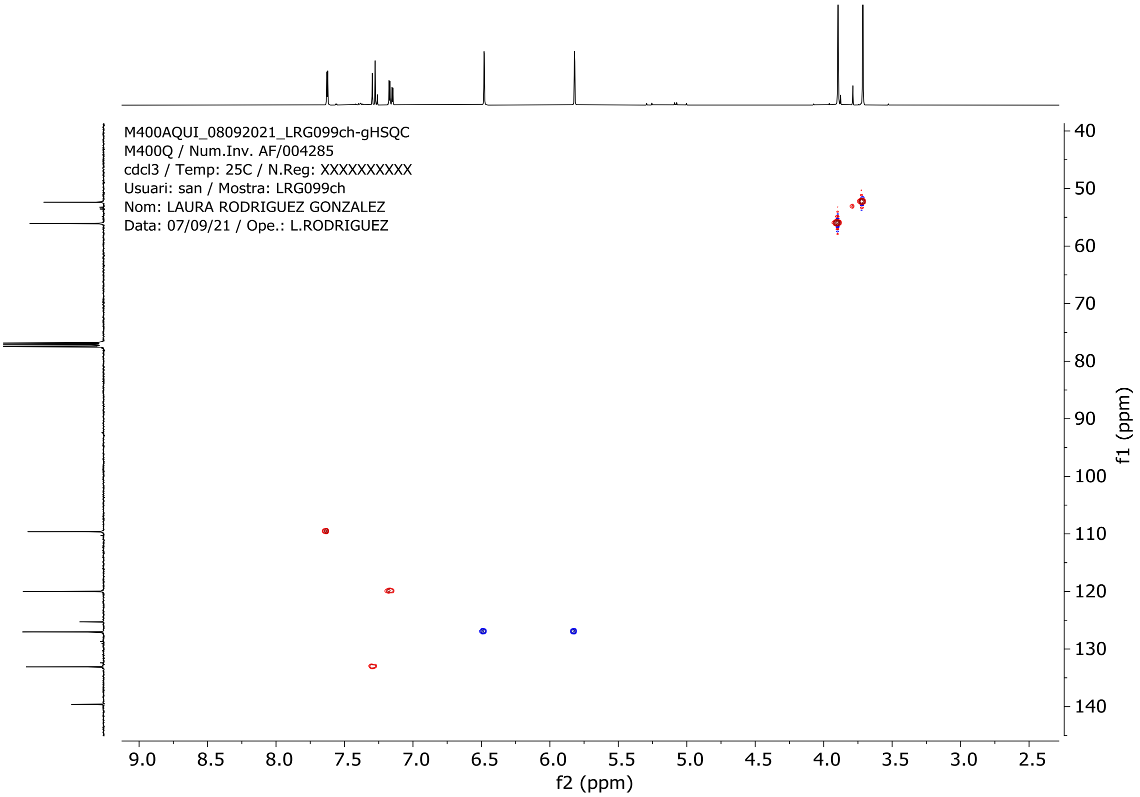
M400AQUI_08092021_LRG099ch-gHSQC
M400Q / Num.Inv. AF/004285
cdcl3 / Temp: 25C / N.Reg: XXXXXXXXXX
Usuari: san / Mostra: LRG099ch
Nom: LAURA RODRIGUEZ GONZALEZ
Data: 07/09/21 / Ope.: L.RODRIGUEZ
f2 (ppm)
f1 (ppm)

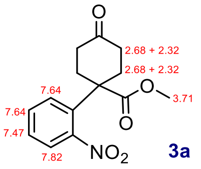

3a

VNMRS400A_07042021_LRG018CH-H1 400 MHz
VNMRS400F / Num.Inv. 205984
cdcl3 / Temp: 25C / N.Reg: XXXXXXXXXX
Usuari: san / Mostra: LRG018CH
Nom: LAURA RODRIGUEZ GONZALEZ
Data: 07/04/21 / Ope.: L.RODRIGUEZ

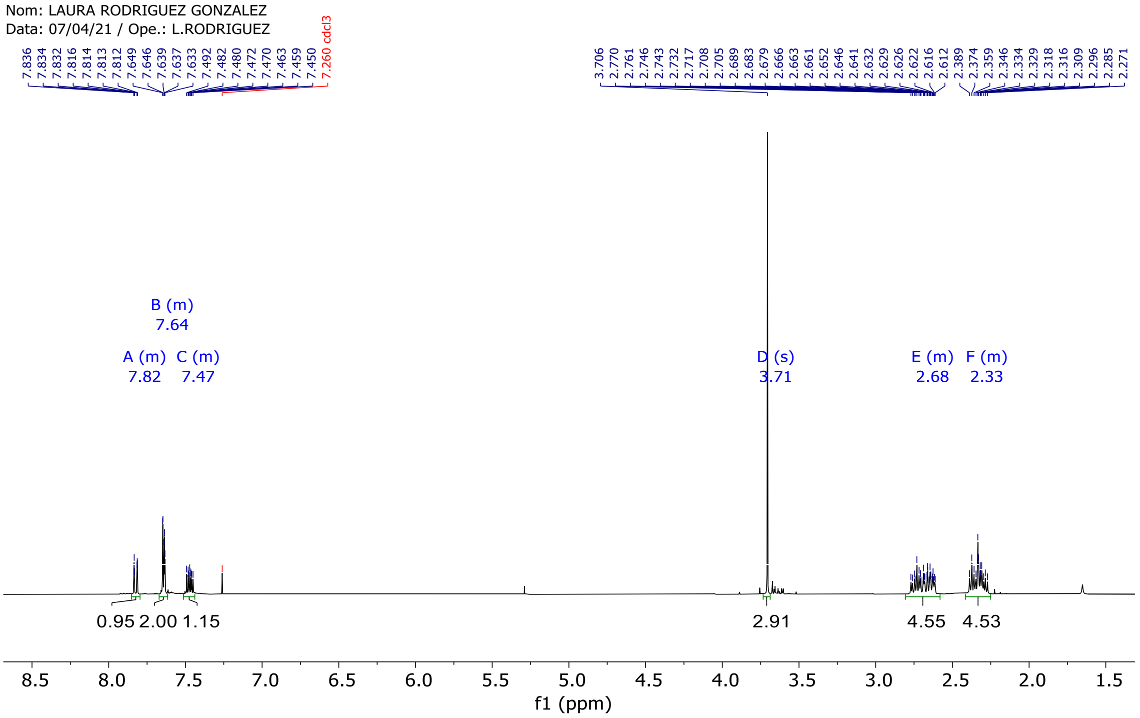

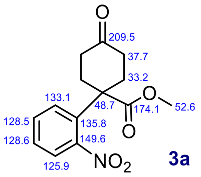

3a

VNMRS400A_07042021_LRG018CH-C13{1H} 101 MHz
VNMRS400F / Num.Inv. 205984
cdcl3 / Temp: 25C / N.Reg: XXXXXXXXXX
Usuari: san / Mostra: LRG018CH
Nom: LAURA RODRIGUEZ GONZALEZ
Data: 07/04/21 / Ope.: L.RODRIGUEZ

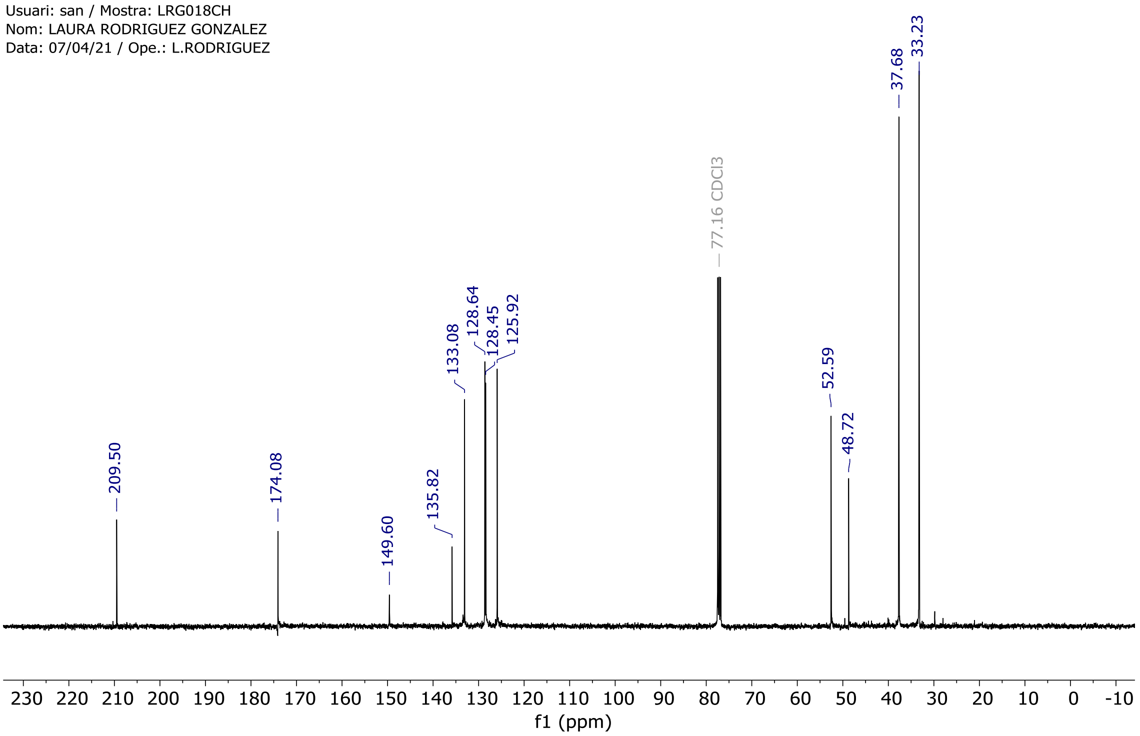

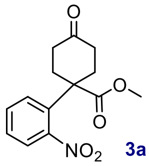

3a

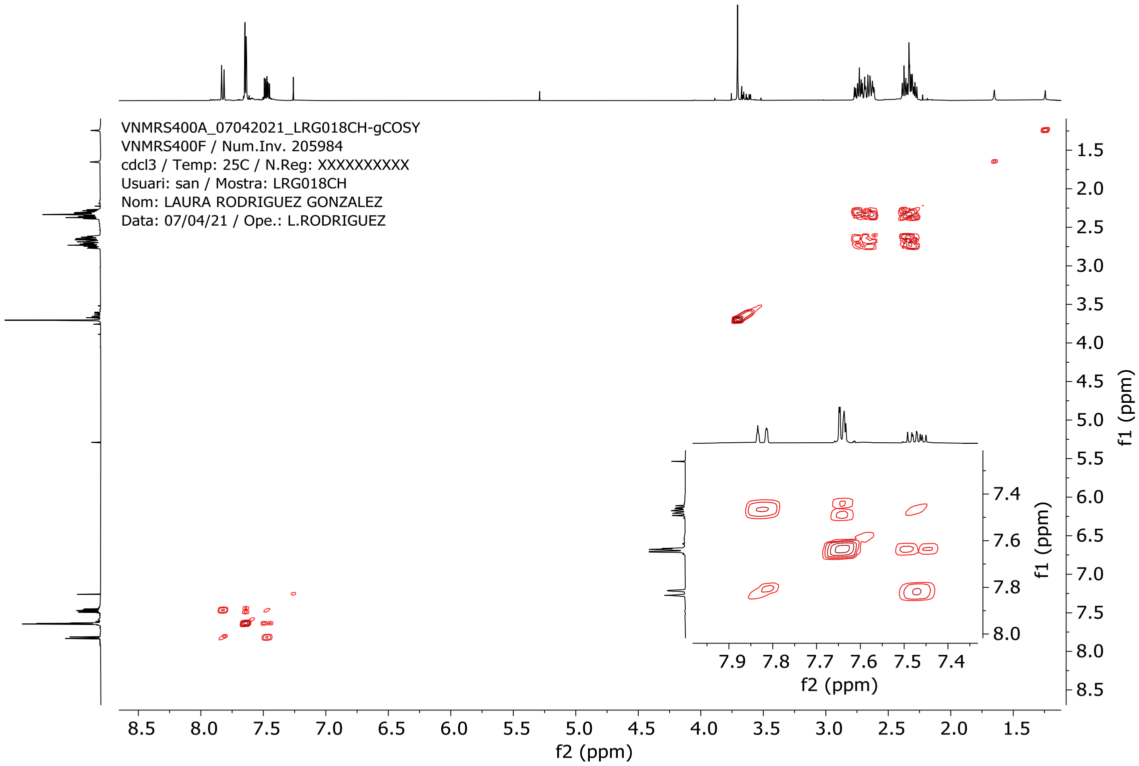

VNMRS400A_07042021_LRG018CH-gCOSY
VNMRS400F / Num.Inv. 205984
cdcl3 / Temp: 25C / N.Reg: XXXXXXXXXX
Usuari: san / Mostra: LRG018CH
Nom: LAURA RODRIGUEZ GONZALEZ
Data: 07/04/21 / Ope.: L.RODRIGUEZ
f1 (ppm)
f2 (ppm)

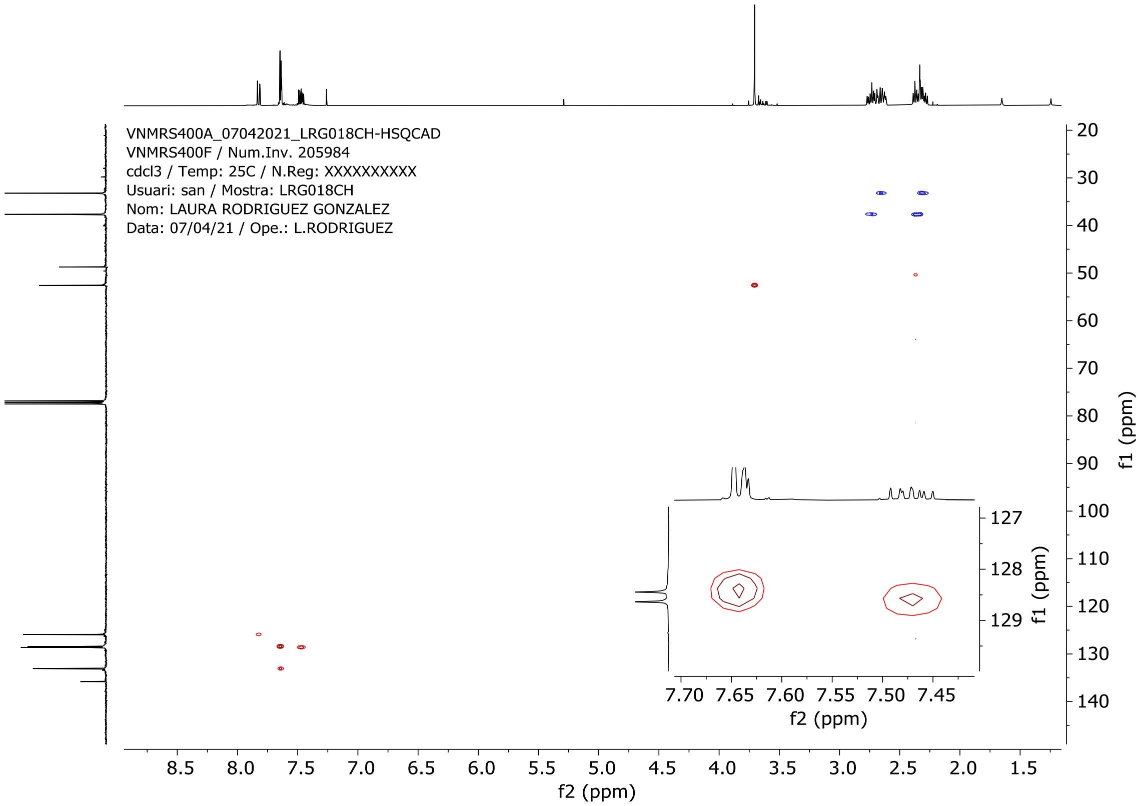

VNMRS400A_07042021_LRG018CH-HSQCAD
VNMRS400F / Num.Inv. 205984
cdcl3 / Temp: 25C / N.Reg: XXXXXXXXXX
Usuari: san / Mostra: LRG018CH
Nom: LAURA RODRIGUEZ GONZALEZ
Data: 07/04/21 / Ope.: L.RODRIGUEZ
f1 (ppm)
f2 (ppm)

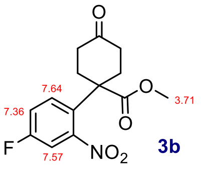

3b

M400AQUI_13092021_LRG102TEST-H1 400 MHz
M400Q / Num.Inv. AF/004285
cdcl3 / Temp: 25C / N.Reg: XXXXXXXXXX
Usuari: san / Mostra: LRG102TEST
Nom: LAURA RODRIGUEZ GONZALEZ
Data: 13/09/21 / Ope.: L.RODRIGUEZ

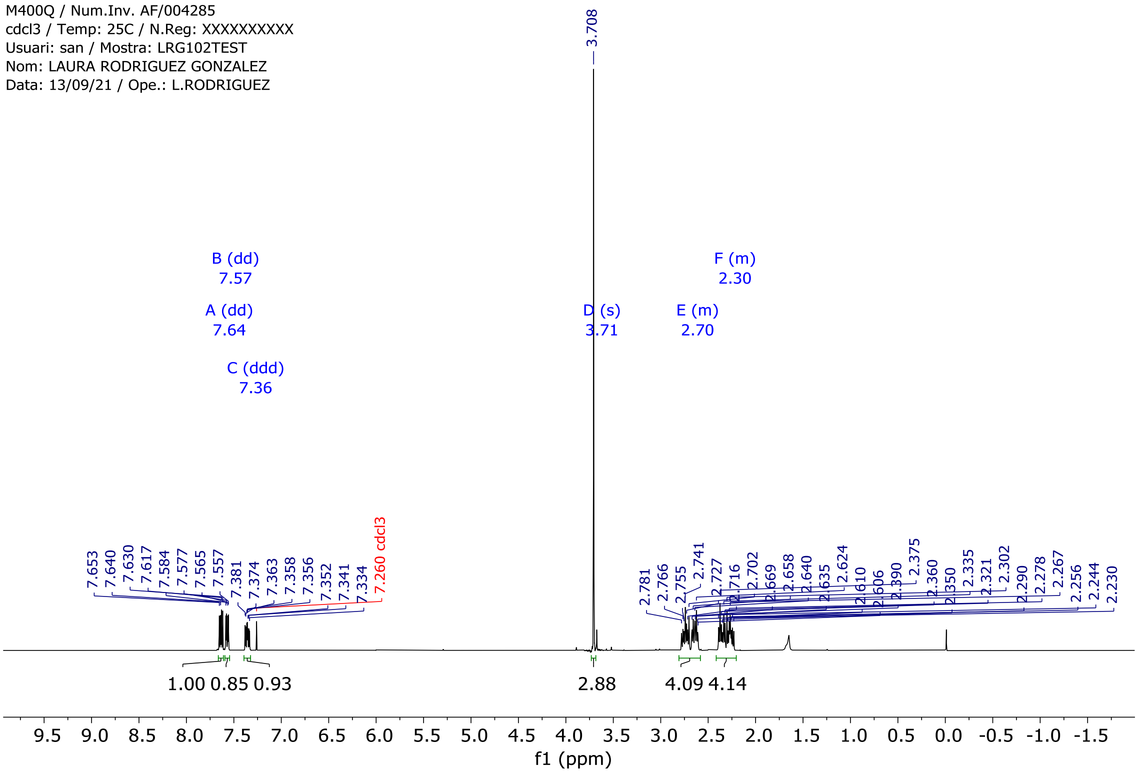

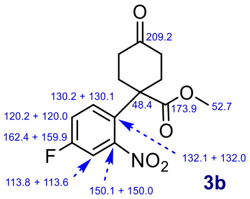

3b

san-1643-2021.2.fid LRG102-13C{1H} 101 MHz
Equip: B400Q / N.Inv: 1009989
N.Reg:
Usuari: / Mostra:
Nom:
Data: 13/09/2021 18:09:50 h./ Ope.:

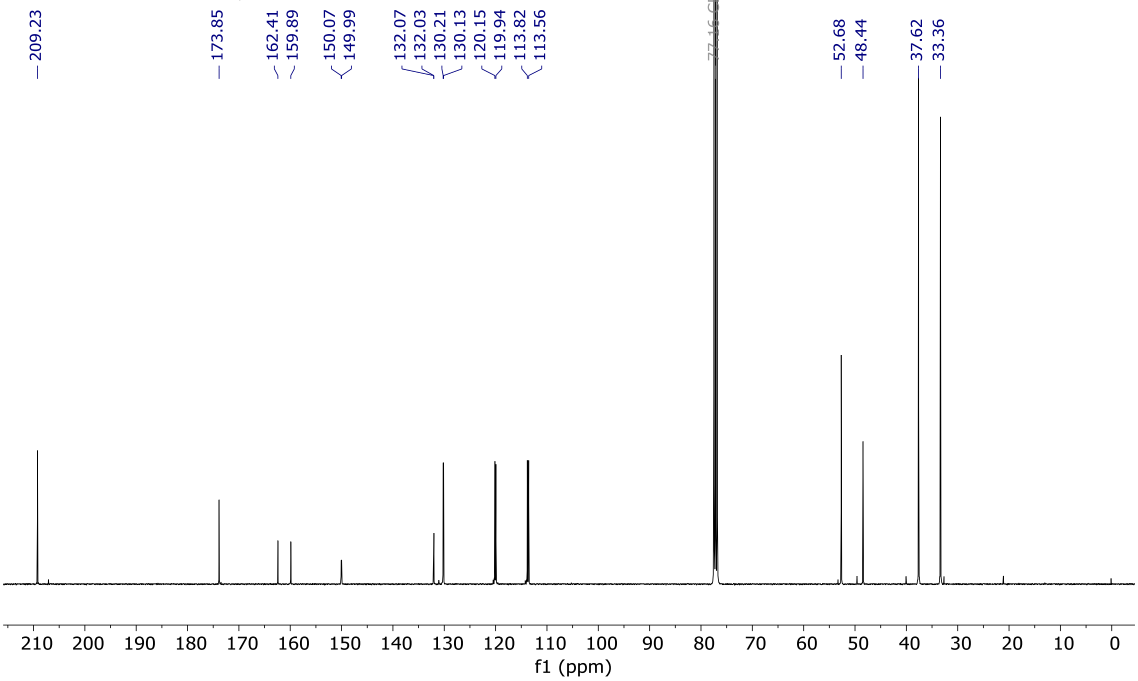

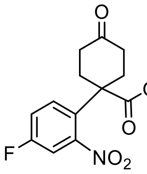

3b

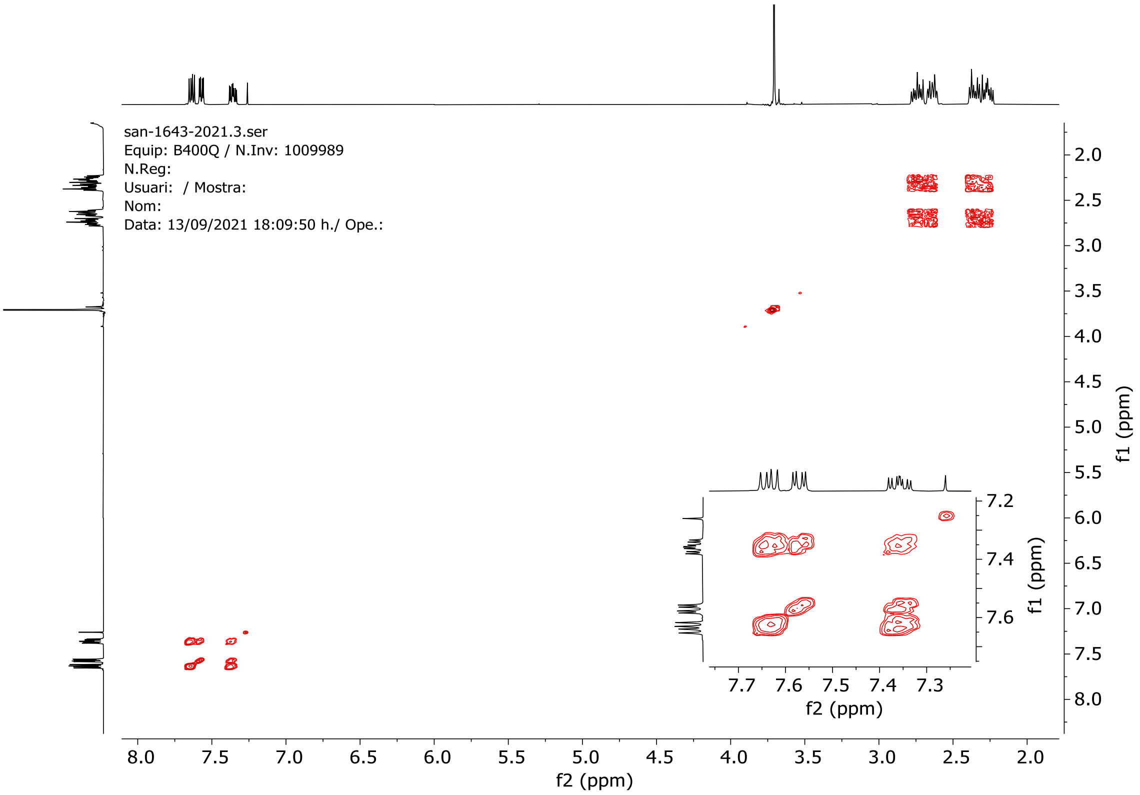
san-1643-2021.3.ser
Equip: B400Q / N.Inv: 1009989
N.Reg:
Usuari: / Mostra:
Nom:
Data: 13/09/2021 18:09:50 h./ Ope.:
f1 (ppm)
f2 (ppm)

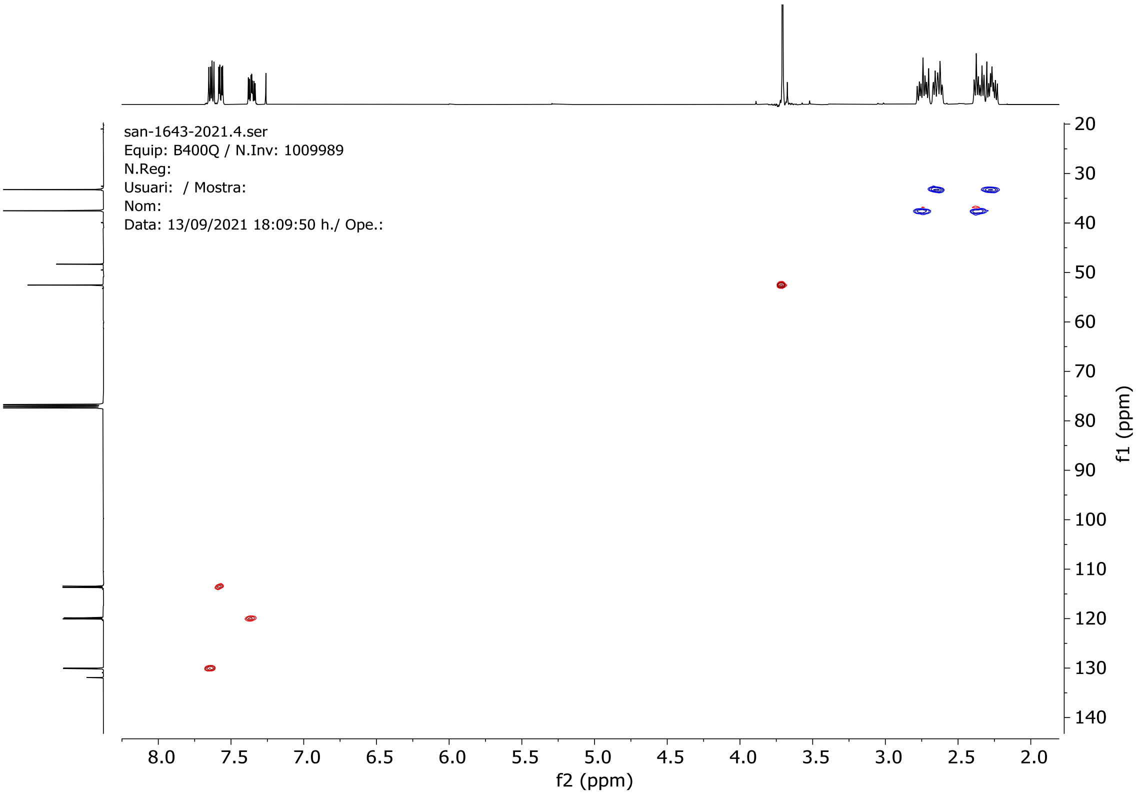
san-1643-2021.4.ser
Equip: B400Q / N.Inv: 1009989
N.Reg:
Usuari: / Mostra:
Nom:
Data: 13/09/2021 18:09:50 h./ Ope.:
f1 (ppm)
f2 (ppm)

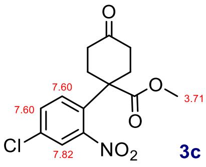

3c

san-1662-2021.1.fid 400 MHz
Equip: B400Q / N.Inv: 1009989
N.Reg: 1662/2021
Usuari: san / Mostra: LRG105ch
Nom: LAURA RODRIGUEZ GONZALEZ
Data: 15/09/2021 19:53:58 h./ Ope.: ALBERT GALLEN

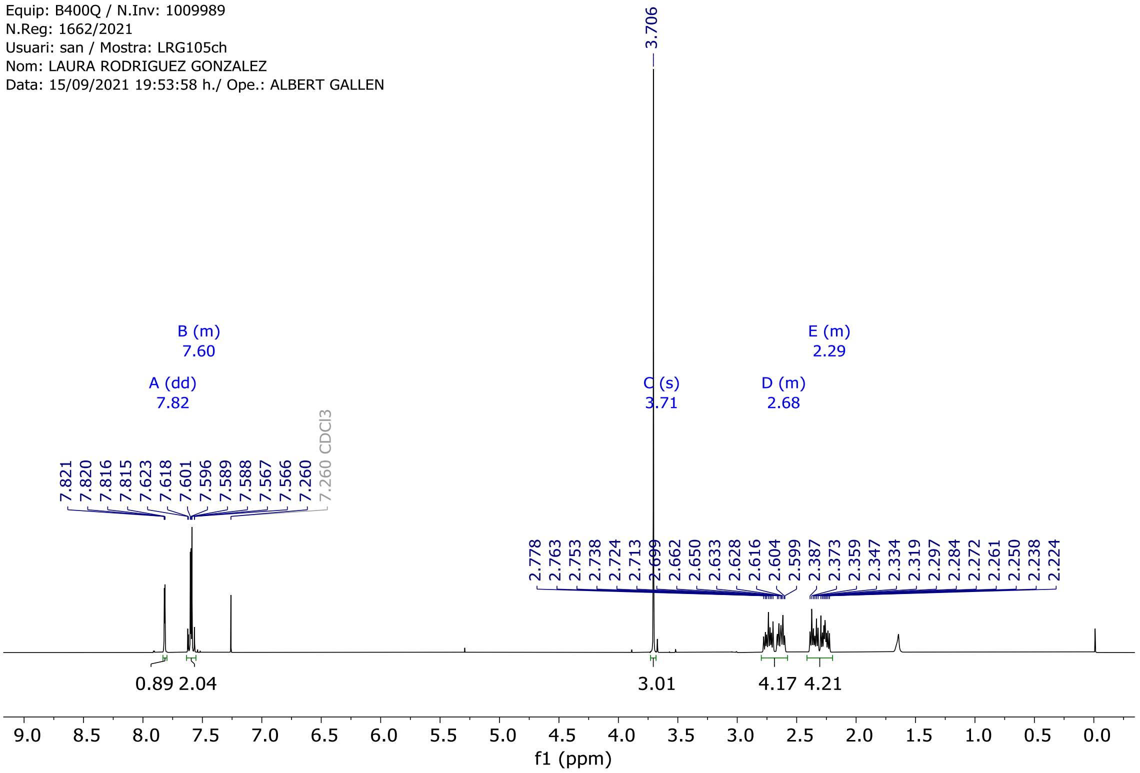

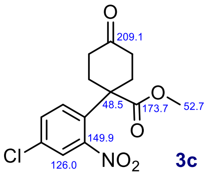

3c

san-1662-2021.2.fid LRG105-13C{1H} 101 MHz
Equip: B400Q / N.Inv: 1009989
N.Reg: 1662/2021
Usuari: san / Mostra: LRG105ch
Nom: LAURA RODRIGUEZ GONZALEZ
Data: 15/09/2021 19:53:58 h./ Ope.: ALBERT GALLEN

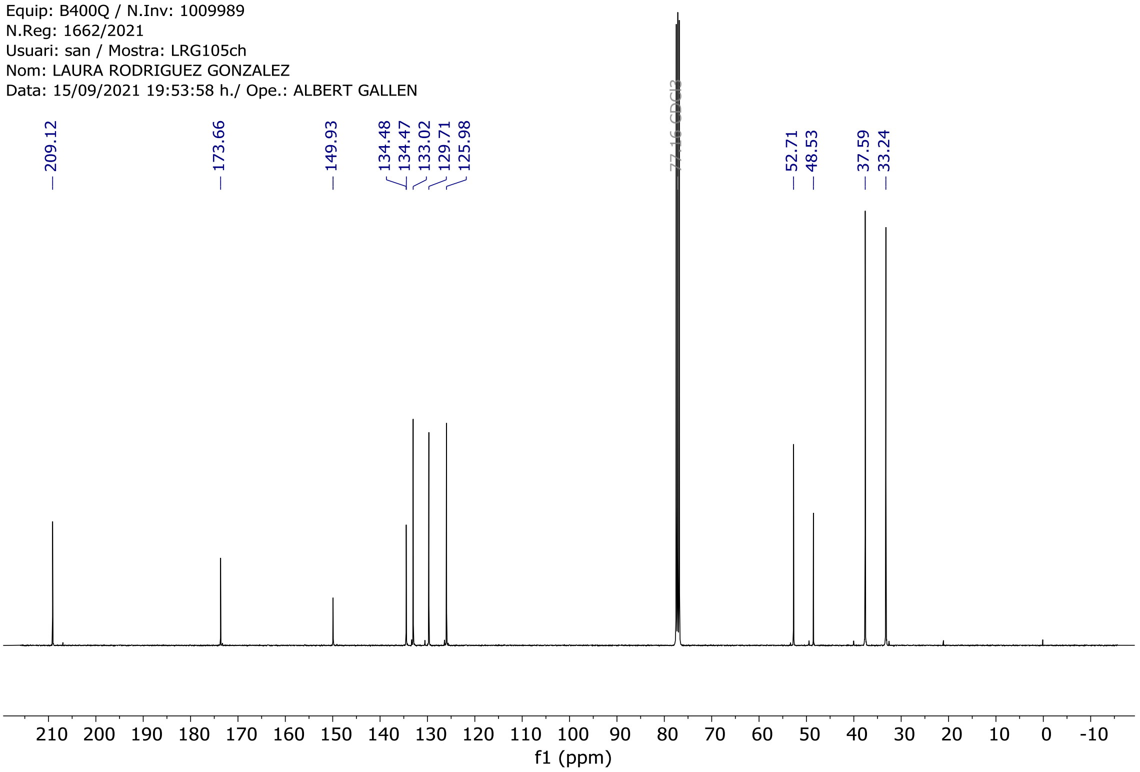

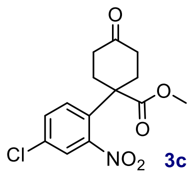
O
O
O
Cl
NO2

3c

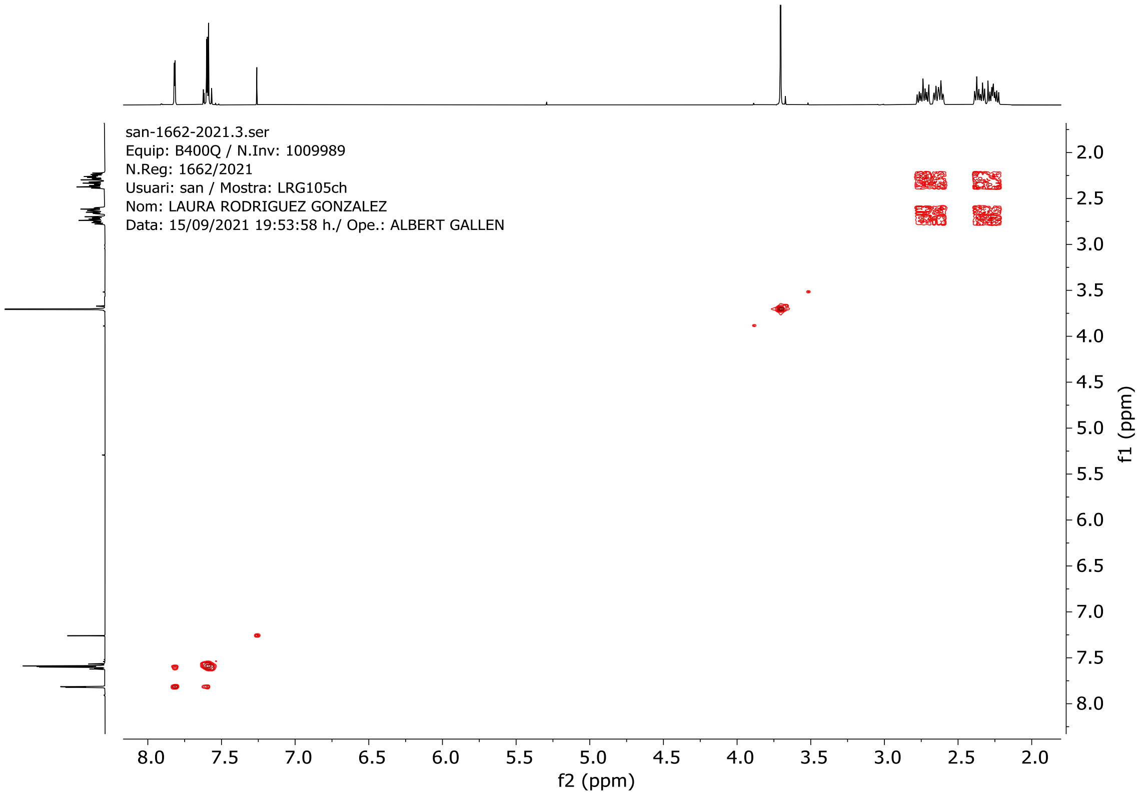
san-1662-2021.3.ser
Equip: B400Q / N.Inv: 1009989
N.Reg: 1662/2021
Usuari: san / Mostra: LRG105ch
Nom: LAURA RODRIGUEZ GONZALEZ
Data: 15/09/2021 19:53:58 h./ Ope.: ALBERT GALLEN
f1 (ppm)
f2 (ppm)

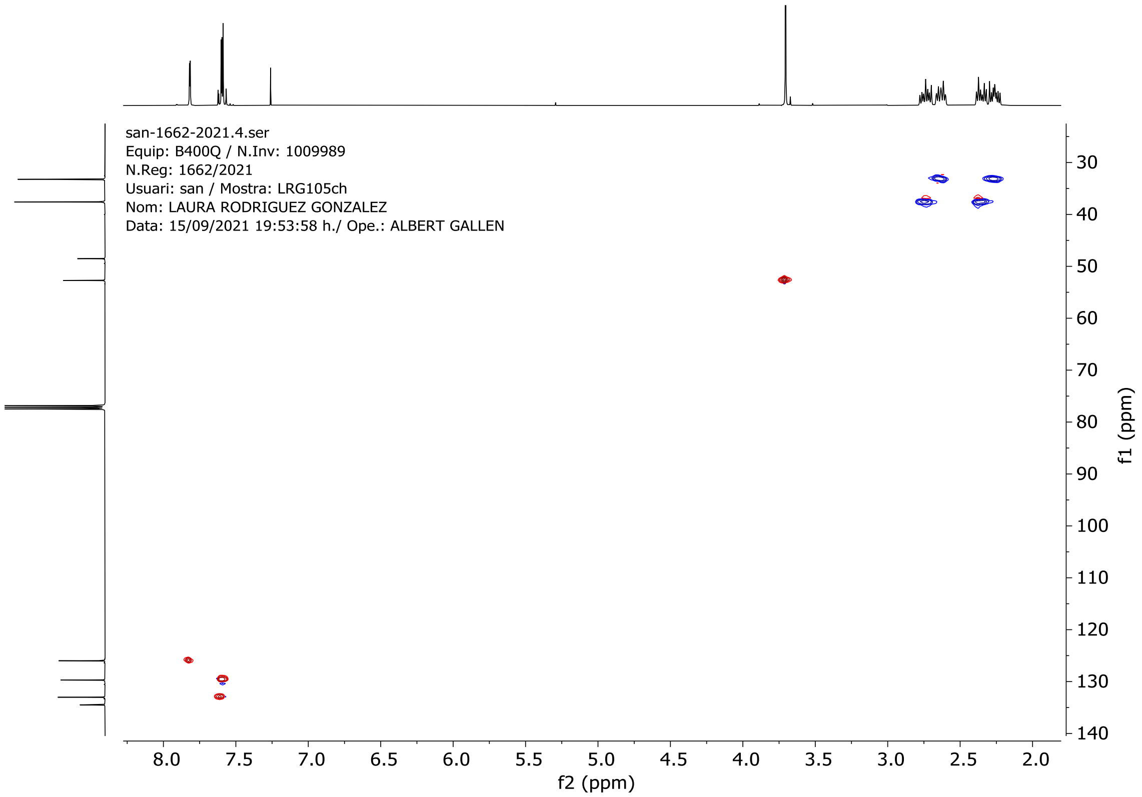
san-1662-2021.4.ser
Equip: B400Q / N.Inv: 1009989
N.Reg: 1662/2021
Usuari: san / Mostra: LRG105ch
Nom: LAURA RODRIGUEZ GONZALEZ
Data: 15/09/2021 19:53:58 h./ Ope.: ALBERT GALLEN
f1 (ppm)
f2 (ppm)

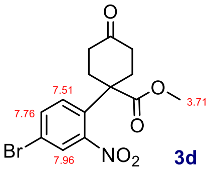

3d

M400AQUI_21102021_LRG090DRY-H1 400 MHz
M400Q / Num.Inv. AF/004285
cdcl3 / Temp: 25C / N.Reg: XXXXXXXXXX
Usuari: san / Mostra: LRG090DRY
Nom: LAURA RODRIGUEZ GONZALEZ
Data: 21/10/21 / Ope.: L.RODRIGUEZ

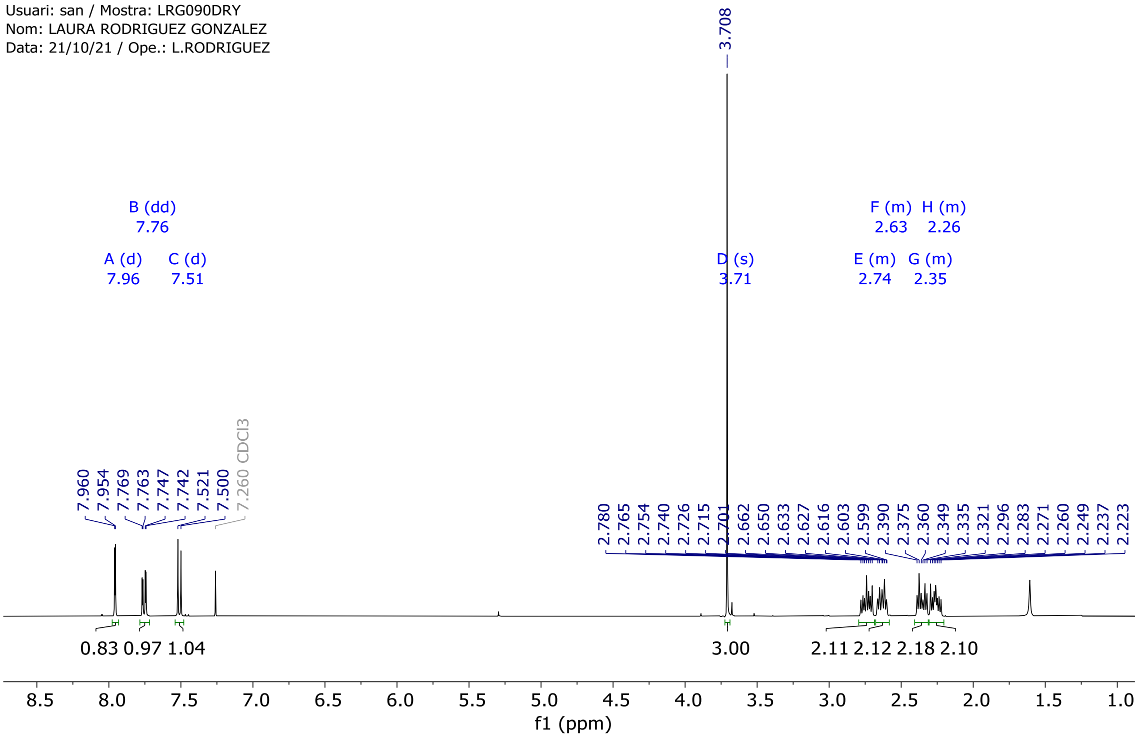

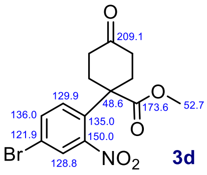

3d

M400AQUI_19072021_LRG090CH-C13{1H} 101 MHz
M400Q / Num.Inv. AF/004285
cdcl3 / Temp: 25C / N.Reg: XXXXXXXXXX
Usuari: san / Mostra: LRG090CH
Nom: LAURA RODRIGUEZ GONZALEZ
Data: 19/07/21 / Ope.: L.RODRIGUEZ

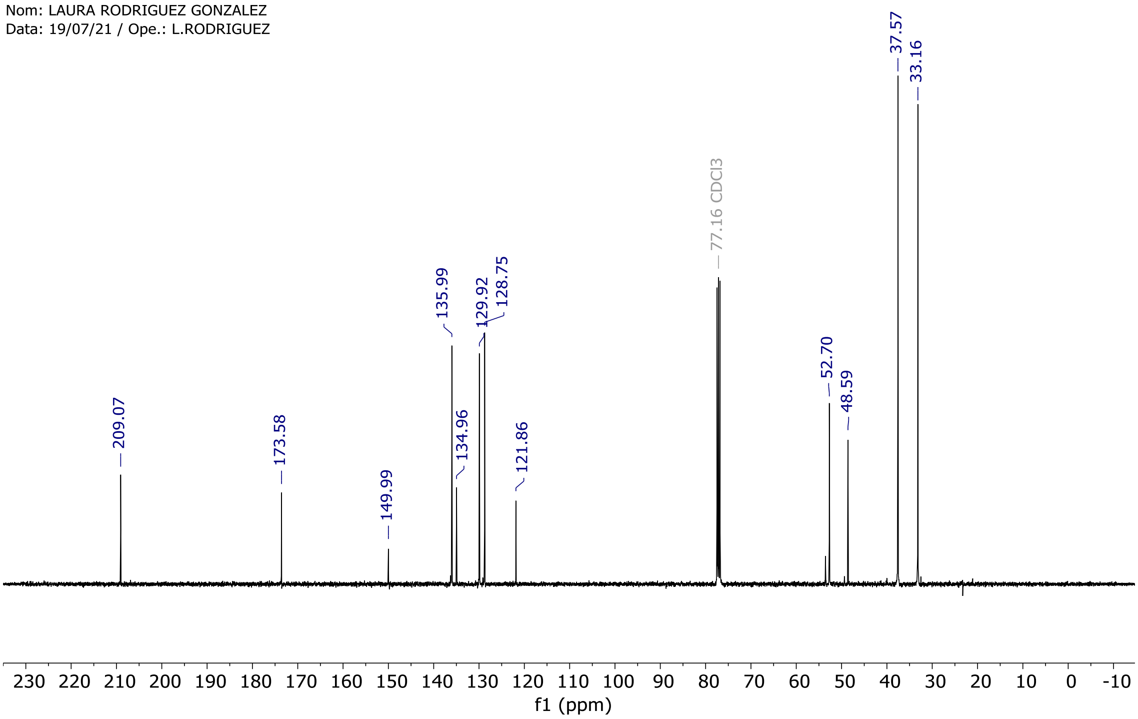

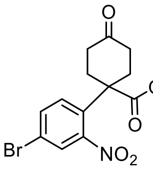
O
O
O
Br
NO2

3d

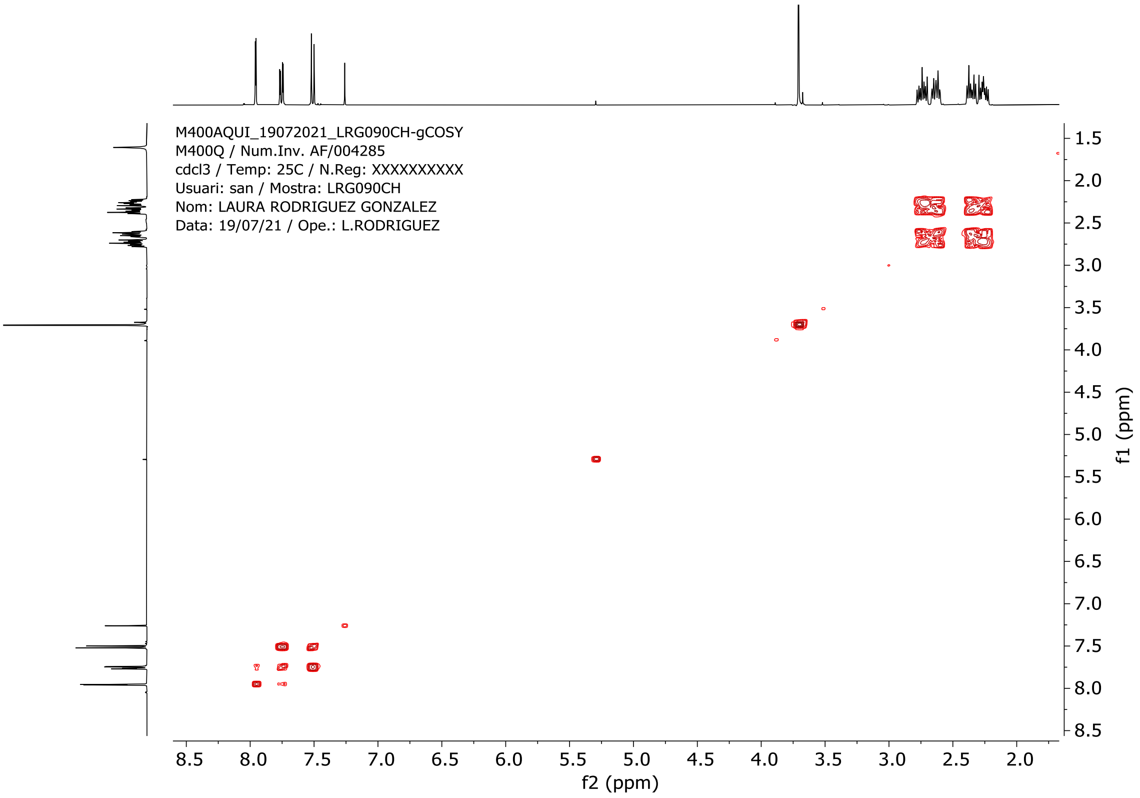
M400AQUI_19072021_LRG090CH-gCOSY
M400Q / Num.Inv. AF/004285
cdcl3 / Temp: 25C / N.Reg: XXXXXXXXXX
Usuari: san / Mostra: LRG090CH
Nom: LAURA RODRIGUEZ GONZALEZ
Data: 19/07/21 / Ope.: L.RODRIGUEZ
f2 (ppm)
f1 (ppm)

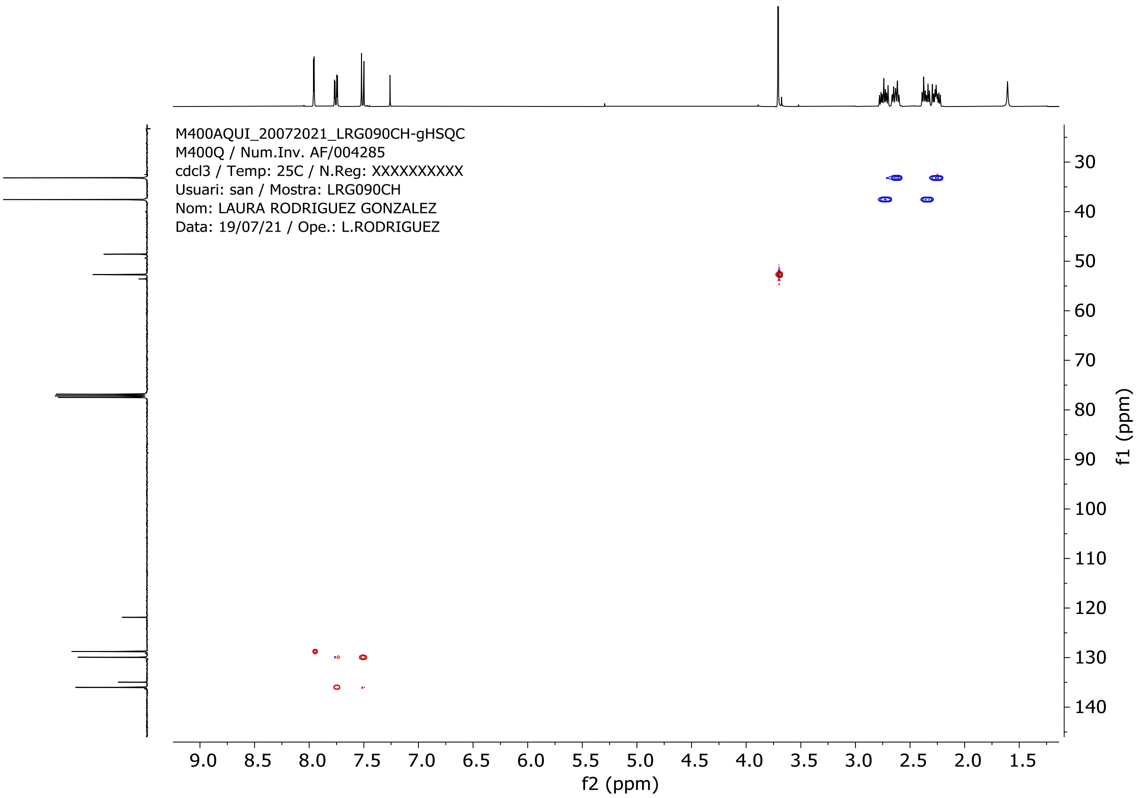
M400AQUI_20072021_LRG090CH-gHSQC
M400Q / Num.Inv. AF/004285
cdcl3 / Temp: 25C / N.Reg: XXXXXXXXXX
Usuari: san / Mostra: LRG090CH
Nom: LAURA RODRIGUEZ GONZALEZ
Data: 19/07/21 / Ope.: L.RODRIGUEZ
f2 (ppm)
f1 (ppm)

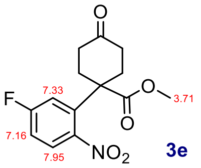

3e

M400AQUI_21102021_LRG087DRY-H1 400 MHz
M400Q / Num.Inv. AF/004285
cdcl3 / Temp: 25C / N.Reg: XXXXXXXXXX
Usuari: san / Mostra: LRG087DRY
Nom: LAURA RODRIGUEZ GONZALEZ
Data: 21/10/21 / Ope.: L.RODRIGUEZ

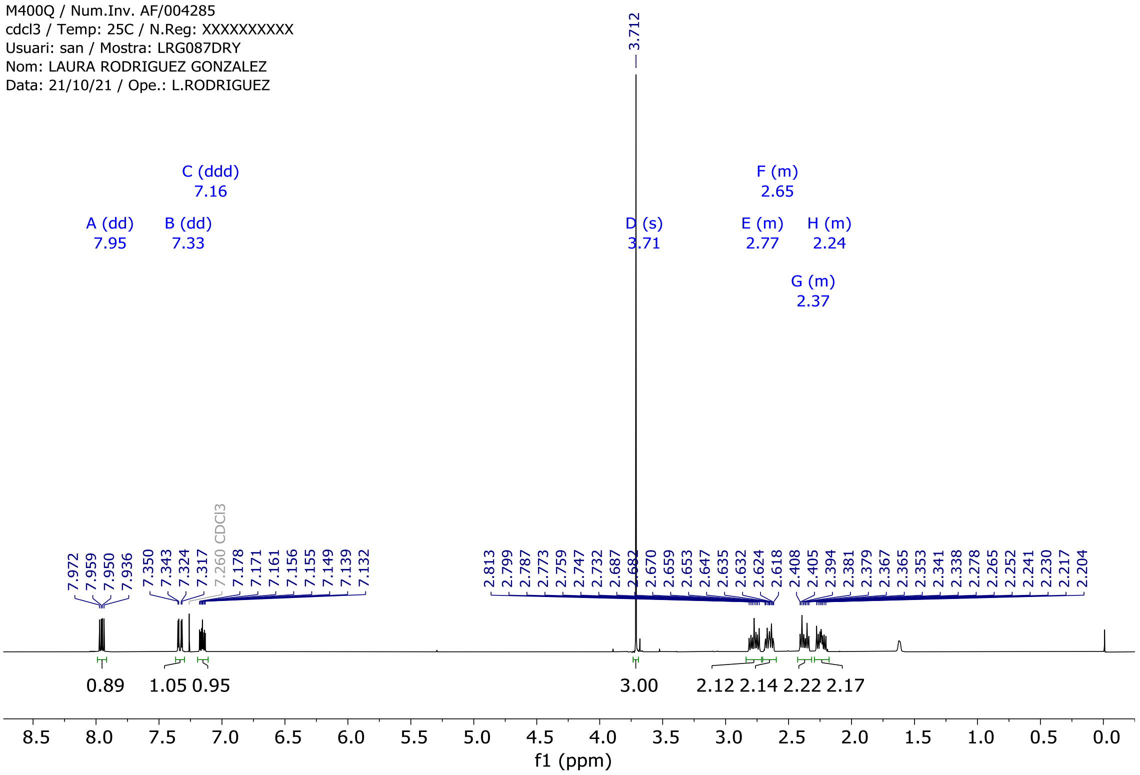

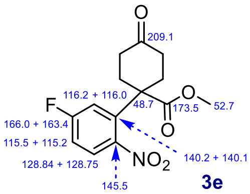

3e

M400AQUI_14072021_LRG087CHARAC-C13{1H} 101 MHz
M400Q / Num.Inv. AF/004285
cdcl3 / Temp: 25C / N.Reg: XXXXXXXXXX
Usuari: san / Mostra: LRG087CHARAC
Nom: LAURA RODRIGUEZ GONZALEZ
Data: 14/07/21 / Ope.: L.RODRIGUEZ

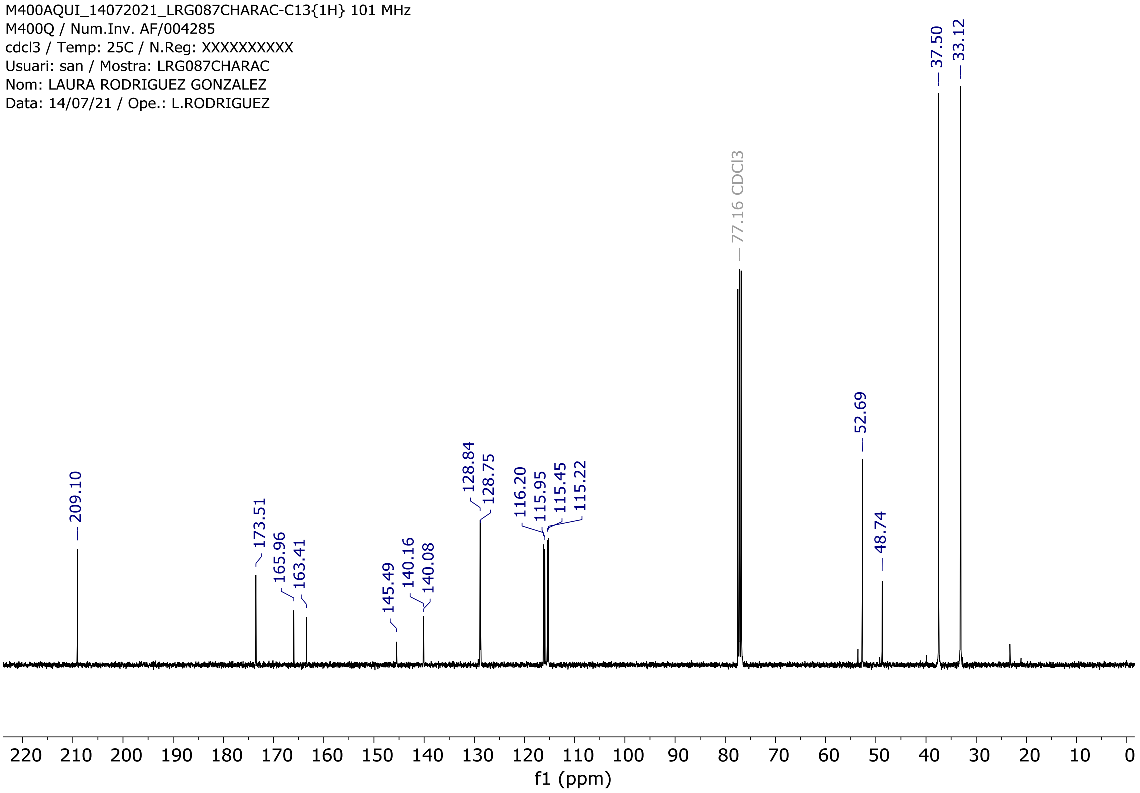

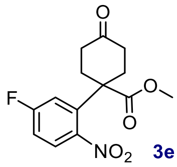
F
NO2
O

3e

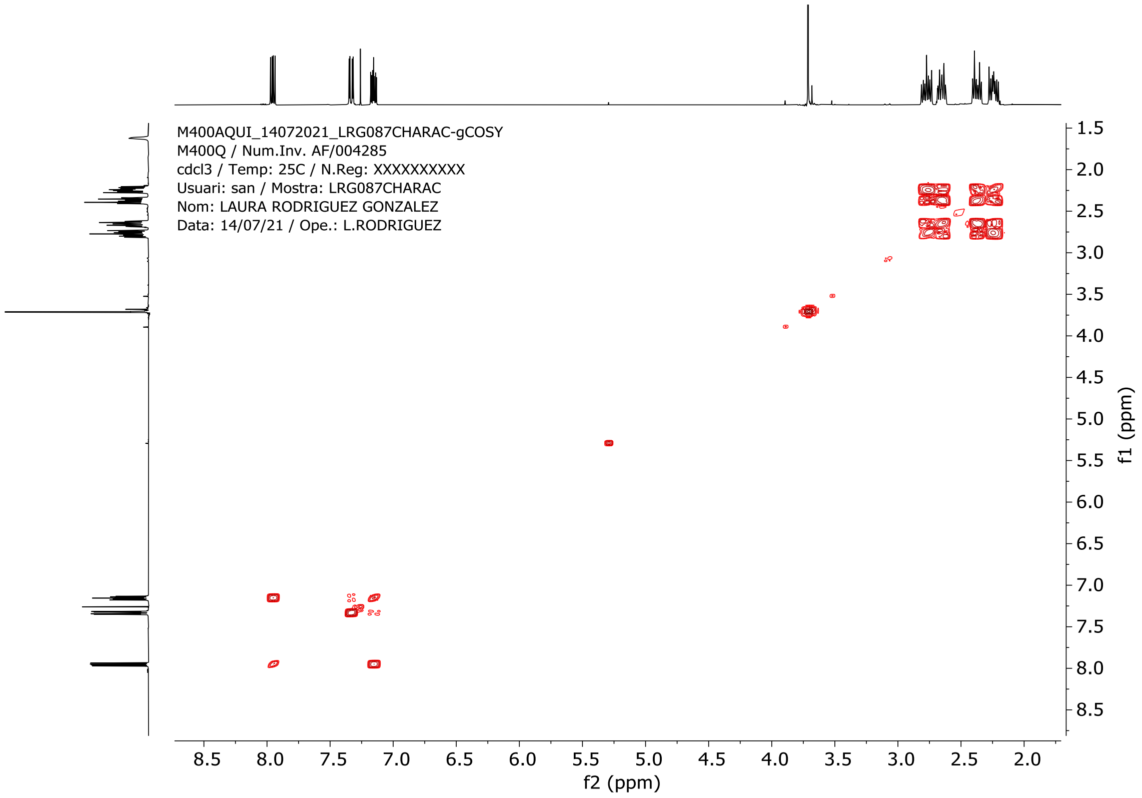
M400AQUI_14072021_LRG087CHARAC-gCOSY
M400Q / Num.Inv. AF/004285
cdcl3 / Temp: 25C / N.Reg: XXXXXXXXXX
Usuari: san / Mostra: LRG087CHARAC
Nom: LAURA RODRIGUEZ GONZALEZ
Data: 14/07/21 / Ope.: L.RODRIGUEZ
f2 (ppm)
f1 (ppm)

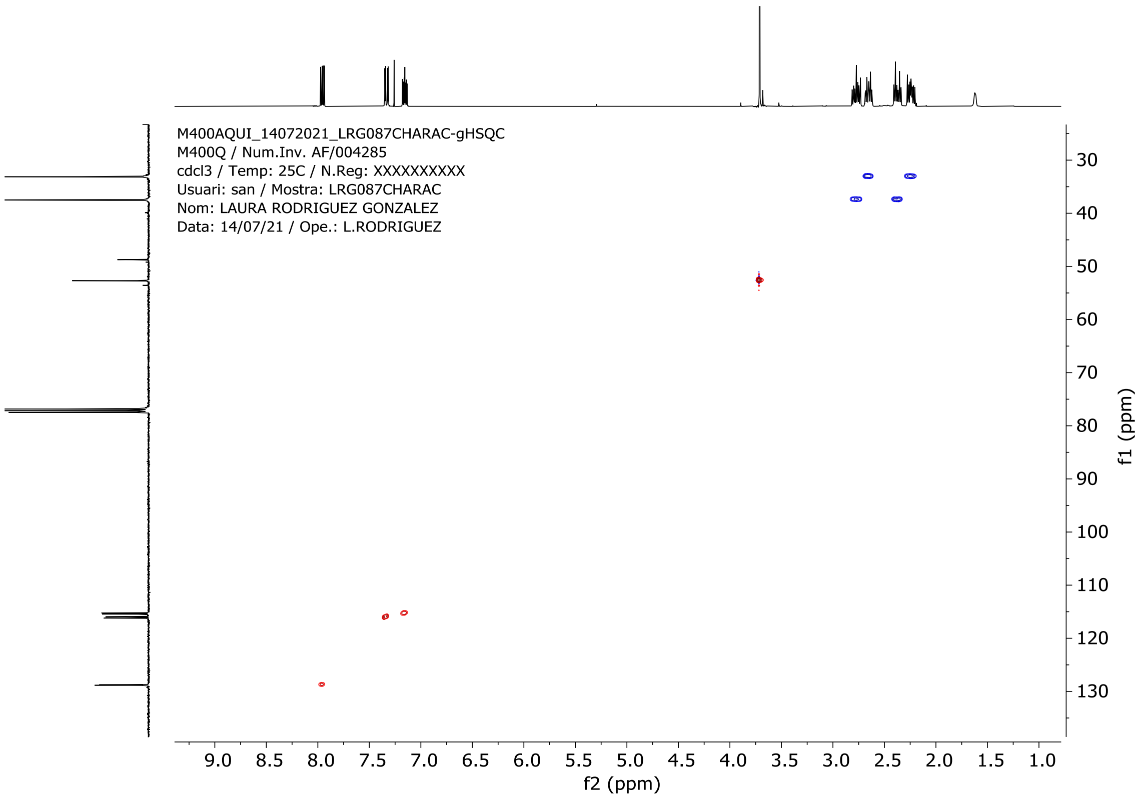
M400AQUI_14072021_LRG087CHARAC-gHSQC
M400Q / Num.Inv. AF/004285
cdcl3 / Temp: 25C / N.Reg: XXXXXXXXXX
Usuari: san / Mostra: LRG087CHARAC
Nom: LAURA RODRIGUEZ GONZALEZ
Data: 14/07/21 / Ope.: L.RODRIGUEZ
f2 (ppm)
f1 (ppm)

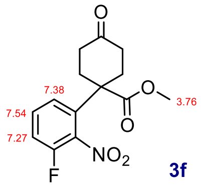

M400AQUI_21092021_LRG110DRY-H1 400 MHz
M400Q / Num.Inv. AF/004285
cdcl3 / Temp: 25C / N.Reg: XXXXXXXXXX
Usuari: san / Mostra: LRG110DRY
Nom: LAURA RODRIGUEZ GONZALEZ
Data: 21/09/21 / Ope.: L.RODRIGUEZ

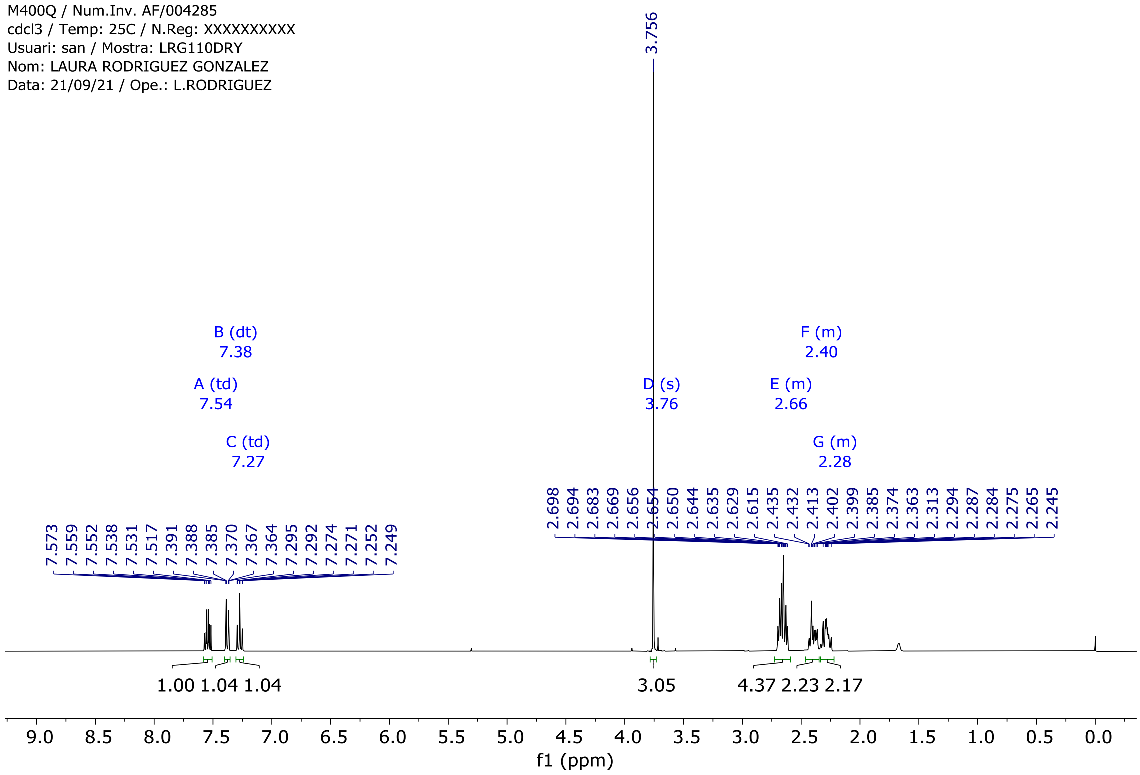

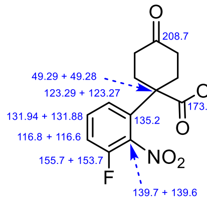

LRG110bruker500.2.fid LRG110-13C{1H} 126 MHz
Equip: B500Q / N.Inv: 1028917
N.Reg: 1715/2021
Usuari: san / Mostra: LRG110ch
Nom: LAURA RODRIGUEZ GONZALEZ
Data: 20/09/2021 15:35:26 h./ Ope.: VICTOR MERIEL

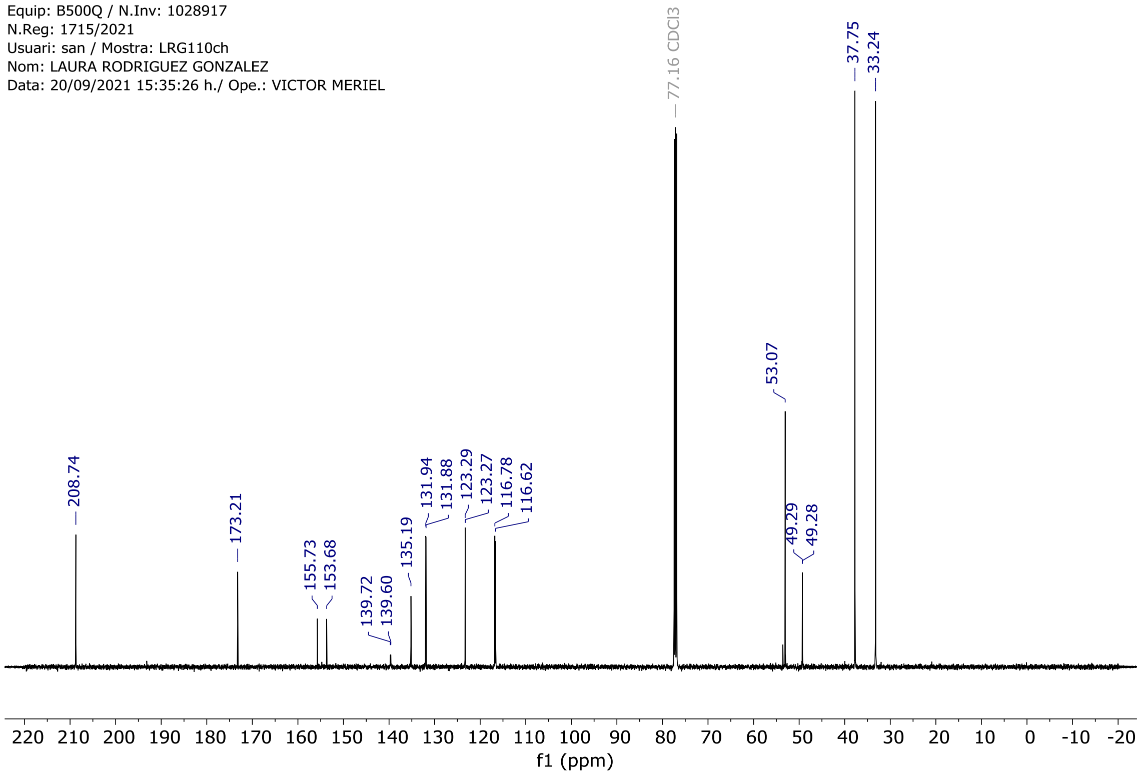

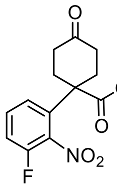
O
O
O
NO2
F

3f

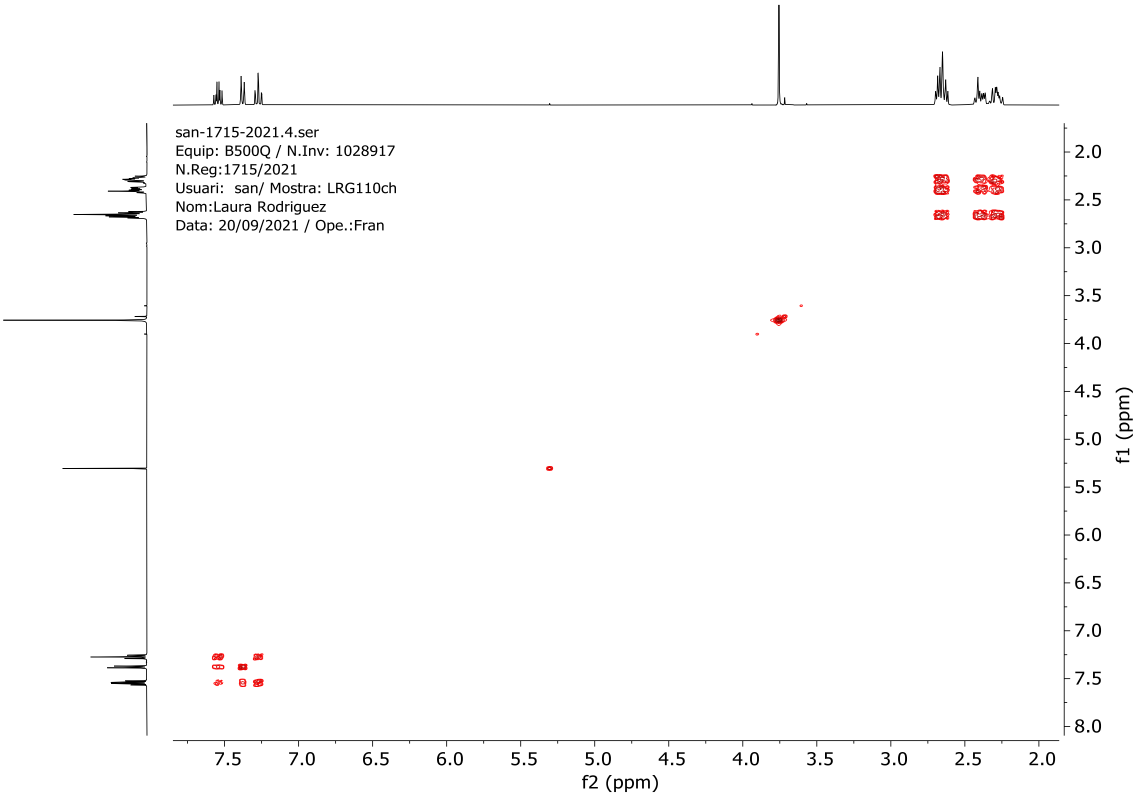
san-1715-2021.4.ser
Equip: B500Q / N.Inv: 1028917
N.Reg:1715/2021
Usuari: san/ Mostra: LRG110ch
Nom:Laura Rodriguez
Data: 20/09/2021 / Ope.:Fran
f2 (ppm)
f1 (ppm)

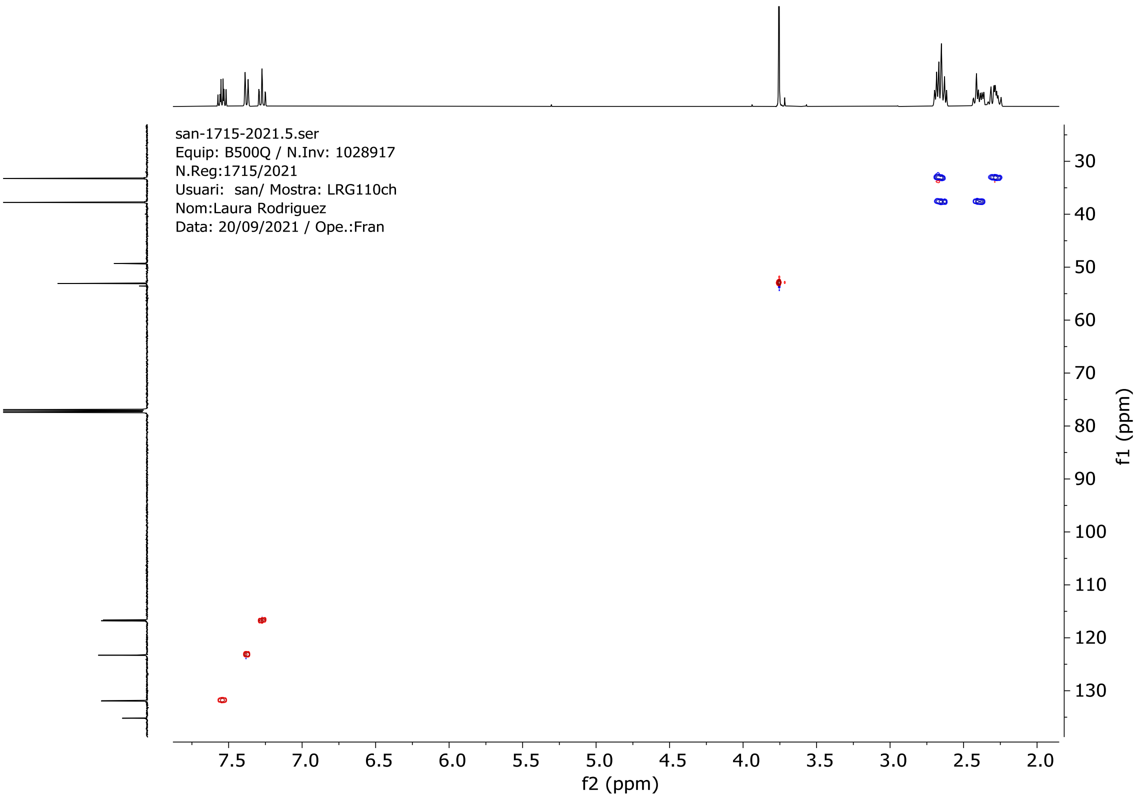
san-1715-2021.5.ser
Equip: B500Q / N.Inv: 1028917
N.Reg:1715/2021
Usuari: san/ Mostra: LRG110ch
Nom:Laura Rodriguez
Data: 20/09/2021 / Ope.:Fran
f2 (ppm)
f1 (ppm)

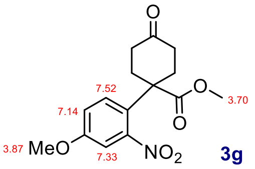

3g

M400AQUI_01102021_LRG117-H1 400 MHz
M400Q / Num.Inv. AF/004285
cdcl3 / Temp: 25C / N.Reg: XXXXXXXXXX
Usuari: san / Mostra: LRG117
Nom: LAURA RODRIGUEZ GONZALEZ
Data: 01/10/21 / Ope.: L.RODRIGUEZ

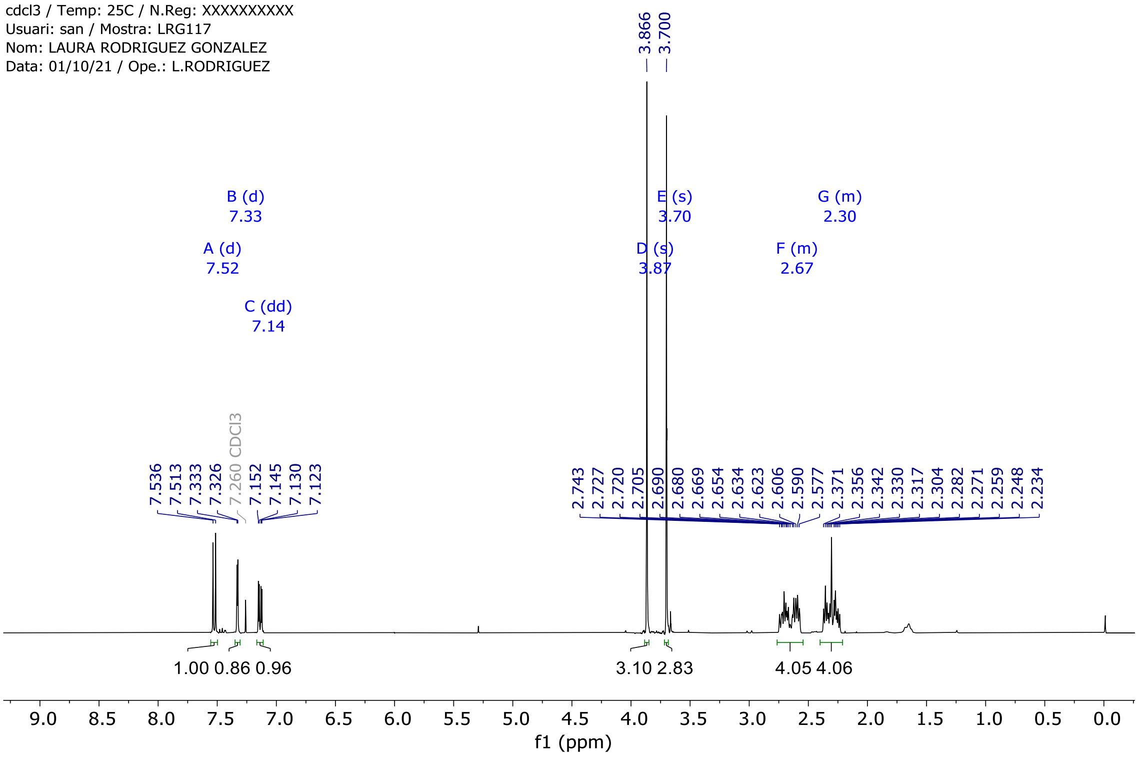

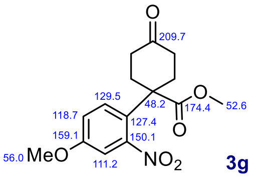

3g

M400AQUI_01102021_LRG117-C13{1H} 101 MHz
M400Q / Num.Inv. AF/004285
cdcl3 / Temp: 25C / N.Reg: XXXXXXXXXX
Usuari: san / Mostra: LRG117
Nom: LAURA RODRIGUEZ GONZALEZ
Data: 01/10/21 / Ope.: L.RODRIGUEZ

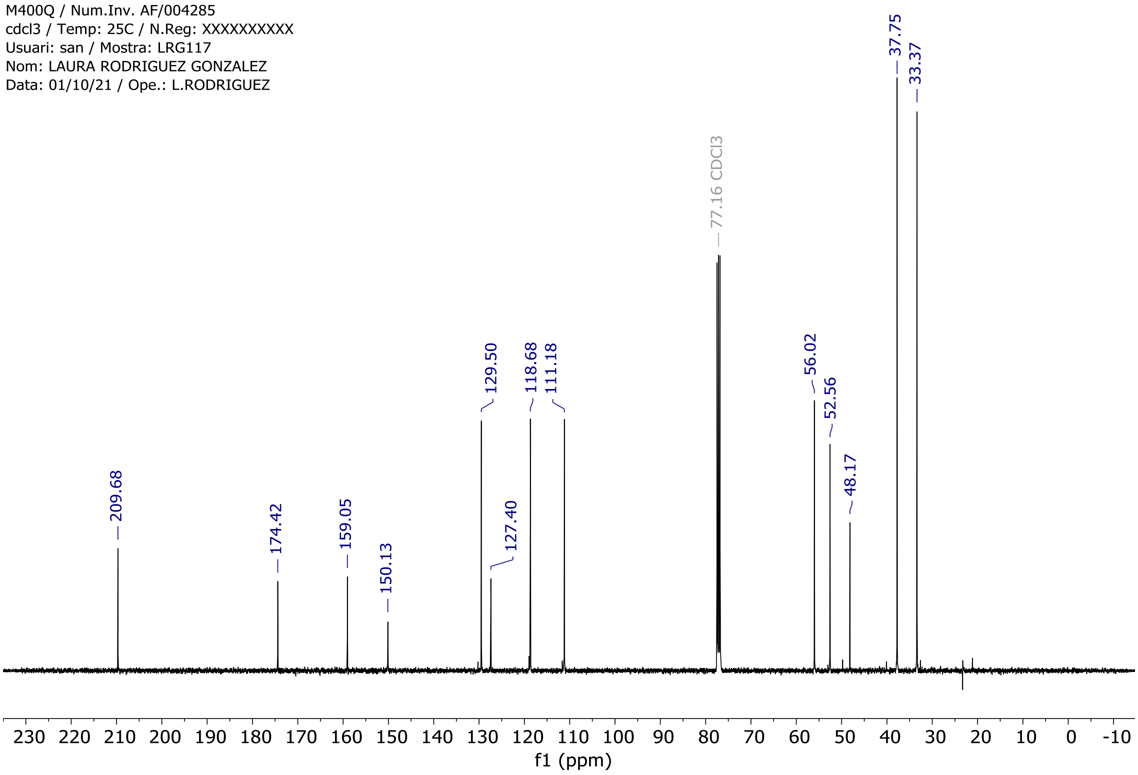

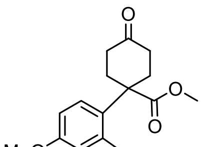

3g

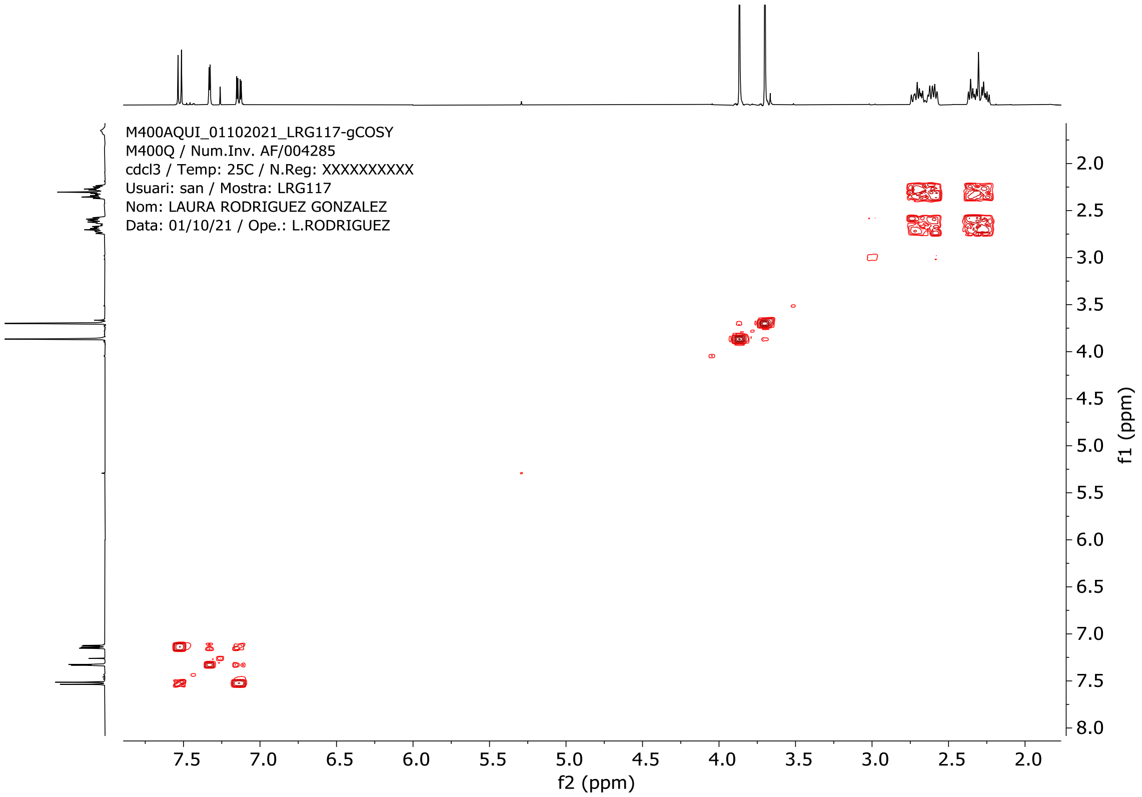
M400AQUI_01102021_LRG117-gCOSY
M400Q / Num.Inv. AF/004285
cdcl3 / Temp: 25C / N.Reg: XXXXXXXXXX
Usuari: san / Mostra: LRG117
Nom: LAURA RODRIGUEZ GONZALEZ
Data: 01/10/21 / Ope.: L.RODRIGUEZ
f1 (ppm)
f2 (ppm)

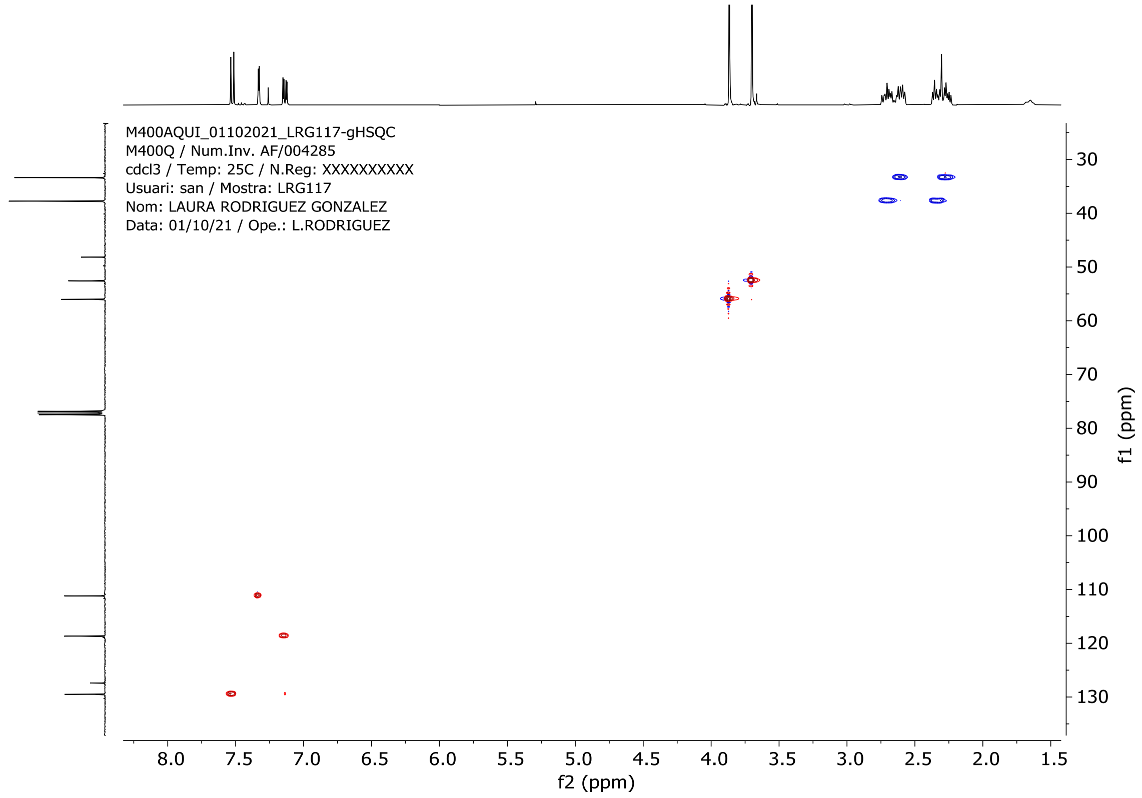
M400AQUI_01102021_LRG117-gHSQC
M400Q / Num.Inv. AF/004285
cdcl3 / Temp: 25C / N.Reg: XXXXXXXXXX
Usuari: san / Mostra: LRG117
Nom: LAURA RODRIGUEZ GONZALEZ
Data: 01/10/21 / Ope.: L.RODRIGUEZ
f1 (ppm)
f2 (ppm)

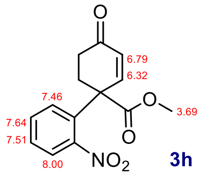

3h

M400APCB_11052021_LRG038CH-H1 400 MHz
M400PCB / Num.Inv. AF/002630
cdcl3 / Temp: 25C / N.Reg: XXXXXXXXXX
Usuari: san / Mostra: LRG038CH
Nom: LAURA RODRIGUEZ GONZALEZ
Data: 10/05/21 / Ope.: L.RODRIGUEZ

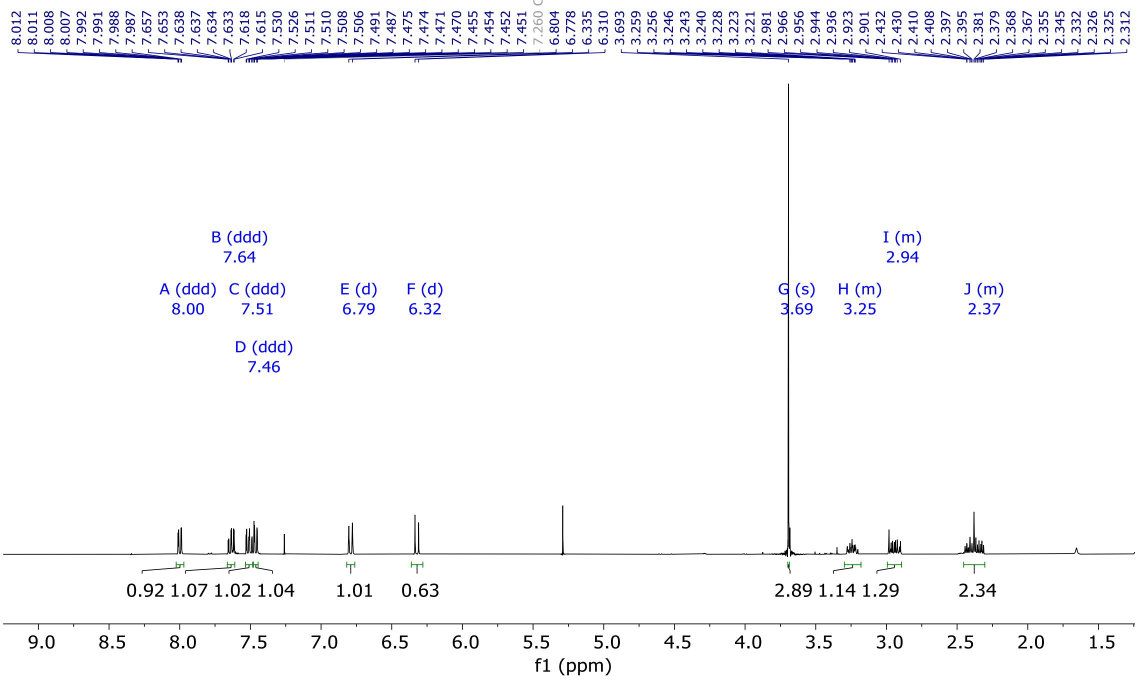

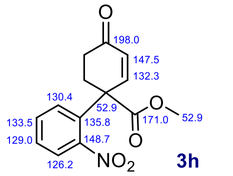

3h

M400APCB_10052021_LRG038CH-C13{1H} 101 MHz
M400PCB / Num.Inv. AF/002630
cdcl3 / Temp: 25C / N.Reg: XXXXXXXXXX
Usuari: san / Mostra: LRG038CH
Nom: LAURA RODRIGUEZ GONZALEZ
Data: 10/05/21 / Ope.: L.RODRIGUEZ

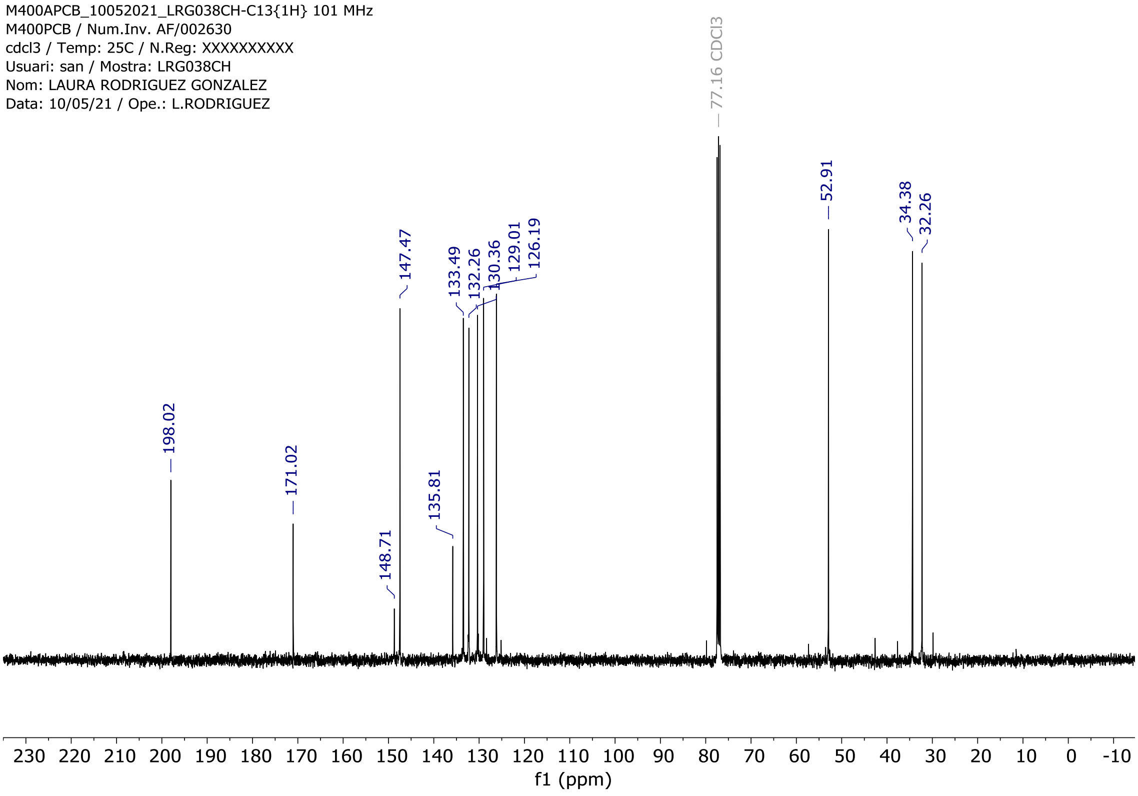

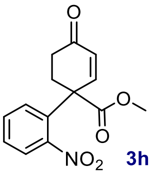

O
O
O
NO_2

3h

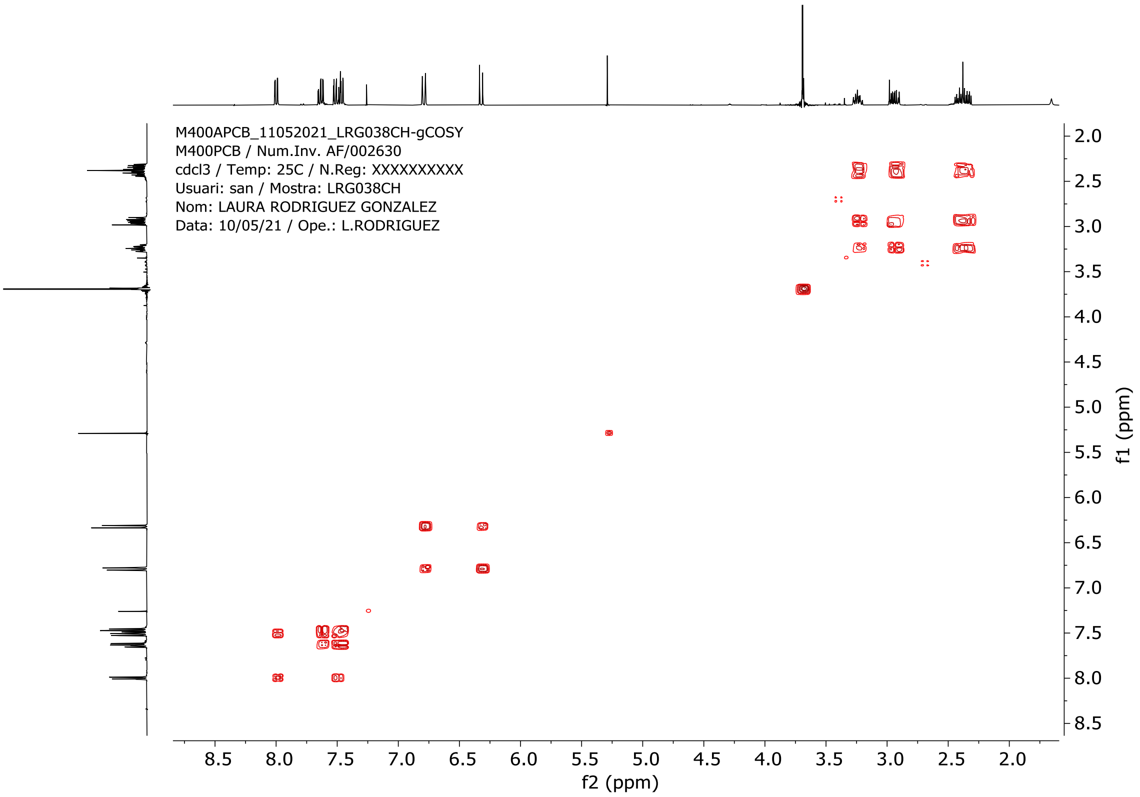

M400APCB_11052021_LRG038CH-gCOSY
M400PCB / Num.Inv. AF/002630
cdcl3 / Temp: 25C / N.Reg: XXXXXXXXXX
Usuari: san / Mostra: LRG038CH
Nom: LAURA RODRIGUEZ GONZALEZ
Data: 10/05/21 / Ope.: L.RODRIGUEZ
f2 (ppm)
f1 (ppm)

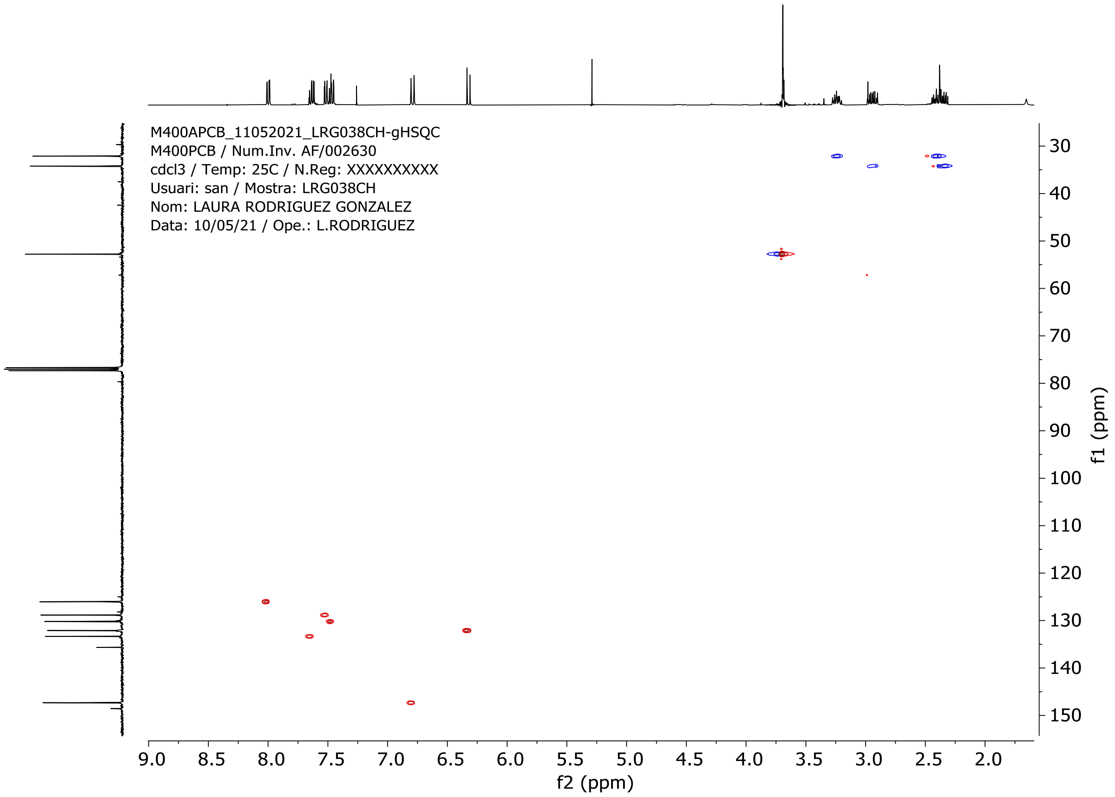

M400APCB_11052021_LRG038CH-gHSQC
M400PCB / Num.Inv. AF/002630
cdcl3 / Temp: 25C / N.Reg: XXXXXXXXXX
Usuari: san / Mostra: LRG038CH
Nom: LAURA RODRIGUEZ GONZALEZ
Data: 10/05/21 / Ope.: L.RODRIGUEZ
f2 (ppm)
f1 (ppm)

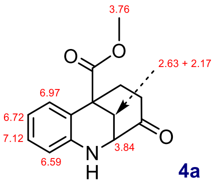

4a

22070260_B400FA_12072022_LRG236P4A.1.fid 400 MHz
Equip: B400F / N.Inv: 1037597
N.Reg: 22070260
Usuari: san / Mostra: LRG236P4A
Nom: LAURA RODRIGUEZ GONZALEZ
Data: 12/07/2022 10:26:27 h./ Ope.: AUTOSERVEI
Experiment: A-H1-zg30 Solvent: CDCl3

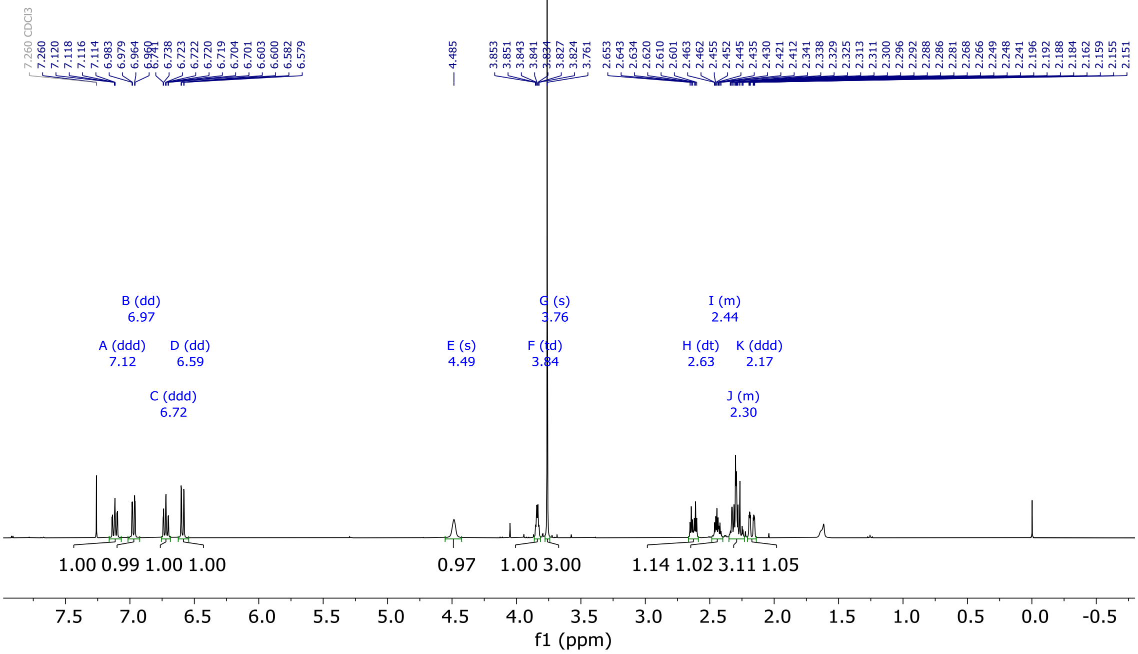

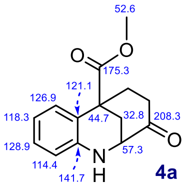

4a

22070260_B400FA_13072022_LRG236P4A.2.fid 13C{H1} 101 MHz
Equip: B400F / N.Inv: 1037597
N.Reg: 22070260
Usuari: san / Mostra: LRG236P4A
Nom: LAURA RODRIGUEZ GONZALEZ
Data: 13/07/2022 03:51:34 h./ Ope.: AUTOSERVEI
Experiment: A-C13-zgpg30 Solvent: CDCl3

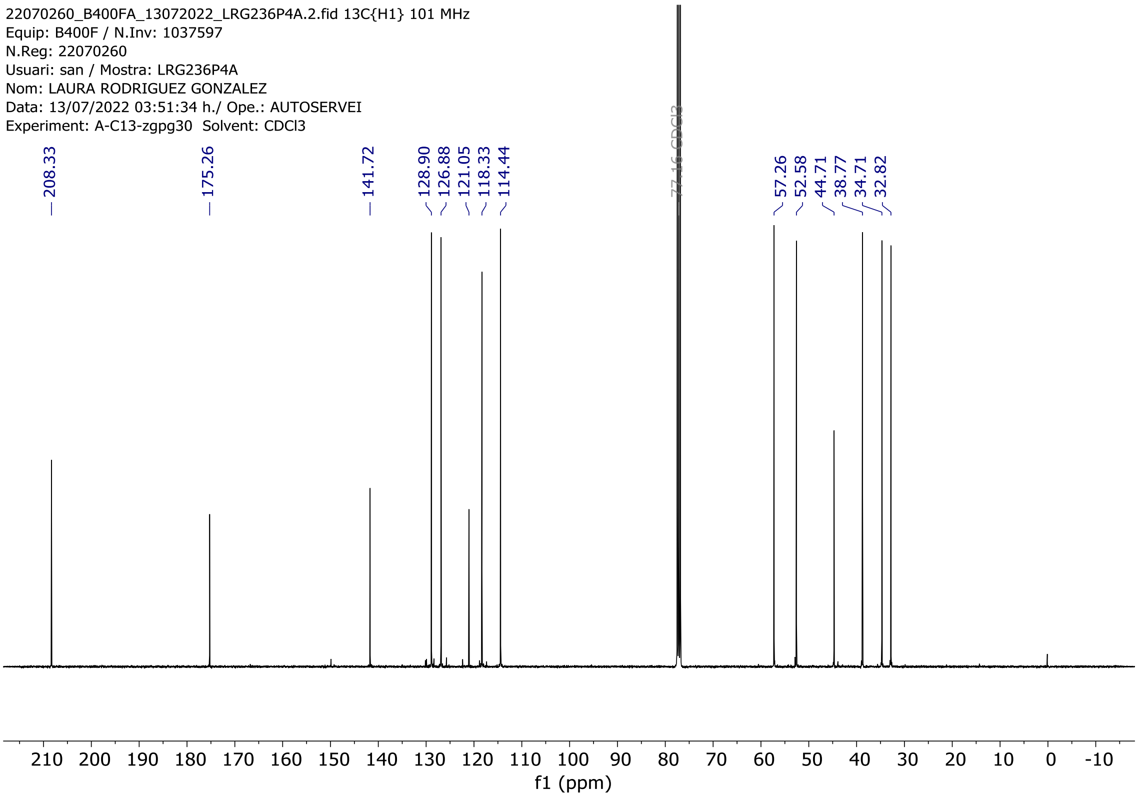

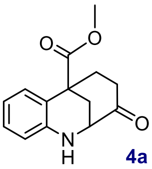

4a

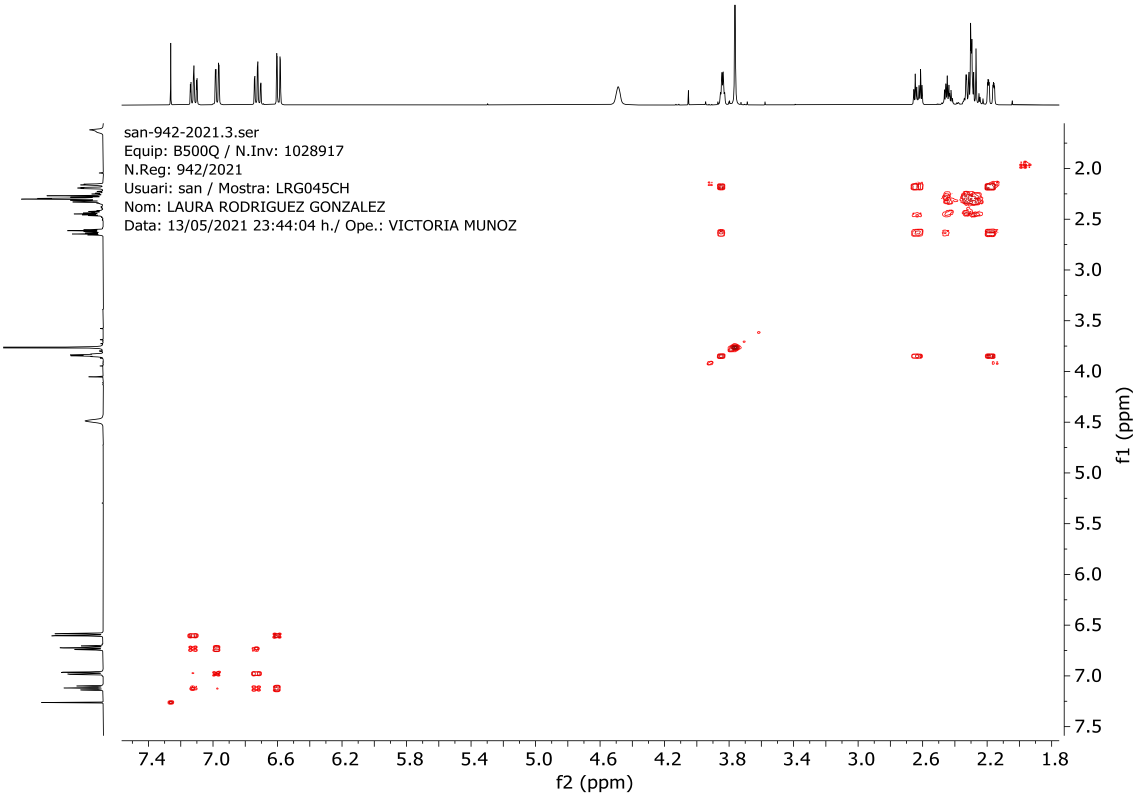
san-942-2021.3.ser
Equip: B500Q / N.Inv: 1028917
N.Reg: 942/2021
Usuari: san / Mostra: LRG045CH
Nom: LAURA RODRIGUEZ GONZALEZ
Data: 13/05/2021 23:44:04 h./ Ope.: VICTORIA MUNOZ
f2 (ppm)
f1 (ppm)

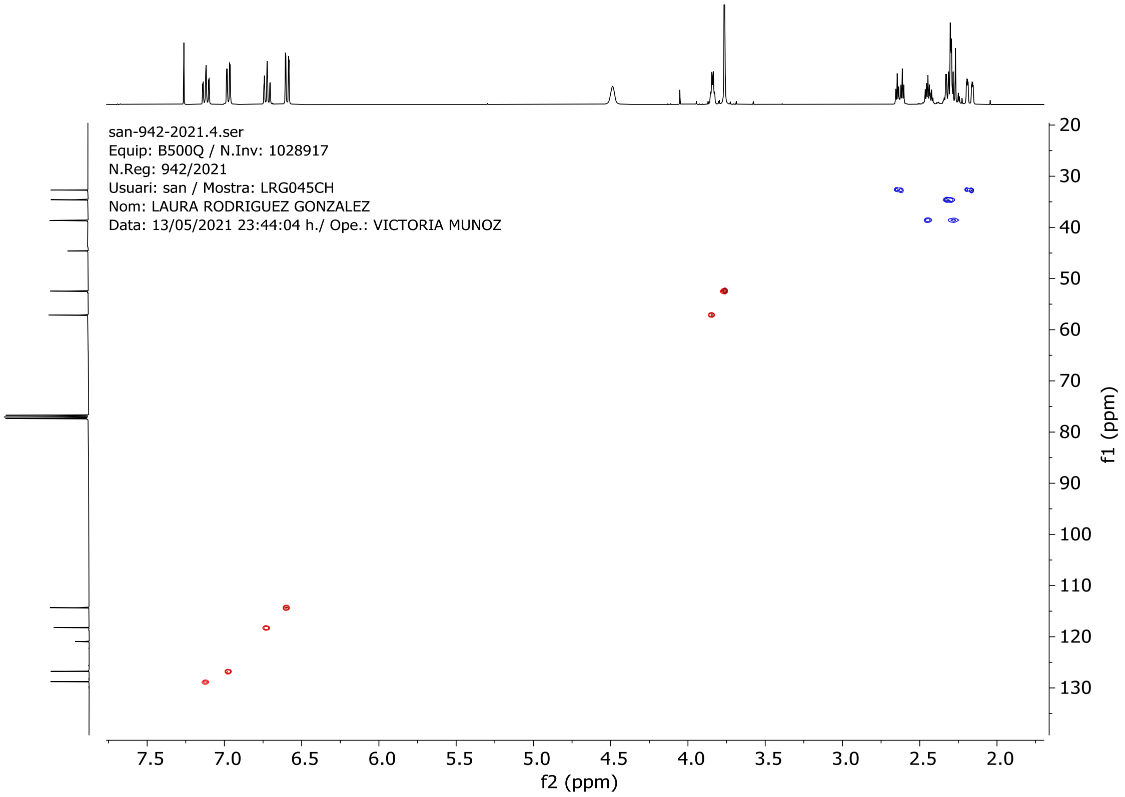
san-942-2021.4.ser
Equip: B500Q / N.Inv: 1028917
N.Reg: 942/2021
Usuari: san / Mostra: LRG045CH
Nom: LAURA RODRIGUEZ GONZALEZ
Data: 13/05/2021 23:44:04 h./ Ope.: VICTORIA MUNOZ
f2 (ppm)
f1 (ppm)

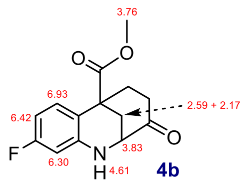

4b

22070385_B400FA_14072022_LRG237PROD4B.1.fid 400 MHz
Equip: B400F / N.Inv: 1037597
N.Reg: 22070385
Usuari: san / Mostra: LRG237PROD4B
Nom: LAURA RODRIGUEZ GONZALEZ
Data: 14/07/2022 13:33:23 h./ Ope.: AUTOSERVEI
Experiment: A-H1-zg30 Solvent: CDCl3

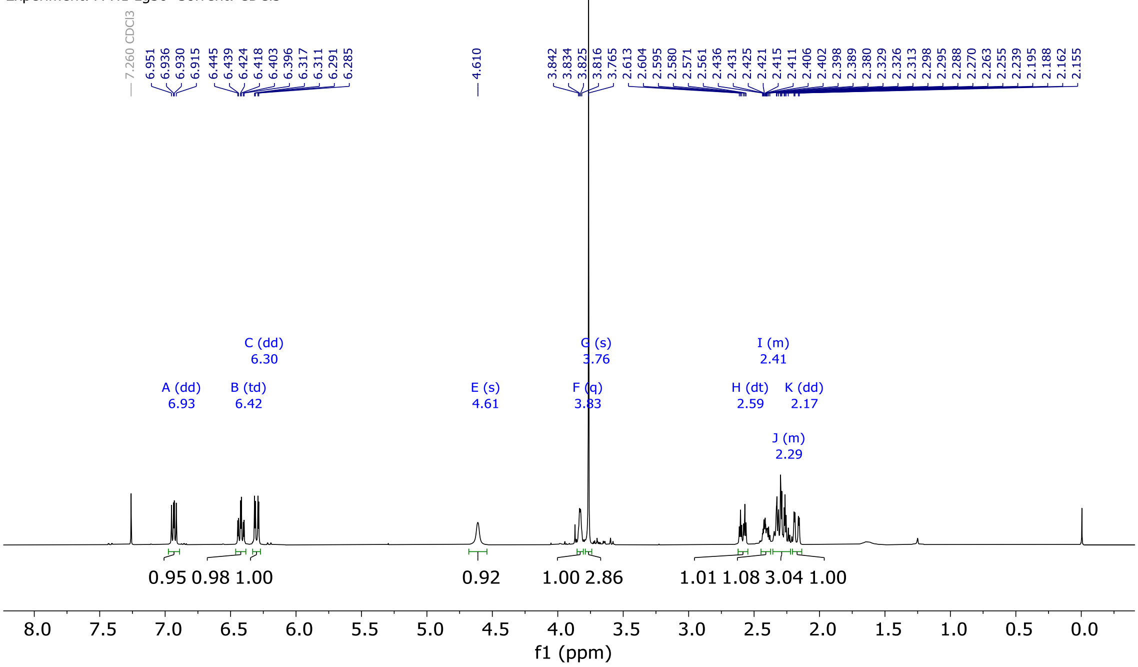

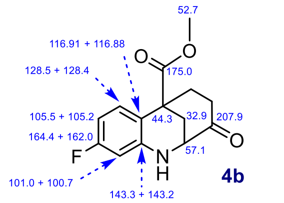

4b

22070326_B400FA_14072022_LRG237P4B.2.fid 13C{1H} 101 MHz
Equip: B400F / N.Inv: 1037597
N.Reg: 22070326
Usuari: san / Mostra: LRG237P4B
Nom: LAURA RODRIGUEZ GONZALEZ
Data: 14/07/2022 05:01:50 h./ Ope.: AUTOSERVEI
Experiment: A-C13-zgpg30 Solvent: CDCl3

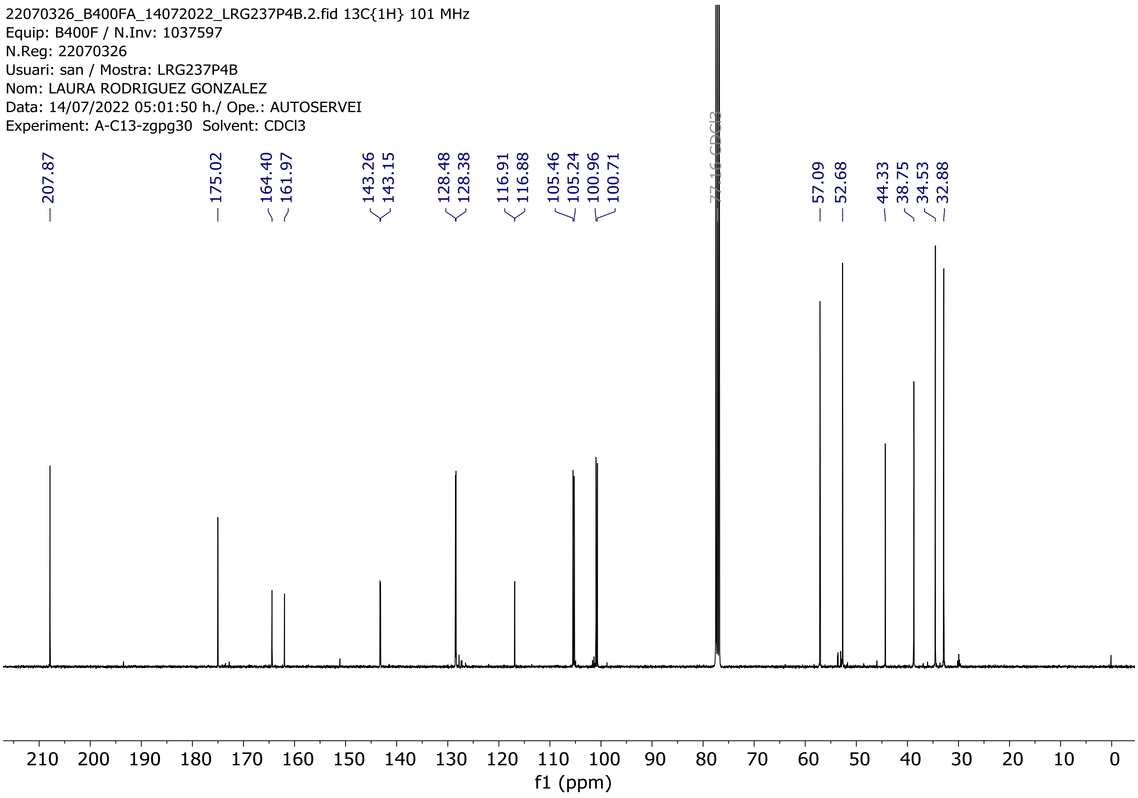

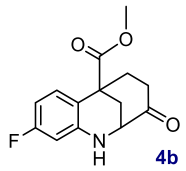

4b

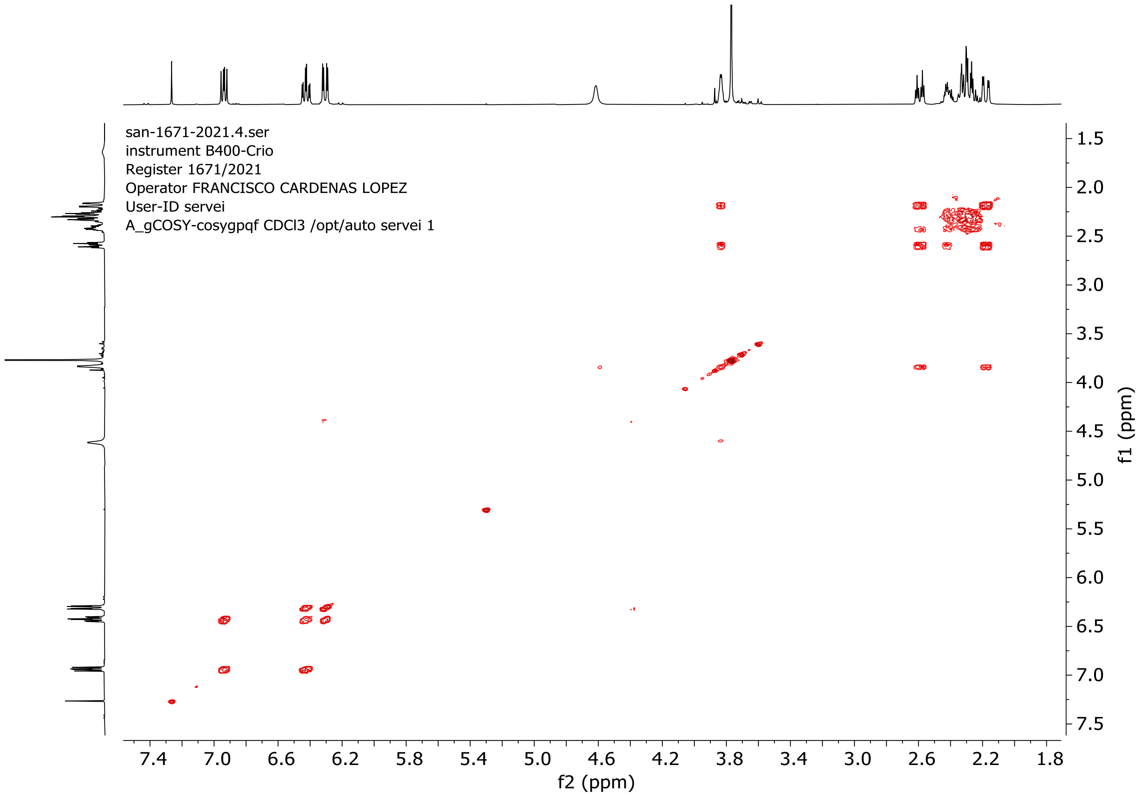
san-1671-2021.4.ser
instrument B400-Crio
Register 1671/2021
Operator FRANCISCO CARDENAS LOPEZ
User-ID servei
A_gCOSY-cosygpqf CDCl3 /opt/auto servei 1
f1 (ppm)
f2 (ppm)

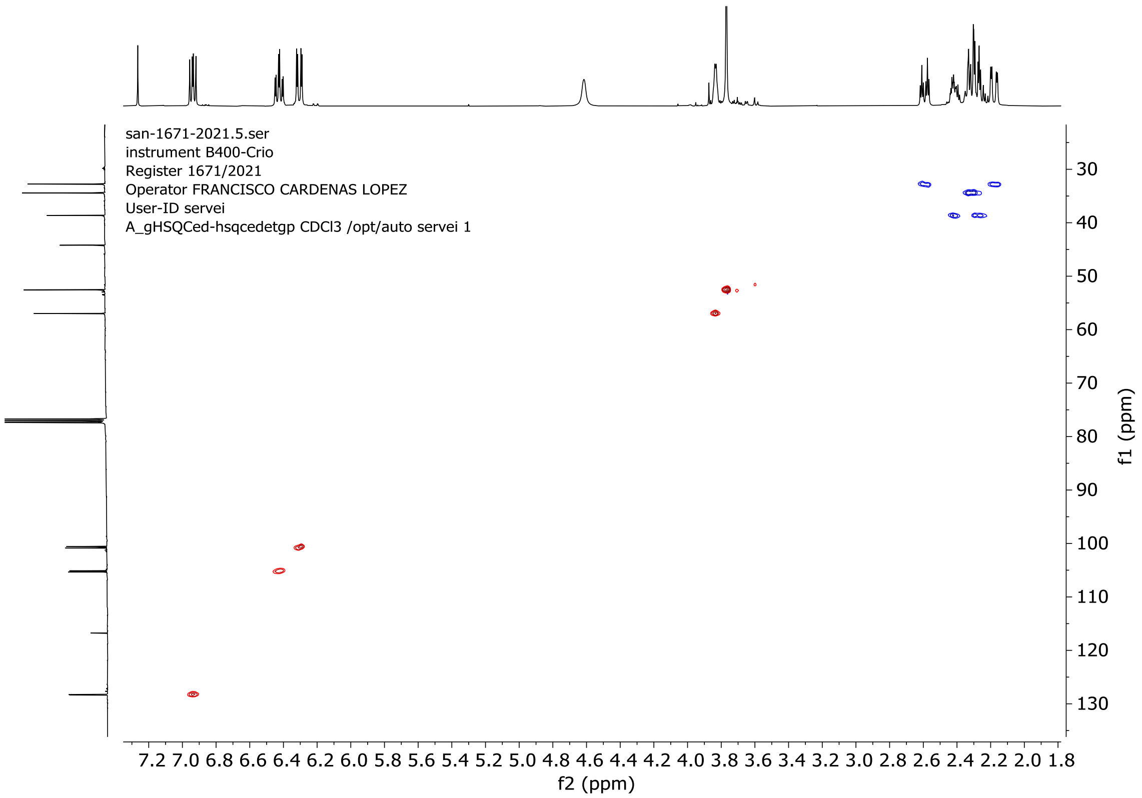
san-1671-2021.5.ser
instrument B400-Crio
Register 1671/2021
Operator FRANCISCO CARDENAS LOPEZ
User-ID servei
A_gHSQCed-hsqcedetgp CDCl3 /opt/auto servei 1
f1 (ppm)
f2 (ppm)

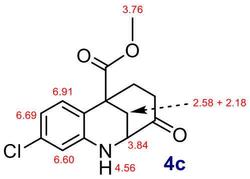

M400AQUI_22092021_LRG111dry-H1 400 MHz
M400Q / Num.Inv. AF/004285
cdcl3 / Temp: 25C / N.Reg: XXXXXXXXXX
Usuari: san / Mostra: LRG111dry
Nom: LAURA RODRIGUEZ GONZALEZ
Data: 22/09/21 / Ope.: L.RODRIGUEZ

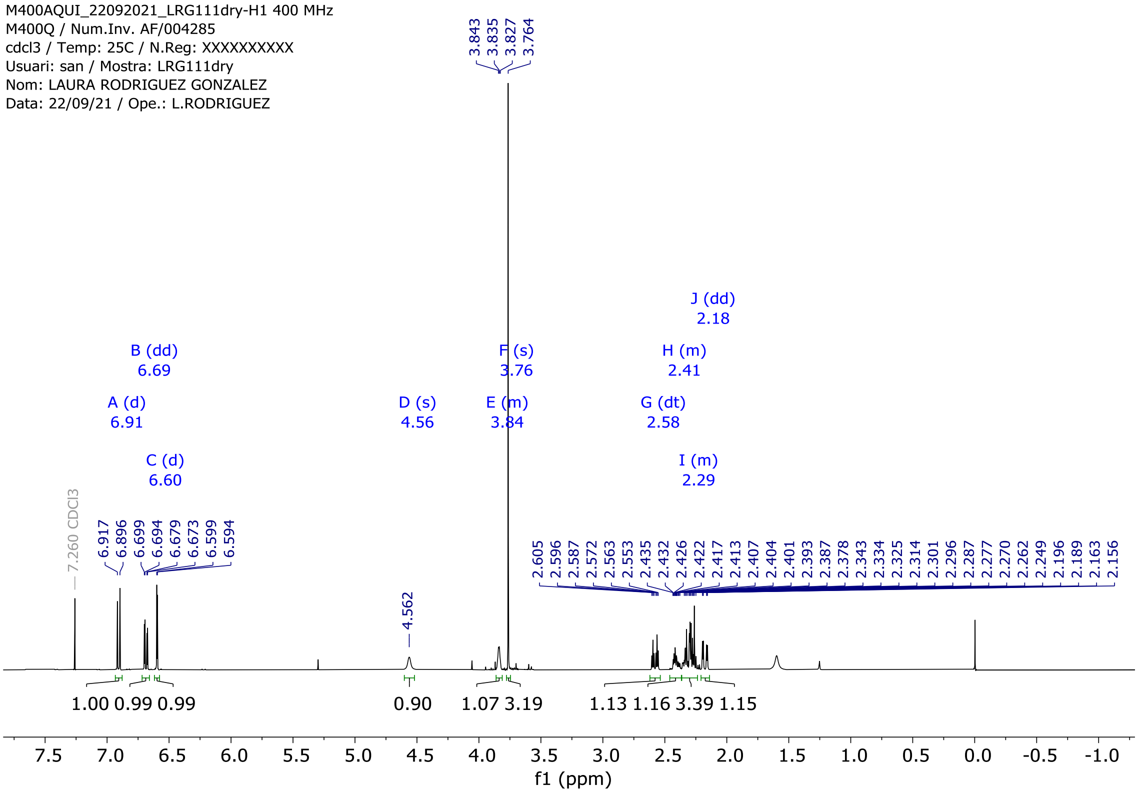

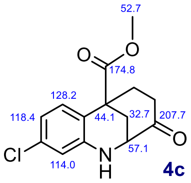

san-1725-2021.2.fid LRG111-13C{1H} 101 MHz
Equip: B400Q / N.Inv: 1009989
N.Reg:
Usuari: / Mostra:
Nom:
Data: 21/09/2021 16:13:33 h./ Ope.:

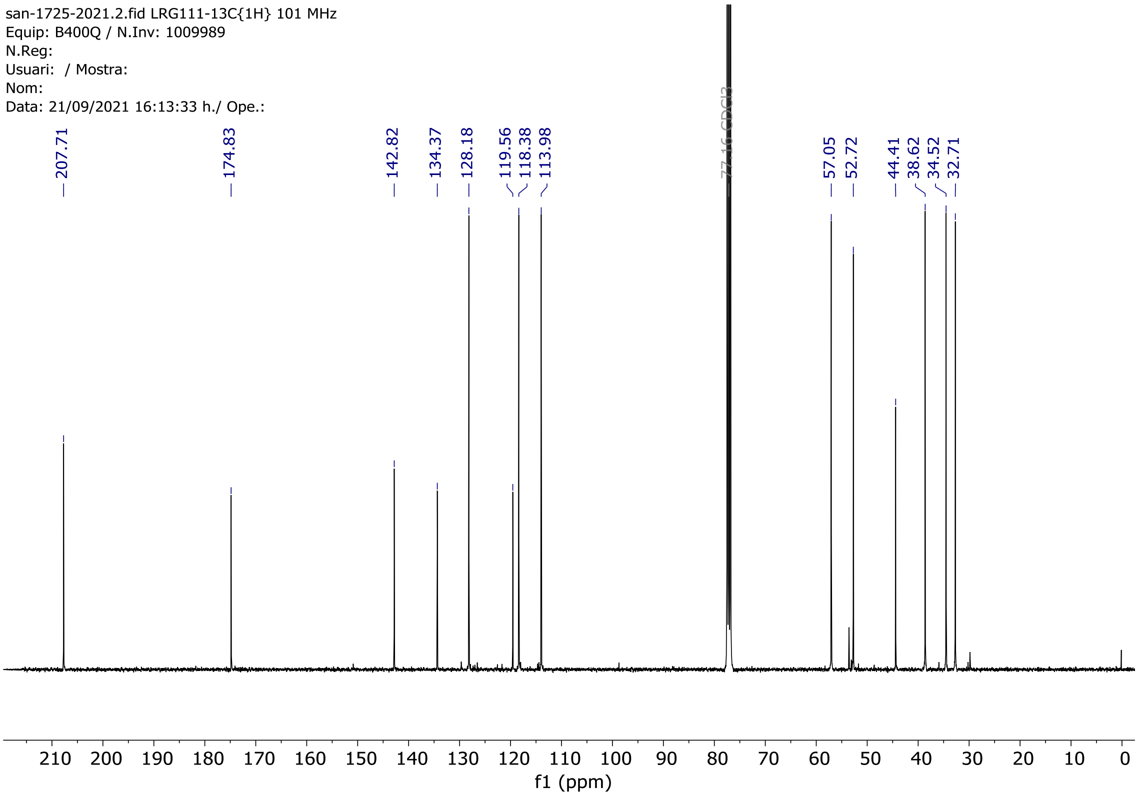

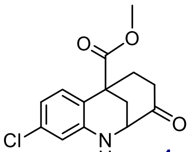
O
O
O
Cl
N
H

4c

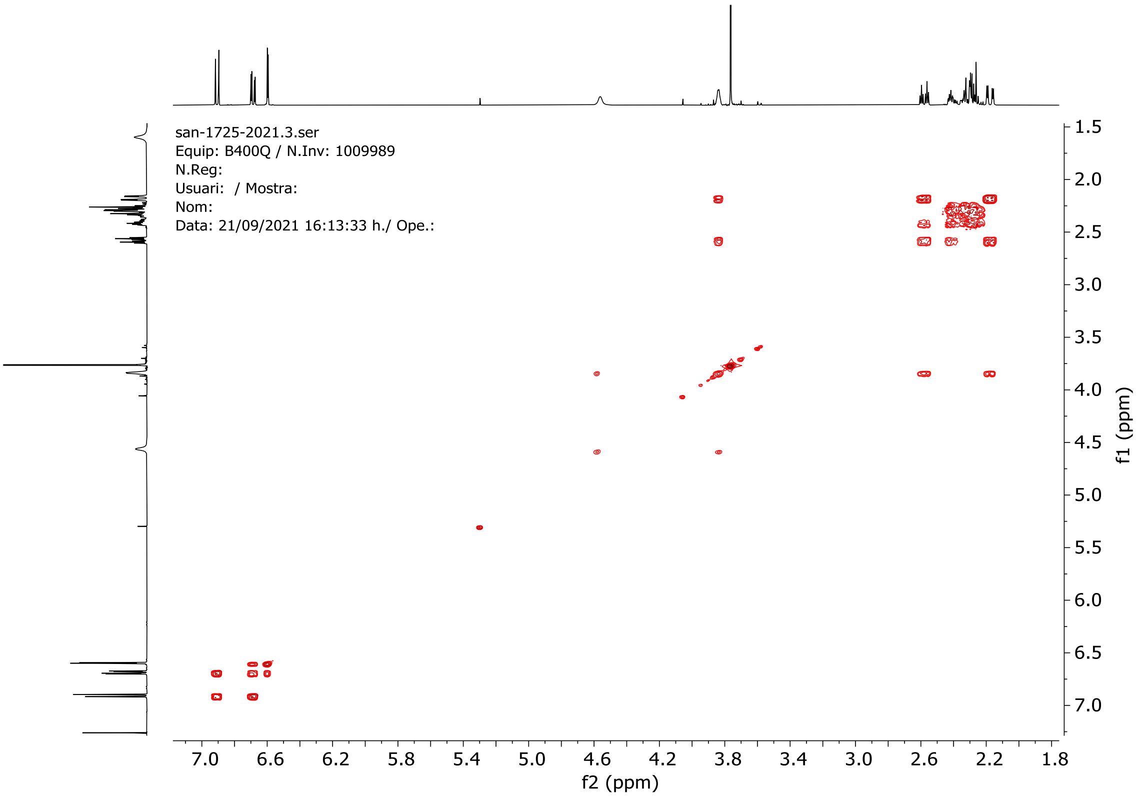
san-1725-2021.3.ser
Equip: B400Q / N.Inv: 1009989
N.Reg:
Usuari: / Mostra:
Nom:
Data: 21/09/2021 16:13:33 h./ Ope.:
f1 (ppm)
f2 (ppm)

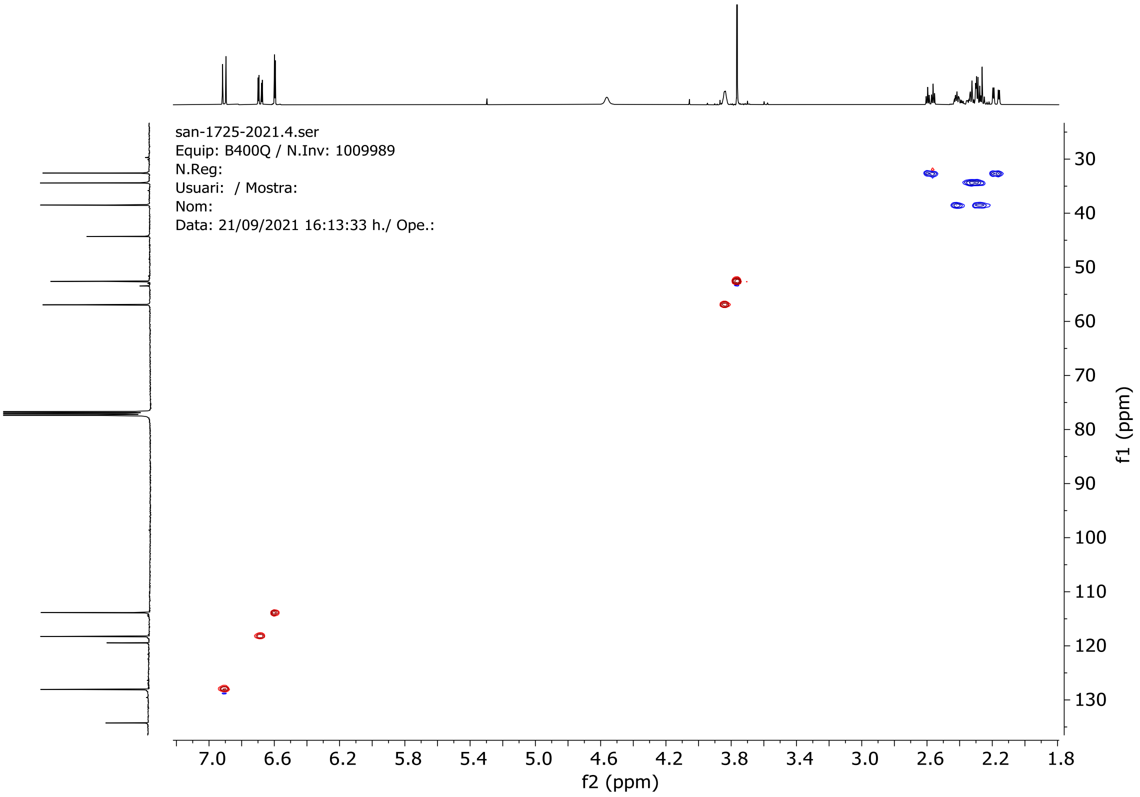
san-1725-2021.4.ser
Equip: B400Q / N.Inv: 1009989
N.Reg:
Usuari: / Mostra:
Nom:
Data: 21/09/2021 16:13:33 h./ Ope.:
f1 (ppm)
f2 (ppm)

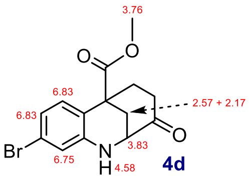

san-1474-2021.9.fid 400 MHz
Equip: B400Q / N.Inv: 1009989
N.Reg: 1474/2021
Usuari: san / Mostra: LRG092ch
Nom: LAURA RODRIGUEZ GONZALEZ
Data: 22/07/2021 14:54:54 h./ Ope.: Victor Meriel

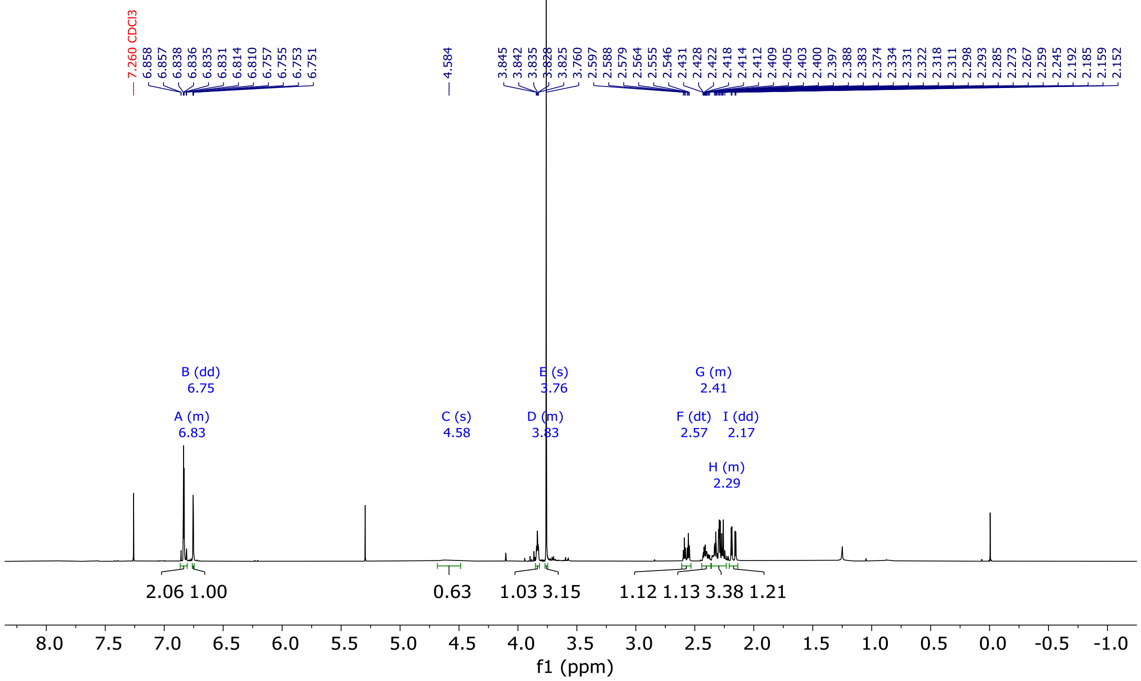

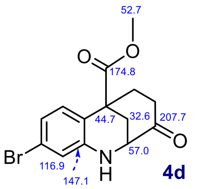

san-1474-2021.10.fid LRG092-13C{1H} 101 MHz
Equip: B400Q / N.Inv: 1009989
N.Reg:
Usuari: / Mostra:
Nom:
Data: 22/07/2021 17:54:40 h./ Ope.:

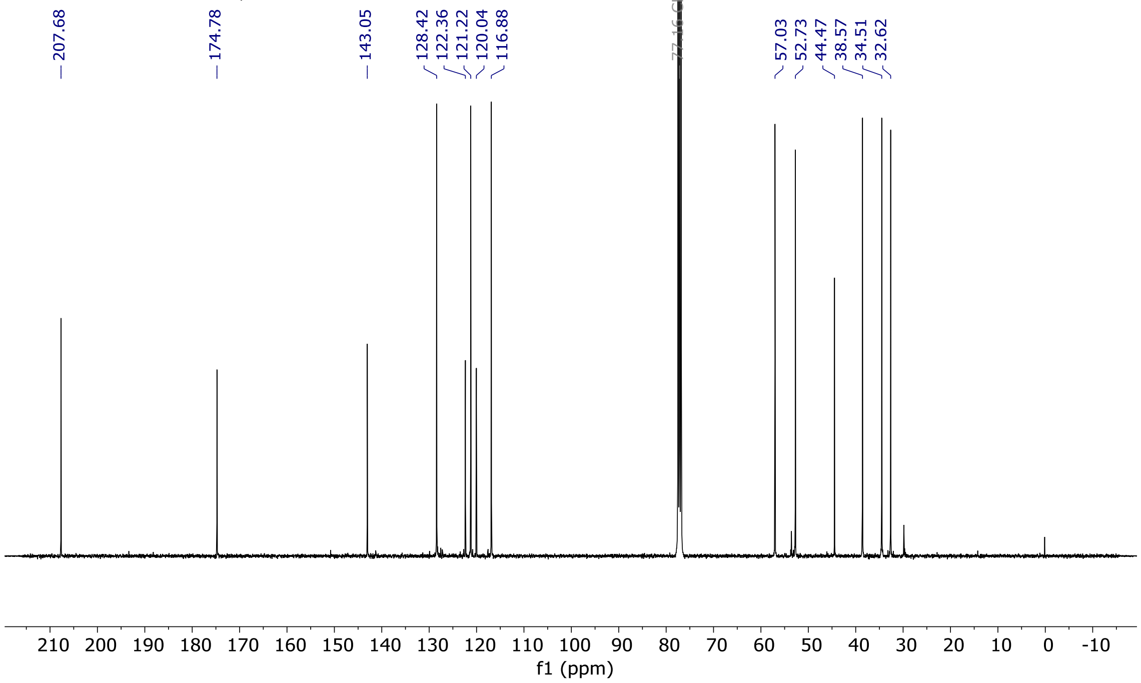

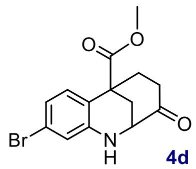
Br
N
H
O
O
O
4d

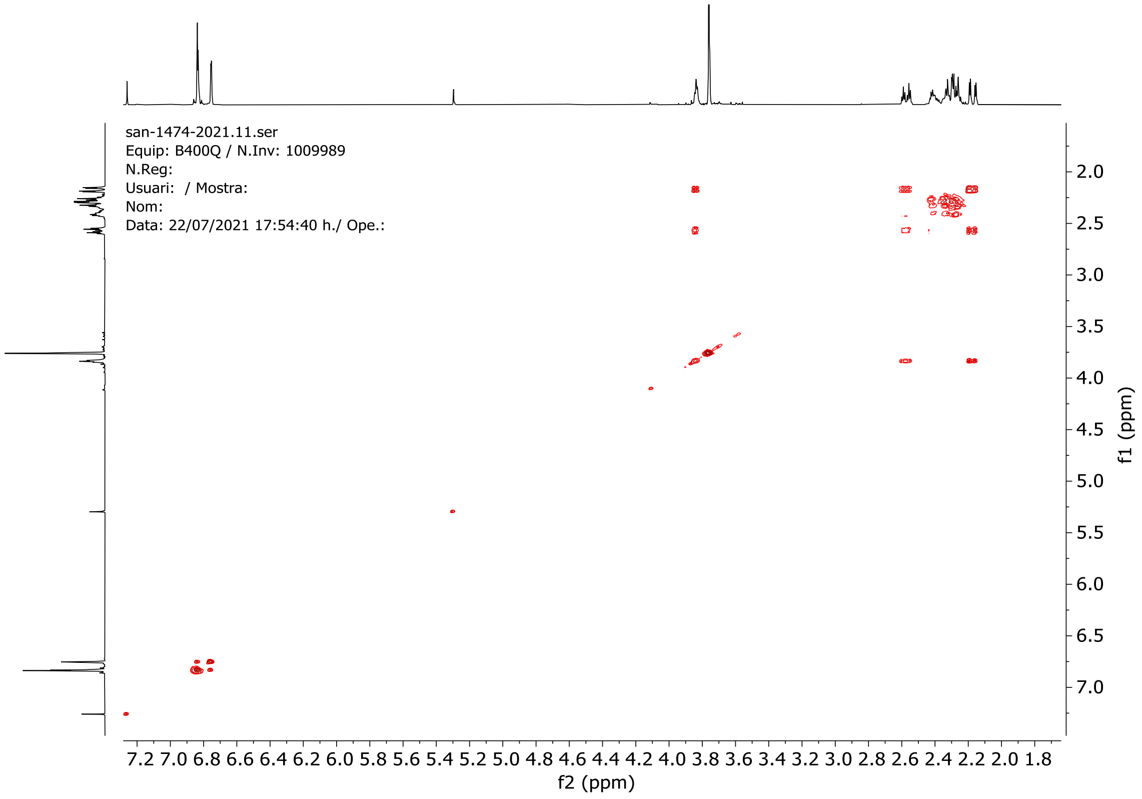
san-1474-2021.11.ser
Equip: B400Q / N.Inv: 1009989
N.Reg:
Usuari: / Mostra:
Nom:
Data: 22/07/2021 17:54:40 h./ Ope.:
f2 (ppm)
f1 (ppm)

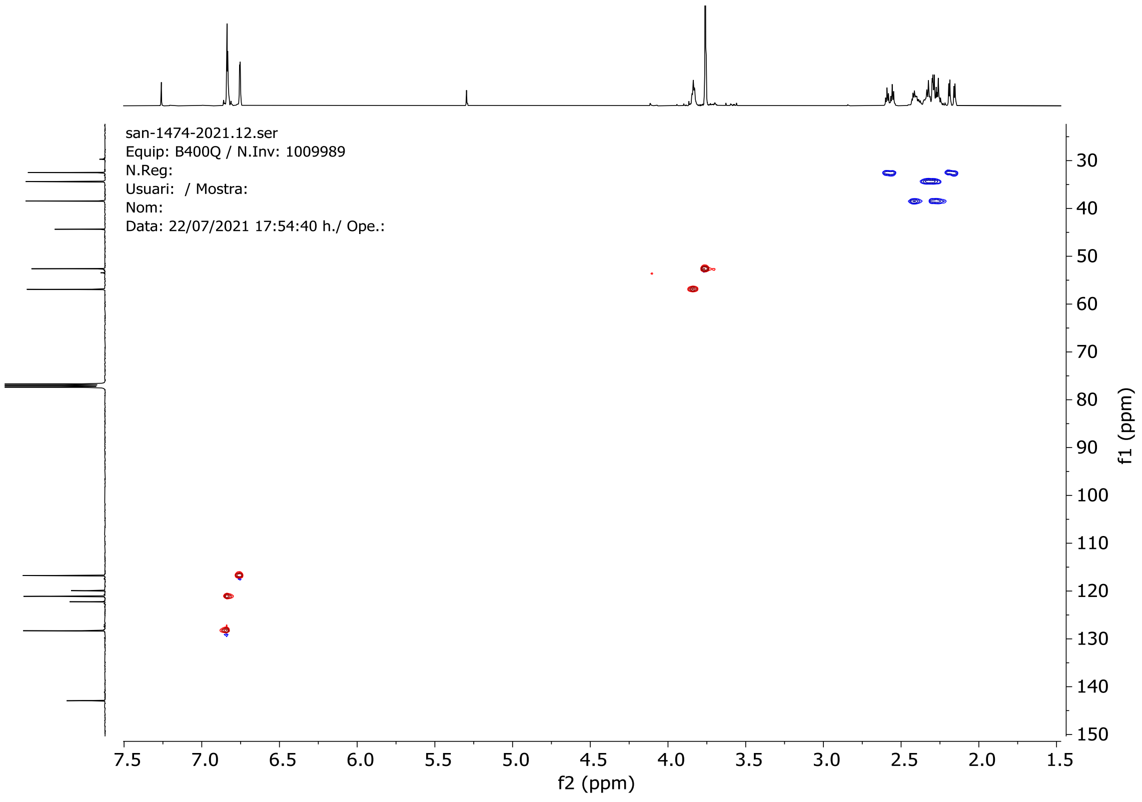
san-1474-2021.12.ser
Equip: B400Q / N.Inv: 1009989
N.Reg:
Usuari: / Mostra:
Nom:
Data: 22/07/2021 17:54:40 h./ Ope.:
f2 (ppm)
f1 (ppm)

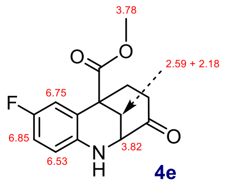

4e

M400AQUI_15072021_LRG089CH-H1 400 MHz
M400Q / Num.Inv. AF/004285
cdcl3 / Temp: 25C / N.Reg: XXXXXXXXXX
Usuari: san / Mostra: LRG089CH
Nom: LAURA RODRIGUEZ GONZALEZ
Data: 15/07/21 / Ope.: L.RODRIGUEZ

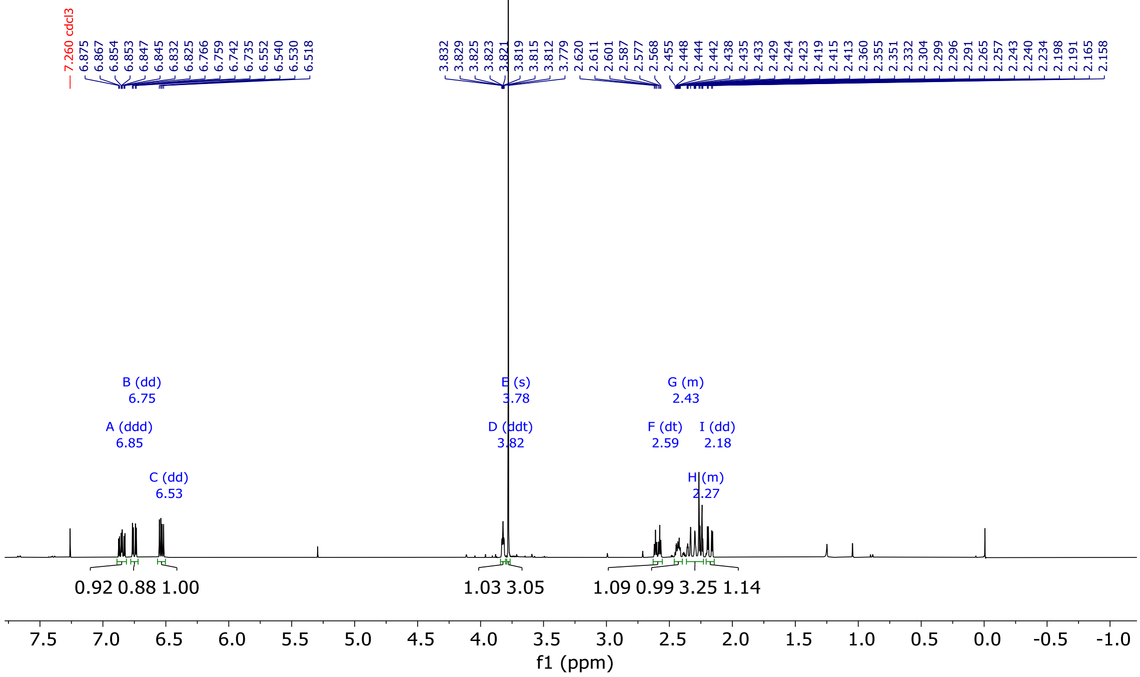

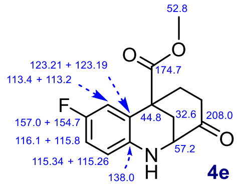

4e

M400AQUI_15072021_LRG089CH-C13{1H} 101 MHz
M400Q / Num.Inv. AF/004285
cdcl3 / Temp: 25C / N.Reg: XXXXXXXXXX
Usuari: san / Mostra: LRG089CH
Nom: LAURA RODRIGUEZ GONZALEZ
Data: 15/07/21 / Ope.: L.RODRIGUEZ

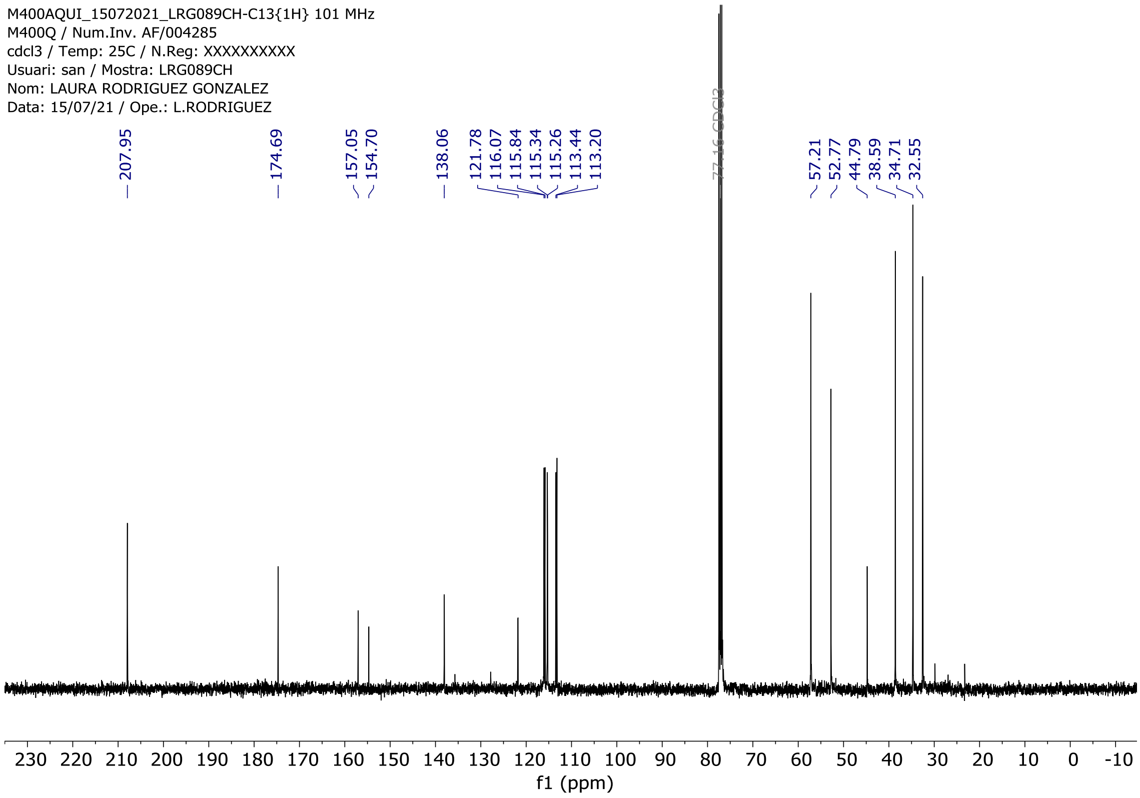

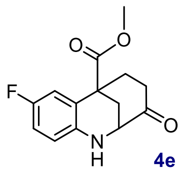
F
O
O
N
H
O

4e

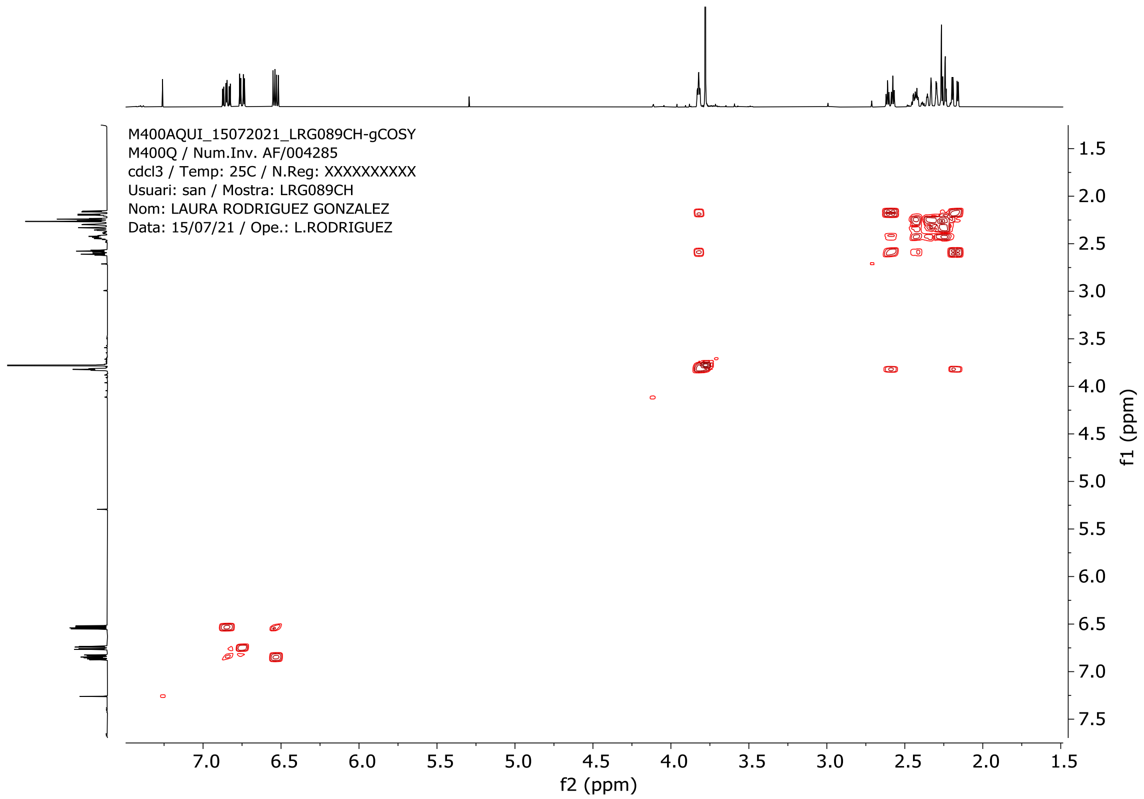
M400AQUI_15072021_LRG089CH-gCOSY
M400Q / Num.Inv. AF/004285
cdcl3 / Temp: 25C / N.Reg: XXXXXXXXXX
Usuari: san / Mostra: LRG089CH
Nom: LAURA RODRIGUEZ GONZALEZ
Data: 15/07/21 / Ope.: L.RODRIGUEZ
f2 (ppm)
f1 (ppm)

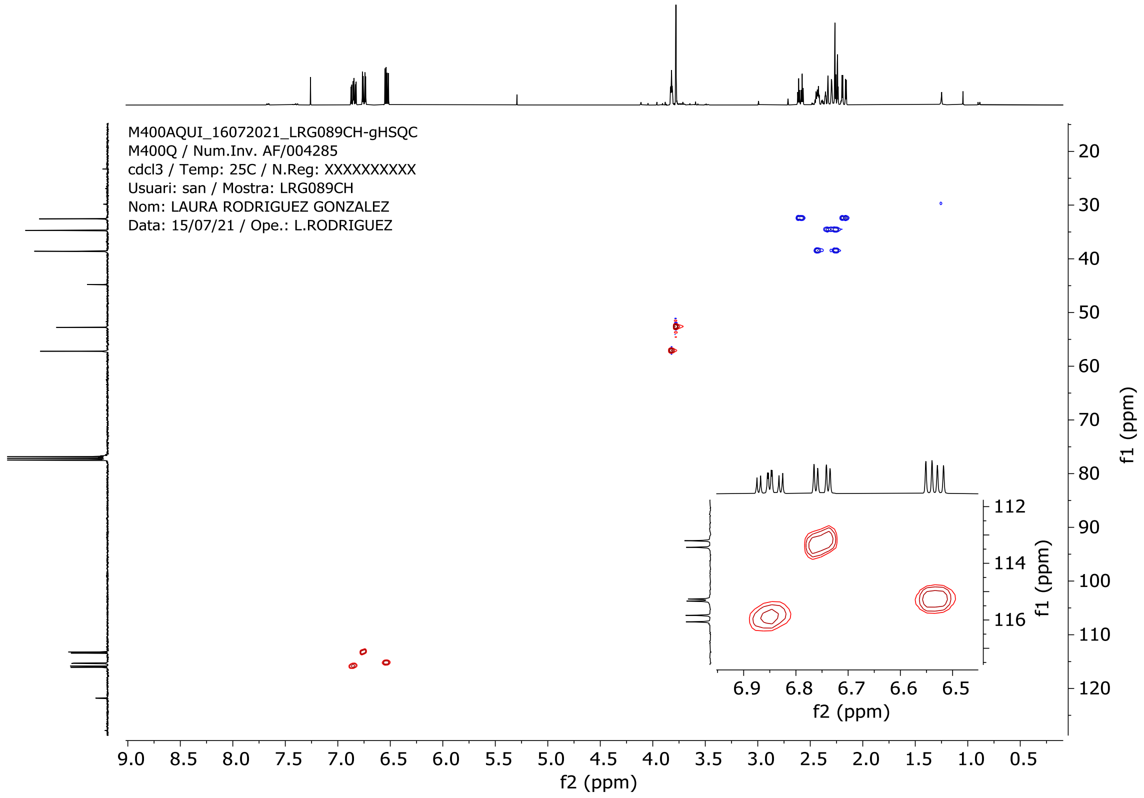
M400AQUI_16072021_LRG089CH-gHSQC
M400Q / Num.Inv. AF/004285
cdcl3 / Temp: 25C / N.Reg: XXXXXXXXXX
Usuari: san / Mostra: LRG089CH
Nom: LAURA RODRIGUEZ GONZALEZ
Data: 15/07/21 / Ope.: L.RODRIGUEZ
f2 (ppm)
f1 (ppm)

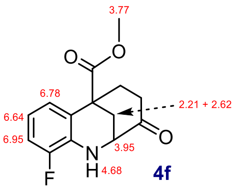

M400AQUI_22092021_LRG113ch-H1 400 MHz
M400Q / Num.Inv. AF/004285
cdcl3 / Temp: 25C / N.Reg: XXXXXXXXXX
Usuari: san / Mostra: LRG113ch
Nom: LAURA RODRIGUEZ GONZALEZ
Data: 22/09/21 / Ope.: L.RODRIGUEZ

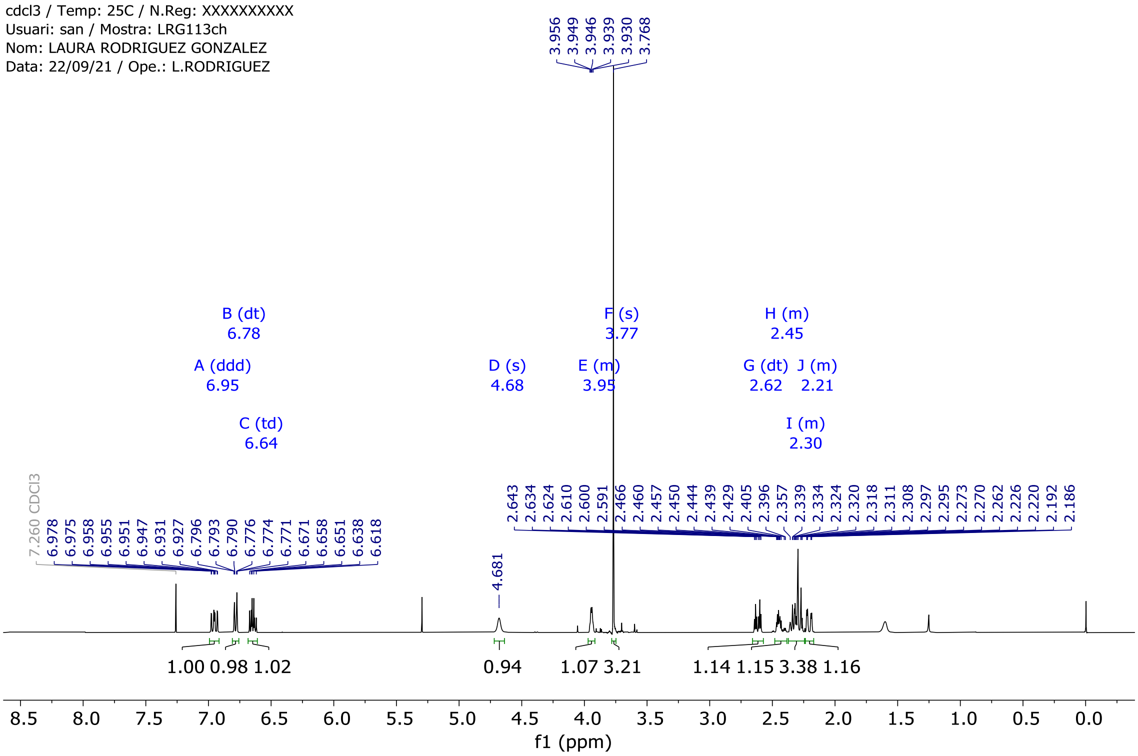

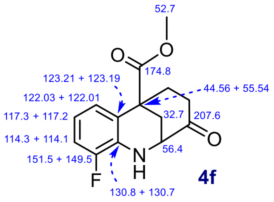

1732-2021_B500Q_23092021_LRG113ch.2.fid 13C{1H} 126 MHz
Equip: B500Q / N.Inv: 1028917
N.Reg:
Usuari: / Mostra:
Nom:
Data: 22/09/2021 17:18:51 h./ Ope.:

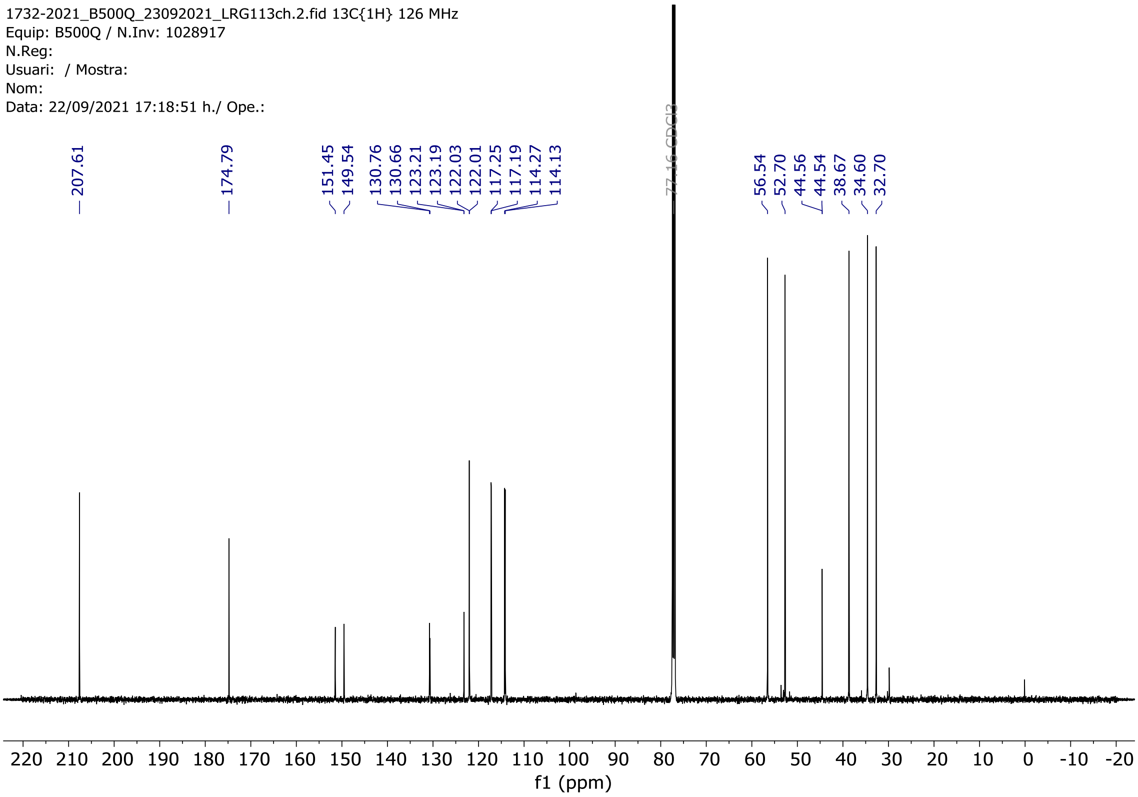

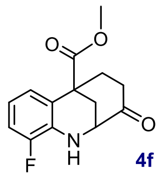

4f

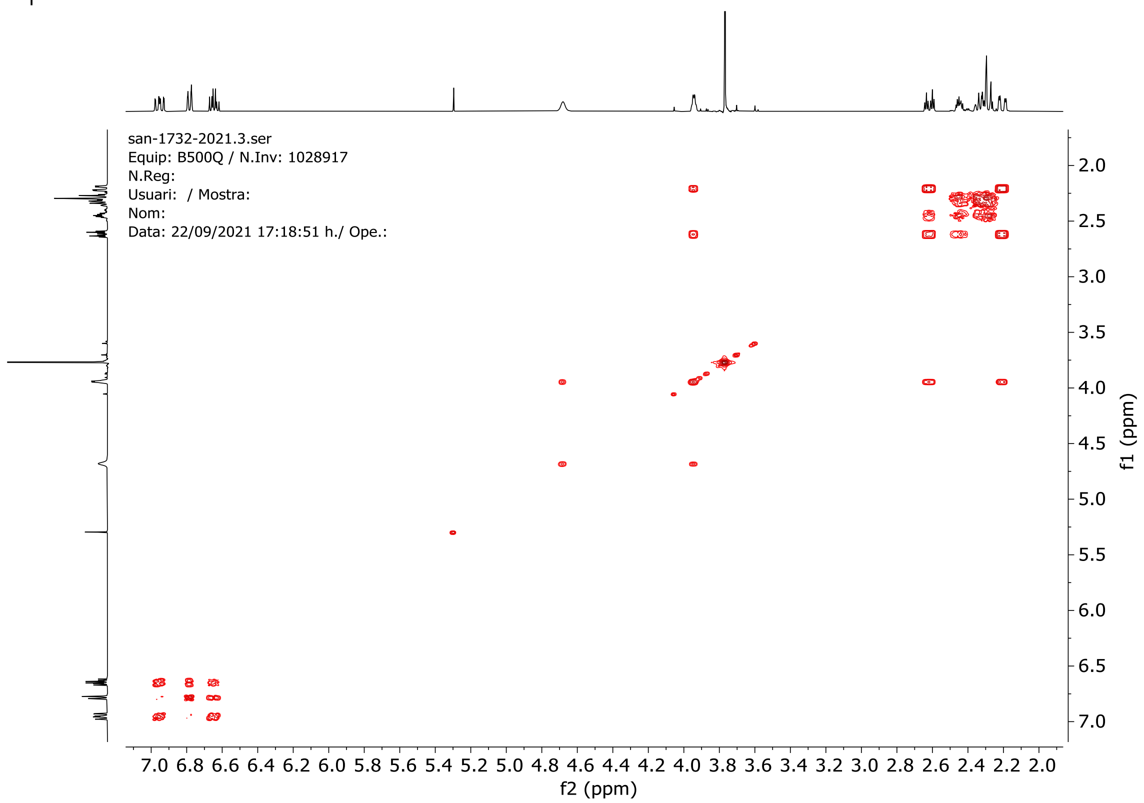

san-1732-2021.3.ser
Equip: B500Q / N.Inv: 1028917
N.Reg:
Usuari: / Mostra:
Nom:
Data: 22/09/2021 17:18:51 h./ Ope.:
f2 (ppm)
f1 (ppm)

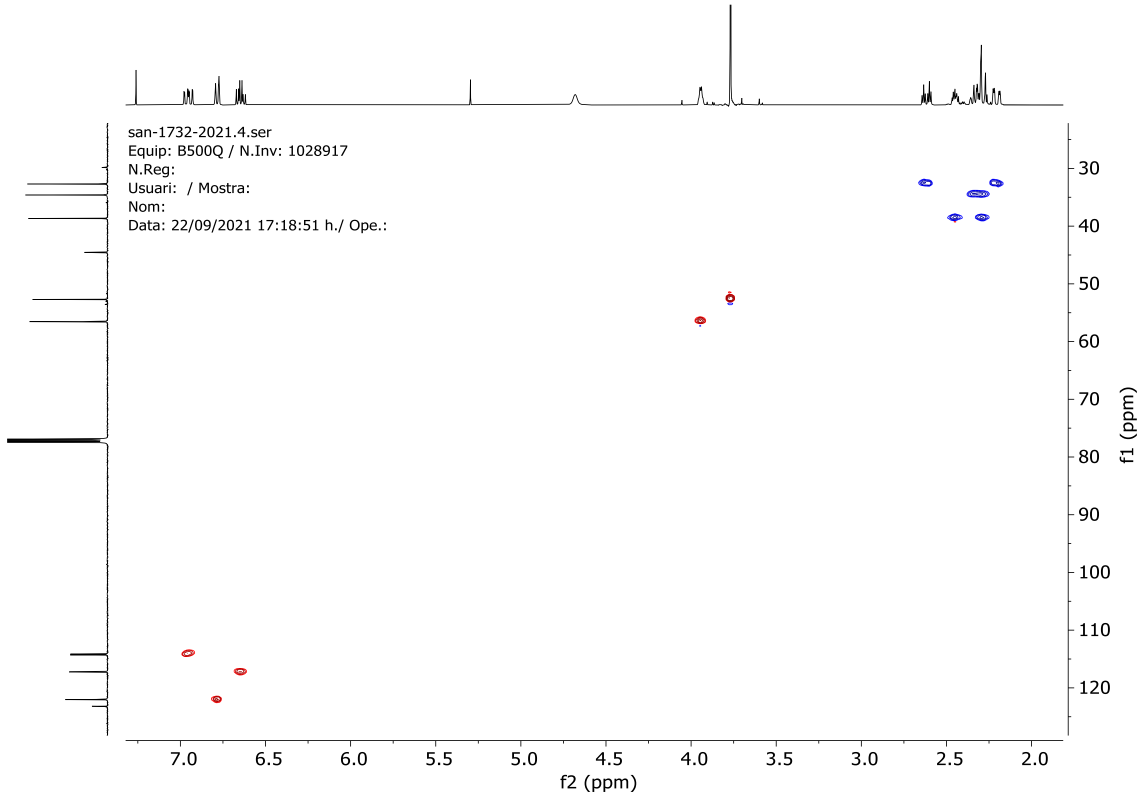

san-1732-2021.4.ser
Equip: B500Q / N.Inv: 1028917
N.Reg:
Usuari: / Mostra:
Nom:
Data: 22/09/2021 17:18:51 h./ Ope.:
f2 (ppm)
f1 (ppm)

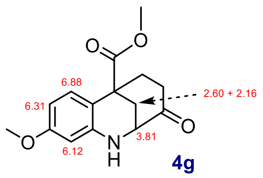

4g

M400AQUI_05102021_LRG118DRY-H1 400 MHz
M400Q / Num.Inv. AF/004285
cdcl3 / Temp: 25C / N.Reg: XXXXXXXXXX
Usuari: san / Mostra: LRG118DRY
Nom: LAURA RODRIGUEZ GONZALEZ
Data: 05/10/21 / Ope.: L.RODRIGUEZ

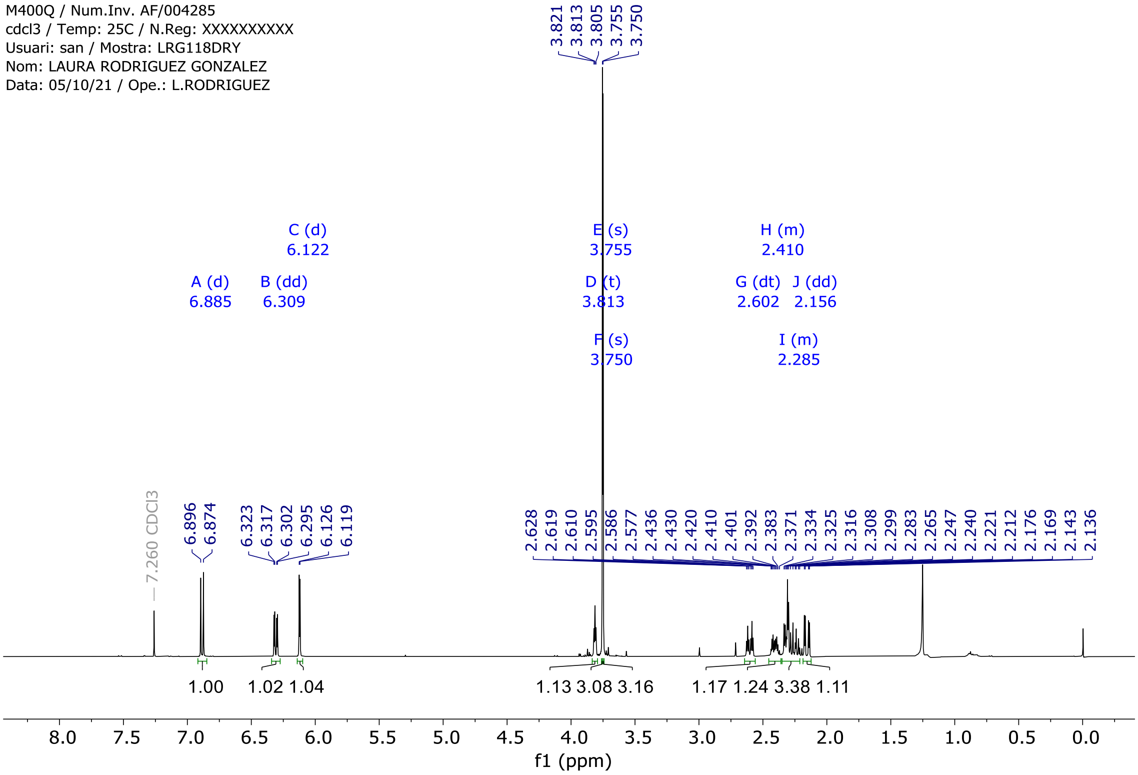

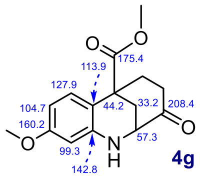

4g

1782-2021_B500Q_04102021_LRG118ch.2.fid 13C{1H} 126 MHz
Register 1782/2021
Operator AGUSTIN MARTINEZ

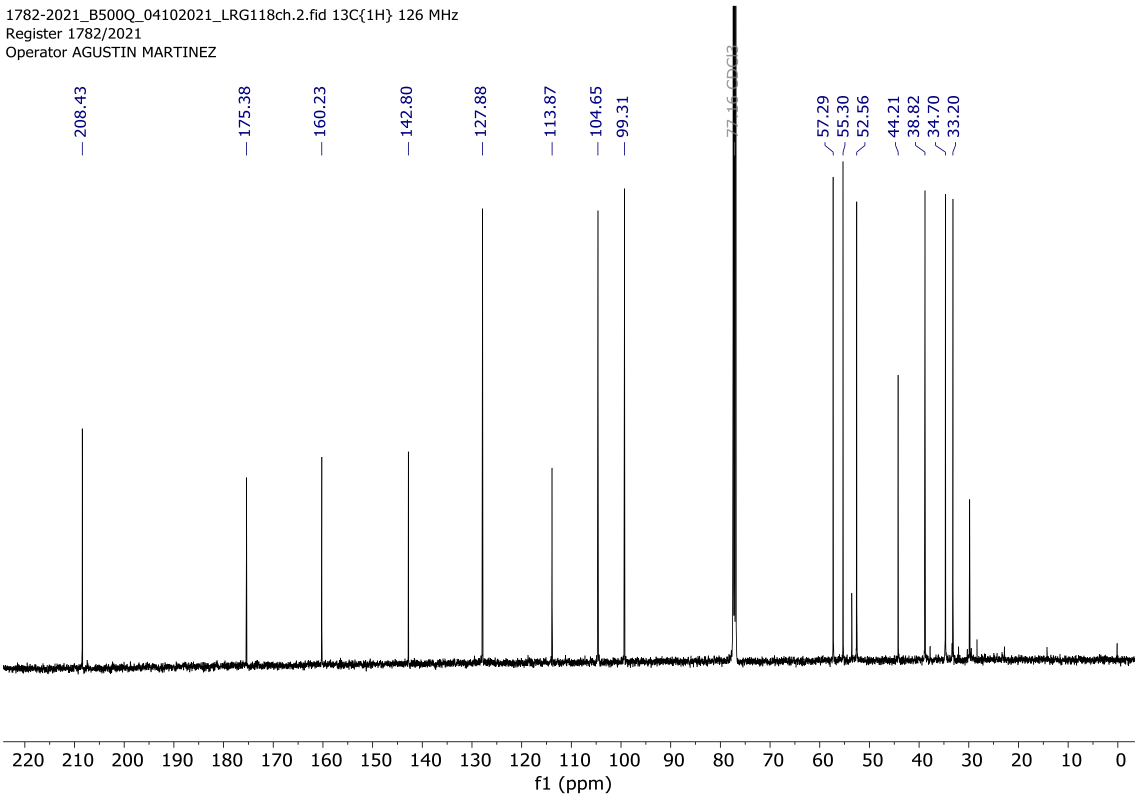

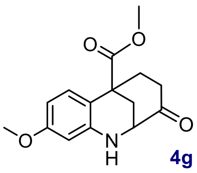

4g

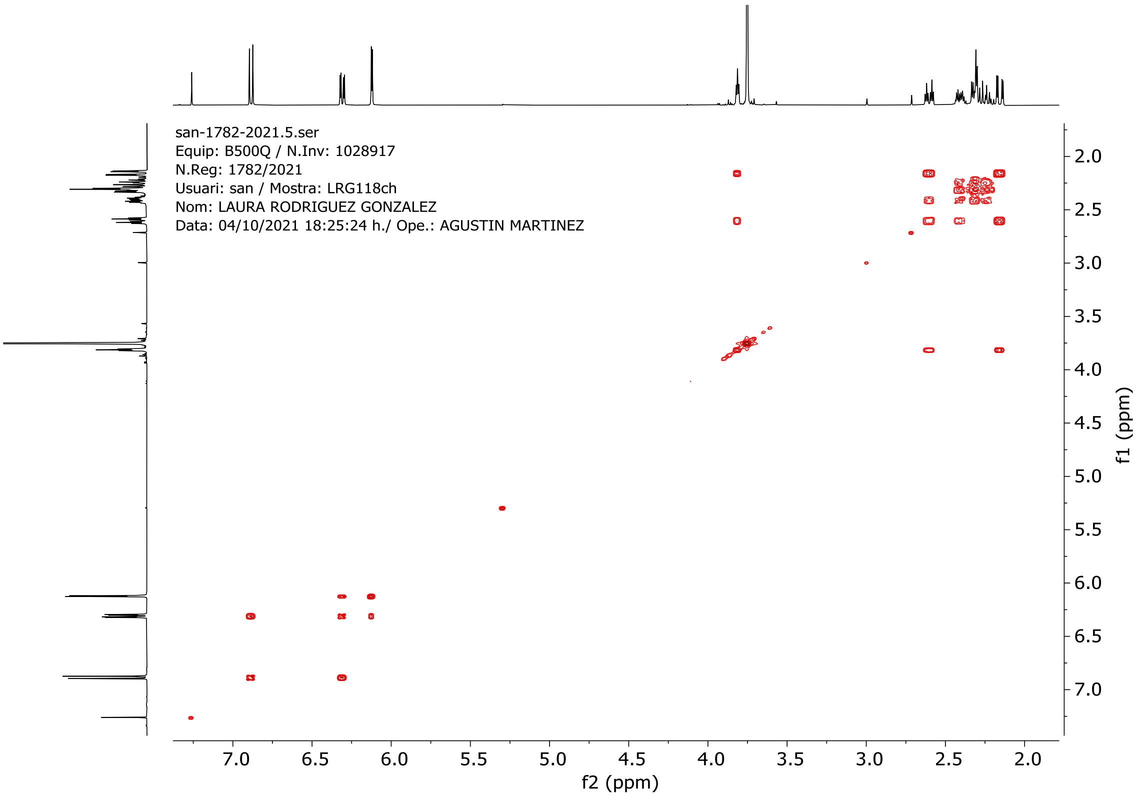

san-1782-2021.5.ser
Equip: B500Q / N.Inv: 1028917
N.Reg: 1782/2021
Usuari: san / Mostra: LRG118ch
Nom: LAURA RODRIGUEZ GONZALEZ
Data: 04/10/2021 18:25:24 h./ Ope.: AGUSTIN MARTINEZ
f2 (ppm)
f1 (ppm)

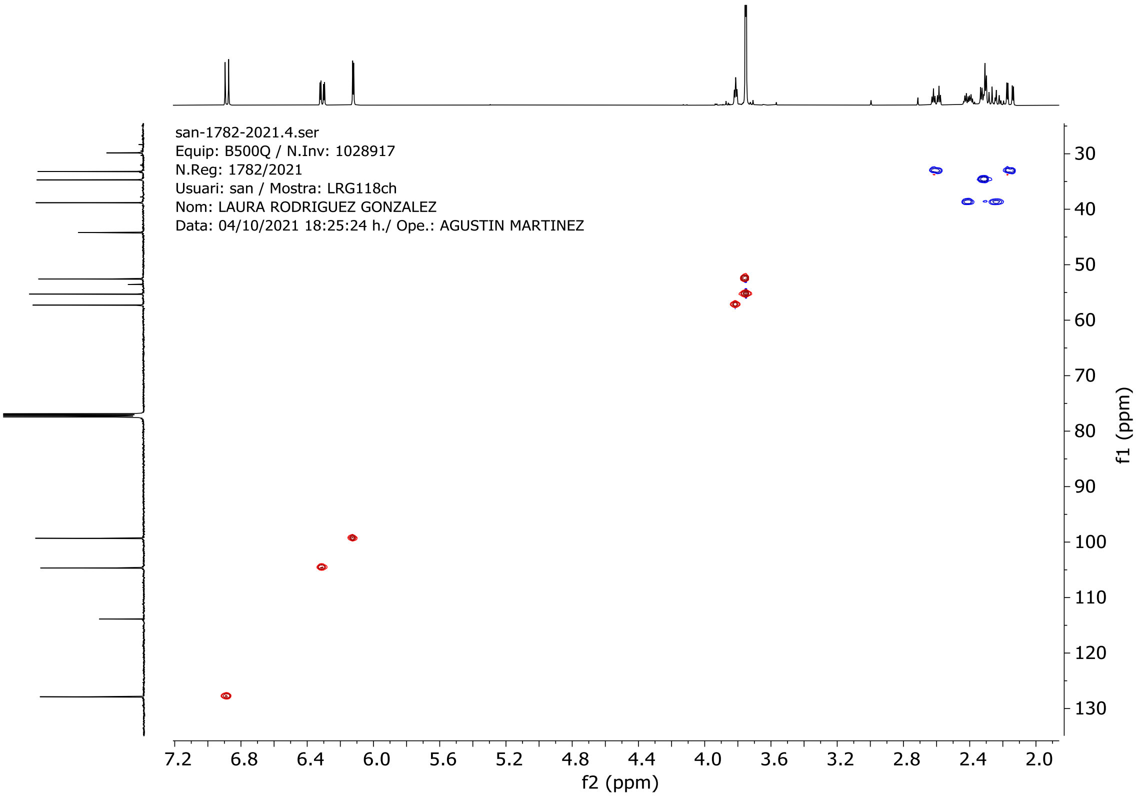

san-1782-2021.4.ser
Equip: B500Q / N.Inv: 1028917
N.Reg: 1782/2021
Usuari: san / Mostra: LRG118ch
Nom: LAURA RODRIGUEZ GONZALEZ
Data: 04/10/2021 18:25:24 h./ Ope.: AGUSTIN MARTINEZ
f2 (ppm)
f1 (ppm)

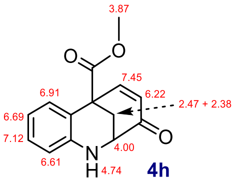

M400AQUI_19072021_LRG091-H1 400 MHz
M400Q / Num.Inv. AF/004285
cdcl3 / Temp: 25C / N.Reg: XXXXXXXXXX
Usuari: san / Mostra: LRG091
Nom: LAURA RODRIGUEZ GONZALEZ
Data: 19/07/21 / Ope.: L.RODRIGUEZ

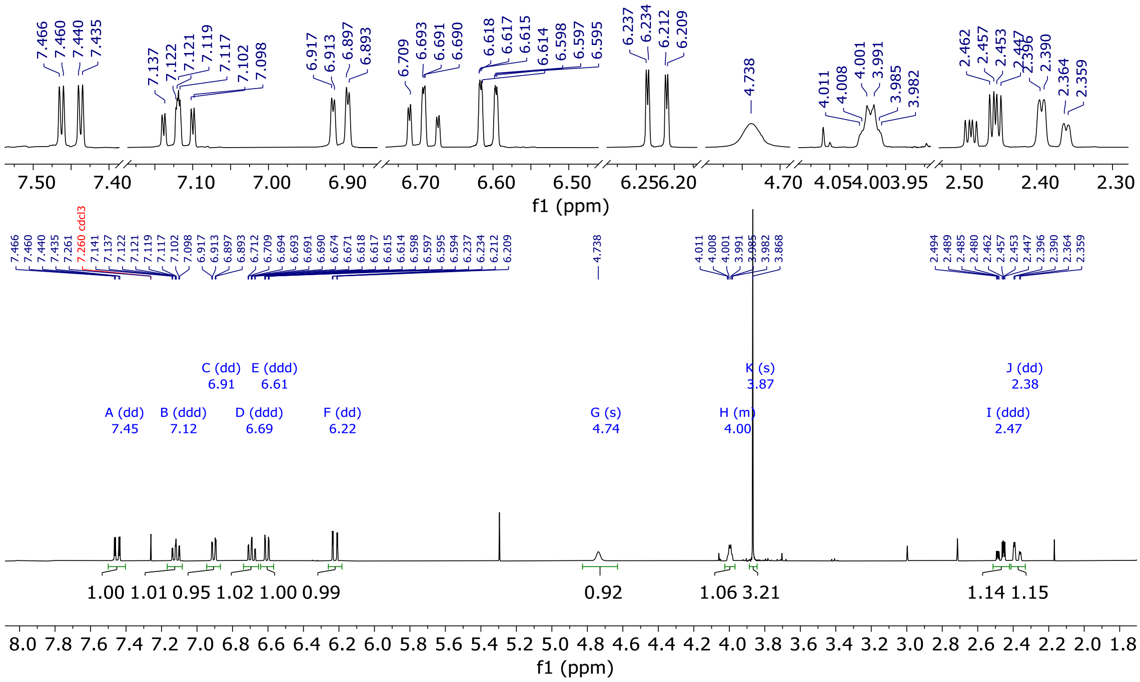

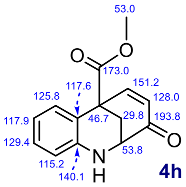

san-1455-2021.2.fid LRG091-13C{1H} 101 MHz
Equip: B400Q / N.Inv: 1009989
N.Reg:
Usuari: / Mostra:
Nom:
Data: 19/07/2021 18:26:42 h./ Ope.:

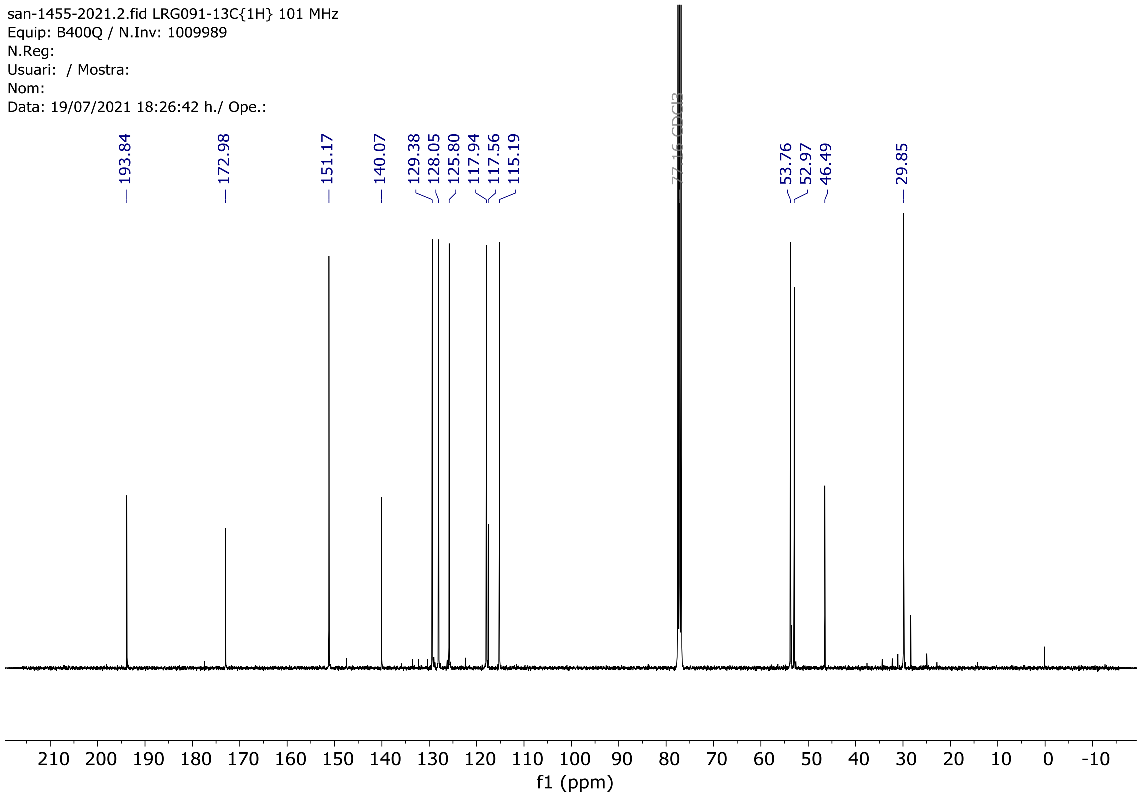

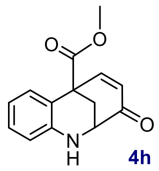

4h

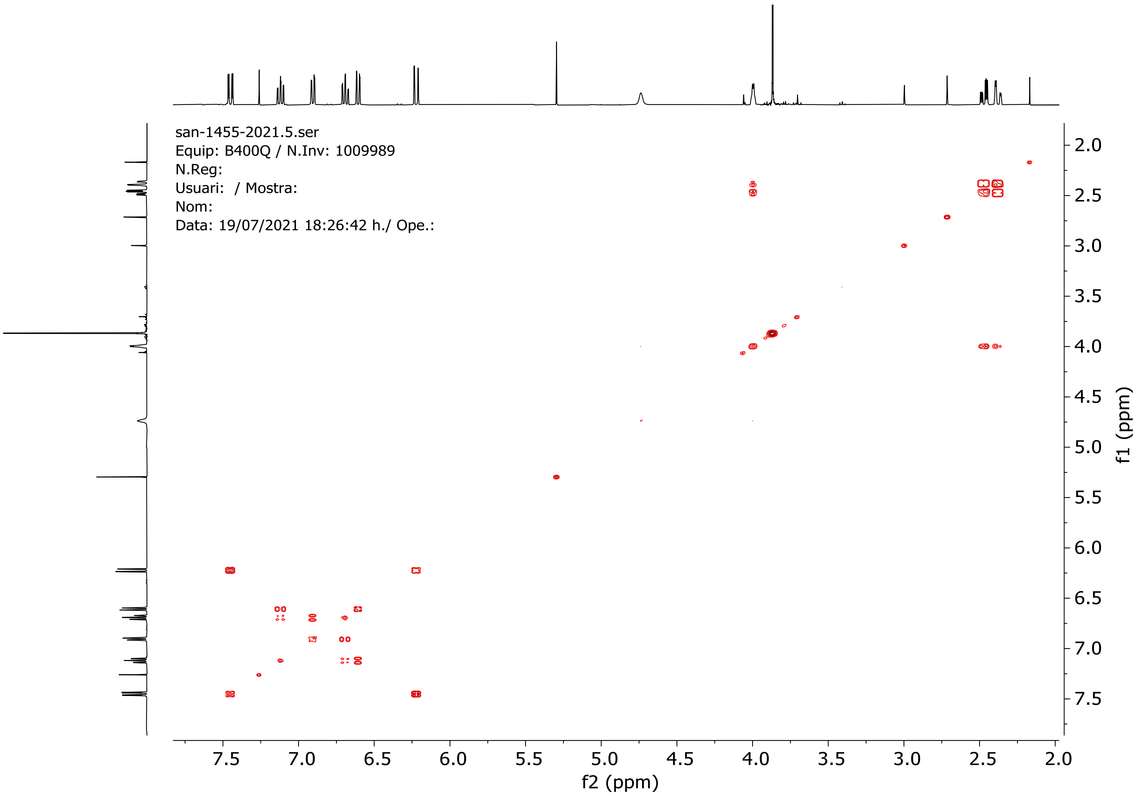
san-1455-2021.5.ser
Equip: B400Q / N.Inv: 1009989
N.Reg:
Usuari: / Mostra:
Nom:
Data: 19/07/2021 18:26:42 h./ Ope.:
f1 (ppm)
f2 (ppm)

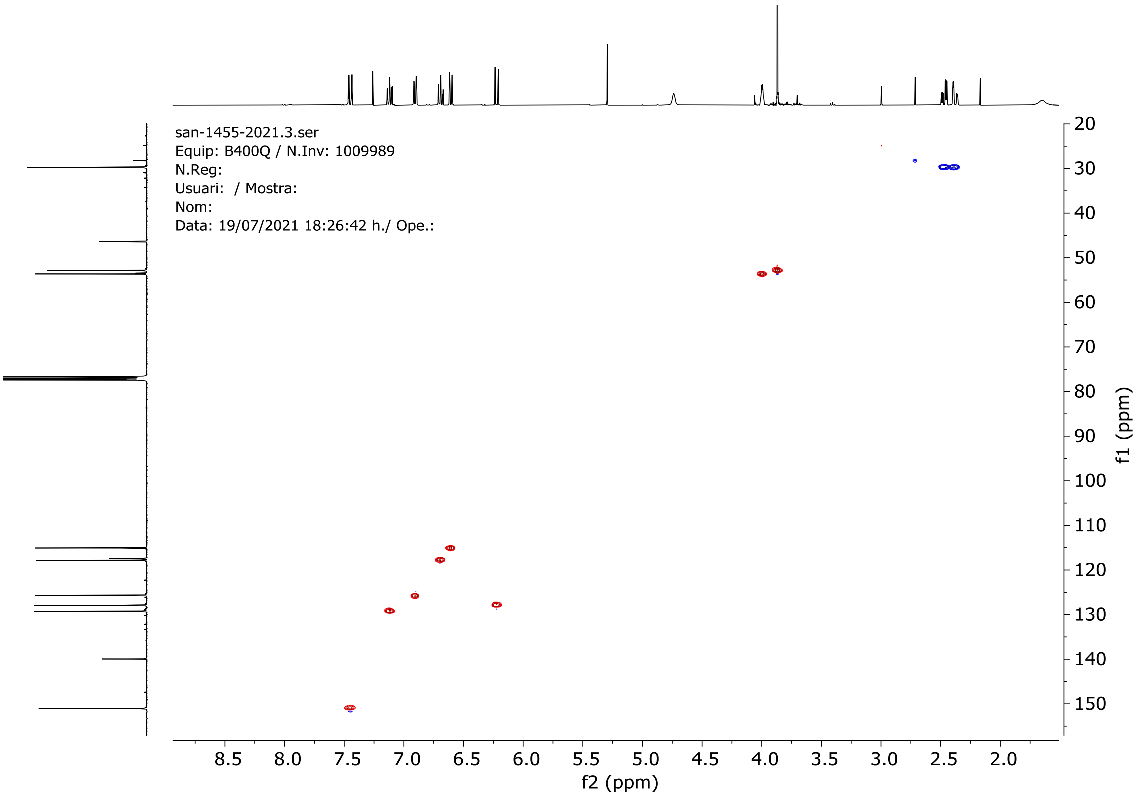
san-1455-2021.3.ser
Equip: B400Q / N.Inv: 1009989
N.Reg:
Usuari: / Mostra:
Nom:
Data: 19/07/2021 18:26:42 h./ Ope.:
f1 (ppm)
f2 (ppm)

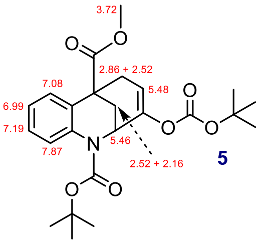

5

22070586_B400FA_20072022_LRG242.1.fid 400 MHz
Equip: B400F / N.Inv: 1037597
N.Reg: 22070586
Usuari: san / Mostra: LRG242
Nom: LAURA RODRIGUEZ GONZALEZ
Data: 20/07/2022 16:28:31 h./ Ope.: AUTOSERVEI
Experiment: A-H1-zg30 Solvent: CDCl3

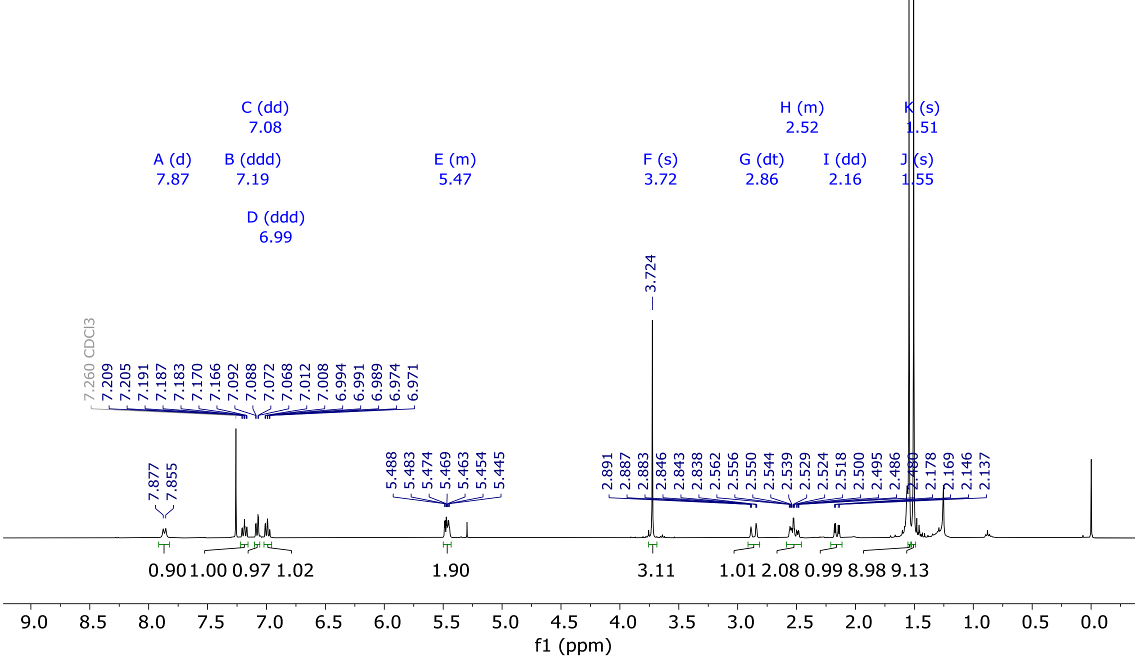

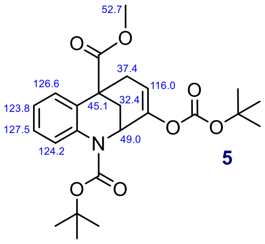

5

9755-2022_B400FA_21072022_LRG242CH.2.fid 13C{1H} 101 MHz
Equip: B400F / N.Inv: 1037597
N.Reg: 9755/2022
Usuari: san / Mostra: LRG242CH
Nom: LAURA RODRIGUEZ GONZALEZ
Data: 21/07/2022 21:27:21 h./ Ope.: servei Unitat RMN
Experiment: A-C13-zgpg30 Solvent: CDCl3

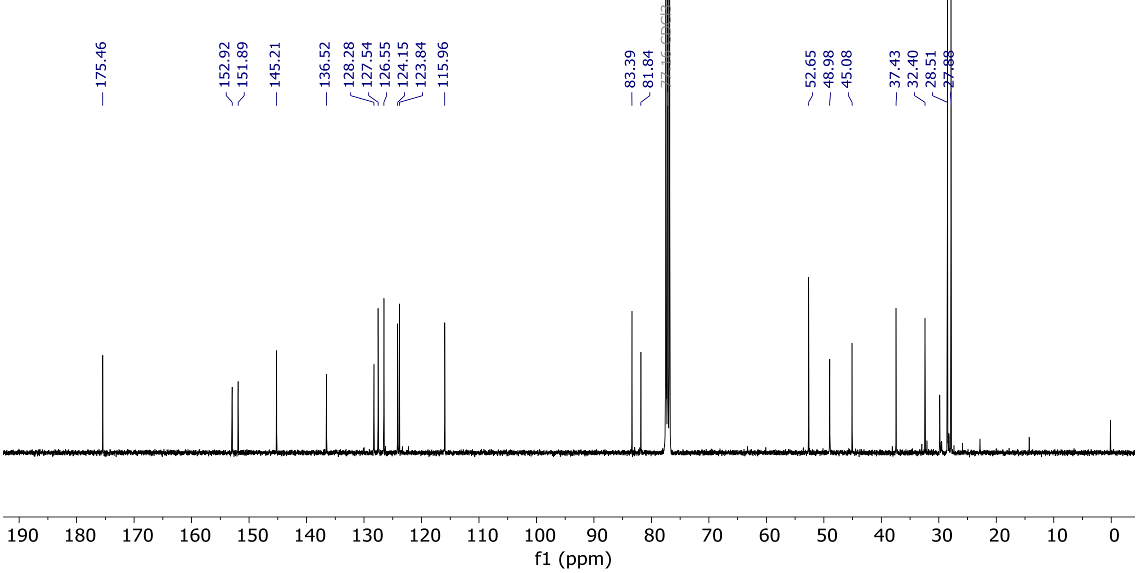

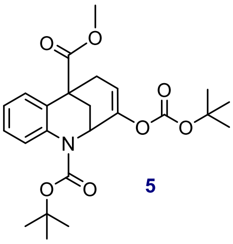

5

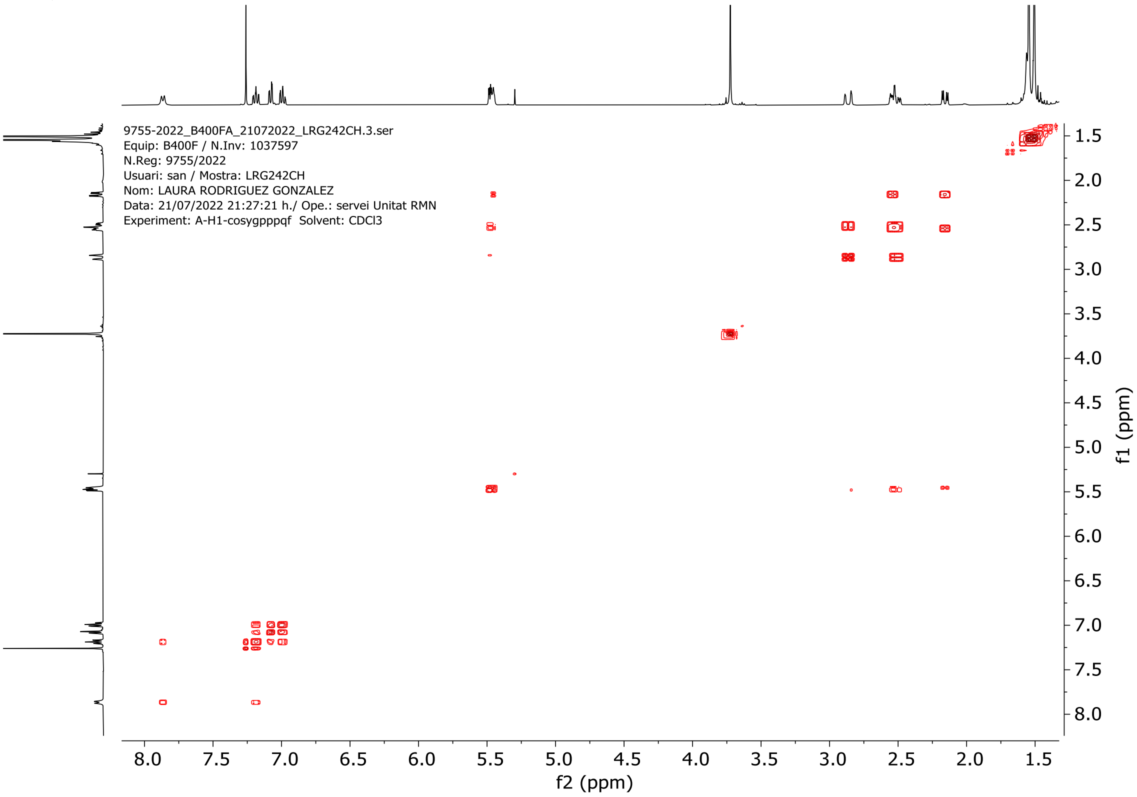

9755-2022_B400FA_21072022_LRG242CH.3.ser
Equip: B400F / N.Inv: 1037597
N.Reg: 9755/2022
Usuari: san / Mostra: LRG242CH
Nom: LAURA RODRIGUEZ GONZALEZ
Data: 21/07/2022 21:27:21 h./ Ope.: servei Unitat RMN
Experiment: A-H1-cosygpppqf Solvent: CDCl3
f1 (ppm)
f2 (ppm)

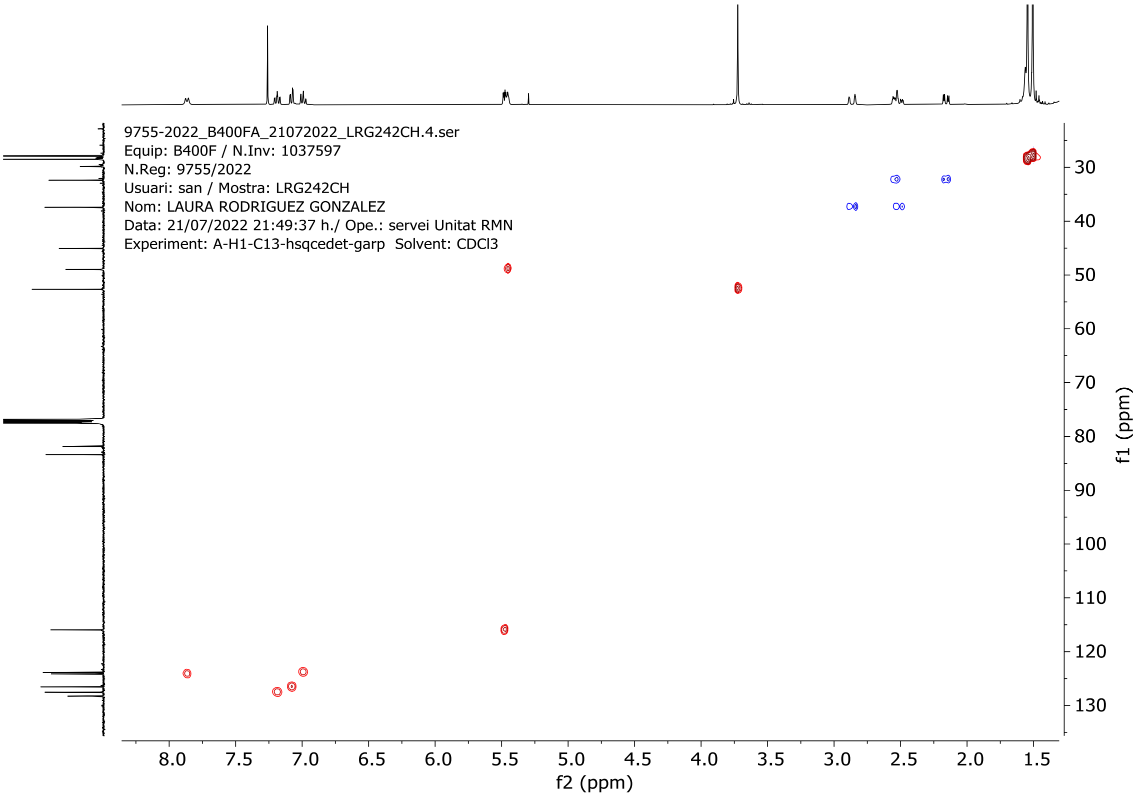

9755-2022_B400FA_21072022_LRG242CH.4.ser
Equip: B400F / N.Inv: 1037597
N.Reg: 9755/2022
Usuari: san / Mostra: LRG242CH
Nom: LAURA RODRIGUEZ GONZALEZ
Data: 21/07/2022 21:49:37 h./ Ope.: servei Unitat RMN
Experiment: A-H1-C13-hsqcedet-garp Solvent: CDCl3
f1 (ppm)
f2 (ppm)

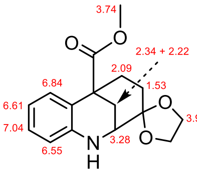

6

22070808_B400FA_26072022_LRG245PROT.1.fid 400 MHz
Equip: B400F / N.Inv: 1037597
N.Reg: 22070808
Usuari: san / Mostra: LRG245PROT
Nom: LAURA RODRIGUEZ GONZALEZ
Data: 26/07/2022 15:52:53 h./ Ope.: AUTOSERVEI
Experiment: A-H1-zg30 Solvent: CDCl3

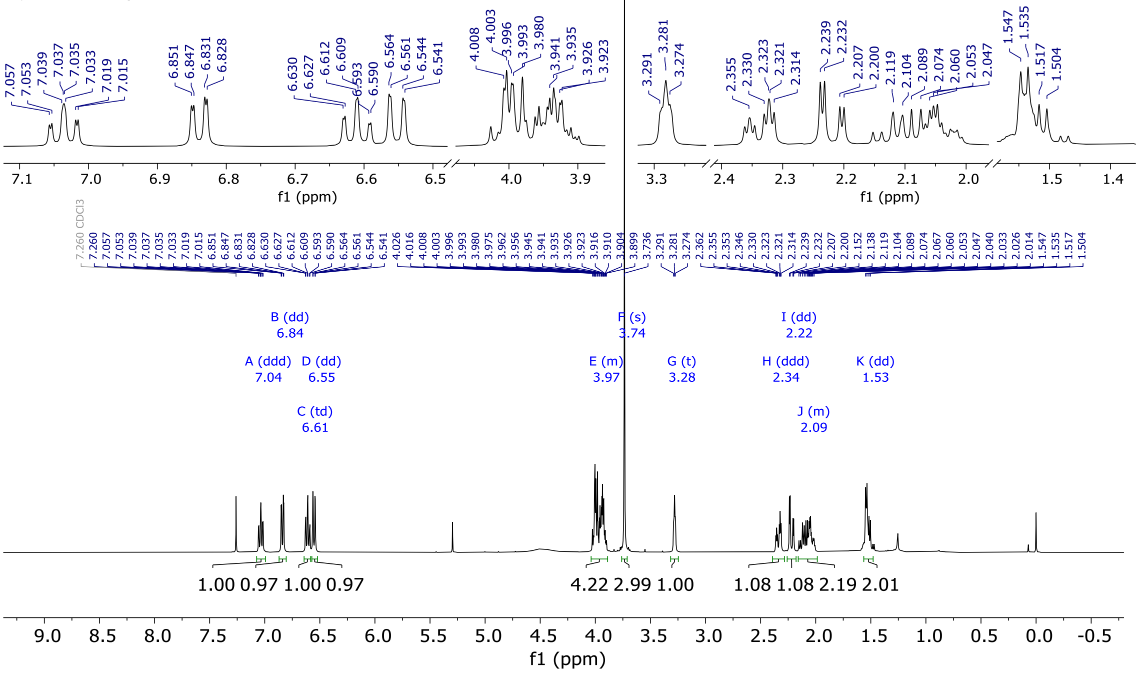

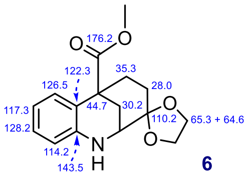

6

san-9944-2022.2.fid LRG245-13C{1H} 101 MHz
Equip: B400Q / N.Inv: 1035091
N.Reg:
Usuari: / Mostra:
Nom:
Data: 27/07/2022 18:10:35 h./ Ope.:

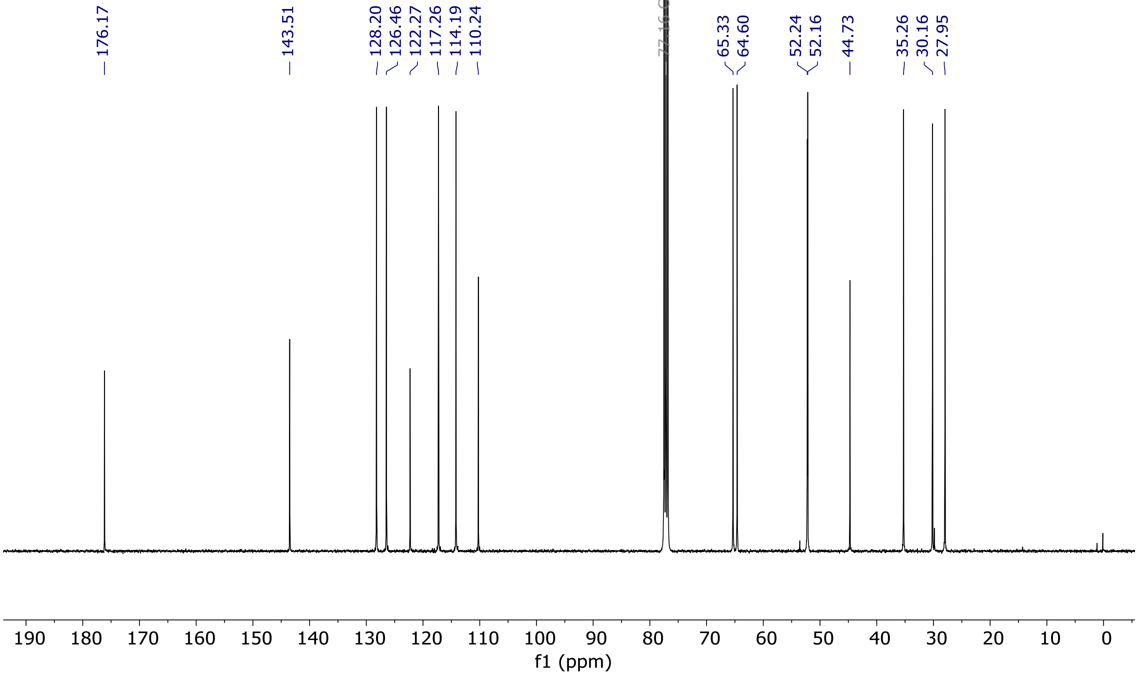

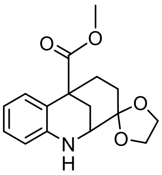

6

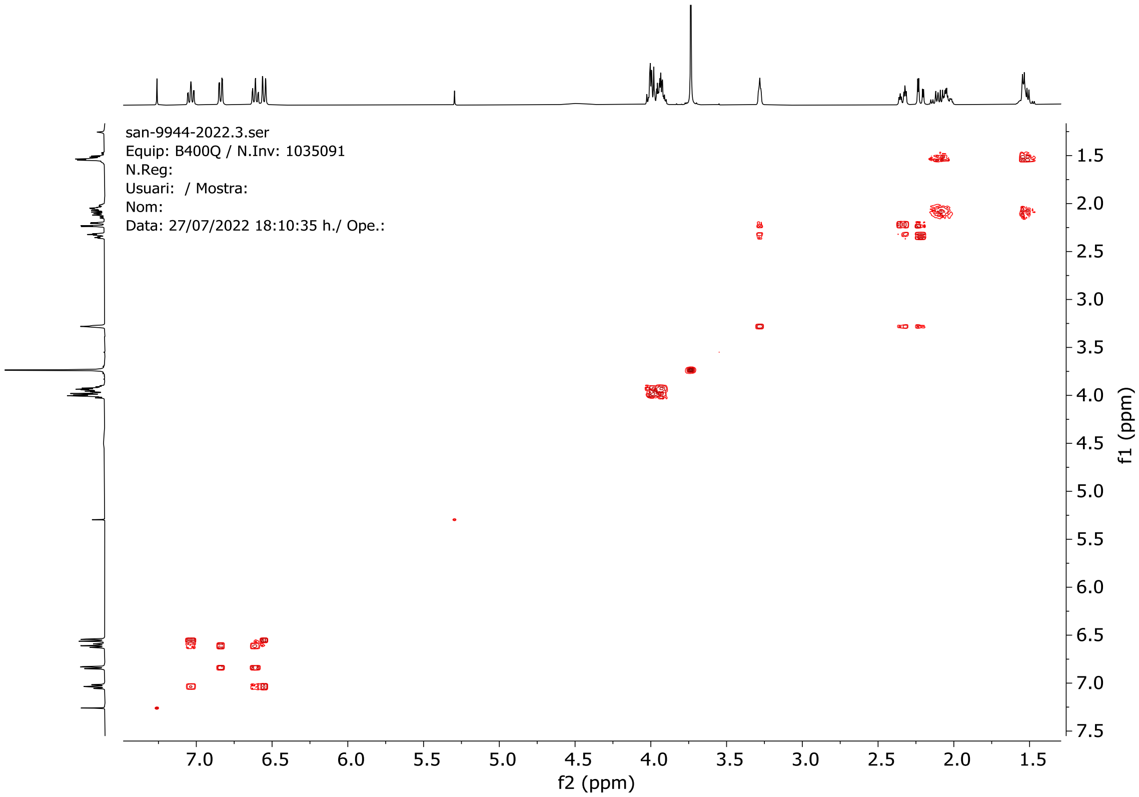
san-9944-2022.3.ser
Equip: B400Q / N.Inv: 1035091
N.Reg:
Usuari: / Mostra:
Nom:
Data: 27/07/2022 18:10:35 h./ Ope.:
f2 (ppm)
f1 (ppm)
7.0 6.5 6.0 5.5 5.0 4.5 4.0 3.5 3.0 2.5 2.0 1.5
1.5 2.0 2.5 3.0 3.5 4.0 4.5 5.0 5.5 6.0 6.5 7.0 7.5

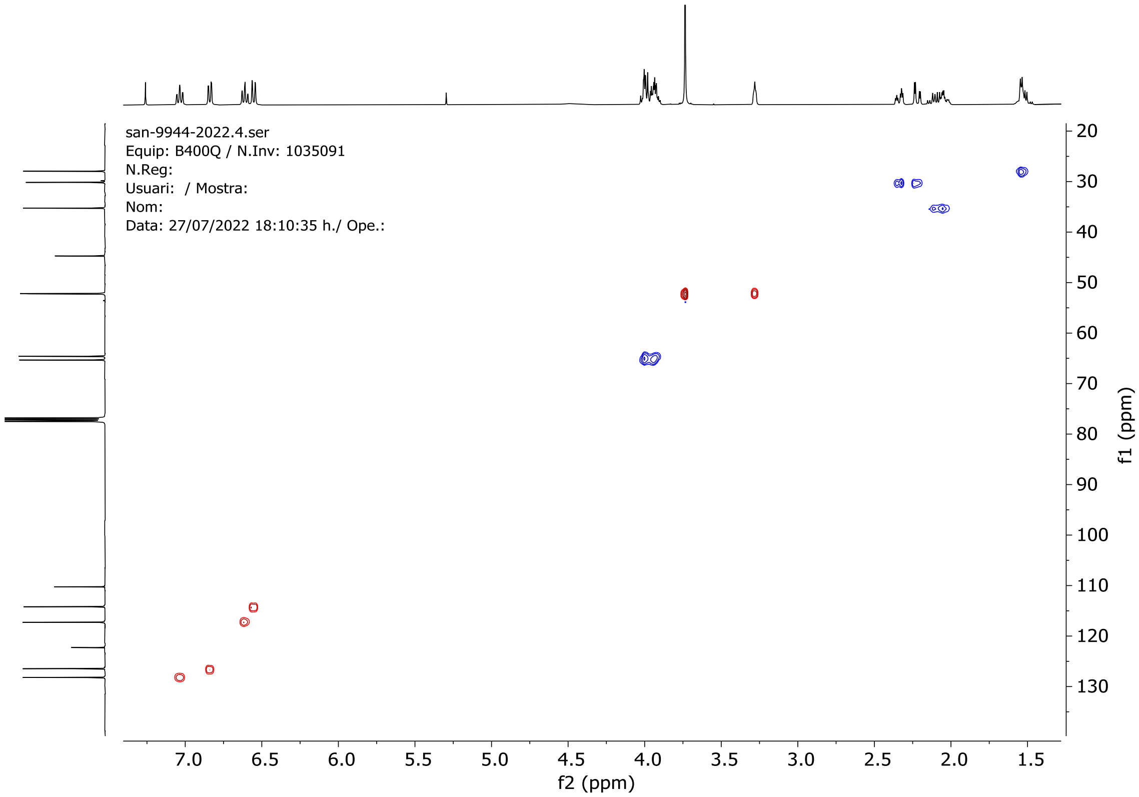
san-9944-2022.4.ser
Equip: B400Q / N.Inv: 1035091
N.Reg:
Usuari: / Mostra:
Nom:
Data: 27/07/2022 18:10:35 h./ Ope.:
f2 (ppm)
f1 (ppm)
7.0 6.5 6.0 5.5 5.0 4.5 4.0 3.5 3.0 2.5 2.0 1.5
20 30 40 50 60 70 80 90 100 110 120 130

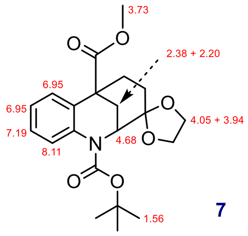

7

22090107_B400FA_06092022_LRG246CH.1.fid 400 MHz
Equip: B400F / N.Inv: 1037597
N.Reg: 22090107
Usuari: san / Mostra: LRG246CH
Nom: LAURA RODRIGUEZ GONZALEZ
Data: 06/09/2022 16:11:25 h./ Ope.: AUTOSERVEI
Experiment: A-H1-zg30 Solvent: CDCl3

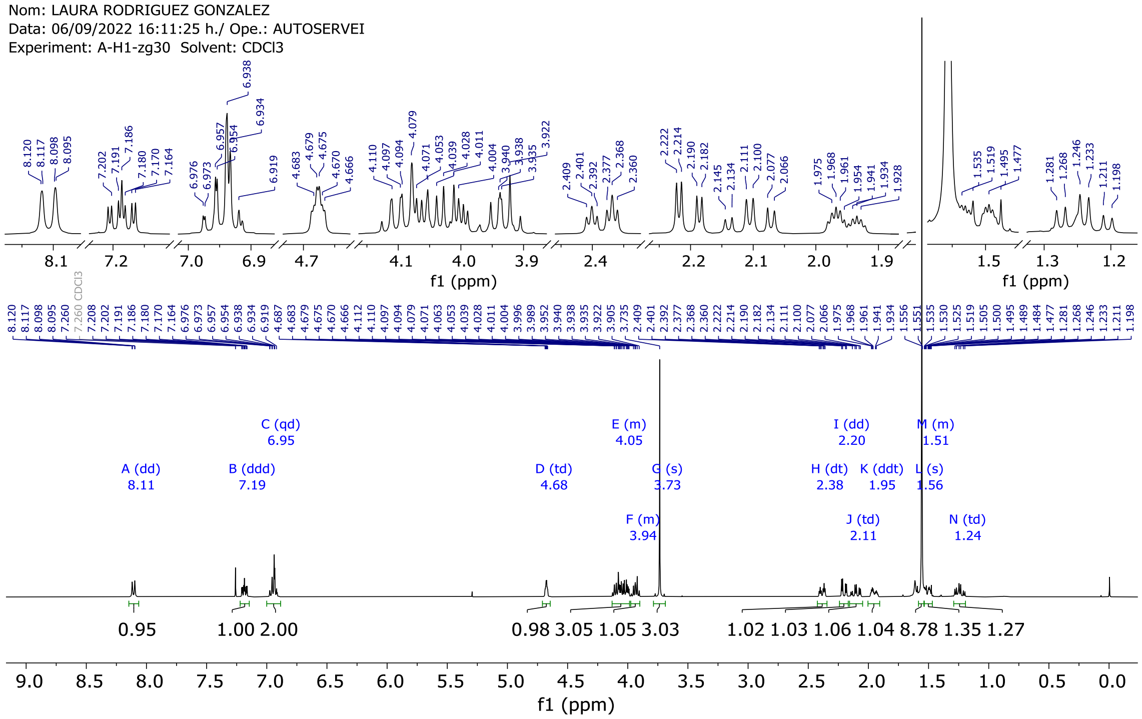

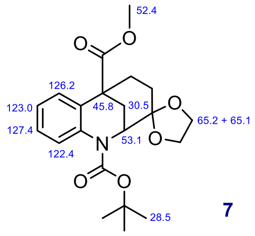

7

22090107_B400FA_07092022_LRG246CH.2.fid 13C{1H} 101 MHz
Equip: B400F / N.Inv: 1037597
N.Reg: 22090107
Usuari: san / Mostra: LRG246CH
Nom: LAURA RODRIGUEZ GONZALEZ
Data: 07/09/2022 03:57:05 h./ Ope.: AUTOSERVEI
Experiment: A-C13-zgpg30 Solvent: CDCl3

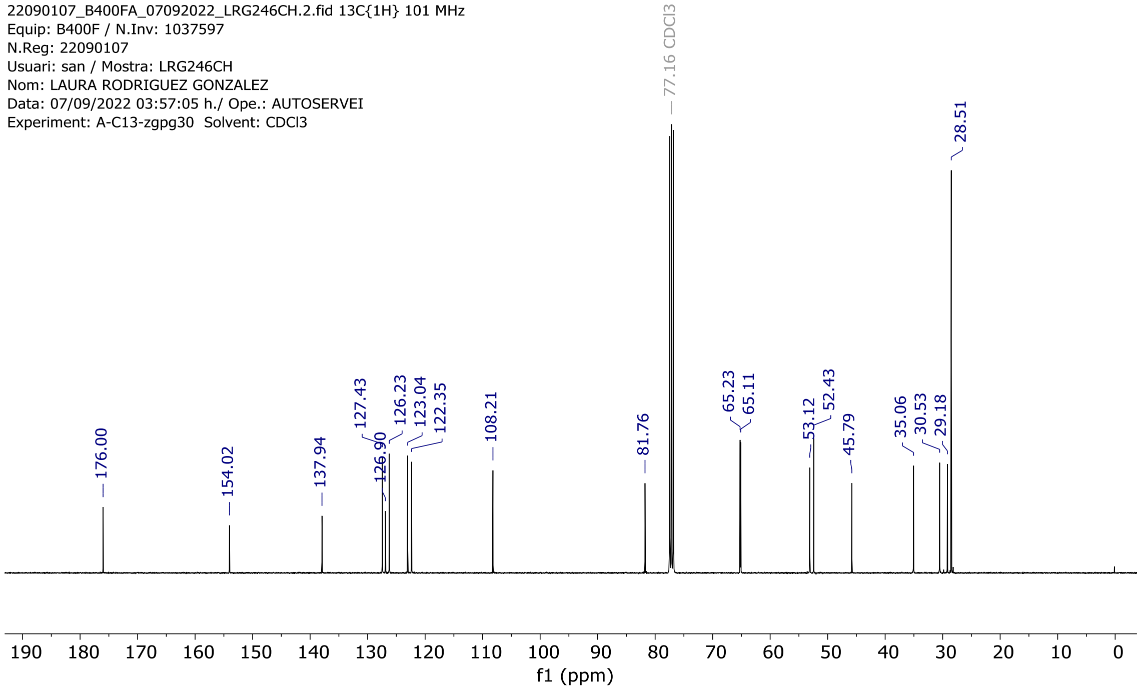

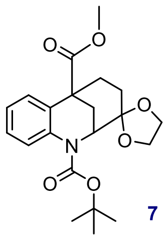

7

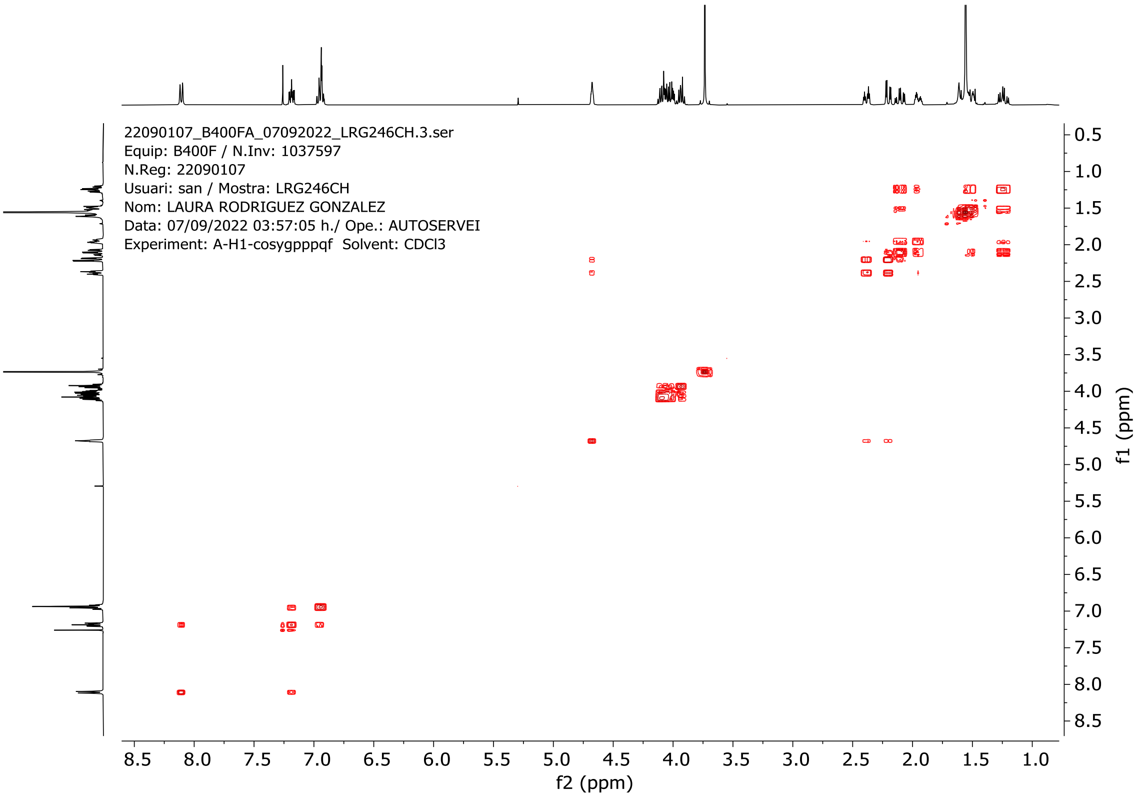
22090107_B400FA_07092022_LRG246CH.3.ser
Equip: B400F / N.Inv: 1037597
N.Reg: 22090107
Usuari: san / Mostra: LRG246CH
Nom: LAURA RODRIGUEZ GONZALEZ
Data: 07/09/2022 03:57:05 h./ Ope.: AUTOSERVEI
Experiment: A-H1-cosygpppqf Solvent: CDCl3
f2 (ppm)
f1 (ppm)

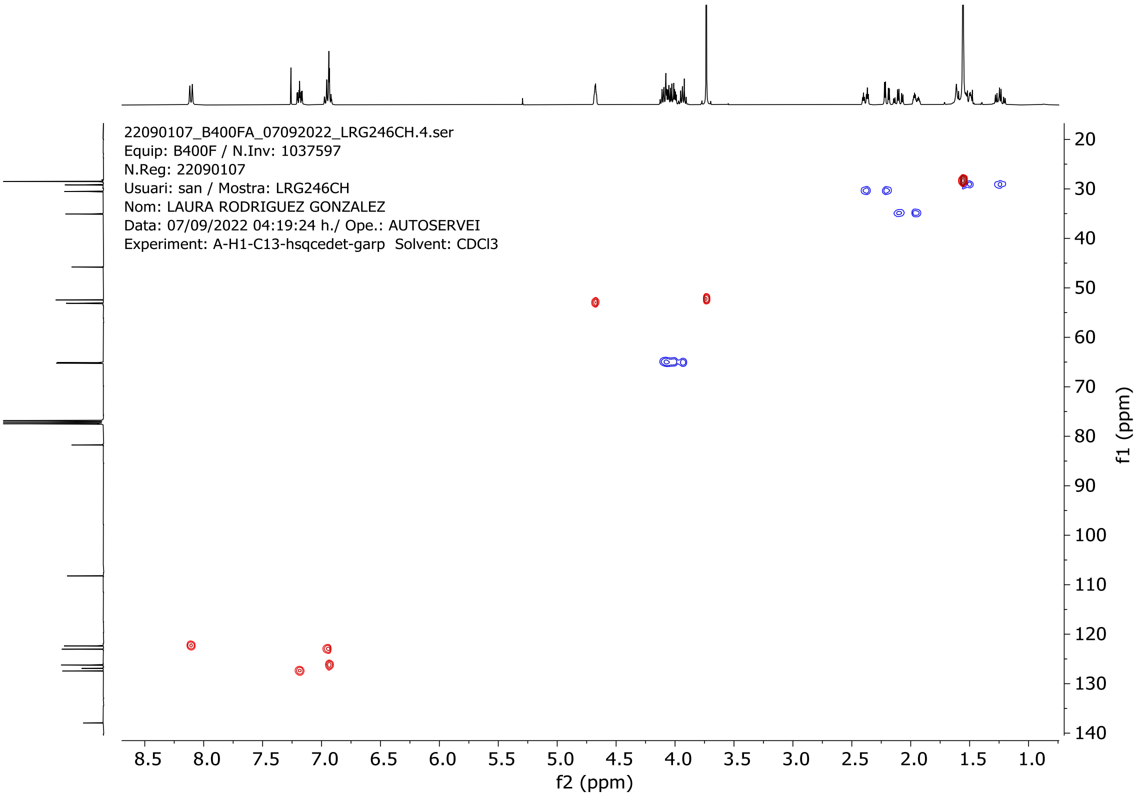
22090107_B400FA_07092022_LRG246CH.4.ser
Equip: B400F / N.Inv: 1037597
N.Reg: 22090107
Usuari: san / Mostra: LRG246CH
Nom: LAURA RODRIGUEZ GONZALEZ
Data: 07/09/2022 04:19:24 h./ Ope.: AUTOSERVEI
Experiment: A-H1-C13-hsqcedet-garp Solvent: CDCl3
f2 (ppm)
f1 (ppm)
