# Supplementary figures and images for: Base-Mediated Nitrophenyl Reductive Cyclization for the Synthesis of Hexahydro-2,6-methano-1-benzazocines
Source: J Org Chem. 2022 Nov 4;87(22):15693–702. doi: 10.1021/acs.joc.2c02205 (PMC9680033; doi:10.1021/acs.joc.2c02205)

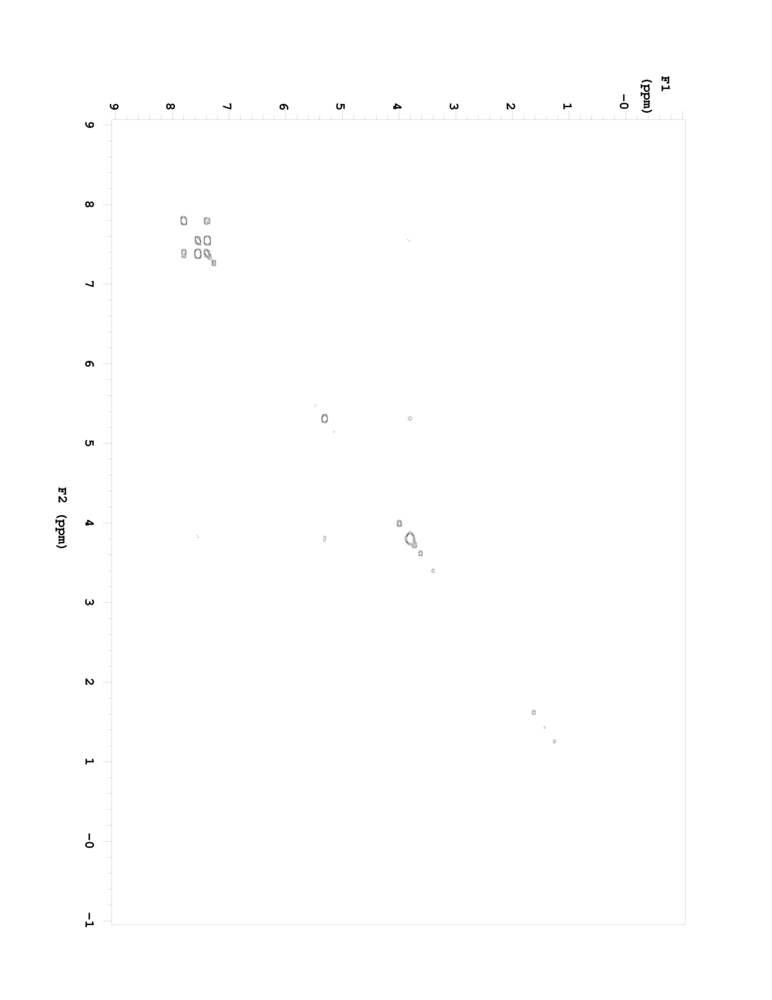

Supplement: Supplementary file 2 — jo2c02205_si_002.zip [file jo2c02205_si_002.zip › FID for publication/Compound 1b/Compound 1b-COSY/espectro.jpg]

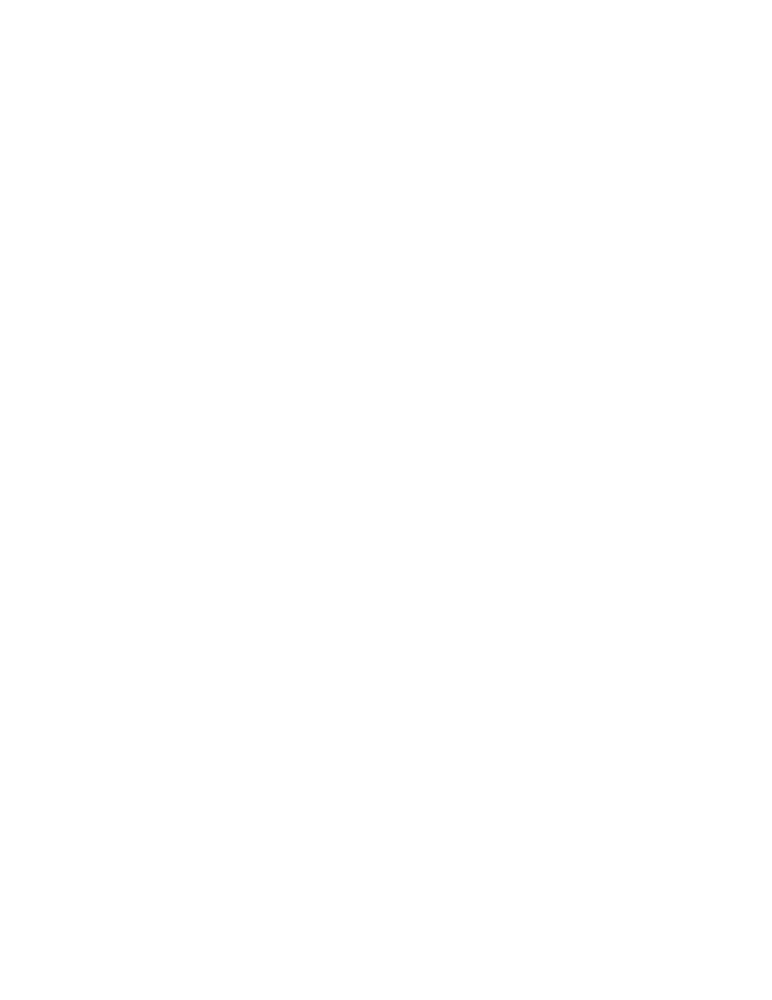

Supplement: Supplementary file 2 — jo2c02205_si_002.zip [file jo2c02205_si_002.zip › FID for publication/Compound 1b/Compound 1b-F19/espectro.jpg]

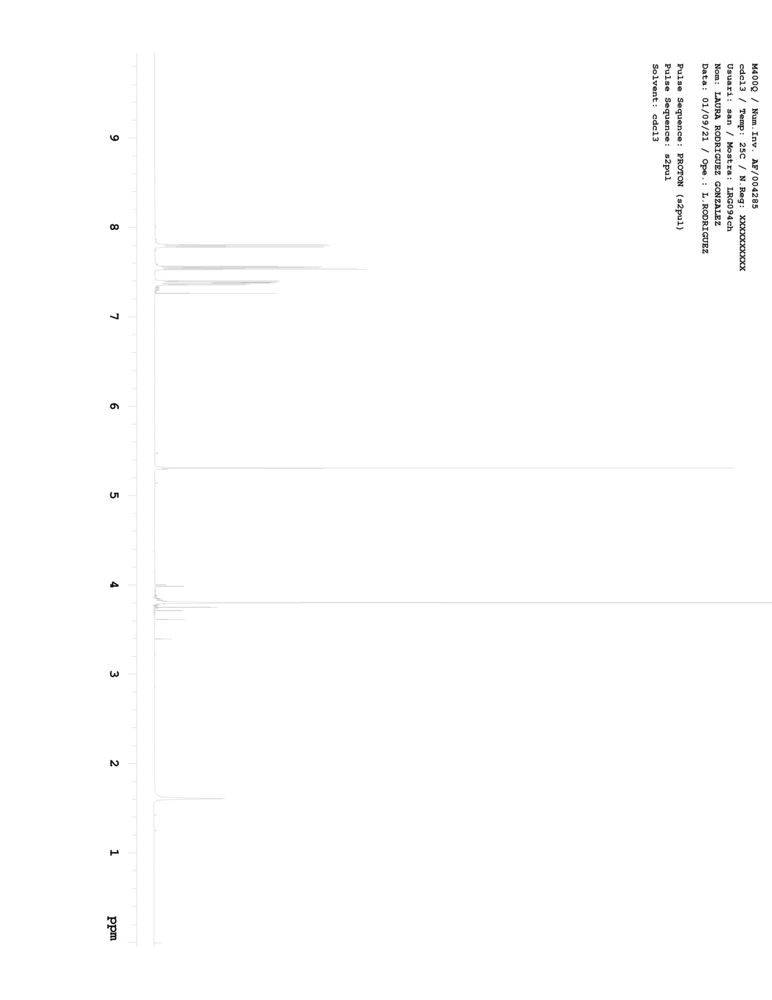

Supplement: Supplementary file 2 — jo2c02205_si_002.zip [file jo2c02205_si_002.zip › FID for publication/Compound 1b/Compound 1b-H1/espectro.jpg]

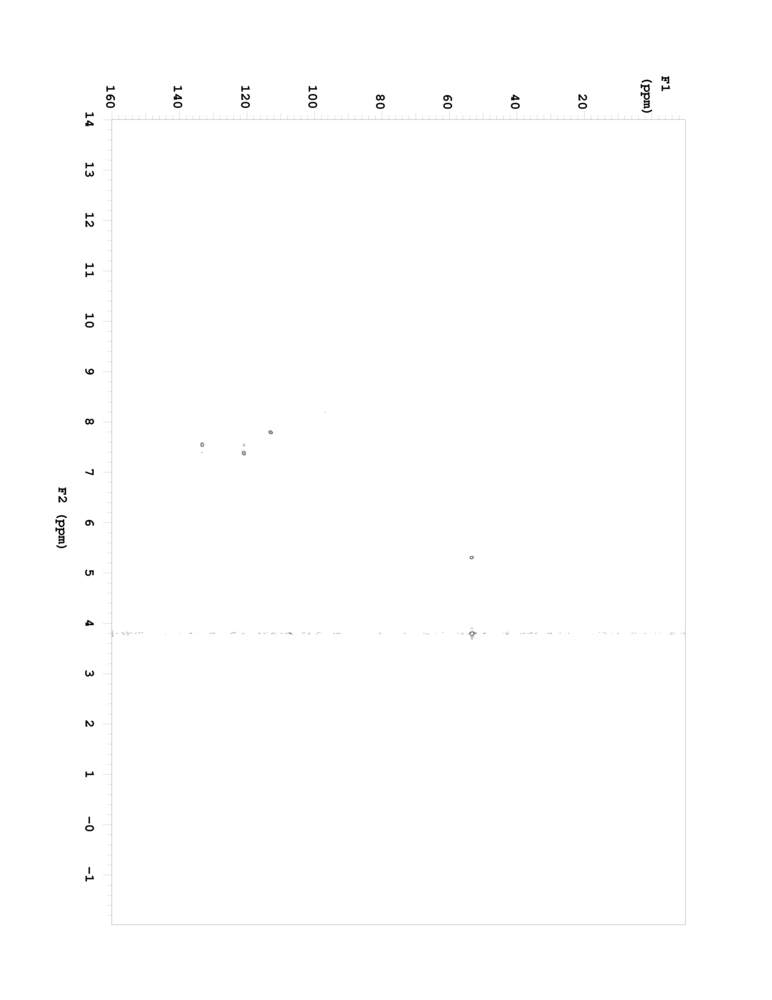

Supplement: Supplementary file 2 — jo2c02205_si_002.zip [file jo2c02205_si_002.zip › FID for publication/Compound 1b/Compound 1b-HSQC/espectro.jpg]

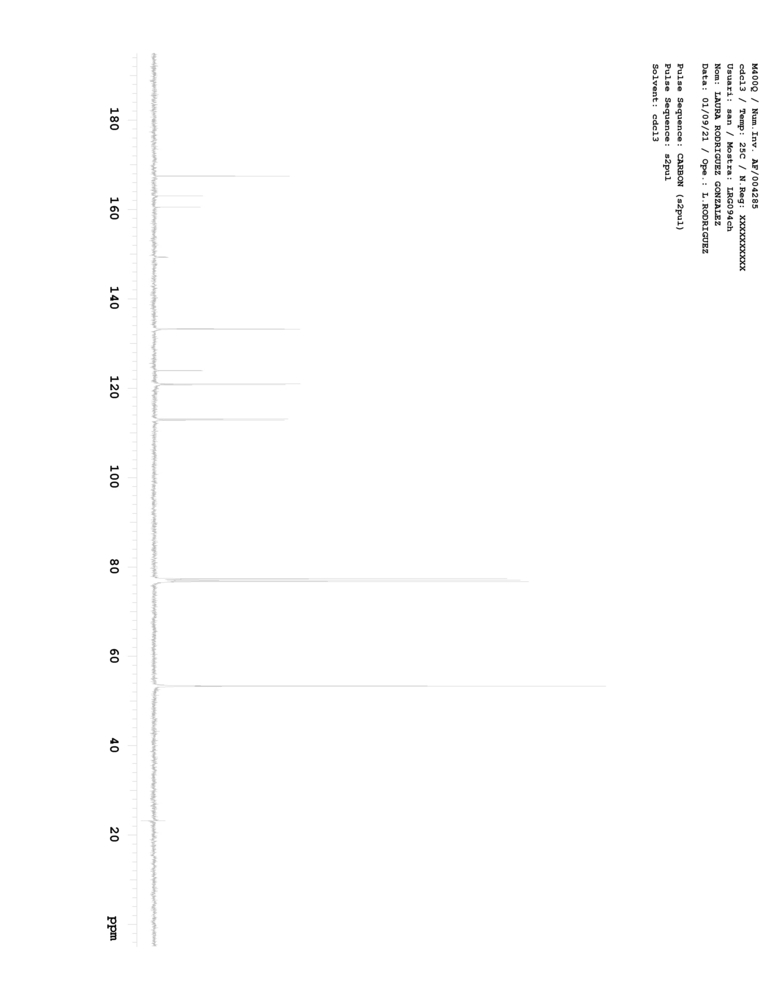

Supplement: Supplementary file 2 — jo2c02205_si_002.zip [file jo2c02205_si_002.zip › FID for publication/Compound 1b/Compund 1b-C13/espectro.jpg]

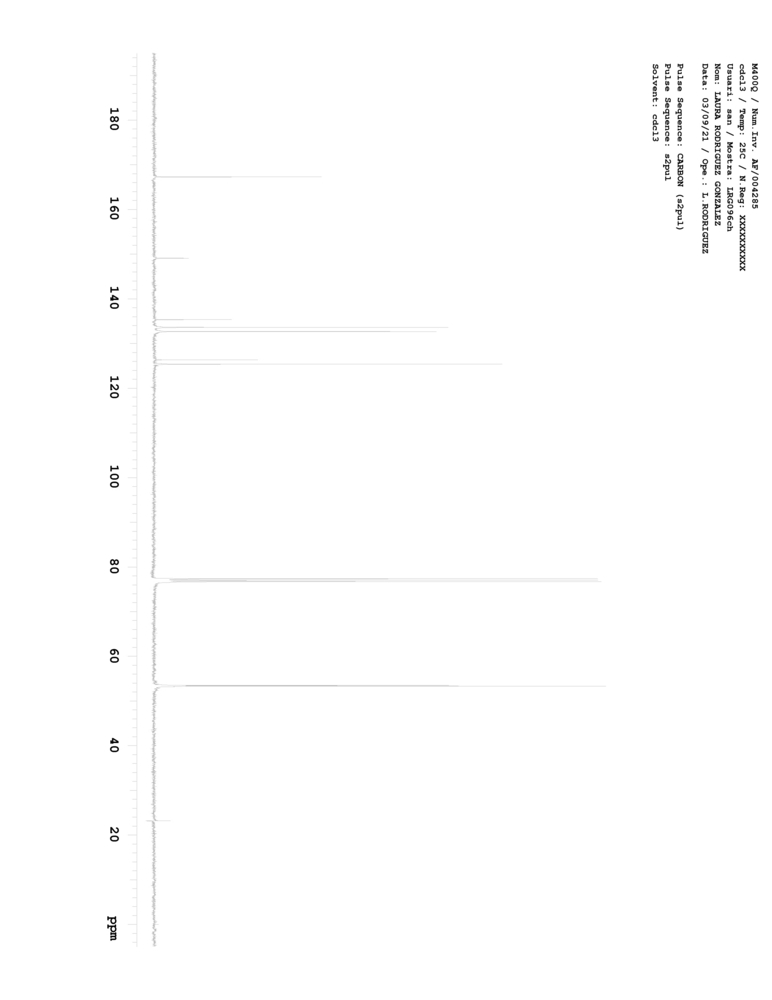

Supplement: Supplementary file 2 — jo2c02205_si_002.zip [file jo2c02205_si_002.zip › FID for publication/Compound 1c/Compound 1c-C13/espectro.jpg]

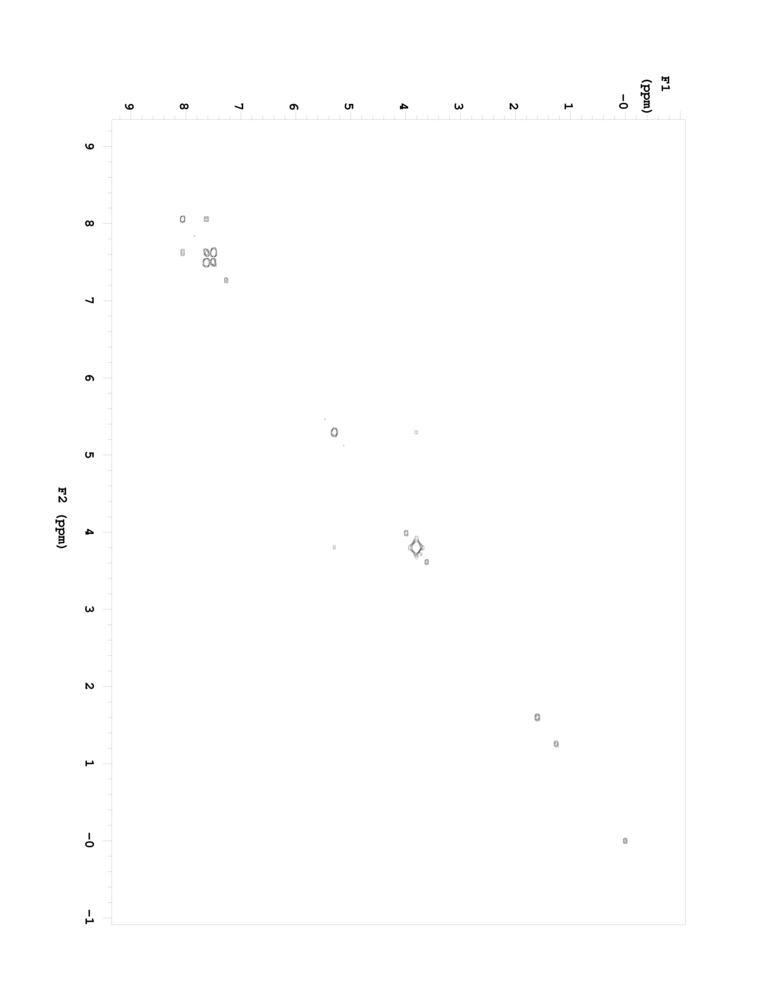

Supplement: Supplementary file 2 — jo2c02205_si_002.zip [file jo2c02205_si_002.zip › FID for publication/Compound 1c/Compound 1c-COSY/espectro.jpg]

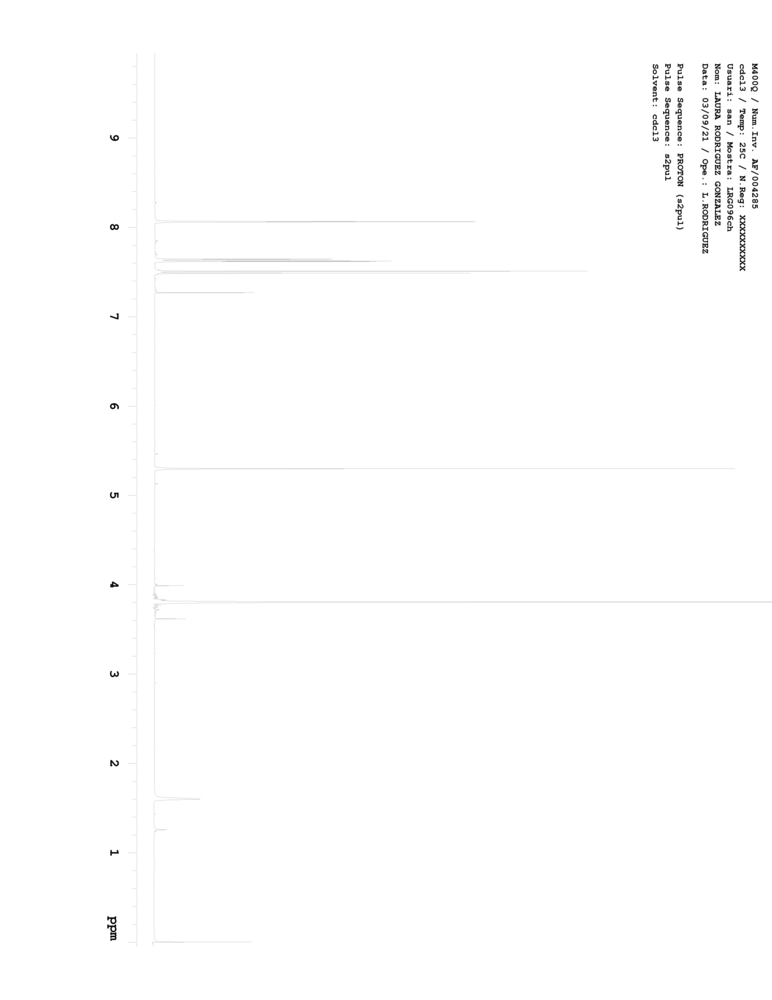

Supplement: Supplementary file 2 — jo2c02205_si_002.zip [file jo2c02205_si_002.zip › FID for publication/Compound 1c/Compound 1c-H1/espectro.jpg]

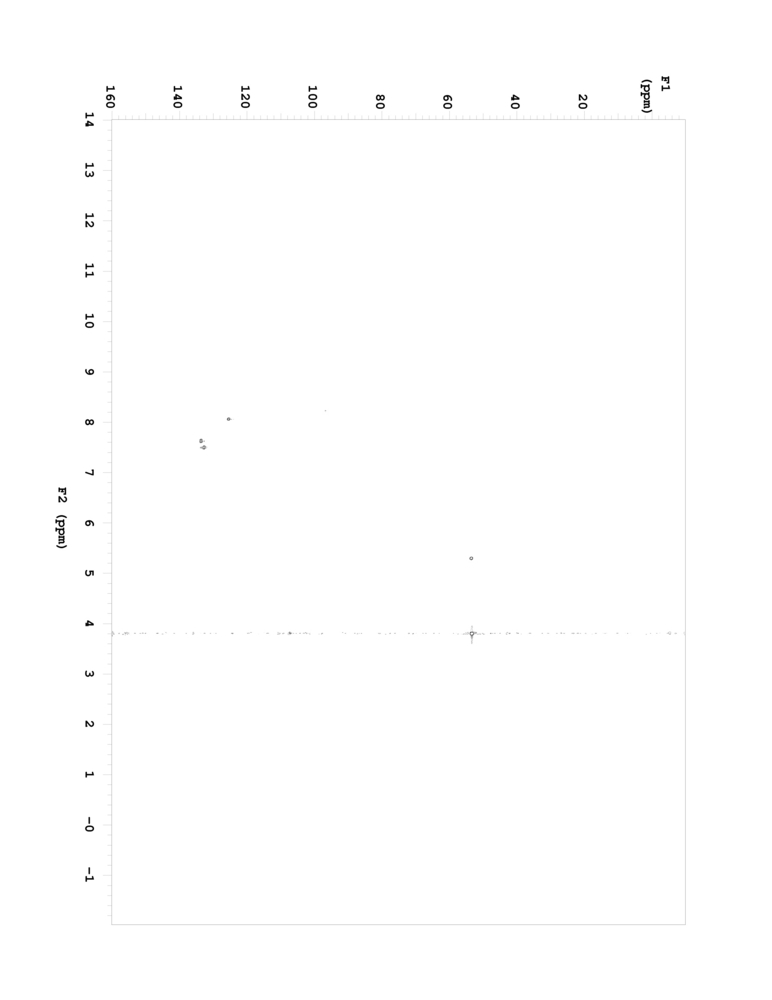

Supplement: Supplementary file 2 — jo2c02205_si_002.zip [file jo2c02205_si_002.zip › FID for publication/Compound 1c/Compound 1c-HSQC/espectro.jpg]

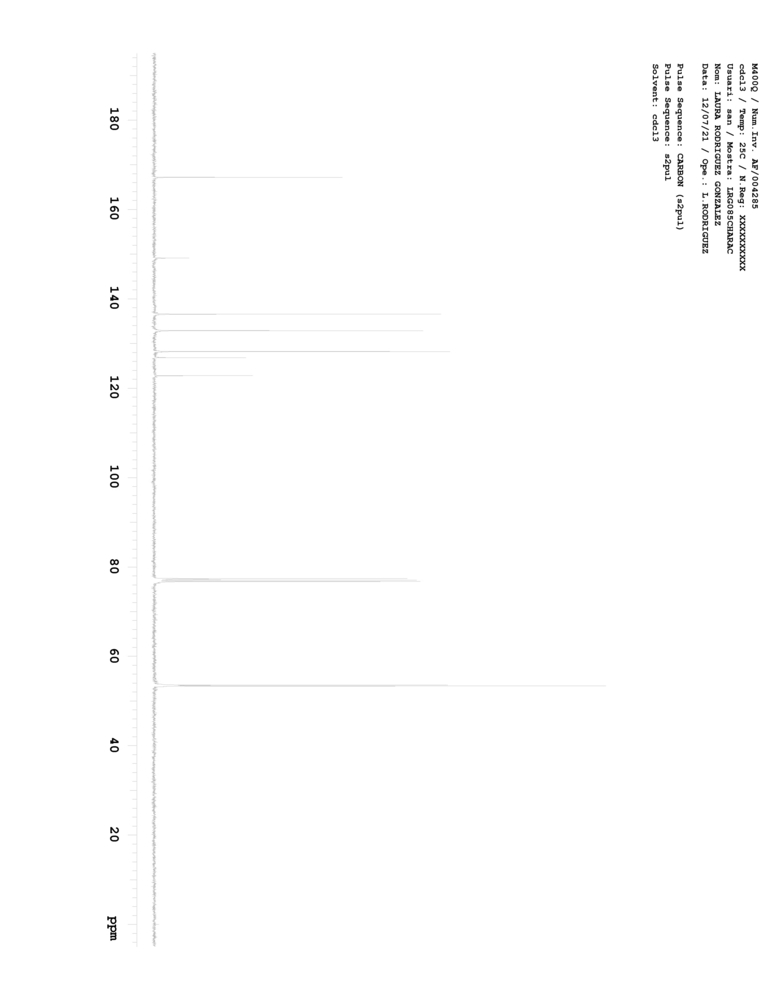

Supplement: Supplementary file 2 — jo2c02205_si_002.zip [file jo2c02205_si_002.zip › FID for publication/Compound 1d/Compound 1d-C13/espectro.jpg]

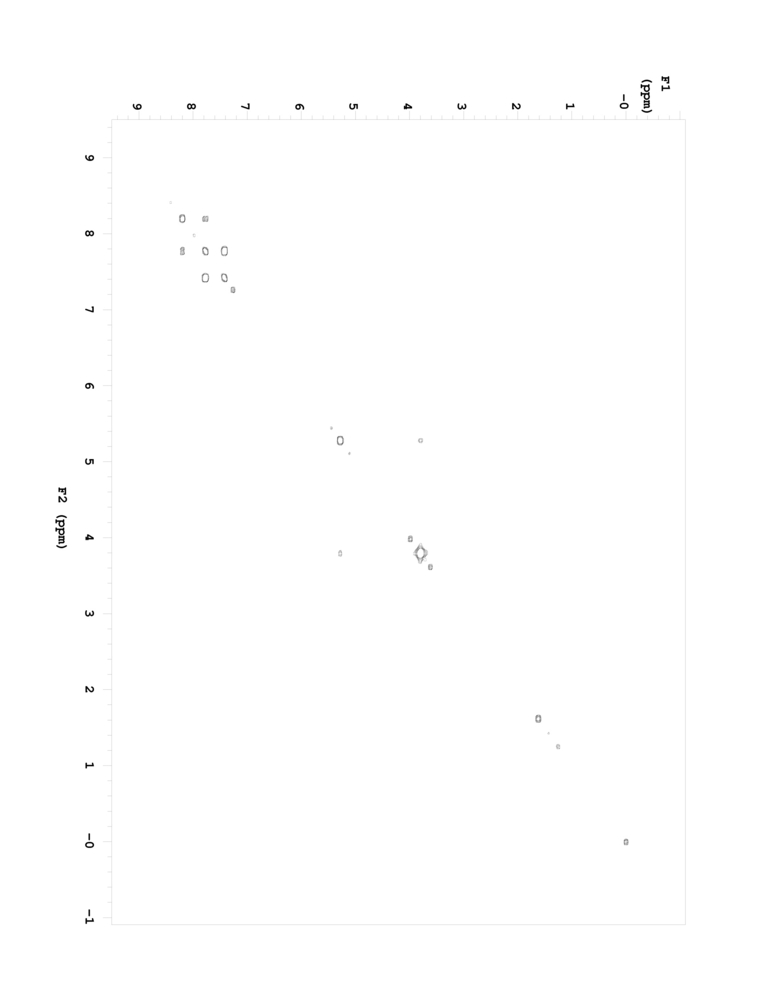

Supplement: Supplementary file 2 — jo2c02205_si_002.zip [file jo2c02205_si_002.zip › FID for publication/Compound 1d/Compound 1d-COSY/espectro.jpg]

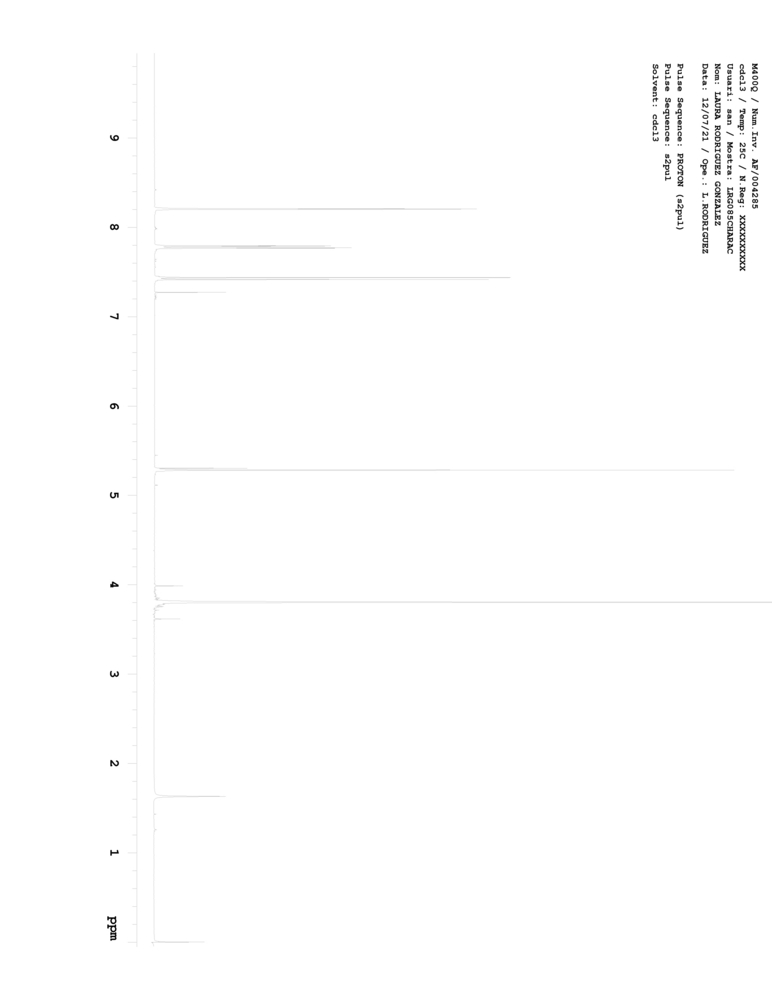

Supplement: Supplementary file 2 — jo2c02205_si_002.zip [file jo2c02205_si_002.zip › FID for publication/Compound 1d/Compound 1d-H1/espectro.jpg]

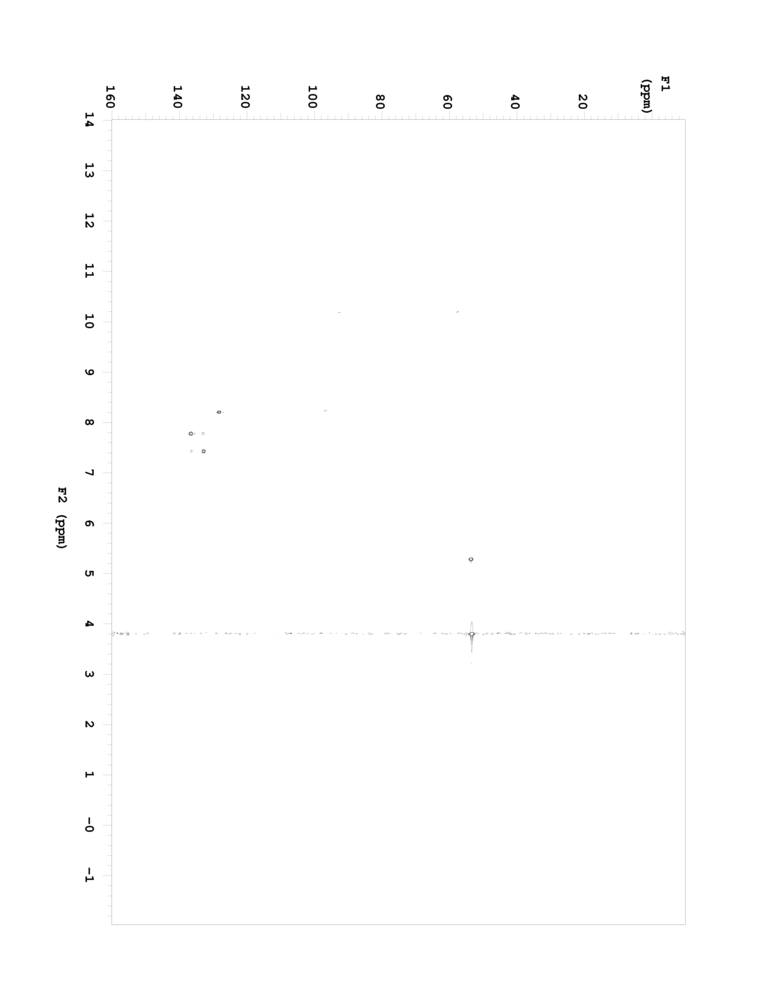

Supplement: Supplementary file 2 — jo2c02205_si_002.zip [file jo2c02205_si_002.zip › FID for publication/Compound 1d/Compound 1d-HSQC/espectro.jpg]

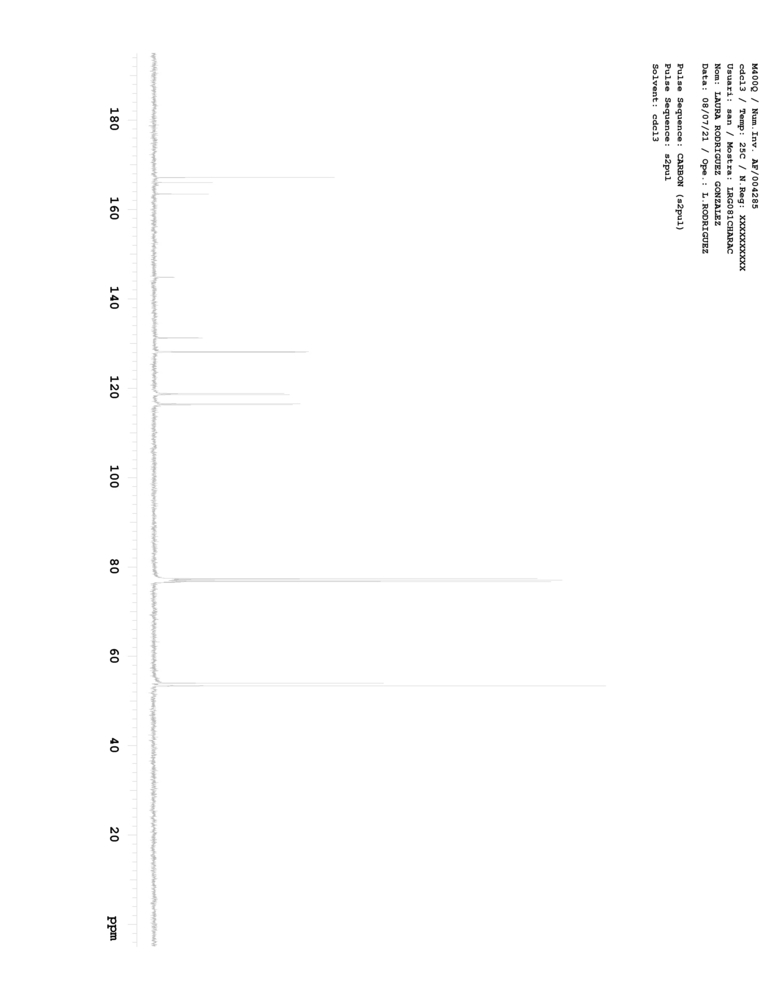

Supplement: Supplementary file 2 — jo2c02205_si_002.zip [file jo2c02205_si_002.zip › FID for publication/Compound 1e/Compound 1e-C13/espectro.jpg]

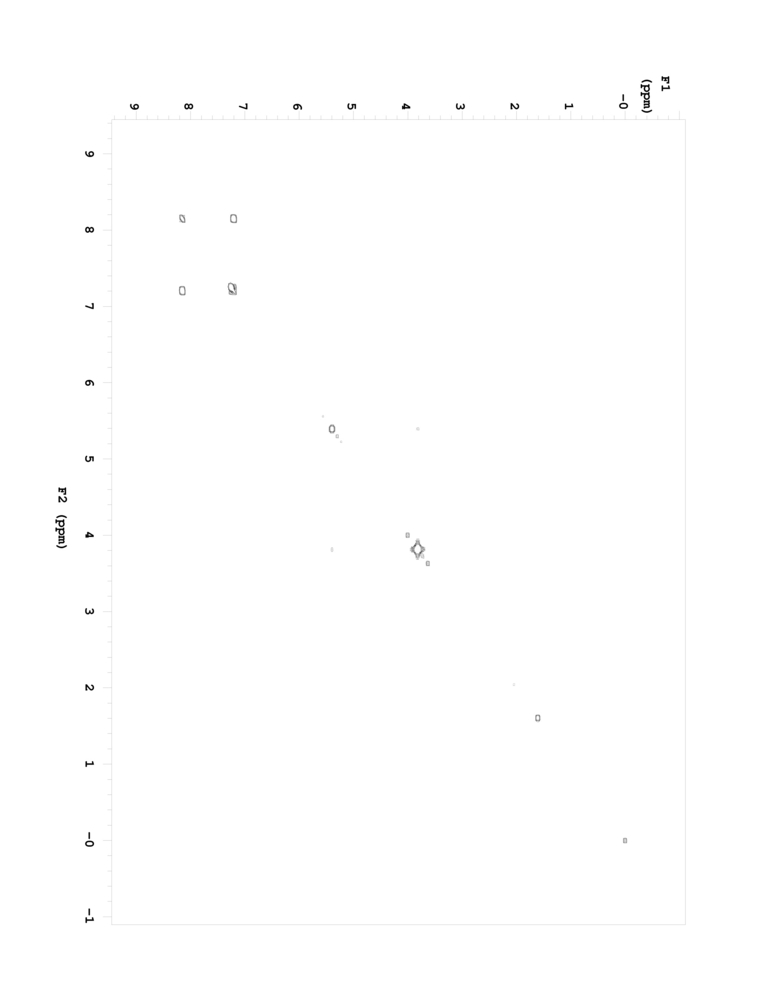

Supplement: Supplementary file 2 — jo2c02205_si_002.zip [file jo2c02205_si_002.zip › FID for publication/Compound 1e/Compound 1e-COSY/espectro.jpg]

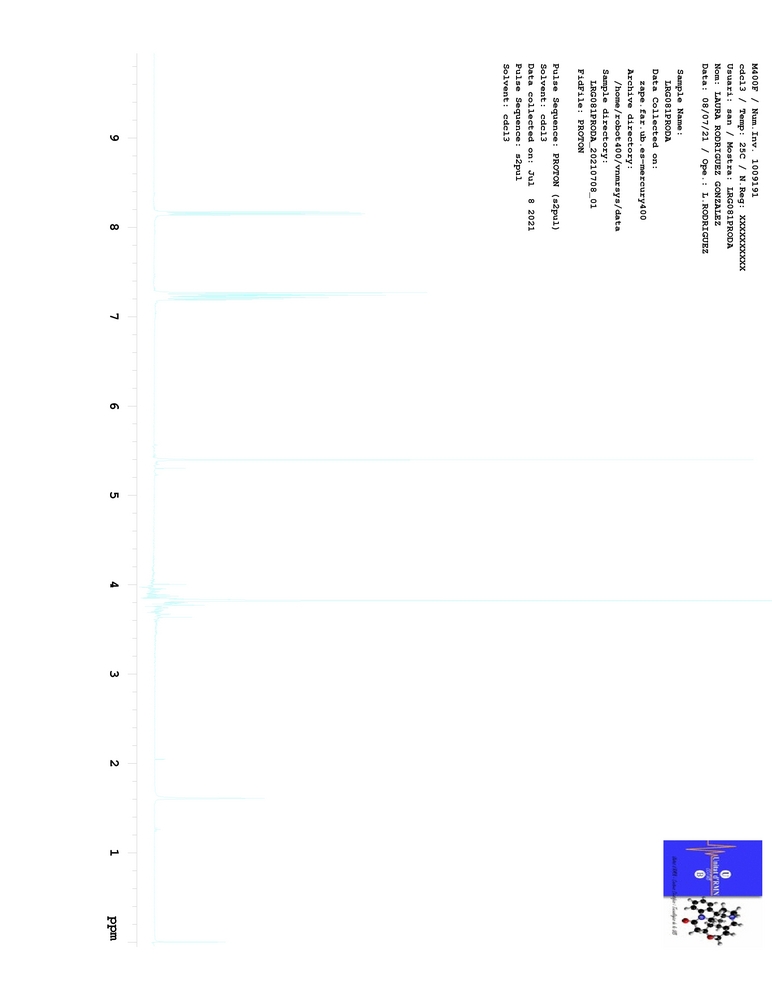

Supplement: Supplementary file 2 — jo2c02205_si_002.zip [file jo2c02205_si_002.zip › FID for publication/Compound 1e/Compound 1e-H1/espectro.jpg]

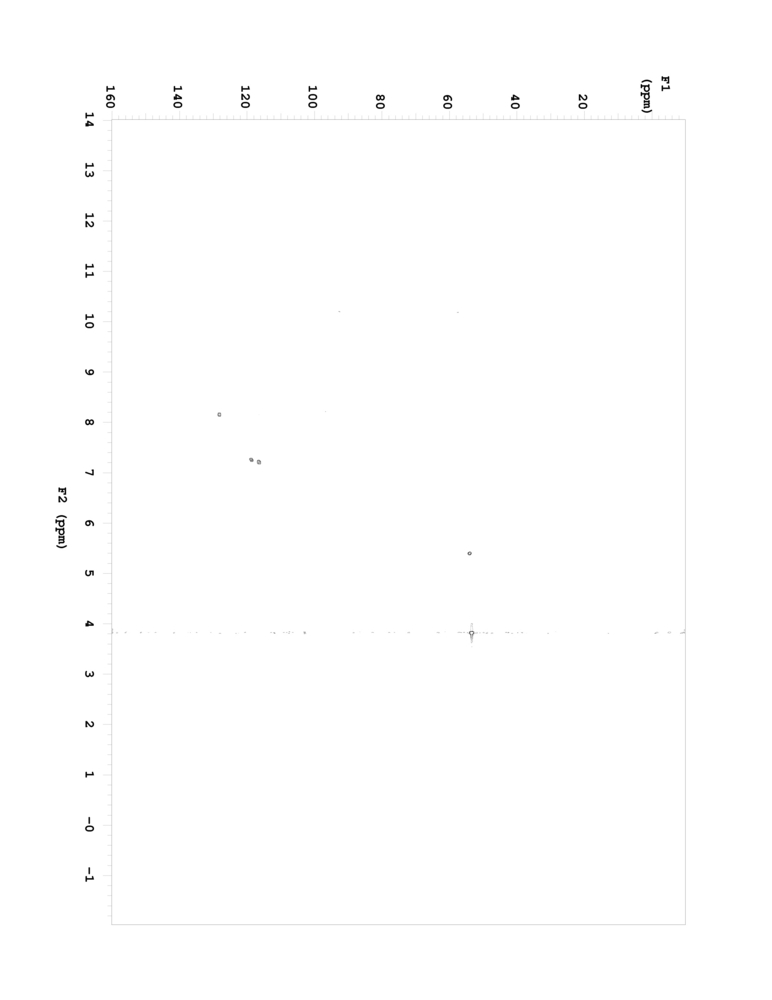

Supplement: Supplementary file 2 — jo2c02205_si_002.zip [file jo2c02205_si_002.zip › FID for publication/Compound 1e/Compound 1e-HSQC/espectro.jpg]

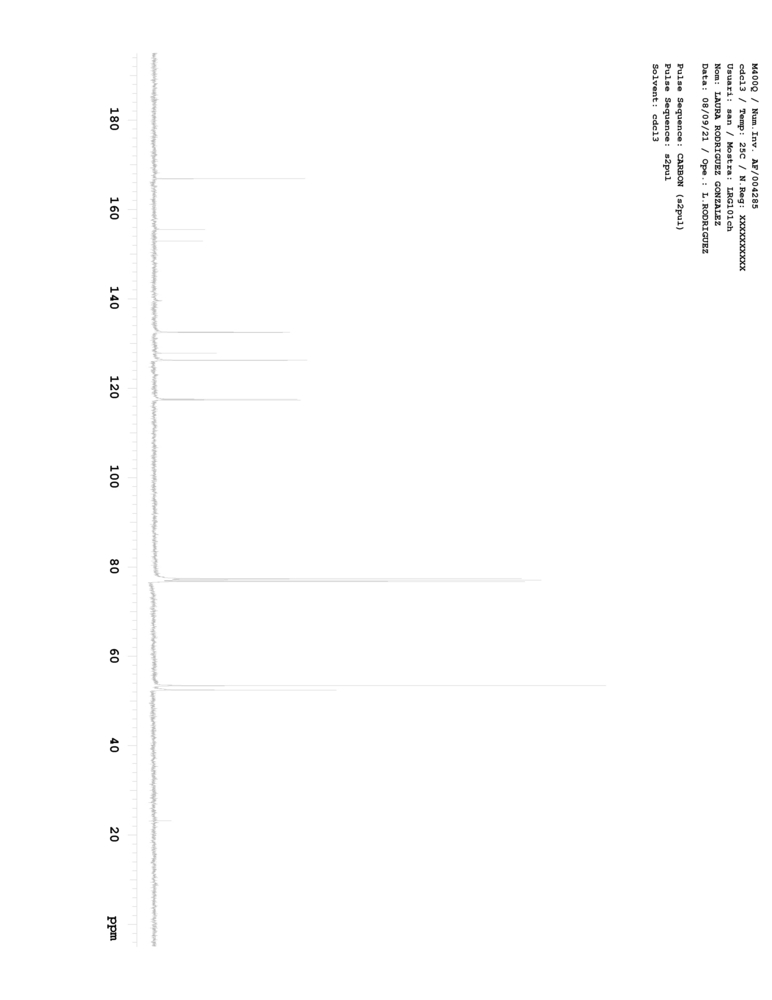

Supplement: Supplementary file 2 — jo2c02205_si_002.zip [file jo2c02205_si_002.zip › FID for publication/Compound 1f/Compound 1f-C13/espectro.jpg]

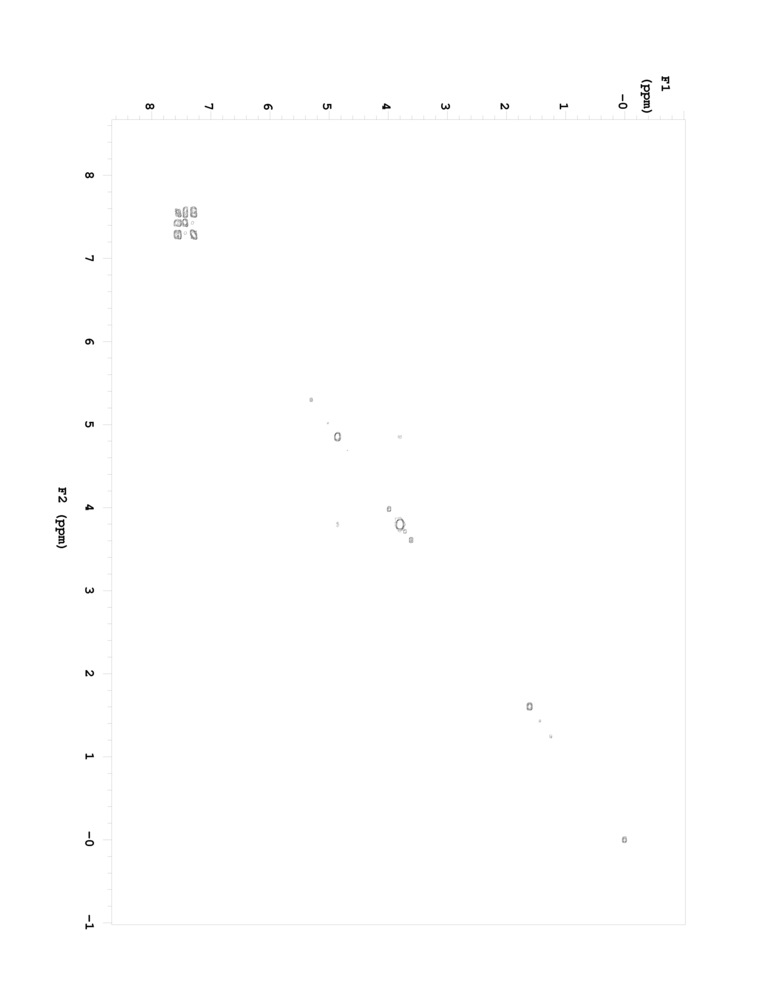

Supplement: Supplementary file 2 — jo2c02205_si_002.zip [file jo2c02205_si_002.zip › FID for publication/Compound 1f/Compound 1f-COSY/espectro.jpg]

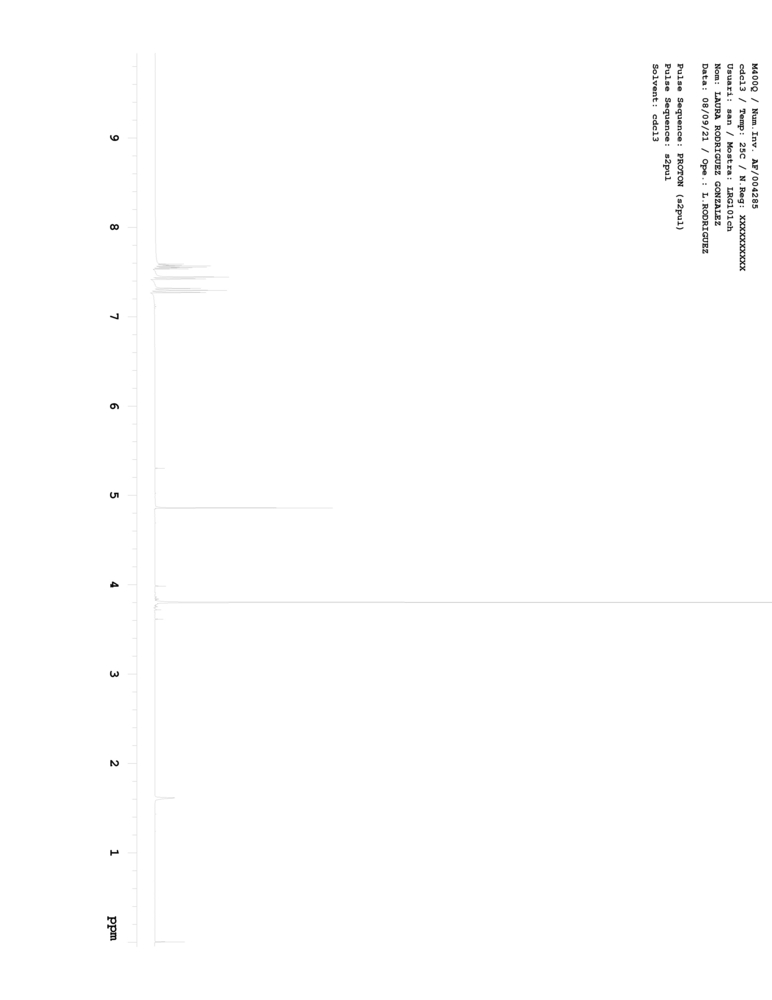

Supplement: Supplementary file 2 — jo2c02205_si_002.zip [file jo2c02205_si_002.zip › FID for publication/Compound 1f/Compound 1f-H1/espectro.jpg]

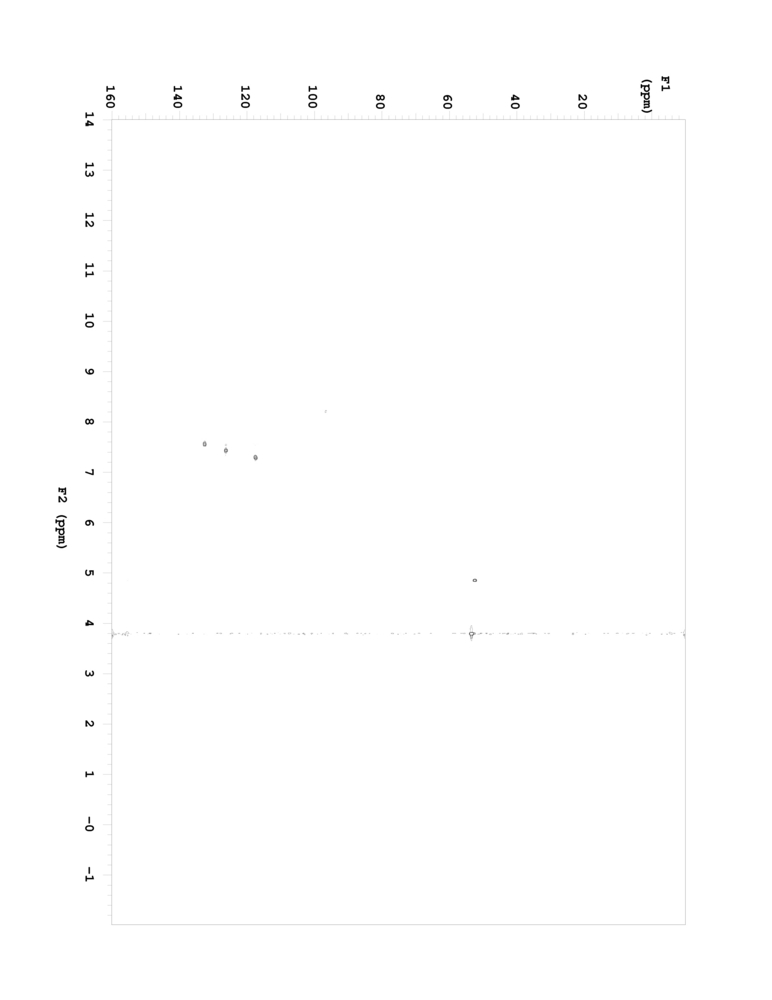

Supplement: Supplementary file 2 — jo2c02205_si_002.zip [file jo2c02205_si_002.zip › FID for publication/Compound 1f/Compound 1f-HSQC/espectro.jpg]

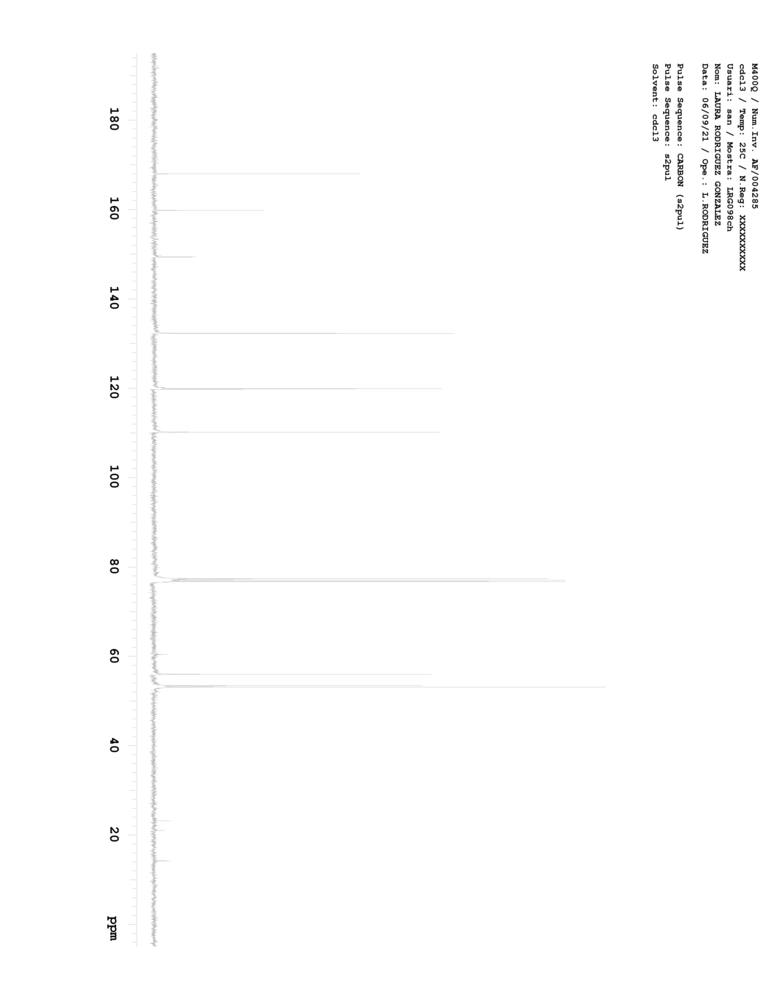

Supplement: Supplementary file 2 — jo2c02205_si_002.zip [file jo2c02205_si_002.zip › FID for publication/Compound 1g/Compound 1g-C13/espectro.jpg]

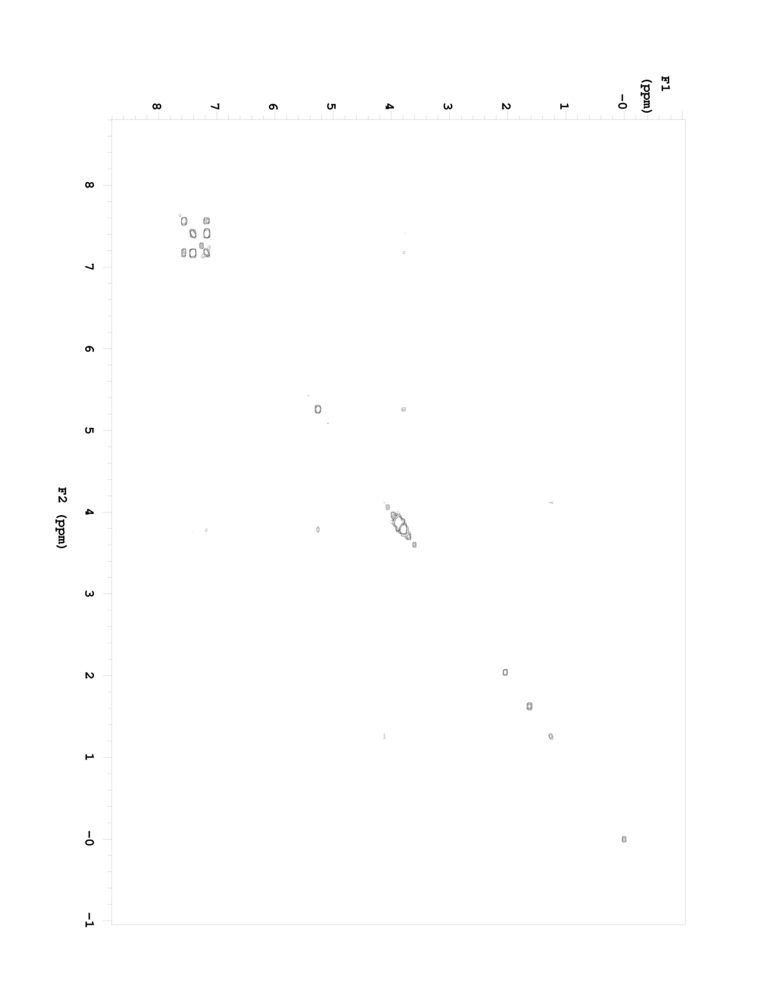

Supplement: Supplementary file 2 — jo2c02205_si_002.zip [file jo2c02205_si_002.zip › FID for publication/Compound 1g/Compound 1g-COSY/espectro.jpg]

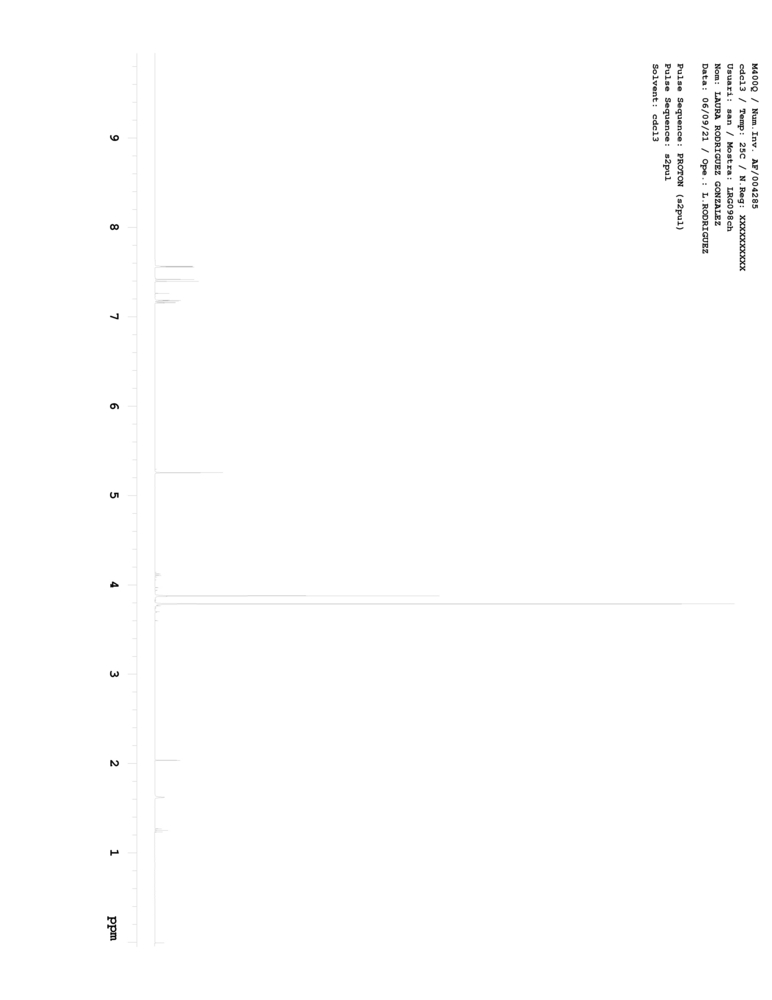

Supplement: Supplementary file 2 — jo2c02205_si_002.zip [file jo2c02205_si_002.zip › FID for publication/Compound 1g/Compound 1g-H1/espectro.jpg]

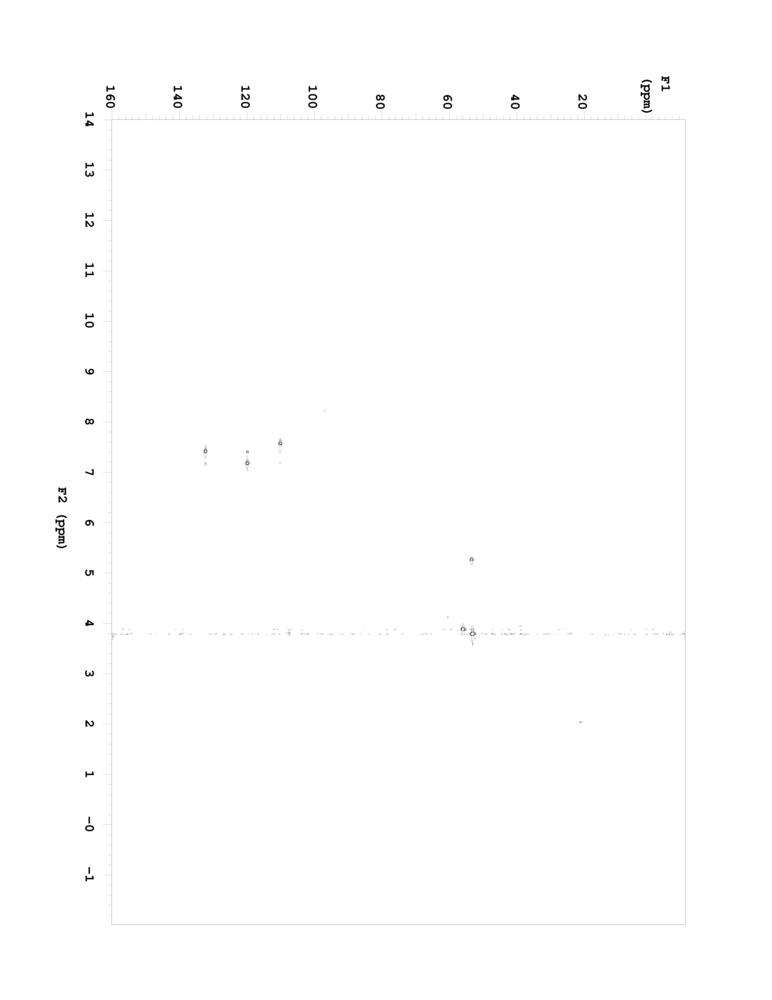

Supplement: Supplementary file 2 — jo2c02205_si_002.zip [file jo2c02205_si_002.zip › FID for publication/Compound 1g/Compound 1g-HSQC/espectro.jpg]

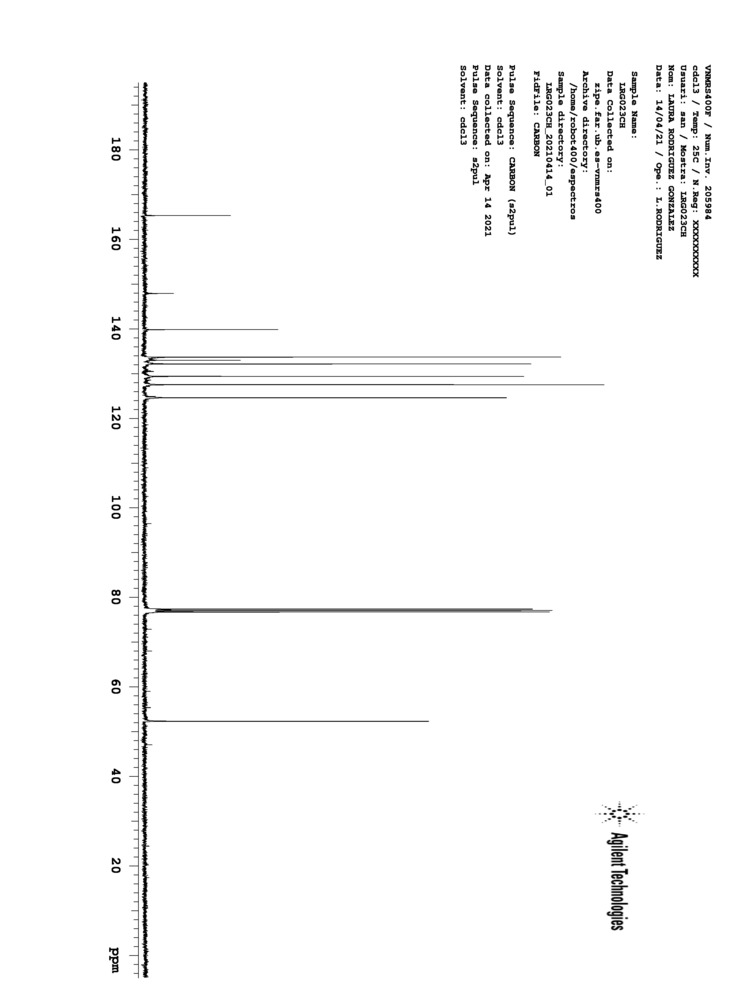

Supplement: Supplementary file 2 — jo2c02205_si_002.zip [file jo2c02205_si_002.zip › FID for publication/Compound 2a/Compound 2a-C13/espectro.jpg]

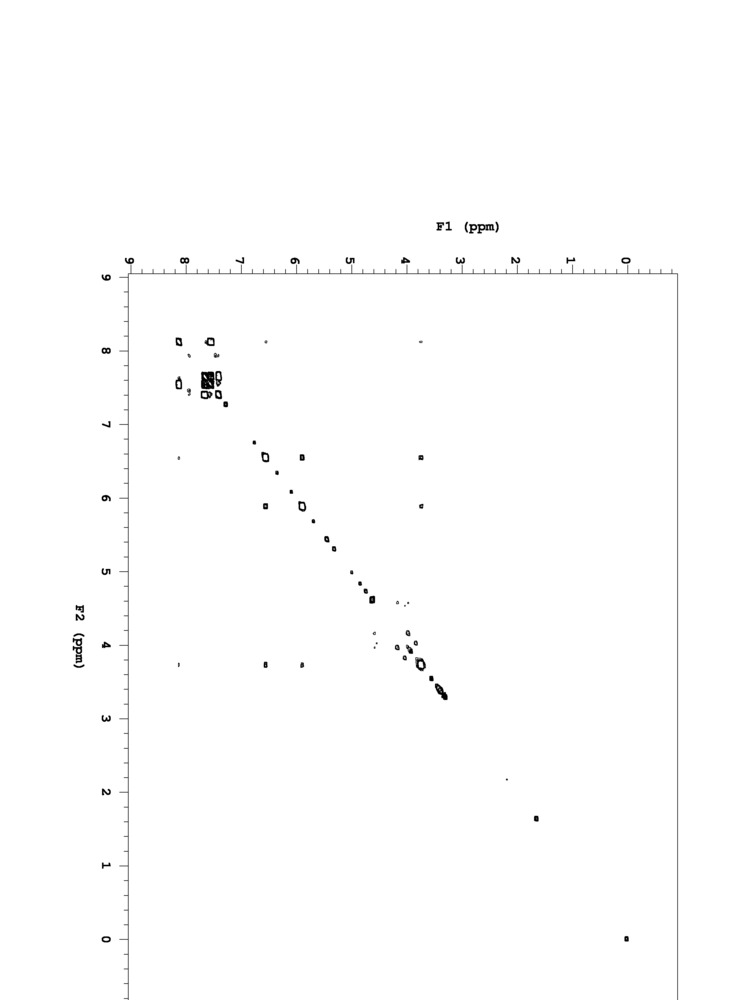

Supplement: Supplementary file 2 — jo2c02205_si_002.zip [file jo2c02205_si_002.zip › FID for publication/Compound 2a/Compound 2a-COSY/espectro.jpg]

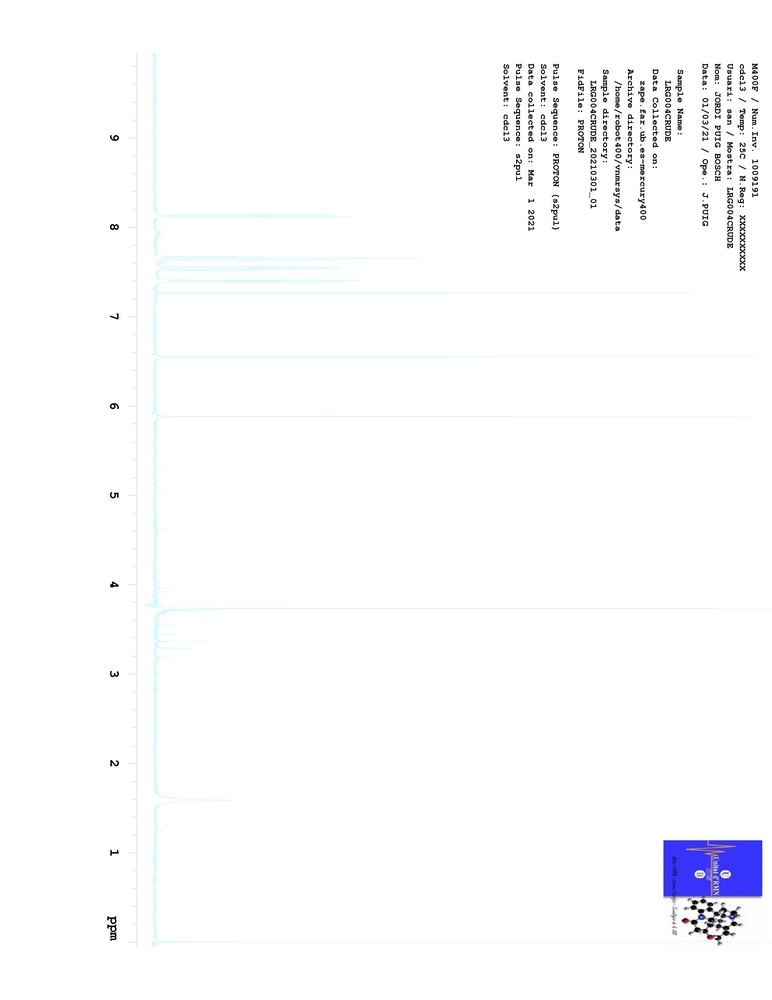

Supplement: Supplementary file 2 — jo2c02205_si_002.zip [file jo2c02205_si_002.zip › FID for publication/Compound 2a/Compound 2a-H1/espectro.jpg]

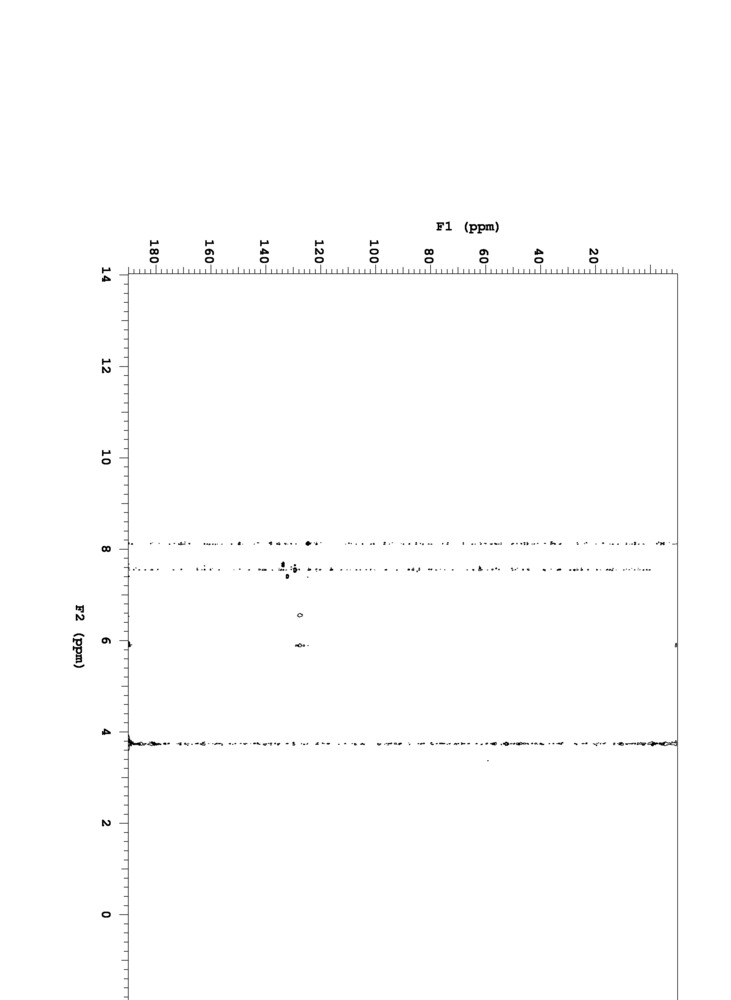

Supplement: Supplementary file 2 — jo2c02205_si_002.zip [file jo2c02205_si_002.zip › FID for publication/Compound 2a/Compound 2a-HSQC/espectro.jpg]

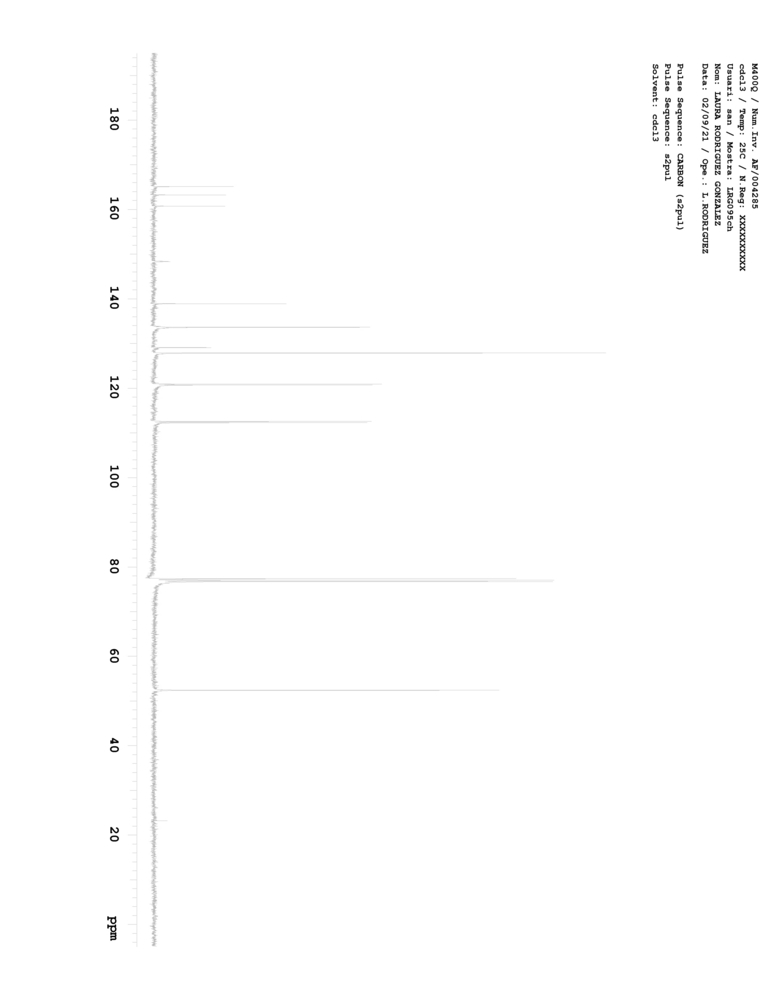

Supplement: Supplementary file 2 — jo2c02205_si_002.zip [file jo2c02205_si_002.zip › FID for publication/Compound 2b/Compound 2b-C13/espectro.jpg]

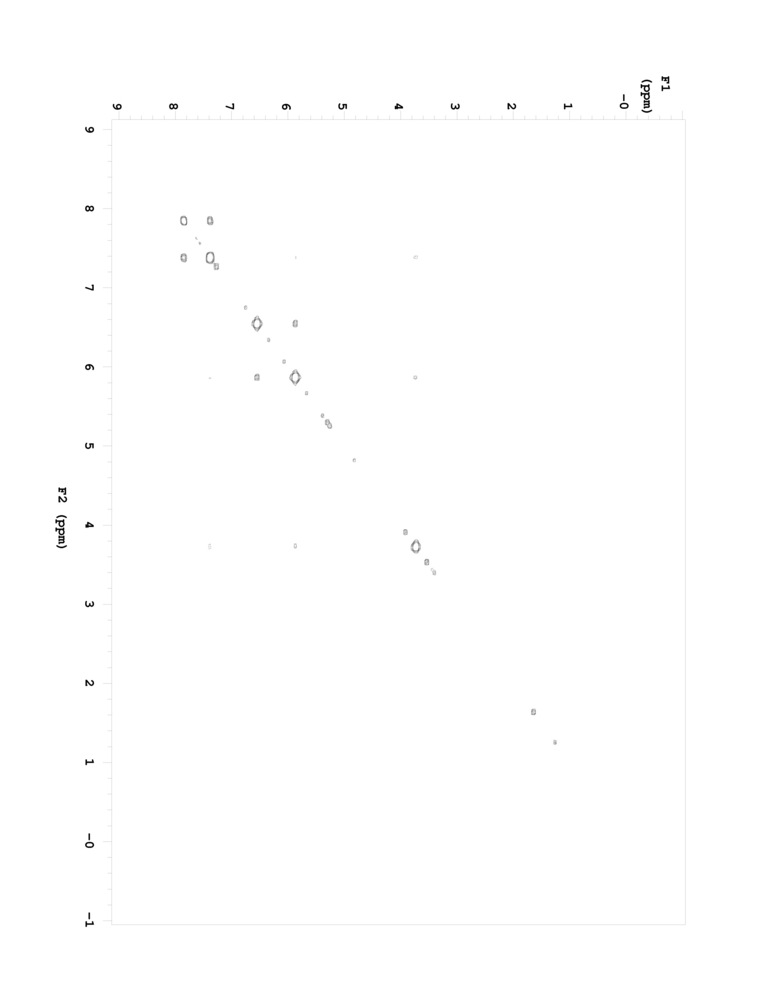

Supplement: Supplementary file 2 — jo2c02205_si_002.zip [file jo2c02205_si_002.zip › FID for publication/Compound 2b/Compound 2b-COSY/espectro.jpg]

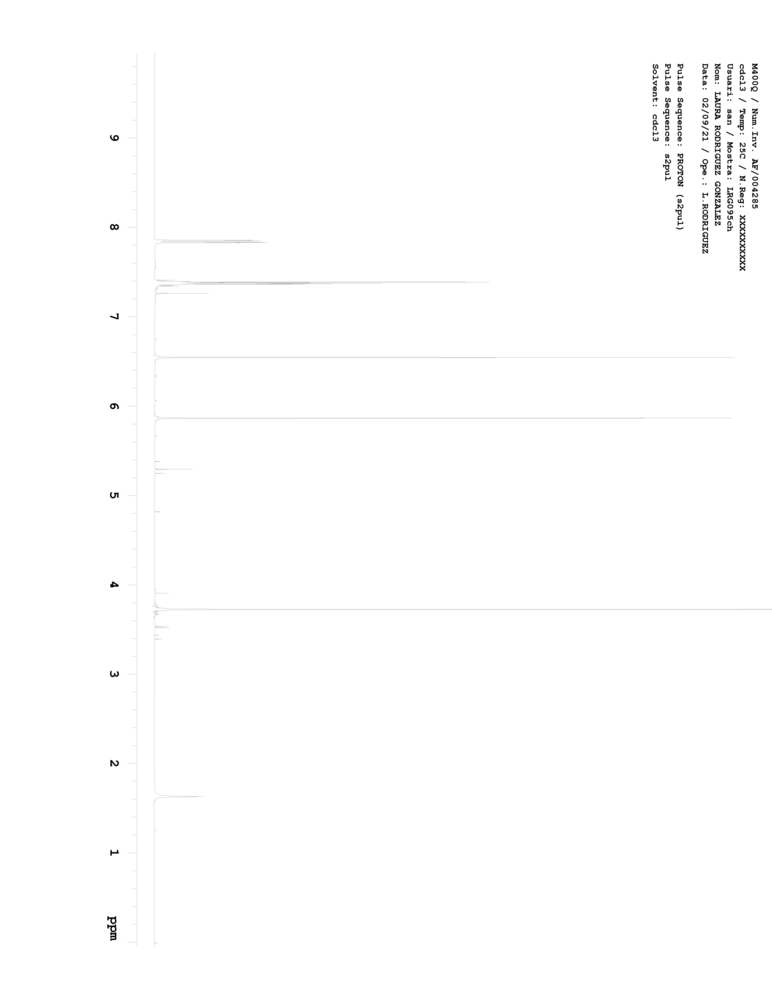

Supplement: Supplementary file 2 — jo2c02205_si_002.zip [file jo2c02205_si_002.zip › FID for publication/Compound 2b/Compound 2b-H1/espectro.jpg]

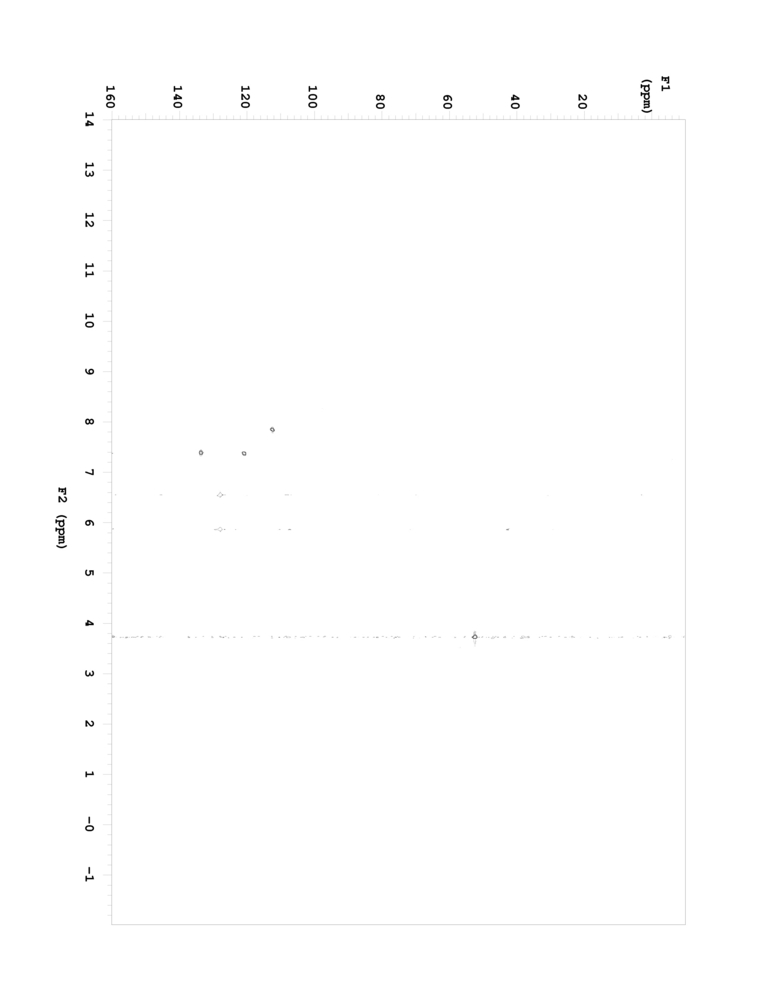

Supplement: Supplementary file 2 — jo2c02205_si_002.zip [file jo2c02205_si_002.zip › FID for publication/Compound 2b/Compound 2b-HSQC/espectro.jpg]
