# Supplementary material for: Base-Mediated Nitrophenyl Reductive Cyclization for the Synthesis of Hexahydro-2,6-methano-1-benzazocines
Source: J Org Chem. 2022 Nov 4;87(22):15693–702. doi: 10.1021/acs.joc.2c02205 (PMC9680033; doi:10.1021/acs.joc.2c02205)
Supplement: Supplementary file 2 — jo2c02205_si_002.zip [file jo2c02205_si_002.zip › FID for publication/Compound 3f/Compound 3f-COSY/pdata/1/san-1715-2021_4_1.pdf]

Register 1715/2021
Operator VICTOR MERIEL

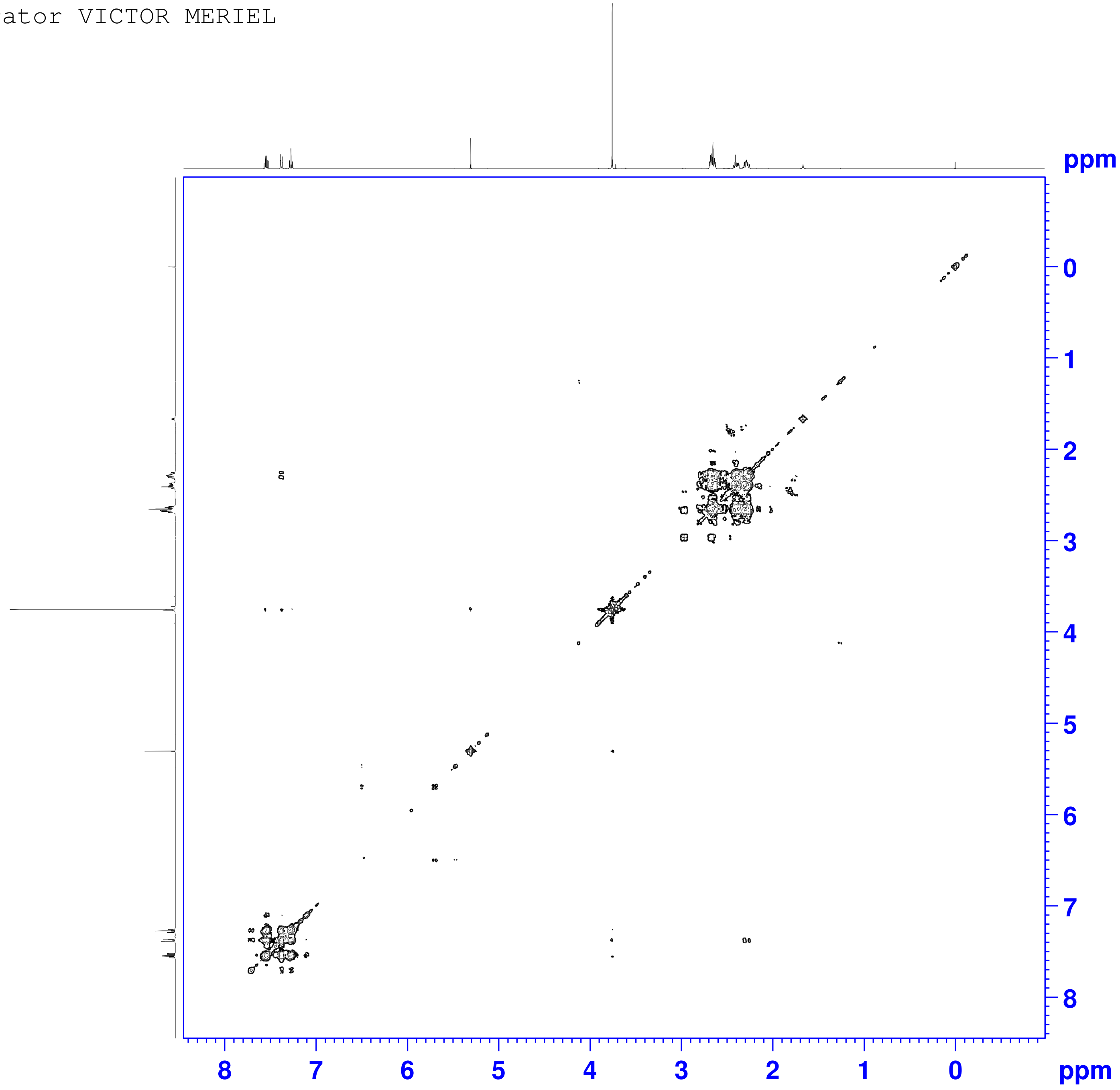

Current Data Parameters

NAME	san-1715-2021
EXPNO	4
PROCNO	1

F2 - Acquisition Parameters

Date_	20210920
Time	18.48 h
INSTRUM	Avance Neo
PROBHD	Z151574_0067 (
PULPROG	cosygpppqf
TD	2048
SOLVENT	CDCl3
NS	4
DS	16
SWH	4716.981 Hz
FIDRES	4.606427 Hz
AQ	0.2170880 sec
RG	64
DW	106.000 usec
DE	6.50 usec
TE	298.0 K
D0	0.00000300 sec
D1	0.97951949 sec
D11	0.03000000 sec
D12	0.00002000 sec
D13	0.00000400 sec
D16	0.00020000 sec
IN0	0.00021200 sec
TDav	1
SFO1	500.1318731 MHz
NUC1	1H
P0	8.00 usec
P1	8.00 usec
P17	2500.00 usec
PLW1	25.07999992 W
PLW10	1.78349996 W
GPNAM[1]	SMSQ10.100
GPZ1	10.00 %
P16	1000.00 usec

F1 - Acquisition parameters

TD	512
SFO1	500.1319 MHz
FIDRES	18.425707 Hz
SW	9.431 ppm
FnMODE	QF

F2 - Processing parameters

SI	2048
SF	500.1300050 MHz
WDW	QSINE
SSB	0
LB	0 Hz
GB	0
PC	1.40

F1 - Processing parameters

SI	1024
MC2	QF
SF	500.1300050 MHz
WDW	QSINE
SSB	0
LB	0 Hz
GB	0
