# Supplementary material for: Base-Mediated Nitrophenyl Reductive Cyclization for the Synthesis of Hexahydro-2,6-methano-1-benzazocines
Source: J Org Chem. 2022 Nov 4;87(22):15693–702. doi: 10.1021/acs.joc.2c02205 (PMC9680033; doi:10.1021/acs.joc.2c02205)
Supplement: Supplementary file 2 — jo2c02205_si_002.zip [file jo2c02205_si_002.zip › FID for publication/Compound 3f/Compound 3f-F19/pdata/1/san-1715-2021_3_1.pdf]

Register 1715/2021
Operator VICTOR MERIEL

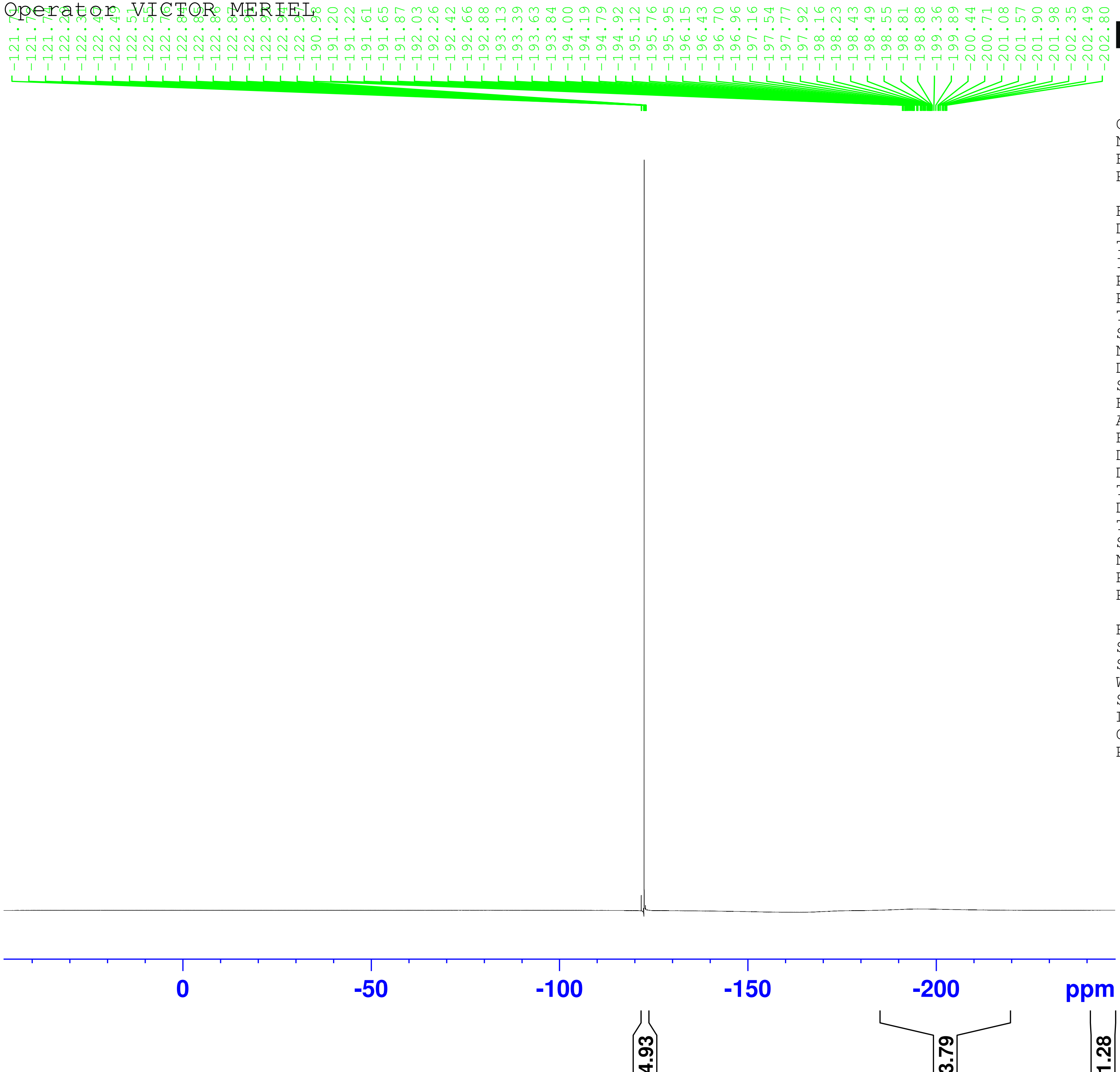

Current Data Parameters

NAME	san-1715-2021
EXPNO	3
PROCNO	1

F2 - Acquisition Parameters

Date_	20210920
Time	18.01 h
INSTRUM	Avance Neo
PROBHD	Z151574_0067 (
PULPROG	zg
TD	694444
SOLVENT	CDCl3
NS	128
DS	4
SWH	138888.891 Hz
FIDRES	0.400000 Hz
AQ	2.4999983 sec
RG	101
DW	3.600 usec
DE	6.50 usec
TE	298.0 K
D1	1.00000000 sec
TD0	1
SFO1	470.5453180 MHz
NUC1	19F
P1	10.00 usec
PLW1	62.49399948 W

F2 - Processing parameters

SI	65536
SF	470.5923772 MHz
WDW	EM
SSB	0
LB	0.50 Hz
GB	0
PC	1.00
