# Supplementary material for: Base-Mediated Nitrophenyl Reductive Cyclization for the Synthesis of Hexahydro-2,6-methano-1-benzazocines
Source: J Org Chem. 2022 Nov 4;87(22):15693–702. doi: 10.1021/acs.joc.2c02205 (PMC9680033; doi:10.1021/acs.joc.2c02205)
Supplement: Supplementary file 2 — jo2c02205_si_002.zip [file jo2c02205_si_002.zip › FID for publication/Compound 3f/Compound 3f-HSQC/pdata/1/san-1715-2021_5_1.pdf]

Register 1715/2021
Operator VICTOR MERIEL

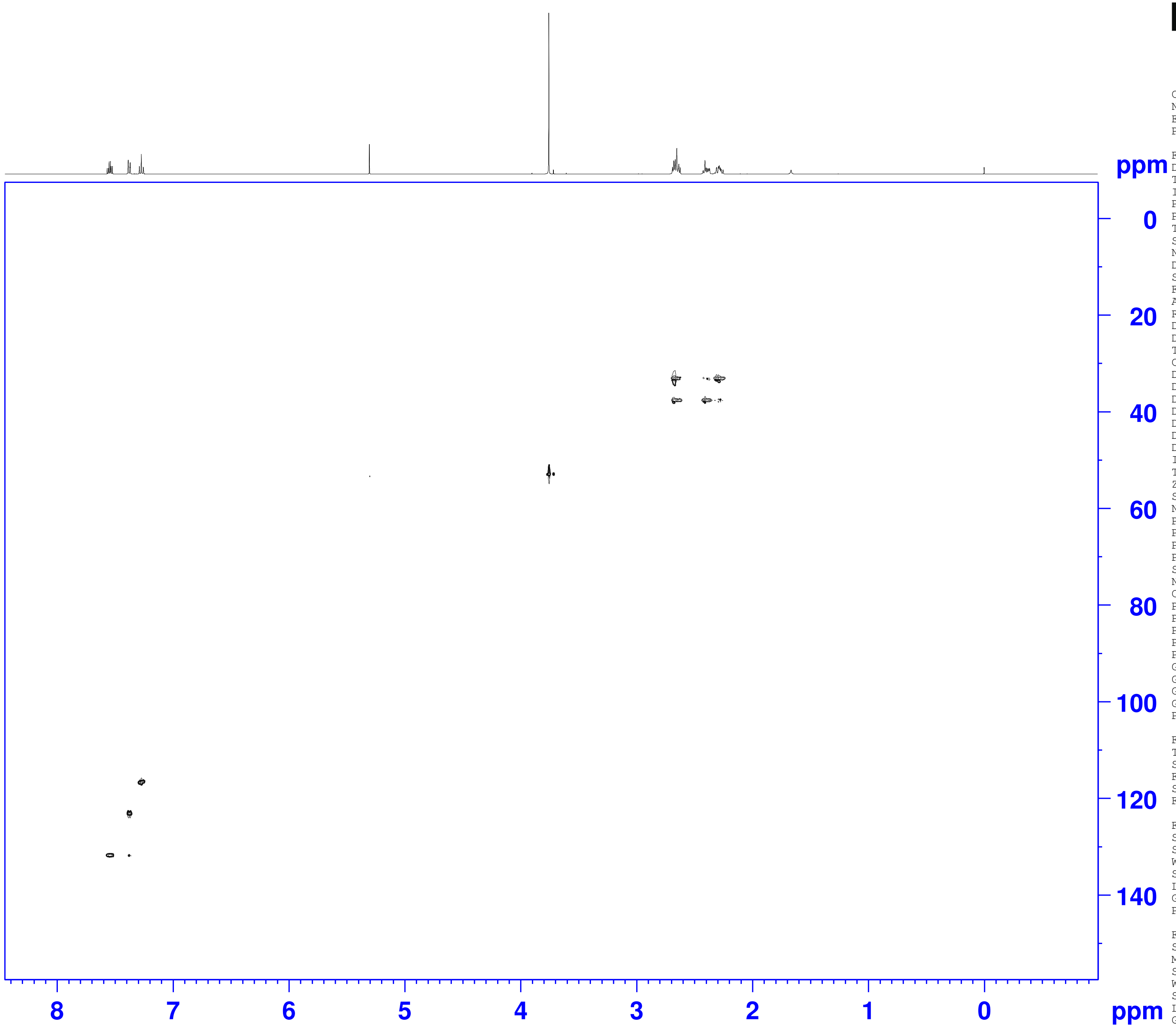

Current Data Parameters

NAME	san-1715-2021	
EXPNO	5	
PROCNO	1	

F2 - Acquisition Parameters

Date_	20210920	
Time	19.34	h
INSTRUM	Avance Neo	
PROBHD	Z151574_0067 (	
PULPROG	hsqcedetgp	
TD	2048	
SOLVENT	CDCl3	
NS	4	
DS	64	
SWH	4716.981	Hz
FIDRES	4.606427	Hz
AQ	0.2170880	sec
RG	101	
DW	106.000	usec
DE	6.50	usec
TE	298.0	K
CNST2	145.0000000	
D0	0.00000300	sec
D1	1.00000000	sec
D4	0.00172414	sec
D11	0.03000000	sec
D13	0.00000400	sec
D16	0.00020000	sec
D21	0.00345000	sec
IN0	0.00002410	sec
TDav	1	
ZGOPTNS		
SFO1	500.1318731	MHz
NUC1	1H	
P1	8.00	usec
P2	16.00	usec
P28	0	usec
PLW1	25.07999992	W
SFO2	125.7672204	MHz
NUC2	13C	
CPDPRG[2	garp4	
P3	9.00	usec
P4	18.00	usec
PCPD2	70.00	usec
PLW2	109.94000244	W
PLW12	1.81729996	W
GPNAM[1]	SMSQ10.100	
GPZ1	80.00	%
GPNAM[2]	SMSQ10.100	
GPZ2	20.10	%
P16	1000.00	usec

F1 - Acquisition parameters

TD	512	
SFO1	125.7672	MHz
FIDRES	81.063553	Hz
SW	165.005	ppm
FnMODE	Echo-Antiecho	

F2 - Processing parameters

SI	2048	
SF	500.1300050	MHz
WDW	QSINE	
SSB	2	
LB	0	Hz
GB	0	
PC	1.40	

F1 - Processing parameters

SI	1024	
MC2	echo-antiecho	
SF	125.7577885	MHz
WDW	QSINE	
SSB	2	
LB	0	Hz
GB	0	
