# Supplementary material for: Base-Mediated Nitrophenyl Reductive Cyclization for the Synthesis of Hexahydro-2,6-methano-1-benzazocines
Source: J Org Chem. 2022 Nov 4;87(22):15693–702. doi: 10.1021/acs.joc.2c02205 (PMC9680033; doi:10.1021/acs.joc.2c02205)
Supplement: Supplementary file 2 — jo2c02205_si_002.zip [file jo2c02205_si_002.zip › FID for publication/Compound 4f/Compound 4f-COSY/pdata/1/san-1732-2021_3_1.pdf]

Register 1732/2021
Operator ALBERT GALLEN

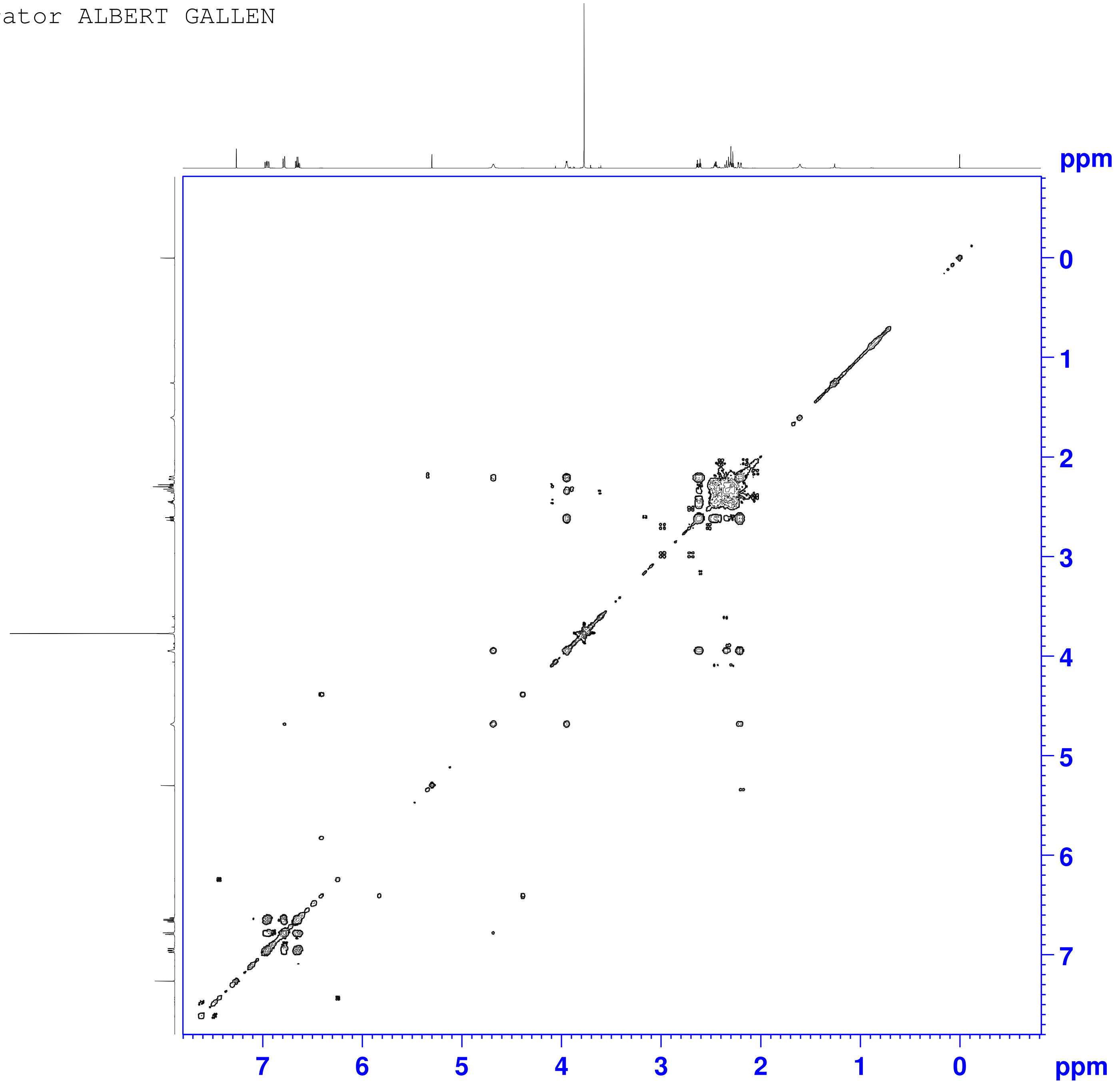

BRUKER

Current Data Parameters
NAME san-1732-2021
EXPNO 3
PROCNO 1

F2 - Acquisition Parameters
Date_ 20210922
Time 18.04 h
INSTRUM Avance Neo
PROBHD Z151574_0067 (
PULPROG cosygpppqf
TD 2048
SOLVENT CDCl3
NS 4
DS 16
SWH 4310.345 Hz
FIDRES 4.209321 Hz
AQ 0.2375680 sec
RG 101
DW 116.000 usec
DE 6.50 usec
TE 298.0 K
D0 0.00000300 sec
D1 0.95903963 sec
D11 0.03000000 sec
D12 0.00002000 sec
D13 0.00000400 sec
D16 0.00020000 sec
IN0 0.00023200 sec
TDav 1
SFO1 500.1317568 MHz
NUC1 1H
P0 8.00 usec
P1 8.00 usec
P17 2500.00 usec
PLW1 25.07999992 W
PLW10 1.78349996 W
GPNAM[1] SMSQ10.100
GPZ1 10.00 %
P16 1000.00 usec

F1 - Acquisition parameters
TD 512
SFO1 500.1318 MHz
FIDRES 16.837284 Hz
SW 8.618 ppm
FnMODE QF

F2 - Processing parameters
SI 2048
SF 500.1300104 MHz
WDW QSINE
SSB 0
LB 0 Hz
GB 0
PC 1.40

F1 - Processing parameters
SI 1024
MC2 QF
SF 500.1300104 MHz
WDW QSINE
SSB 0
LB 0 Hz
GB 0
