# Supplementary material for: Base-Mediated Nitrophenyl Reductive Cyclization for the Synthesis of Hexahydro-2,6-methano-1-benzazocines
Source: J Org Chem. 2022 Nov 4;87(22):15693–702. doi: 10.1021/acs.joc.2c02205 (PMC9680033; doi:10.1021/acs.joc.2c02205)
Supplement: Supplementary file 2 — jo2c02205_si_002.zip [file jo2c02205_si_002.zip › FID for publication/Compound 4f/Compound 4f-F19/pdata/1/san-1732-2021_5_1.pdf]

Register 1732/2021
Operator ALBERT GALLEN

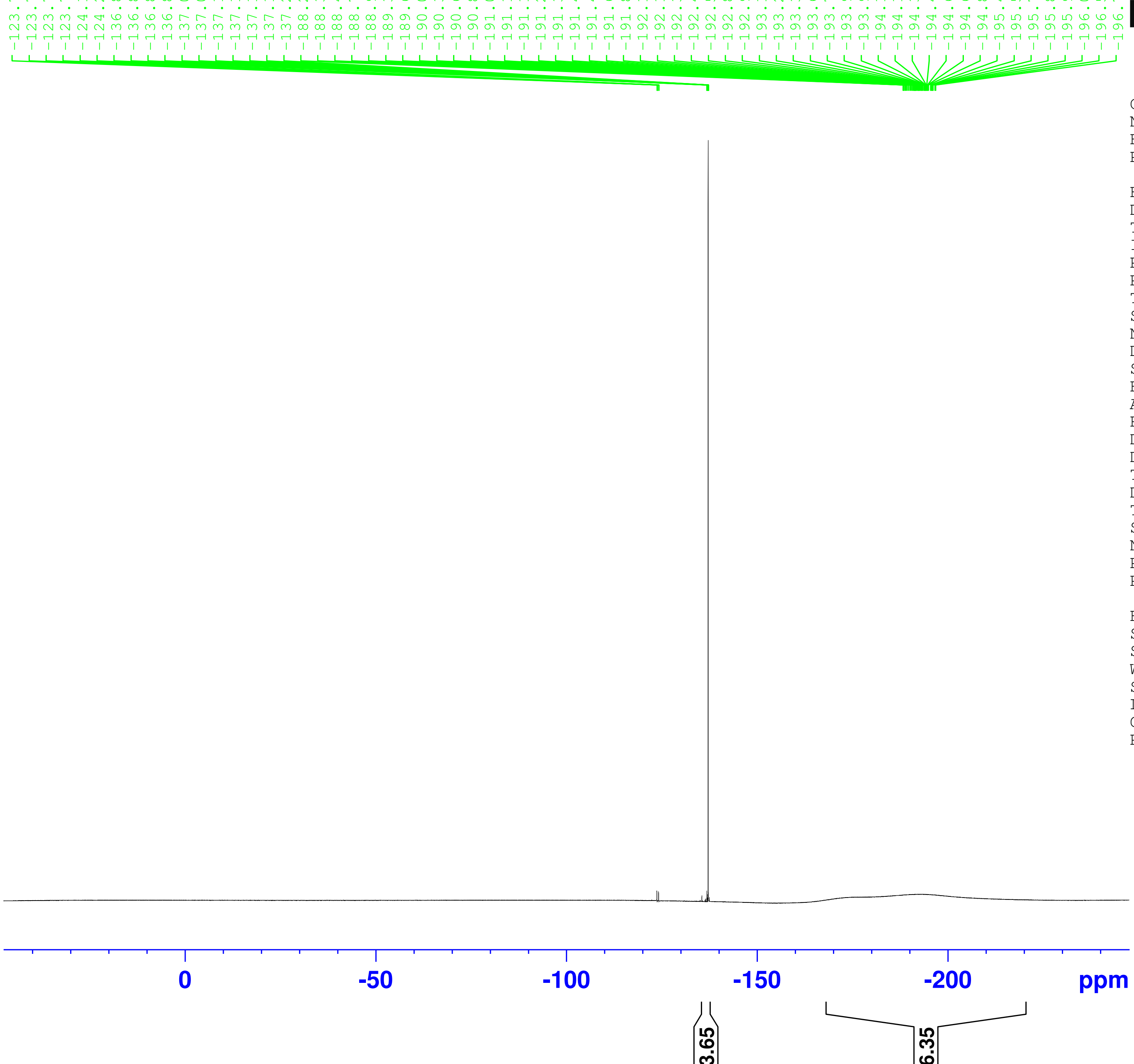

BRUKER

Current Data Parameters
NAME san-1732-2021
EXPNO 5
PROCNO 1

F2 - Acquisition Parameters
Date_ 20210922
Time 18.56 h
INSTRUM Avance Neo
PROBHD Z151574_0067 (
PULPROG zg
TD 694444
SOLVENT CDCl3
NS 128
DS 4
SWH 138888.891 Hz
FIDRES 0.400000 Hz
AQ 2.4999983 sec
RG 101
DW 3.600 usec
DE 6.50 usec
TE 298.0 K
D1 1.00000000 sec
TD0 1
SFO1 470.5453180 MHz
NUC1 19F
P1 10.00 usec
PLW1 62.49399948 W

F2 - Processing parameters
SI 65536
SF 470.5923772 MHz
WDW EM
SSB 0
LB 0.50 Hz
GB 0
PC 1.00
