# Supplementary material for: Base-Mediated Nitrophenyl Reductive Cyclization for the Synthesis of Hexahydro-2,6-methano-1-benzazocines
Source: J Org Chem. 2022 Nov 4;87(22):15693–702. doi: 10.1021/acs.joc.2c02205 (PMC9680033; doi:10.1021/acs.joc.2c02205)
Supplement: Supplementary file 2 — jo2c02205_si_002.zip [file jo2c02205_si_002.zip › FID for publication/Compound 4f/Compound 4f-HSQC/pdata/1/san-1732-2021_4_1.pdf]

Register 1732/2021
Operator ALBERT GALLEN

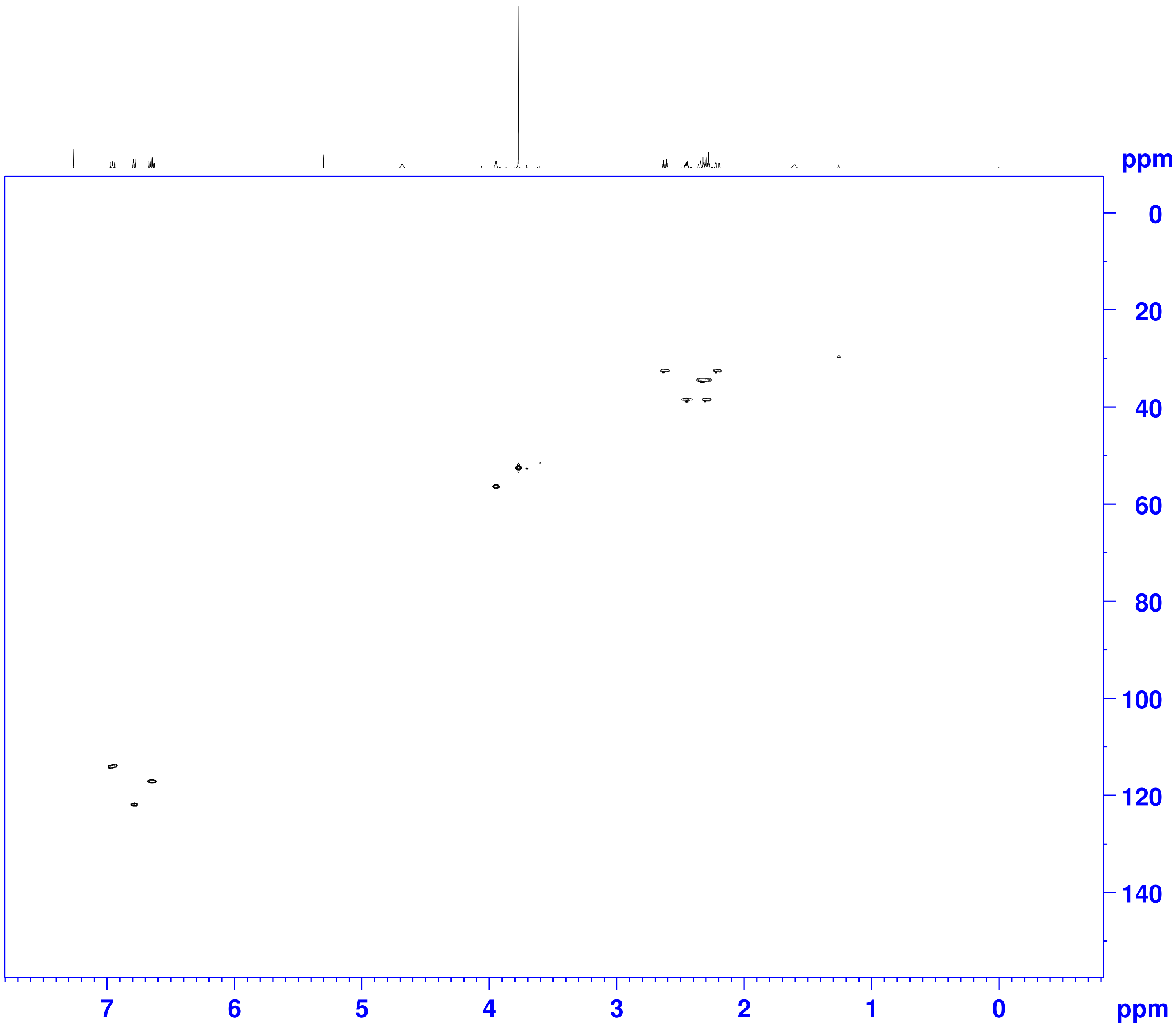


Current Data Parameters
NAME san-1732-2021
EXPNO 4
PROCNO 1

F2 - Acquisition Parameters
Date_ 20210922
Time 18.46 h
INSTRUM Avance Neo
PROBHD Z151574_0067 (
PULPROG hsqcedetgp
TD 1024
SOLVENT CDCl3
NS 4
DS 64
SWH 4310.345 Hz
FIDRES 8.418642 Hz
AQ 0.1187840 sec
RG 101
DW 116.000 usec
DE 6.50 usec
TE 298.0 K
CNST2 145.0000000
D0 0.00000300 sec
D1 1.00000000 sec
D4 0.00172414 sec
D11 0.03000000 sec
D13 0.00000400 sec
D16 0.00020000 sec
D21 0.00345000 sec
IN0 0.00002410 sec
TDav 1
ZGOPTNS
SFO1 500.1317568 MHz
NUC1 1H
P1 8.00 usec
P2 16.00 usec
P28 0 usec
PLW1 25.07999992 W
SFO2 125.7672204 MHz
NUC2 13C
CPDPRG[2 garp4
P3 9.00 usec
P4 18.00 usec
PCPD2 70.00 usec
PLW2 109.94000244 W
PLW12 1.81729996 W
GPNAM[1] SMSQ10.100
GPZ1 80.00 %
GPNAM[2] SMSQ10.100
GPZ2 20.10 %
P16 1000.00 usec

F1 - Acquisition parameters
TD 512
SFO1 125.7672 MHz
FIDRES 81.063553 Hz
SW 165.005 ppm
FnMODE Echo-Antiecho

F2 - Processing parameters
SI 1024
SF 500.1300104 MHz
WDW QSINE
SSB 2
LB 0 Hz
GB 0
PC 1.40

F1 - Processing parameters
SI 1024
MC2 echo-antiecho
SF 125.7577885 MHz
WDW QSINE
SSB 2
LB 0 Hz
GB 0
