# Supplementary material for: Base-Mediated Nitrophenyl Reductive Cyclization for the Synthesis of Hexahydro-2,6-methano-1-benzazocines
Source: J Org Chem. 2022 Nov 4;87(22):15693–702. doi: 10.1021/acs.joc.2c02205 (PMC9680033; doi:10.1021/acs.joc.2c02205)
Supplement: Supplementary file 2 — jo2c02205_si_002.zip [file jo2c02205_si_002.zip › FID for publication/structure file_BRADSHAW.docx]

The spectrometers used to collect the data are:

- **Varian VNMRS 400 MHz**
- **Varian Mercury 400 MHz**
- **Bruker 400 MHz**
- **Bruker 500 MHz**

The acquisition software used is VnmrJ.

The processing program used to analyze the data is MestreNova.

**Structure file**

**Dimethyl (2-Nitrophenyl)malonate (1a)**

Compound identifier (MolFile):

SMILES: O=C(C(C(OC)=O)C1=CC=CC=C1[N+]([O-])=O)OC

MOL Text:

Actelion Java MolfileCreator 1.0

18 18 0 0 0 0 0 0 0 0999 V2000

3.4641	-1.5000	-0.0000 O	0 0 0 0 0 0 0 0 0 0 0 0

3.4641	-0.5000	-0.0000 C	0 0 0 0 0 0 0 0 0 0 0 0

4.3301	-0.0000	-0.0000 O	0 0 0 0 0 0 0 0 0 0 0 0

5.1962	-0.5000	-0.0000 C	0 0 0 0 0 0 0 0 0 0 0 0

2.5981	-0.0000	-0.0000 C	0 0 0 0 0 0 0 0 0 0 0 0

2.5981	1.0000	-0.0000 C	0 0 0 0 0 0 0 0 0 0 0 0

3.4641	1.5000	-0.0000 C	0 0 0 0 0 0 0 0 0 0 0 0

3.4641	2.5000	-0.0000 C	0 0 0 0 0 0 0 0 0 0 0 0

2.5981	3.0000	-0.0000 C	0 0 0 0 0 0 0 0 0 0 0 0

1.7321	2.5000	-0.0000 C	0 0 0 0 0 0 0 0 0 0 0 0

1.7321	1.5000	-0.0000 C	0 0 0 0 0 0 0 0 0 0 0 0

0.8660	1.0000	-0.0000 N	0 0 0 0 0 0 0 0 0 0 0 0

-0.0000	1.5000	-0.0000 O	0 0 0 0 0 0 0 0 0 0 0 0

0.8721	-0.2105	-0.0000 O	0 0 0 0 0 0 0 0 0 0 0 0

1.7321	-0.5000	-0.0000 C	0 0 0 0 0 0 0 0 0 0 0 0

0.6186	-0.0012	-0.0000 O	0 0 0 0 0 0 0 0 0 0 0 0

0.0000	-0.5000	-0.0000 C	0 0 0 0 0 0 0 0 0 0 0 0

1.7321	-1.5000	-0.0000 O	0 0 0 0 0 0 0 0 0 0 0 0

2 1 2 0 0 0 0

3 2 1 0 0 0 0

4 3 1 0 0 0 0

5 2 1 0 0 0 0

6 5 1 0 0 0 0

7 6 2 0 0 0 0

8 7 1 0 0 0 0

9 8 2 0 0 0 0

10 9 1 0 0 0 0

11 10 2 0 0 0 0

11 6 1 0 0 0 0

12 11 1 0 0 0 0

13 12 1 0 0 0 0

14 12 2 0 0 0 0

15 5 1 0 0 0 0

16 15 1 0 0 0 0

17 16 1 0 0 0 0

18 15 2 0 0 0 0

M CHG 2 12	1 13 -1

M END

**Dimethyl-2-(4-Fluoro-2-nitrophenyl)malonate (1b)**

Compound identifier (MolFile):

SMILES: FC1=CC=C(C([N+]([O-])=O)=C1)C(C(OC)=O)C(OC)=O

MOL Text:

Actelion Java MolfileCreator 1.0

19 19 0 0 0 0 0 0 0 0999 V2000

3.9729	6.1167	-0.0000 F	0 0 0 0 0 0 0 0 0 0 0 0

3.9729	4.5875	-0.0000 C	0 0 0 0 0 0 0 0 0 0 0 0

2.6486	3.8229	-0.0000 C	0 0 0 0 0 0 0 0 0 0 0 0

2.6486	2.2938	-0.0000 C	0 0 0 0 0 0 0 0 0 0 0 0

3.9729	1.5292	-0.0000 C	0 0 0 0 0 0 0 0 0 0 0 0

5.2972	2.2938	-0.0000 C	0 0 0 0 0 0 0 0 0 0 0 0

6.6215	1.5292	-0.0000 N	0 0 0 0 0 0 0 0 0 0 0 0

6.6215	0.3823	-0.0000 O	0 0 0 0 0 0 0 0 0 0 0 0

7.9458	2.2938	-0.0000 O	0 0 0 0 0 0 0 0 0 0 0 0

5.2972	3.8229	-0.0000 C	0 0 0 0 0 0 0 0 0 0 0 0

3.9729	-0.0000	-0.0000 C	0 0 0 0 0 0 0 0 0 0 0 0

5.2972	-0.7646	-0.0000 C	0 0 0 0 0 0 0 0 0 0 0 0

6.6215	-0.1911	-0.0000 O	0 0 0 0 0 0 0 0 0 0 0 0

7.9458	-0.7646	-0.0000 C	0 0 0 0 0 0 0 0 0 0 0 0

5.2972	-2.2938	-0.0000 O	0 0 0 0 0 0 0 0 0 0 0 0

2.6486	-0.7646	-0.0000 C	0 0 0 0 0 0 0 0 0 0 0 0

1.3243	-0.0000	-0.0000 O	0 0 0 0 0 0 0 0 0 0 0 0

0.0000	-0.7646	-0.0000 C	0 0 0 0 0 0 0 0 0 0 0 0

2.6486	-2.2938	-0.0000 O	0 0 0 0 0 0 0 0 0 0 0 0

2 1 1 0 0 0 0

3 2 2 0 0 0 0

4 3 1 0 0 0 0

5 4 2 0 0 0 0

6 5 1 0 0 0 0

7 6 1 0 0 0 0

8 7 1 0 0 0 0

9 7 2 0 0 0 0

10 6 2 0 0 0 0

10 2 1 0 0 0 0

11 5 1 0 0 0 0

12 11 1 0 0 0 0

13 12 1 0 0 0 0

14 13 1 0 0 0 0

15 12 2 0 0 0 0

16 11 1 0 0 0 0

17 16 1 0 0 0 0

18 17 1 0 0 0 0

19 16 2 0 0 0 0

M CHG 2	7	1	8 -1

M END

**Dimethyl-2-(4-Chloro-2-nitrophenyl)malonate (1c)**

Compound identifier (MolFile):

SMILES: ClC1=CC=C(C(C(OC)=O)C(OC)=O)C([N+]([O-])=O)=C1

MOL Text:

Actelion Java MolfileCreator 1.0

19 19 0 0 0 0 0 0 0 0999 V2000

3.9729	6.1167	-0.0000 Cl 0 0 0 0 0 0 0 0 0 0 0 0

3.9729	4.5875	-0.0000 C	0 0 0 0 0 0 0 0 0 0 0 0

2.6486	3.8229	-0.0000 C	0 0 0 0 0 0 0 0 0 0 0 0

2.6486	2.2938	-0.0000 C	0 0 0 0 0 0 0 0 0 0 0 0

3.9729	1.5292	-0.0000 C	0 0 0 0 0 0 0 0 0 0 0 0

3.9729	-0.0000	-0.0000 C	0 0 0 0 0 0 0 0 0 0 0 0

5.2972	-0.7646	-0.0000 C	0 0 0 0 0 0 0 0 0 0 0 0

6.6215	-0.1911	-0.0000 O	0 0 0 0 0 0 0 0 0 0 0 0

7.9458	-0.7646	-0.0000 C	0 0 0 0 0 0 0 0 0 0 0 0

5.2972	-2.2938	-0.0000 O	0 0 0 0 0 0 0 0 0 0 0 0

2.6486	-0.7646	-0.0000 C	0 0 0 0 0 0 0 0 0 0 0 0

1.3243	-0.0000	-0.0000 O	0 0 0 0 0 0 0 0 0 0 0 0

0.0000	-0.7646	-0.0000 C	0 0 0 0 0 0 0 0 0 0 0 0

2.6486	-2.2938	-0.0000 O	0 0 0 0 0 0 0 0 0 0 0 0

5.2972	2.2938	-0.0000 C	0 0 0 0 0 0 0 0 0 0 0 0

6.6215	1.5292	-0.0000 N	0 0 0 0 0 0 0 0 0 0 0 0

6.6215	0.3823	-0.0000 O	0 0 0 0 0 0 0 0 0 0 0 0

7.9458	2.2938	-0.0000 O	0 0 0 0 0 0 0 0 0 0 0 0

5.2972	3.8229	-0.0000 C	0 0 0 0 0 0 0 0 0 0 0 0

2 1 1 0 0 0 0

3 2 2 0 0 0 0

4 3 1 0 0 0 0

5 4 2 0 0 0 0

6 5 1 0 0 0 0

7 6 1 0 0 0 0

8 7 1 0 0 0 0

9 8 1 0 0 0 0

10 7 2 0 0 0 0

11 6 1 0 0 0 0

12 11 1 0 0 0 0

13 12 1 0 0 0 0

14 11 2 0 0 0 0

15 5 1 0 0 0 0

16 15 1 0 0 0 0

17 16 1 0 0 0 0

18 16 2 0 0 0 0

19 15 2 0 0 0 0

19 2 1 0 0 0 0

M CHG 2 16	1 17 -1

M END

**Dimethyl-2-(4-Bromo-2-nitrophenyl)malonate (1d)**

Compound identifier (MolFile):

SMILES: BrC1=CC=C(C(C(OC)=O)C(OC)=O)C([N+]([O-])=O)=C1

MOL Text:

Actelion Java MolfileCreator 1.0

19 19 0 0 0 0 0 0 0 0999 V2000

3.9729	6.1167	-0.0000 Br 0 0 0 0 0 0 0 0 0 0 0 0

3.9729	4.5875	-0.0000 C	0 0 0 0 0 0 0 0 0 0 0 0

2.6486	3.8229	-0.0000 C	0 0 0 0 0 0 0 0 0 0 0 0

2.6486	2.2938	-0.0000 C	0 0 0 0 0 0 0 0 0 0 0 0

3.9729	1.5292	-0.0000 C	0 0 0 0 0 0 0 0 0 0 0 0

3.9729	-0.0000	-0.0000 C	0 0 0 0 0 0 0 0 0 0 0 0

5.2972	-0.7646	-0.0000 C	0 0 0 0 0 0 0 0 0 0 0 0

6.6215	-0.1911	-0.0000 O	0 0 0 0 0 0 0 0 0 0 0 0

7.9458	-0.7646	-0.0000 C	0 0 0 0 0 0 0 0 0 0 0 0

5.2972	-2.2938	-0.0000 O	0 0 0 0 0 0 0 0 0 0 0 0

2.6486	-0.7646	-0.0000 C	0 0 0 0 0 0 0 0 0 0 0 0

1.3243	-0.0000	-0.0000 O	0 0 0 0 0 0 0 0 0 0 0 0

0.0000	-0.7646	-0.0000 C	0 0 0 0 0 0 0 0 0 0 0 0

2.6486	-2.2938	-0.0000 O	0 0 0 0 0 0 0 0 0 0 0 0

5.2972	2.2938	-0.0000 C	0 0 0 0 0 0 0 0 0 0 0 0

6.6215	1.5292	-0.0000 N	0 0 0 0 0 0 0 0 0 0 0 0

6.6215	0.3823	-0.0000 O	0 0 0 0 0 0 0 0 0 0 0 0

7.9458	2.2938	-0.0000 O	0 0 0 0 0 0 0 0 0 0 0 0

5.2972	3.8229	-0.0000 C	0 0 0 0 0 0 0 0 0 0 0 0

2 1 1 0 0 0 0

3 2 2 0 0 0 0

4 3 1 0 0 0 0

5 4 2 0 0 0 0

6 5 1 0 0 0 0

7 6 1 0 0 0 0

8 7 1 0 0 0 0

9 8 1 0 0 0 0

10 7 2 0 0 0 0

11 6 1 0 0 0 0

12 11 1 0 0 0 0

13 12 1 0 0 0 0

14 11 2 0 0 0 0

15 5 1 0 0 0 0

16 15 1 0 0 0 0

17 16 1 0 0 0 0

18 16 2 0 0 0 0

19 15 2 0 0 0 0

19 2 1 0 0 0 0

M CHG 2 16	1 17 -1

M END

**Dimethyl 2-(5-Fluoro-2-nitrophenyl)malonate (1e)**

Compound identifier (MolFile):

SMILES: FC1=CC=C([N+]([O-])=O)C(C(C(OC)=O)C(OC)=O)=C1

MOL Text:

Actelion Java MolfileCreator 1.0

19 19 0 0 0 0 0 0 0 0999 V2000

4.3301	3.0000	-0.0000 F	0 0 0 0 0 0 0 0 0 0 0 0

3.4641	2.5000	-0.0000 C	0 0 0 0 0 0 0 0 0 0 0 0

2.5981	3.0000	-0.0000 C	0 0 0 0 0 0 0 0 0 0 0 0

1.7321	2.5000	-0.0000 C	0 0 0 0 0 0 0 0 0 0 0 0

1.7321	1.5000	-0.0000 C	0 0 0 0 0 0 0 0 0 0 0 0

0.8660	1.0000	-0.0000 N	0 0 0 0 0 0 0 0 0 0 0 0

-0.0000	1.5000	-0.0000 O	0 0 0 0 0 0 0 0 0 0 0 0

0.8284	-0.2026	-0.0000 O	0 0 0 0 0 0 0 0 0 0 0 0

2.5981	1.0000	-0.0000 C	0 0 0 0 0 0 0 0 0 0 0 0

2.5981	-0.0000	-0.0000 C	0 0 0 0 0 0 0 0 0 0 0 0

3.4641	-0.5000	-0.0000 C	0 0 0 0 0 0 0 0 0 0 0 0

4.3301	-0.0000	-0.0000 O	0 0 0 0 0 0 0 0 0 0 0 0

5.1962	-0.5000	-0.0000 C	0 0 0 0 0 0 0 0 0 0 0 0

3.4641	-1.5000	-0.0000 O	0 0 0 0 0 0 0 0 0 0 0 0

1.7321	-0.5000	-0.0000 C	0 0 0 0 0 0 0 0 0 0 0 0

1.1003	-0.0073	-0.0000 O	0 0 0 0 0 0 0 0 0 0 0 0

0.0000	-0.5000	-0.0000 C	0 0 0 0 0 0 0 0 0 0 0 0

1.7321	-1.5000	-0.0000 O	0 0 0 0 0 0 0 0 0 0 0 0

3.4641	1.5000	-0.0000 C	0 0 0 0 0 0 0 0 0 0 0 0

2 1 1 0 0 0 0

3 2 2 0 0 0 0

4 3 1 0 0 0 0

5 4 2 0 0 0 0

6 5 1 0 0 0 0

7 6 1 0 0 0 0

8 6 2 0 0 0 0

9 5 1 0 0 0 0

10 9 1 0 0 0 0

11 10 1 0 0 0 0

12 11 1 0 0 0 0

13 12 1 0 0 0 0

14 11 2 0 0 0 0

15 10 1 0 0 0 0

16 15 1 0 0 0 0

17 16 1 0 0 0 0

18 15 2 0 0 0 0

19 9 2 0 0 0 0

19 2 1 0 0 0 0

M CHG 2	6	1	7 -1

M END

**Dimethyl-2-(3-Fluoro-2-nitrophenyl)malonate (1f)**

Compound identifier (MolFile):

SMILES: FC1=C([N+]([O-])=O)C(C(C(OC)=O)C(OC)=O)=CC=C1

MOL Text:

Actelion Java MolfileCreator 1.0

19 19 0 0 0 0 0 0 0 0999 V2000

1.3127	4.5474	-0.0000 F	0 0 0 0 0 0 0 0 0 0 0 0

2.6254	3.7895	-0.0000 C	0 0 0 0 0 0 0 0 0 0 0 0

2.6254	2.2737	-0.0000 C	0 0 0 0 0 0 0 0 0 0 0 0

1.3127	1.5158	-0.0000 N	0 0 0 0 0 0 0 0 0 0 0 0

0.0000	2.2737	-0.0000 O	0 0 0 0 0 0 0 0 0 0 0 0

0.9669	0.1549	-0.0000 O	0 0 0 0 0 0 0 0 0 0 0 0

3.9381	1.5158	-0.0000 C	0 0 0 0 0 0 0 0 0 0 0 0

3.9381	-0.0000	-0.0000 C	0 0 0 0 0 0 0 0 0 0 0 0

5.2508	-0.7579	-0.0000 C	0 0 0 0 0 0 0 0 0 0 0 0

6.5635	-0.0000	-0.0000 O	0 0 0 0 0 0 0 0 0 0 0 0

7.8763	-0.7579	-0.0000 C	0 0 0 0 0 0 0 0 0 0 0 0

5.2508	-2.2737	-0.0000 O	0 0 0 0 0 0 0 0 0 0 0 0

2.6254	-0.7579	-0.0000 C	0 0 0 0 0 0 0 0 0 0 0 0

2.6254	-2.2737	-0.0000 O	0 0 0 0 0 0 0 0 0 0 0 0

1.3127	-3.0316	-0.0000 C	0 0 0 0 0 0 0 0 0 0 0 0

1.4856	-0.0774	-0.0000 O	0 0 0 0 0 0 0 0 0 0 0 0

5.2508	2.2737	-0.0000 C	0 0 0 0 0 0 0 0 0 0 0 0

5.2508	3.7895	-0.0000 C	0 0 0 0 0 0 0 0 0 0 0 0

3.9381	4.5474	-0.0000 C	0 0 0 0 0 0 0 0 0 0 0 0

2 1 1 0 0 0 0

3 2 2 0 0 0 0

4 3 1 0 0 0 0

5 4 1 0 0 0 0

6 4 2 0 0 0 0

7 3 1 0 0 0 0

8 7 1 0 0 0 0

9 8 1 0 0 0 0

10 9 1 0 0 0 0

11 10 1 0 0 0 0

12 9 2 0 0 0 0

13 8 1 0 0 0 0

14 13 1 0 0 0 0

15 14 1 0 0 0 0

16 13 2 0 0 0 0

17 7 2 0 0 0 0

18 17 1 0 0 0 0

19 18 2 0 0 0 0

19 2 1 0 0 0 0

M CHG 2	4	1	5 -1

M END

**Dimethyl-2-(4-Methoxy-2-nitrophenyl)malonate (1g)**

Compound identifier (MolFile):

SMILES: O=C(C(C(OC)=O)C1=CC=C(OC)C=C1[N+]([O-])=O)OC

MOL Text:

Actelion Java MolfileCreator 1.0

20 20 0 0 0 0 0 0 0 0999 V2000

1.7321	-1.5000	-0.0000 O	0 0 0 0 0 0 0 0 0 0 0 0

1.7321	-0.5000	-0.0000 C	0 0 0 0 0 0 0 0 0 0 0 0

2.5981	-0.0000	-0.0000 C	0 0 0 0 0 0 0 0 0 0 0 0

3.4641	-0.5000	-0.0000 C	0 0 0 0 0 0 0 0 0 0 0 0

4.3301	-0.0000	-0.0000 O	0 0 0 0 0 0 0 0 0 0 0 0

5.1962	-0.5000	-0.0000 C	0 0 0 0 0 0 0 0 0 0 0 0

3.4641	-1.5000	-0.0000 O	0 0 0 0 0 0 0 0 0 0 0 0

2.5981	1.0000	-0.0000 C	0 0 0 0 0 0 0 0 0 0 0 0

3.4641	1.5000	-0.0000 C	0 0 0 0 0 0 0 0 0 0 0 0

3.4641	2.5000	-0.0000 C	0 0 0 0 0 0 0 0 0 0 0 0

2.5981	3.0000	-0.0000 C	0 0 0 0 0 0 0 0 0 0 0 0

2.5981	4.0000	-0.0000 O	0 0 0 0 0 0 0 0 0 0 0 0

1.7321	4.5000	-0.0000 C	0 0 0 0 0 0 0 0 0 0 0 0

1.7321	2.5000	-0.0000 C	0 0 0 0 0 0 0 0 0 0 0 0

1.7321	1.5000	-0.0000 C	0 0 0 0 0 0 0 0 0 0 0 0

0.8660	1.0000	-0.0000 N	0 0 0 0 0 0 0 0 0 0 0 0

0.0000	1.5000	-0.0000 O	0 0 0 0 0 0 0 0 0 0 0 0

0.8613	-0.1813	-0.0000 O	0 0 0 0 0 0 0 0 0 0 0 0

1.1140	-0.0010	-0.0000 O	0 0 0 0 0 0 0 0 0 0 0 0

0.0000	-0.5000	-0.0000 C	0 0 0 0 0 0 0 0 0 0 0 0

2 1 2 0 0 0 0

3 2 1 0 0 0 0

4 3 1 0 0 0 0

5 4 1 0 0 0 0

6 5 1 0 0 0 0

7 4 2 0 0 0 0

8 3 1 0 0 0 0

9 8 2 0 0 0 0

10 9 1 0 0 0 0

11 10 2 0 0 0 0

12 11 1 0 0 0 0

13 12 1 0 0 0 0

14 11 1 0 0 0 0

15 14 2 0 0 0 0

15 8 1 0 0 0 0

16 15 1 0 0 0 0

17 16 1 0 0 0 0

18 16 2 0 0 0 0

19 2 1 0 0 0 0

20 19 1 0 0 0 0

M CHG 2 16	1 17 -1

M END

**Methyl 2-(2-Nitrophenyl)acrylate (2a)**

Compound identifier (MolFile):

SMILES: C=C(C(OC)=O)C1=CC=CC=C1[N+]([O-])=O

MOL Text:

Actelion Java MolfileCreator 1.0

15 15 0 0 0 0 0 0 0 0999 V2000

1.7321	-0.5000	-0.0000 C	0 0 0 0 0 0 0 0 0 0 0 0

2.5981	-0.0000	-0.0000 C	0 0 0 0 0 0 0 0 0 0 0 0

3.4641	-0.5000	-0.0000 C	0 0 0 0 0 0 0 0 0 0 0 0

4.3301	0.0000	-0.0000 O	0 0 0 0 0 0 0 0 0 0 0 0

5.1962	-0.5000	-0.0000 C	0 0 0 0 0 0 0 0 0 0 0 0

3.4641	-1.5000	-0.0000 O	0 0 0 0 0 0 0 0 0 0 0 0

2.5981	1.0000	-0.0000 C	0 0 0 0 0 0 0 0 0 0 0 0

3.4641	1.5000	-0.0000 C	0 0 0 0 0 0 0 0 0 0 0 0

3.4641	2.5000	-0.0000 C	0 0 0 0 0 0 0 0 0 0 0 0

2.5981	3.0000	-0.0000 C	0 0 0 0 0 0 0 0 0 0 0 0

1.7321	2.5000	-0.0000 C	0 0 0 0 0 0 0 0 0 0 0 0

1.7321	1.5000	-0.0000 C	0 0 0 0 0 0 0 0 0 0 0 0

0.8660	1.0000	-0.0000 N	0 0 0 0 0 0 0 0 0 0 0 0

-0.0000	1.5000	-0.0000 O	0 0 0 0 0 0 0 0 0 0 0 0

0.8660	-0.0000	-0.0000 O	0 0 0 0 0 0 0 0 0 0 0 0

2 1 2 0 0 0 0

3 2 1 0 0 0 0

4 3 1 0 0 0 0

5 4 1 0 0 0 0

6 3 2 0 0 0 0

7 2 1 0 0 0 0

8 7 2 0 0 0 0

9 8 1 0 0 0 0

10 9 2 0 0 0 0

11 10 1 0 0 0 0

12 11 2 0 0 0 0

12 7 1 0 0 0 0

13 12 1 0 0 0 0

14 13 1 0 0 0 0

15 13 2 0 0 0 0

M CHG 2 13	1 14 -1

M END

**Methyl 2-(4-Fluoro-2-nitrophenyl)acrylate (2b)**

Compound identifier (MolFile):

SMILES: C=C(C(OC)=O)C1=CC=C(F)C=C1[N+]([O-])=O

MOL Text:

Actelion Java MolfileCreator 1.0

16 16 0 0 0 0 0 0 0 0999 V2000

1.7321	-0.5000	-0.0000 C	0 0 0 0 0 0 0 0 0 0 0 0

2.5981	-0.0000	-0.0000 C	0 0 0 0 0 0 0 0 0 0 0 0

3.4641	-0.5000	-0.0000 C	0 0 0 0 0 0 0 0 0 0 0 0

4.3301	0.0000	-0.0000 O	0 0 0 0 0 0 0 0 0 0 0 0

5.1962	-0.5000	-0.0000 C	0 0 0 0 0 0 0 0 0 0 0 0

3.4641	-1.5000	-0.0000 O	0 0 0 0 0 0 0 0 0 0 0 0

2.5981	1.0000	-0.0000 C	0 0 0 0 0 0 0 0 0 0 0 0

3.4641	1.5000	-0.0000 C	0 0 0 0 0 0 0 0 0 0 0 0

3.4641	2.5000	-0.0000 C	0 0 0 0 0 0 0 0 0 0 0 0

2.5981	3.0000	-0.0000 C	0 0 0 0 0 0 0 0 0 0 0 0

2.5981	4.0000	-0.0000 F	0 0 0 0 0 0 0 0 0 0 0 0

1.7321	2.5000	-0.0000 C	0 0 0 0 0 0 0 0 0 0 0 0

1.7321	1.5000	-0.0000 C	0 0 0 0 0 0 0 0 0 0 0 0

0.8660	1.0000	-0.0000 N	0 0 0 0 0 0 0 0 0 0 0 0

-0.0000	1.5000	-0.0000 O	0 0 0 0 0 0 0 0 0 0 0 0

0.8660	-0.0000	-0.0000 O	0 0 0 0 0 0 0 0 0 0 0 0

2 1 2 0 0 0 0

3 2 1 0 0 0 0

4 3 1 0 0 0 0

5 4 1 0 0 0 0

6 3 2 0 0 0 0

7 2 1 0 0 0 0

8 7 2 0 0 0 0

9 8 1 0 0 0 0

10 9 2 0 0 0 0

11 10 1 0 0 0 0

12 10 1 0 0 0 0

13 12 2 0 0 0 0

13 7 1 0 0 0 0

14 13 1 0 0 0 0

15 14 1 0 0 0 0

16 14 2 0 0 0 0

M CHG 2 14	1 15 -1

M END

**Methyl 2-(4-Chloro-2-nitrophenyl)acrylate (2c)**

Compound identifier (MolFile):

SMILES: C=C(C(OC)=O)C1=CC=C(Cl)C=C1[N+]([O-])=O

MOL Text:

Actelion Java MolfileCreator 1.0

16 16 0 0 0 0 0 0 0 0999 V2000

1.7321	-0.5000	-0.0000 C	0 0 0 0 0 0 0 0 0 0 0 0

2.5981	-0.0000	-0.0000 C	0 0 0 0 0 0 0 0 0 0 0 0

3.4641	-0.5000	-0.0000 C	0 0 0 0 0 0 0 0 0 0 0 0

4.3301	0.0000	-0.0000 O	0 0 0 0 0 0 0 0 0 0 0 0

5.1962	-0.5000	-0.0000 C	0 0 0 0 0 0 0 0 0 0 0 0

3.4641	-1.5000	-0.0000 O	0 0 0 0 0 0 0 0 0 0 0 0

2.5981	1.0000	-0.0000 C	0 0 0 0 0 0 0 0 0 0 0 0

3.4641	1.5000	-0.0000 C	0 0 0 0 0 0 0 0 0 0 0 0

3.4641	2.5000	-0.0000 C	0 0 0 0 0 0 0 0 0 0 0 0

2.5981	3.0000	-0.0000 C	0 0 0 0 0 0 0 0 0 0 0 0

2.5981	4.0000	-0.0000 Cl 0 0 0 0 0 0 0 0 0 0 0 0

1.7321	2.5000	-0.0000 C	0 0 0 0 0 0 0 0 0 0 0 0

1.7321	1.5000	-0.0000 C	0 0 0 0 0 0 0 0 0 0 0 0

0.8660	1.0000	-0.0000 N	0 0 0 0 0 0 0 0 0 0 0 0

-0.0000	1.5000	-0.0000 O	0 0 0 0 0 0 0 0 0 0 0 0

0.8660	-0.0000	-0.0000 O	0 0 0 0 0 0 0 0 0 0 0 0

2 1 2 0 0 0 0

3 2 1 0 0 0 0

4 3 1 0 0 0 0

5 4 1 0 0 0 0

6 3 2 0 0 0 0

7 2 1 0 0 0 0

8 7 2 0 0 0 0

9 8 1 0 0 0 0

10 9 2 0 0 0 0

11 10 1 0 0 0 0

12 10 1 0 0 0 0

13 12 2 0 0 0 0

13 7 1 0 0 0 0

14 13 1 0 0 0 0

15 14 1 0 0 0 0

16 14 2 0 0 0 0

M CHG 2 14	1 15 -1

M END

**Methyl 2-(4-Bromo-2-nitrophenyl)acrylate (2d)**

Compound identifier (MolFile):

SMILES: C=C(C(OC)=O)C1=CC=C(Br)C=C1[N+]([O-])=O

MOL Text:

Actelion Java MolfileCreator 1.0

16 16 0 0 0 0 0 0 0 0999 V2000

1.7321	-0.5000	-0.0000 C	0 0 0 0 0 0 0 0 0 0 0 0

2.5981	-0.0000	-0.0000 C	0 0 0 0 0 0 0 0 0 0 0 0

3.4641	-0.5000	-0.0000 C	0 0 0 0 0 0 0 0 0 0 0 0

4.3301	0.0000	-0.0000 O	0 0 0 0 0 0 0 0 0 0 0 0

5.1962	-0.5000	-0.0000 C	0 0 0 0 0 0 0 0 0 0 0 0

3.4641	-1.5000	-0.0000 O	0 0 0 0 0 0 0 0 0 0 0 0

2.5981	1.0000	-0.0000 C	0 0 0 0 0 0 0 0 0 0 0 0

3.4641	1.5000	-0.0000 C	0 0 0 0 0 0 0 0 0 0 0 0

3.4641	2.5000	-0.0000 C	0 0 0 0 0 0 0 0 0 0 0 0

2.5981	3.0000	-0.0000 C	0 0 0 0 0 0 0 0 0 0 0 0

2.5981	4.0000	-0.0000 Br 0 0 0 0 0 0 0 0 0 0 0 0

1.7321	2.5000	-0.0000 C	0 0 0 0 0 0 0 0 0 0 0 0

1.7321	1.5000	-0.0000 C	0 0 0 0 0 0 0 0 0 0 0 0

0.8660	1.0000	-0.0000 N	0 0 0 0 0 0 0 0 0 0 0 0

-0.0000	1.5000	-0.0000 O	0 0 0 0 0 0 0 0 0 0 0 0

0.8660	-0.0000	-0.0000 O	0 0 0 0 0 0 0 0 0 0 0 0

2 1 2 0 0 0 0

3 2 1 0 0 0 0

4 3 1 0 0 0 0

5 4 1 0 0 0 0

6 3 2 0 0 0 0

7 2 1 0 0 0 0

8 7 2 0 0 0 0

9 8 1 0 0 0 0

10 9 2 0 0 0 0

11 10 1 0 0 0 0

12 10 1 0 0 0 0

13 12 2 0 0 0 0

13 7 1 0 0 0 0

14 13 1 0 0 0 0

15 14 1 0 0 0 0

16 14 2 0 0 0 0

M CHG 2 14	1 15 -1

M END

**Methyl 2-(5-Fluoro-2-nitrophenyl)acrylate (2e)**

Compound identifier (MolFile):

SMILES: C=C(C(OC)=O)C1=CC(F)=CC=C1[N+]([O-])=O

MOL Text:

Actelion Java MolfileCreator 1.0

16 16 0 0 0 0 0 0 0 0999 V2000

1.7321	-0.5000	-0.0000 C	0 0 0 0 0 0 0 0 0 0 0 0

2.5981	-0.0000	-0.0000 C	0 0 0 0 0 0 0 0 0 0 0 0

3.4641	-0.5000	-0.0000 C	0 0 0 0 0 0 0 0 0 0 0 0

4.3301	0.0000	-0.0000 O	0 0 0 0 0 0 0 0 0 0 0 0

5.1962	-0.5000	-0.0000 C	0 0 0 0 0 0 0 0 0 0 0 0

3.4641	-1.5000	-0.0000 O	0 0 0 0 0 0 0 0 0 0 0 0

2.5981	1.0000	-0.0000 C	0 0 0 0 0 0 0 0 0 0 0 0

3.4641	1.5000	-0.0000 C	0 0 0 0 0 0 0 0 0 0 0 0

3.4641	2.5000	-0.0000 C	0 0 0 0 0 0 0 0 0 0 0 0

4.3301	3.0000	-0.0000 F	0 0 0 0 0 0 0 0 0 0 0 0

2.5981	3.0000	-0.0000 C	0 0 0 0 0 0 0 0 0 0 0 0

1.7321	2.5000	-0.0000 C	0 0 0 0 0 0 0 0 0 0 0 0

1.7321	1.5000	-0.0000 C	0 0 0 0 0 0 0 0 0 0 0 0

0.8660	1.0000	-0.0000 N	0 0 0 0 0 0 0 0 0 0 0 0

-0.0000	1.5000	-0.0000 O	0 0 0 0 0 0 0 0 0 0 0 0

0.8660	-0.0000	-0.0000 O	0 0 0 0 0 0 0 0 0 0 0 0

2 1 2 0 0 0 0

3 2 1 0 0 0 0

4 3 1 0 0 0 0

5 4 1 0 0 0 0

6 3 2 0 0 0 0

7 2 1 0 0 0 0

8 7 2 0 0 0 0

9 8 1 0 0 0 0

10 9 1 0 0 0 0

11 9 2 0 0 0 0

12 11 1 0 0 0 0

13 12 2 0 0 0 0

13 7 1 0 0 0 0

14 13 1 0 0 0 0

15 14 1 0 0 0 0

16 14 2 0 0 0 0

M CHG 2 14	1 15 -1

M END

**Methyl 2-(3-Fluoro-2-nitrophenyl)acrylate (2f)**

Compound identifier (MolFile):

SMILES: C=C(C(OC)=O)C1=CC=CC(F)=C1[N+]([O-])=O

MOL Text:

Actelion Java MolfileCreator 1.0

16 16 0 0 0 0 0 0 0 0999 V2000

1.7321	-0.5000	-0.0000 C	0 0 0 0 0 0 0 0 0 0 0 0

2.5981	-0.0000	-0.0000 C	0 0 0 0 0 0 0 0 0 0 0 0

3.4641	-0.5000	-0.0000 C	0 0 0 0 0 0 0 0 0 0 0 0

4.3301	0.0000	-0.0000 O	0 0 0 0 0 0 0 0 0 0 0 0

5.1962	-0.5000	-0.0000 C	0 0 0 0 0 0 0 0 0 0 0 0

3.4641	-1.5000	-0.0000 O	0 0 0 0 0 0 0 0 0 0 0 0

2.5981	1.0000	-0.0000 C	0 0 0 0 0 0 0 0 0 0 0 0

3.4641	1.5000	-0.0000 C	0 0 0 0 0 0 0 0 0 0 0 0

3.4641	2.5000	-0.0000 C	0 0 0 0 0 0 0 0 0 0 0 0

2.5981	3.0000	-0.0000 C	0 0 0 0 0 0 0 0 0 0 0 0

1.7321	2.5000	-0.0000 C	0 0 0 0 0 0 0 0 0 0 0 0

0.8660	3.0000	-0.0000 F	0 0 0 0 0 0 0 0 0 0 0 0

1.7321	1.5000	-0.0000 C	0 0 0 0 0 0 0 0 0 0 0 0

0.8660	1.0000	-0.0000 N	0 0 0 0 0 0 0 0 0 0 0 0

-0.0000	1.5000	-0.0000 O	0 0 0 0 0 0 0 0 0 0 0 0

0.8660	-0.0000	-0.0000 O	0 0 0 0 0 0 0 0 0 0 0 0

2 1 2 0 0 0 0

3 2 1 0 0 0 0

4 3 1 0 0 0 0

5 4 1 0 0 0 0

6 3 2 0 0 0 0

7 2 1 0 0 0 0

8 7 2 0 0 0 0

9 8 1 0 0 0 0

10 9 2 0 0 0 0

11 10 1 0 0 0 0

12 11 1 0 0 0 0

13 11 2 0 0 0 0

13 7 1 0 0 0 0

14 13 1 0 0 0 0

15 14 1 0 0 0 0

16 14 2 0 0 0 0

M CHG 2 14	1 15 -1

M END

**Methyl 2-(4-Methoxy-2-nitrophenyl)acrylate (2g)**

Compound identifier (MolFile):

SMILES: C=C(C(OC)=O)C1=CC=C(OC)C=C1[N+]([O-])=O

MOL Text:

Actelion Java MolfileCreator 1.0

17 17 0 0 0 0 0 0 0 0999 V2000

2.5981	-0.7500	-0.0000 C	0 0 0 0 0 0 0 0 0 0 0 0

3.8971	-0.0000	-0.0000 C	0 0 0 0 0 0 0 0 0 0 0 0

5.1962	-0.7500	-0.0000 C	0 0 0 0 0 0 0 0 0 0 0 0

6.4952	0.0000	-0.0000 O	0 0 0 0 0 0 0 0 0 0 0 0

7.7942	-0.7500	-0.0000 C	0 0 0 0 0 0 0 0 0 0 0 0

5.1962	-2.2500	-0.0000 O	0 0 0 0 0 0 0 0 0 0 0 0

3.8971	1.5000	-0.0000 C	0 0 0 0 0 0 0 0 0 0 0 0

5.1962	2.2500	-0.0000 C	0 0 0 0 0 0 0 0 0 0 0 0

5.1962	3.7500	-0.0000 C	0 0 0 0 0 0 0 0 0 0 0 0

3.8971	4.5000	-0.0000 C	0 0 0 0 0 0 0 0 0 0 0 0

3.8971	6.0000	-0.0000 O	0 0 0 0 0 0 0 0 0 0 0 0

2.5981	6.7500	-0.0000 C	0 0 0 0 0 0 0 0 0 0 0 0

2.5981	3.7500	-0.0000 C	0 0 0 0 0 0 0 0 0 0 0 0

2.5981	2.2500	-0.0000 C	0 0 0 0 0 0 0 0 0 0 0 0

1.2990	1.5000	-0.0000 N	0 0 0 0 0 0 0 0 0 0 0 0

0.0000	2.2500	-0.0000 O	0 0 0 0 0 0 0 0 0 0 0 0

1.2990	-0.0000	-0.0000 O	0 0 0 0 0 0 0 0 0 0 0 0

2 1 2 0 0 0 0

3 2 1 0 0 0 0

4 3 1 0 0 0 0

5 4 1 0 0 0 0

6 3 2 0 0 0 0

7 2 1 0 0 0 0

8 7 2 0 0 0 0

9 8 1 0 0 0 0

10 9 2 0 0 0 0

11 10 1 0 0 0 0

12 11 1 0 0 0 0

13 10 1 0 0 0 0

14 13 2 0 0 0 0

14 7 1 0 0 0 0

15 14 1 0 0 0 0

16 15 1 0 0 0 0

17 15 2 0 0 0 0

M CHG 2 15	1 16 -1

M END

**Methyl-1-(2-Nitrophenyl)-4-oxocylohexanecarboxylate (3a)**

Compound identifier (MolFile):

SMILES: O=C(CC1)CCC1(C(OC)=O)C2=CC=CC=C2[N+]([O-])=O

MOL Text:

Actelion Java MolfileCreator 1.0

20 21 0 0 0 0 0 0 0 0999 V2000

0.8660	4.0000	-0.0000 O	0 0 0 0 0 0 0 0 0 0 0 0

0.8660	3.0000	-0.0000 C	0 0 0 0 0 0 0 0 0 0 0 0

-0.0000	2.5000	-0.0000 C	0 0 0 0 0 0 0 0 0 0 0 0

-0.0000	1.5000	-0.0000 C	0 0 0 0 0 0 0 0 0 0 0 0

1.7321	2.5000	-0.0000 C	0 0 0 0 0 0 0 0 0 0 0 0

1.7321	1.5000	-0.0000 C	0 0 0 0 0 0 0 0 0 0 0 0

0.8660	1.0000	-0.0000 C	0 0 0 0 0 0 0 0 0 0 0 0

0.8660	-0.0000	-0.0000 C	0 0 0 0 0 0 0 0 0 0 0 0

1.8376	-0.6266	-0.0000 O	0 0 0 0 0 0 0 0 0 0 0 0

2.5981	-0.0000	-0.0000 C	0 0 0 0 0 0 0 0 0 0 0 0

-0.2083	-0.4278	-0.0000 O	0 0 0 0 0 0 0 0 0 0 0 0

0.0000	0.5000	-0.0000 C	0 0 0 0 0 0 0 0 0 0 0 0

-0.8660	1.0000	-0.0000 C	0 0 0 0 0 0 0 0 0 0 0 0

-1.7321	0.5000	-0.0000 C	0 0 0 0 0 0 0 0 0 0 0 0

-1.7321	-0.5000	-0.0000 C	0 0 0 0 0 0 0 0 0 0 0 0

-0.8660	-1.0000	-0.0000 C	0 0 0 0 0 0 0 0 0 0 0 0

0.0658	-0.6653	-0.0000 C	0 0 0 0 0 0 0 0 0 0 0 0

0.8660	-1.0000	-0.0000 N	0 0 0 0 0 0 0 0 0 0 0 0

0.8660	-2.0000	-0.0000 O	0 0 0 0 0 0 0 0 0 0 0 0

1.7372	-0.2740	-0.0000 O	0 0 0 0 0 0 0 0 0 0 0 0

2 1 2 0 0 0 0

3 2 1 0 0 0 0

4 3 1 0 0 0 0

5 2 1 0 0 0 0

6 5 1 0 0 0 0

7 6 1 0 0 0 0

7 4 1 0 0 0 0

8 7 1 0 0 0 0

9 8 1 0 0 0 0

10 9 1 0 0 0 0

11 8 2 0 0 0 0

12 7 1 0 0 0 0

13 12 2 0 0 0 0

14 13 1 0 0 0 0

15 14 2 0 0 0 0

16 15 1 0 0 0 0

17 16 2 0 0 0 0

17 12 1 0 0 0 0

18 17 1 0 0 0 0

19 18 1 0 0 0 0

20 18 2 0 0 0 0

M CHG 2 18	1 19 -1

M END

**Methyl 1-(4-Fluoro-2-nitrophenyl)-4-oxocyclohexane-1-carboxylate (3b)**

Compound identifier (MolFile):

SMILES: O=C(CC1)CCC1(C(OC)=O)C2=CC=C(F)C=C2[N+]([O-])=O

MOL Text:

Actelion Java MolfileCreator 1.0

21 22 0 0 0 0 0 0 0 0999 V2000

0.8660	4.0000	-0.0000 O	0 0 0 0 0 0 0 0 0 0 0 0

0.8660	3.0000	-0.0000 C	0 0 0 0 0 0 0 0 0 0 0 0

-0.0000	2.5000	-0.0000 C	0 0 0 0 0 0 0 0 0 0 0 0

-0.0000	1.5000	-0.0000 C	0 0 0 0 0 0 0 0 0 0 0 0

1.7321	2.5000	-0.0000 C	0 0 0 0 0 0 0 0 0 0 0 0

1.7321	1.5000	-0.0000 C	0 0 0 0 0 0 0 0 0 0 0 0

0.8660	1.0000	-0.0000 C	0 0 0 0 0 0 0 0 0 0 0 0

0.8660	-0.0000	-0.0000 C	0 0 0 0 0 0 0 0 0 0 0 0

1.8376	-0.6266	-0.0000 O	0 0 0 0 0 0 0 0 0 0 0 0

2.5981	-0.0000	-0.0000 C	0 0 0 0 0 0 0 0 0 0 0 0

-0.2083	-0.4278	-0.0000 O	0 0 0 0 0 0 0 0 0 0 0 0

0.0000	0.5000	-0.0000 C	0 0 0 0 0 0 0 0 0 0 0 0

-0.8660	1.0000	-0.0000 C	0 0 0 0 0 0 0 0 0 0 0 0

-1.7321	0.5000	-0.0000 C	0 0 0 0 0 0 0 0 0 0 0 0

-1.7321	-0.5000	-0.0000 C	0 0 0 0 0 0 0 0 0 0 0 0

-2.5981	-1.0000	-0.0000 F	0 0 0 0 0 0 0 0 0 0 0 0

-0.8660	-1.0000	-0.0000 C	0 0 0 0 0 0 0 0 0 0 0 0

0.0658	-0.6653	-0.0000 C	0 0 0 0 0 0 0 0 0 0 0 0

0.8660	-1.0000	-0.0000 N	0 0 0 0 0 0 0 0 0 0 0 0

0.8660	-2.0000	-0.0000 O	0 0 0 0 0 0 0 0 0 0 0 0

1.7372	-0.2740	-0.0000 O	0 0 0 0 0 0 0 0 0 0 0 0

2 1 2 0 0 0 0

3 2 1 0 0 0 0

4 3 1 0 0 0 0

5 2 1 0 0 0 0

6 5 1 0 0 0 0

7 6 1 0 0 0 0

7 4 1 0 0 0 0

8 7 1 0 0 0 0

9 8 1 0 0 0 0

10 9 1 0 0 0 0

11 8 2 0 0 0 0

12 7 1 0 0 0 0

13 12 2 0 0 0 0

14 13 1 0 0 0 0

15 14 2 0 0 0 0

16 15 1 0 0 0 0

17 15 1 0 0 0 0

18 17 2 0 0 0 0

18 12 1 0 0 0 0

19 18 1 0 0 0 0

20 19 1 0 0 0 0

21 19 2 0 0 0 0

M CHG 2 19	1 20 -1

M END

**Methyl 1-(4-Chloro-2-nitrophenyl)-4-oxocyclohexane-1-carboxylate (3c)**

Compound identifier (MolFile):

SMILES: O=C(CC1)CCC1(C(OC)=O)C2=CC=C(Cl)C=C2[N+]([O-])=O

MOL Text:

Actelion Java MolfileCreator 1.0

21 22 0 0 0 0 0 0 0 0999 V2000

0.8660	4.0000	-0.0000 O	0 0 0 0 0 0 0 0 0 0 0 0

0.8660	3.0000	-0.0000 C	0 0 0 0 0 0 0 0 0 0 0 0

-0.0000	2.5000	-0.0000 C	0 0 0 0 0 0 0 0 0 0 0 0

-0.0000	1.5000	-0.0000 C	0 0 0 0 0 0 0 0 0 0 0 0

1.7321	2.5000	-0.0000 C	0 0 0 0 0 0 0 0 0 0 0 0

1.7321	1.5000	-0.0000 C	0 0 0 0 0 0 0 0 0 0 0 0

0.8660	1.0000	-0.0000 C	0 0 0 0 0 0 0 0 0 0 0 0

0.8660	-0.0000	-0.0000 C	0 0 0 0 0 0 0 0 0 0 0 0

1.8376	-0.6266	-0.0000 O	0 0 0 0 0 0 0 0 0 0 0 0

2.5981	-0.0000	-0.0000 C	0 0 0 0 0 0 0 0 0 0 0 0

-0.2083	-0.4278	-0.0000 O	0 0 0 0 0 0 0 0 0 0 0 0

0.0000	0.5000	-0.0000 C	0 0 0 0 0 0 0 0 0 0 0 0

-0.8660	1.0000	-0.0000 C	0 0 0 0 0 0 0 0 0 0 0 0

-1.7321	0.5000	-0.0000 C	0 0 0 0 0 0 0 0 0 0 0 0

-1.7321	-0.5000	-0.0000 C	0 0 0 0 0 0 0 0 0 0 0 0

-2.5981	-1.0000	-0.0000 Cl 0 0 0 0 0 0 0 0 0 0 0 0

-0.8660	-1.0000	-0.0000 C	0 0 0 0 0 0 0 0 0 0 0 0

0.0658	-0.6653	-0.0000 C	0 0 0 0 0 0 0 0 0 0 0 0

0.8660	-1.0000	-0.0000 N	0 0 0 0 0 0 0 0 0 0 0 0

0.8660	-2.0000	-0.0000 O	0 0 0 0 0 0 0 0 0 0 0 0

1.7372	-0.2740	-0.0000 O	0 0 0 0 0 0 0 0 0 0 0 0

2 1 2 0 0 0 0

3 2 1 0 0 0 0

4 3 1 0 0 0 0

5 2 1 0 0 0 0

6 5 1 0 0 0 0

7 6 1 0 0 0 0

7 4 1 0 0 0 0

8 7 1 0 0 0 0

9 8 1 0 0 0 0

10 9 1 0 0 0 0

11 8 2 0 0 0 0

12 7 1 0 0 0 0

13 12 2 0 0 0 0

14 13 1 0 0 0 0

15 14 2 0 0 0 0

16 15 1 0 0 0 0

17 15 1 0 0 0 0

18 17 2 0 0 0 0

18 12 1 0 0 0 0

19 18 1 0 0 0 0

20 19 1 0 0 0 0

21 19 2 0 0 0 0

M CHG 2 19	1 20 -1

M END

**Methyl 1-(4-Bromo-2-nitrophenyl)-4-oxocyclohexane-1-carboxylate (3d)**

Compound identifier (MolFile):

SMILES: O=C(CC1)CCC1(C(OC)=O)C2=CC=C(Br)C=C2[N+]([O-])=O

MOL Text:

Actelion Java MolfileCreator 1.0

21 22 0 0 0 0 0 0 0 0999 V2000

0.8660	4.0000	-0.0000 O	0 0 0 0 0 0 0 0 0 0 0 0

0.8660	3.0000	-0.0000 C	0 0 0 0 0 0 0 0 0 0 0 0

-0.0000	2.5000	-0.0000 C	0 0 0 0 0 0 0 0 0 0 0 0

-0.0000	1.5000	-0.0000 C	0 0 0 0 0 0 0 0 0 0 0 0

1.7321	2.5000	-0.0000 C	0 0 0 0 0 0 0 0 0 0 0 0

1.7321	1.5000	-0.0000 C	0 0 0 0 0 0 0 0 0 0 0 0

0.8660	1.0000	-0.0000 C	0 0 0 0 0 0 0 0 0 0 0 0

0.8660	-0.0000	-0.0000 C	0 0 0 0 0 0 0 0 0 0 0 0

1.8376	-0.6266	-0.0000 O	0 0 0 0 0 0 0 0 0 0 0 0

2.5981	-0.0000	-0.0000 C	0 0 0 0 0 0 0 0 0 0 0 0

-0.2083	-0.4278	-0.0000 O	0 0 0 0 0 0 0 0 0 0 0 0

0.0000	0.5000	-0.0000 C	0 0 0 0 0 0 0 0 0 0 0 0

-0.8660	1.0000	-0.0000 C	0 0 0 0 0 0 0 0 0 0 0 0

-1.7321	0.5000	-0.0000 C	0 0 0 0 0 0 0 0 0 0 0 0

-1.7321	-0.5000	-0.0000 C	0 0 0 0 0 0 0 0 0 0 0 0

-2.5981	-1.0000	-0.0000 Br 0 0 0 0 0 0 0 0 0 0 0 0

-0.8660	-1.0000	-0.0000 C	0 0 0 0 0 0 0 0 0 0 0 0

0.0658	-0.6653	-0.0000 C	0 0 0 0 0 0 0 0 0 0 0 0

0.8660	-1.0000	-0.0000 N	0 0 0 0 0 0 0 0 0 0 0 0

0.8660	-2.0000	-0.0000 O	0 0 0 0 0 0 0 0 0 0 0 0

1.7372	-0.2740	-0.0000 O	0 0 0 0 0 0 0 0 0 0 0 0

2 1 2 0 0 0 0

3 2 1 0 0 0 0

4 3 1 0 0 0 0

5 2 1 0 0 0 0

6 5 1 0 0 0 0

7 6 1 0 0 0 0

7 4 1 0 0 0 0

8 7 1 0 0 0 0

9 8 1 0 0 0 0

10 9 1 0 0 0 0

11 8 2 0 0 0 0

12 7 1 0 0 0 0

13 12 2 0 0 0 0

14 13 1 0 0 0 0

15 14 2 0 0 0 0

16 15 1 0 0 0 0

17 15 1 0 0 0 0

18 17 2 0 0 0 0

18 12 1 0 0 0 0

19 18 1 0 0 0 0

20 19 1 0 0 0 0

21 19 2 0 0 0 0

M CHG 2 19	1 20 -1

M END

**Methyl 1-(5-Fluoro-2-nitrophenyl)-4-oxocyclohexane-1-carboxylate (3e)**

Compound identifier (MolFile):

SMILES: O=C(CC1)CCC1(C(OC)=O)C2=CC(F)=CC=C2[N+]([O-])=O

MOL Text:

Actelion Java MolfileCreator 1.0

21 22 0 0 0 0 0 0 0 0999 V2000

0.8660	4.0000	-0.0000 O	0 0 0 0 0 0 0 0 0 0 0 0

0.8660	3.0000	-0.0000 C	0 0 0 0 0 0 0 0 0 0 0 0

-0.0000	2.5000	-0.0000 C	0 0 0 0 0 0 0 0 0 0 0 0

-0.0000	1.5000	-0.0000 C	0 0 0 0 0 0 0 0 0 0 0 0

1.7321	2.5000	-0.0000 C	0 0 0 0 0 0 0 0 0 0 0 0

1.7321	1.5000	-0.0000 C	0 0 0 0 0 0 0 0 0 0 0 0

0.8660	1.0000	-0.0000 C	0 0 0 0 0 0 0 0 0 0 0 0

0.8660	-0.0000	-0.0000 C	0 0 0 0 0 0 0 0 0 0 0 0

1.8376	-0.6266	-0.0000 O	0 0 0 0 0 0 0 0 0 0 0 0

2.5981	-0.0000	-0.0000 C	0 0 0 0 0 0 0 0 0 0 0 0

-0.2083	-0.4278	-0.0000 O	0 0 0 0 0 0 0 0 0 0 0 0

0.0000	0.5000	-0.0000 C	0 0 0 0 0 0 0 0 0 0 0 0

-0.8660	1.0000	-0.0000 C	0 0 0 0 0 0 0 0 0 0 0 0

-1.7321	0.5000	-0.0000 C	0 0 0 0 0 0 0 0 0 0 0 0

-2.5981	1.0000	-0.0000 F	0 0 0 0 0 0 0 0 0 0 0 0

-1.7321	-0.5000	-0.0000 C	0 0 0 0 0 0 0 0 0 0 0 0

-0.8660	-1.0000	-0.0000 C	0 0 0 0 0 0 0 0 0 0 0 0

0.0658	-0.6653	-0.0000 C	0 0 0 0 0 0 0 0 0 0 0 0

0.8660	-1.0000	-0.0000 N	0 0 0 0 0 0 0 0 0 0 0 0

0.8660	-2.0000	-0.0000 O	0 0 0 0 0 0 0 0 0 0 0 0

1.7372	-0.2740	-0.0000 O	0 0 0 0 0 0 0 0 0 0 0 0

2 1 2 0 0 0 0

3 2 1 0 0 0 0

4 3 1 0 0 0 0

5 2 1 0 0 0 0

6 5 1 0 0 0 0

7 6 1 0 0 0 0

7 4 1 0 0 0 0

8 7 1 0 0 0 0

9 8 1 0 0 0 0

10 9 1 0 0 0 0

11 8 2 0 0 0 0

12 7 1 0 0 0 0

13 12 2 0 0 0 0

14 13 1 0 0 0 0

15 14 1 0 0 0 0

16 14 2 0 0 0 0

17 16 1 0 0 0 0

18 17 2 0 0 0 0

18 12 1 0 0 0 0

19 18 1 0 0 0 0

20 19 1 0 0 0 0

21 19 2 0 0 0 0

M CHG 2 19	1 20 -1

M END

**Methyl 1-(3-Fluoro-2-nitrophenyl)-4-oxocyclohexane-1-carboxylate (3f)**

Compound identifier (MolFile):

SMILES: O=C(CC1)CCC1(C(OC)=O)C2=CC=CC(F)=C2[N+]([O-])=O

MOL Text:

Actelion Java MolfileCreator 1.0

21 22 0 0 0 0 0 0 0 0999 V2000

0.8660	4.0000	-0.0000 O	0 0 0 0 0 0 0 0 0 0 0 0

0.8660	3.0000	-0.0000 C	0 0 0 0 0 0 0 0 0 0 0 0

-0.0000	2.5000	-0.0000 C	0 0 0 0 0 0 0 0 0 0 0 0

-0.0000	1.5000	-0.0000 C	0 0 0 0 0 0 0 0 0 0 0 0

1.7321	2.5000	-0.0000 C	0 0 0 0 0 0 0 0 0 0 0 0

1.7321	1.5000	-0.0000 C	0 0 0 0 0 0 0 0 0 0 0 0

0.8660	1.0000	-0.0000 C	0 0 0 0 0 0 0 0 0 0 0 0

0.8660	-0.0000	-0.0000 C	0 0 0 0 0 0 0 0 0 0 0 0

1.8376	-0.6266	-0.0000 O	0 0 0 0 0 0 0 0 0 0 0 0

2.5981	-0.0000	-0.0000 C	0 0 0 0 0 0 0 0 0 0 0 0

-0.2083	-0.4278	-0.0000 O	0 0 0 0 0 0 0 0 0 0 0 0

0.0000	0.5000	-0.0000 C	0 0 0 0 0 0 0 0 0 0 0 0

-0.8660	1.0000	-0.0000 C	0 0 0 0 0 0 0 0 0 0 0 0

-1.7321	0.5000	-0.0000 C	0 0 0 0 0 0 0 0 0 0 0 0

-1.7321	-0.5000	-0.0000 C	0 0 0 0 0 0 0 0 0 0 0 0

-0.8660	-1.0000	-0.0000 C	0 0 0 0 0 0 0 0 0 0 0 0

-0.8660	-2.0000	-0.0000 F	0 0 0 0 0 0 0 0 0 0 0 0

0.0658	-0.6653	-0.0000 C	0 0 0 0 0 0 0 0 0 0 0 0

0.8660	-1.0000	-0.0000 N	0 0 0 0 0 0 0 0 0 0 0 0

0.8660	-2.0000	-0.0000 O	0 0 0 0 0 0 0 0 0 0 0 0

1.7372	-0.2740	-0.0000 O	0 0 0 0 0 0 0 0 0 0 0 0

2 1 2 0 0 0 0

3 2 1 0 0 0 0

4 3 1 0 0 0 0

5 2 1 0 0 0 0

6 5 1 0 0 0 0

7 6 1 0 0 0 0

7 4 1 0 0 0 0

8 7 1 0 0 0 0

9 8 1 0 0 0 0

10 9 1 0 0 0 0

11 8 2 0 0 0 0

12 7 1 0 0 0 0

13 12 2 0 0 0 0

14 13 1 0 0 0 0

15 14 2 0 0 0 0

16 15 1 0 0 0 0

17 16 1 0 0 0 0

18 16 2 0 0 0 0

18 12 1 0 0 0 0

19 18 1 0 0 0 0

20 19 1 0 0 0 0

21 19 2 0 0 0 0

M CHG 2 19	1 20 -1

M END

**Methyl 1-(4-Methoxy-2-nitrophenyl)-4-oxocyclohexane-1-carboxylate (3g)**

Compound identifier (MolFile):

SMILES: O=C(CC1)CCC1(C(OC)=O)C2=CC=C(OC)C=C2[N+]([O-])=O

MOL Text:

Actelion Java MolfileCreator 1.0

22 23 0 0 0 0 0 0 0 0999 V2000

0.8660	4.0000	-0.0000 O	0 0 0 0 0 0 0 0 0 0 0 0

0.8660	3.0000	-0.0000 C	0 0 0 0 0 0 0 0 0 0 0 0

-0.0000	2.5000	-0.0000 C	0 0 0 0 0 0 0 0 0 0 0 0

-0.0000	1.5000	-0.0000 C	0 0 0 0 0 0 0 0 0 0 0 0

1.7321	2.5000	-0.0000 C	0 0 0 0 0 0 0 0 0 0 0 0

1.7321	1.5000	-0.0000 C	0 0 0 0 0 0 0 0 0 0 0 0

0.8660	1.0000	-0.0000 C	0 0 0 0 0 0 0 0 0 0 0 0

0.8660	-0.0000	-0.0000 C	0 0 0 0 0 0 0 0 0 0 0 0

1.8378	-0.6271	-0.0000 O	0 0 0 0 0 0 0 0 0 0 0 0

2.5981	-0.0000	-0.0000 C	0 0 0 0 0 0 0 0 0 0 0 0

-0.1985	-0.3900	-0.0000 O	0 0 0 0 0 0 0 0 0 0 0 0

0.0000	0.5000	-0.0000 C	0 0 0 0 0 0 0 0 0 0 0 0

-0.8660	1.0000	-0.0000 C	0 0 0 0 0 0 0 0 0 0 0 0

-1.7321	0.5000	-0.0000 C	0 0 0 0 0 0 0 0 0 0 0 0

-1.7321	-0.5000	-0.0000 C	0 0 0 0 0 0 0 0 0 0 0 0

-2.5981	-1.0000	-0.0000 O	0 0 0 0 0 0 0 0 0 0 0 0

-2.5981	-2.0000	-0.0000 C	0 0 0 0 0 0 0 0 0 0 0 0

-0.8660	-1.0000	-0.0000 C	0 0 0 0 0 0 0 0 0 0 0 0

0.0565	-0.6542	-0.0000 C	0 0 0 0 0 0 0 0 0 0 0 0

0.8660	-1.0000	-0.0000 N	0 0 0 0 0 0 0 0 0 0 0 0

0.8660	-2.0000	-0.0000 O	0 0 0 0 0 0 0 0 0 0 0 0

1.7183	-0.2911	-0.0000 O	0 0 0 0 0 0 0 0 0 0 0 0

2 1 2 0 0 0 0

3 2 1 0 0 0 0

4 3 1 0 0 0 0

5 2 1 0 0 0 0

6 5 1 0 0 0 0

7 6 1 0 0 0 0

7 4 1 0 0 0 0

8 7 1 0 0 0 0

9 8 1 0 0 0 0

10 9 1 0 0 0 0

11 8 2 0 0 0 0

12 7 1 0 0 0 0

13 12 2 0 0 0 0

14 13 1 0 0 0 0

15 14 2 0 0 0 0

16 15 1 0 0 0 0

17 16 1 0 0 0 0

18 15 1 0 0 0 0

19 18 2 0 0 0 0

19 12 1 0 0 0 0

20 19 1 0 0 0 0

21 20 1 0 0 0 0

22 20 2 0 0 0 0

M CHG 2 20	1 21 -1

M END

**Methyl 1-(2-Nitrophenyl)-4-oxo-2-cyclohexene-1-carboxylate (3h)**

Compound identifier (MolFile):

SMILES: O=C(CC1)C=CC1(C(OC)=O)C2=CC=CC=C2[N+]([O-])=O

MOL Text:

Actelion Java MolfileCreator 1.0

20 21 0 0 0 0 0 0 0 0999 V2000

0.8660	4.0000	-0.0000 O	0 0 0 0 0 0 0 0 0 0 0 0

0.8660	3.0000	-0.0000 C	0 0 0 0 0 0 0 0 0 0 0 0

-0.0000	2.5000	-0.0000 C	0 0 0 0 0 0 0 0 0 0 0 0

-0.0000	1.5000	-0.0000 C	0 0 0 0 0 0 0 0 0 0 0 0

1.7321	2.5000	-0.0000 C	0 0 0 0 0 0 0 0 0 0 0 0

1.7321	1.5000	-0.0000 C	0 0 0 0 0 0 0 0 0 0 0 0

0.8660	1.0000	-0.0000 C	0 0 0 0 0 0 0 0 0 0 0 0

0.8660	-0.0000	-0.0000 C	0 0 0 0 0 0 0 0 0 0 0 0

1.8376	-0.6266	-0.0000 O	0 0 0 0 0 0 0 0 0 0 0 0

2.5981	-0.0000	-0.0000 C	0 0 0 0 0 0 0 0 0 0 0 0

-0.2083	-0.4278	-0.0000 O	0 0 0 0 0 0 0 0 0 0 0 0

0.0000	0.5000	-0.0000 C	0 0 0 0 0 0 0 0 0 0 0 0

-0.8660	1.0000	-0.0000 C	0 0 0 0 0 0 0 0 0 0 0 0

-1.7321	0.5000	-0.0000 C	0 0 0 0 0 0 0 0 0 0 0 0

-1.7321	-0.5000	-0.0000 C	0 0 0 0 0 0 0 0 0 0 0 0

-0.8660	-1.0000	-0.0000 C	0 0 0 0 0 0 0 0 0 0 0 0

0.0658	-0.6653	-0.0000 C	0 0 0 0 0 0 0 0 0 0 0 0

0.8660	-1.0000	-0.0000 N	0 0 0 0 0 0 0 0 0 0 0 0

0.8660	-2.0000	-0.0000 O	0 0 0 0 0 0 0 0 0 0 0 0

1.7372	-0.2740	-0.0000 O	0 0 0 0 0 0 0 0 0 0 0 0

2 1 2 0 0 0 0

3 2 1 0 0 0 0

4 3 1 0 0 0 0

5 2 1 0 0 0 0

6 5 2 0 0 0 0

7 6 1 0 0 0 0

7 4 1 0 0 0 0

8 7 1 0 0 0 0

9 8 1 0 0 0 0

10 9 1 0 0 0 0

11 8 2 0 0 0 0

12 7 1 0 0 0 0

13 12 2 0 0 0 0

14 13 1 0 0 0 0

15 14 2 0 0 0 0

16 15 1 0 0 0 0

17 16 2 0 0 0 0

17 12 1 0 0 0 0

18 17 1 0 0 0 0

19 18 1 0 0 0 0

20 18 2 0 0 0 0

M CHG 2 18	1 19 -1

M END

**Methyl 3-Oxo-2,3,4,5-tetrahydro-2,6-methanobenzo[b]azocine-6(1H)-carboxylate (4a)**

Compound identifier (MolFile):

SMILES: O=C1CCC2(C(OC)=O)C3=CC=CC=C3NC1C2

MOL Text:

Actelion Java MolfileCreator 1.0

18 20 0 0 0 0 0 0 0 0999 V2000

4.0981	2.5981	-0.0000 O	0 0 0 0 0 0 0 0 0 0 0 0

3.5981	1.7321	-0.0000 C	0 0 0 0 0 0 0 0 0 0 0 0

2.5981	1.7321	-0.0000 C	0 0 0 0 0 0 0 0 0 0 0 0

2.0981	0.8660	-0.0000 C	0 0 0 0 0 0 0 0 0 0 0 0

2.5981	-0.0000	-0.0000 C	0 0 0 0 0 0 0 0 0 0 0 0

1.7321	-0.5000	-0.0000 C	0 0 0 0 0 0 0 0 0 0 0 0

0.8660	-0.0000	-0.0000 O	0 0 0 0 0 0 0 0 0 0 0 0

0.0000	-0.5000	-0.0000 C	0 0 0 0 0 0 0 0 0 0 0 0

1.7321	-1.5000	-0.0000 O	0 0 0 0 0 0 0 0 0 0 0 0

3.2647	-1.1547	-0.0000 C	0 0 0 0 0 0 0 0 0 0 0 0

2.7647	-2.0207	-0.0000 C	0 0 0 0 0 0 0 0 0 0 0 0

3.2647	-2.8868	-0.0000 C	0 0 0 0 0 0 0 0 0 0 0 0

4.2647	-2.8868	-0.0000 C	0 0 0 0 0 0 0 0 0 0 0 0

4.7647	-2.0207	-0.0000 C	0 0 0 0 0 0 0 0 0 0 0 0

4.2647	-1.1547	-0.0000 C	0 0 0 0 0 0 0 0 0 0 0 0

4.7647	-0.2887	-0.0000 N	0 0 0 0 0 0 0 0 0 0 0 0

4.0981	0.8660	-0.0000 C	0 0 0 0 0 0 0 0 0 0 0 0

3.5981	-0.0000	-0.0000 C	0 0 0 0 0 0 0 0 0 0 0 0

2 1 2 0 0 0 0

3 2 1 0 0 0 0

4 3 1 0 0 0 0

5 4 1 0 0 0 0

6 5 1 0 0 0 0

7 6 1 0 0 0 0

8 7 1 0 0 0 0

9 6 2 0 0 0 0

10 5 1 0 0 0 0

11 10 2 0 0 0 0

12 11 1 0 0 0 0

13 12 2 0 0 0 0

14 13 1 0 0 0 0

15 14 2 0 0 0 0

15 10 1 0 0 0 0

16 15 1 0 0 0 0

17 16 1 0 0 0 0

17 2 1 0 0 0 0

18 17 1 0 0 0 0

18 5 1 0 0 0 0

M END

**Methyl 9-Fluoro-3-oxo-2,3,4,5-tetrahydro-2,6-methanobenzo[b]azocine-6(1H)-carboxylate (4b)**

Compound identifier (MolFile):

SMILES: O=C1CCC2(C(OC)=O)C3=CC=C(F)C=C3NC1C2

MOL Text:

Actelion Java MolfileCreator 1.0

19 21 0 0 0 0 0 0 0 0999 V2000

4.0981	2.5981	-0.0000 O	0 0 0 0 0 0 0 0 0 0 0 0

3.5981	1.7321	-0.0000 C	0 0 0 0 0 0 0 0 0 0 0 0

2.5981	1.7321	-0.0000 C	0 0 0 0 0 0 0 0 0 0 0 0

2.0981	0.8660	-0.0000 C	0 0 0 0 0 0 0 0 0 0 0 0

2.5981	-0.0000	-0.0000 C	0 0 0 0 0 0 0 0 0 0 0 0

1.7321	-0.5000	-0.0000 C	0 0 0 0 0 0 0 0 0 0 0 0

0.8660	-0.0000	-0.0000 O	0 0 0 0 0 0 0 0 0 0 0 0

0.0000	-0.5000	-0.0000 C	0 0 0 0 0 0 0 0 0 0 0 0

1.7321	-1.5000	-0.0000 O	0 0 0 0 0 0 0 0 0 0 0 0

3.2647	-1.1547	-0.0000 C	0 0 0 0 0 0 0 0 0 0 0 0

2.7647	-2.0207	-0.0000 C	0 0 0 0 0 0 0 0 0 0 0 0

3.2647	-2.8868	-0.0000 C	0 0 0 0 0 0 0 0 0 0 0 0

4.2647	-2.8868	-0.0000 C	0 0 0 0 0 0 0 0 0 0 0 0

4.7647	-3.7528	-0.0000 F	0 0 0 0 0 0 0 0 0 0 0 0

4.7647	-2.0207	-0.0000 C	0 0 0 0 0 0 0 0 0 0 0 0

4.2647	-1.1547	-0.0000 C	0 0 0 0 0 0 0 0 0 0 0 0

4.7647	-0.2887	-0.0000 N	0 0 0 0 0 0 0 0 0 0 0 0

4.0981	0.8660	-0.0000 C	0 0 0 0 0 0 0 0 0 0 0 0

3.5981	-0.0000	-0.0000 C	0 0 0 0 0 0 0 0 0 0 0 0

2 1 2 0 0 0 0

3 2 1 0 0 0 0

4 3 1 0 0 0 0

5 4 1 0 0 0 0

6 5 1 0 0 0 0

7 6 1 0 0 0 0

8 7 1 0 0 0 0

9 6 2 0 0 0 0

10 5 1 0 0 0 0

11 10 2 0 0 0 0

12 11 1 0 0 0 0

13 12 2 0 0 0 0

14 13 1 0 0 0 0

15 13 1 0 0 0 0

16 15 2 0 0 0 0

16 10 1 0 0 0 0

17 16 1 0 0 0 0

18 17 1 0 0 0 0

18 2 1 0 0 0 0

19 18 1 0 0 0 0

19 5 1 0 0 0 0

M END

**Methyl 9-Chloro-3-oxo-2,3,4,5-tetrahydro-2,6-methanobenzo[b]azocine-6(1H)-carboxylate (4c)**

Compound identifier (MolFile):

SMILES: O=C1CCC2(C(OC)=O)C3=CC=C(Cl)C=C3NC1C2

MOL Text:

Actelion Java MolfileCreator 1.0

19 21 0 0 0 0 0 0 0 0999 V2000

4.0981	2.5981	-0.0000 O	0 0 0 0 0 0 0 0 0 0 0 0

3.5981	1.7321	-0.0000 C	0 0 0 0 0 0 0 0 0 0 0 0

2.5981	1.7321	-0.0000 C	0 0 0 0 0 0 0 0 0 0 0 0

2.0981	0.8660	-0.0000 C	0 0 0 0 0 0 0 0 0 0 0 0

2.5981	-0.0000	-0.0000 C	0 0 0 0 0 0 0 0 0 0 0 0

1.7321	-0.5000	-0.0000 C	0 0 0 0 0 0 0 0 0 0 0 0

0.8660	-0.0000	-0.0000 O	0 0 0 0 0 0 0 0 0 0 0 0

0.0000	-0.5000	-0.0000 C	0 0 0 0 0 0 0 0 0 0 0 0

1.7321	-1.5000	-0.0000 O	0 0 0 0 0 0 0 0 0 0 0 0

3.2647	-1.1547	-0.0000 C	0 0 0 0 0 0 0 0 0 0 0 0

2.7647	-2.0207	-0.0000 C	0 0 0 0 0 0 0 0 0 0 0 0

3.2647	-2.8868	-0.0000 C	0 0 0 0 0 0 0 0 0 0 0 0

4.2647	-2.8868	-0.0000 C	0 0 0 0 0 0 0 0 0 0 0 0

4.7647	-3.7528	-0.0000 Cl 0 0 0 0 0 0 0 0 0 0 0 0

4.7647	-2.0207	-0.0000 C	0 0 0 0 0 0 0 0 0 0 0 0

4.2647	-1.1547	-0.0000 C	0 0 0 0 0 0 0 0 0 0 0 0

4.7647	-0.2887	-0.0000 N	0 0 0 0 0 0 0 0 0 0 0 0

4.0981	0.8660	-0.0000 C	0 0 0 0 0 0 0 0 0 0 0 0

3.5981	-0.0000	-0.0000 C	0 0 0 0 0 0 0 0 0 0 0 0

2 1 2 0 0 0 0

3 2 1 0 0 0 0

4 3 1 0 0 0 0

5 4 1 0 0 0 0

6 5 1 0 0 0 0

7 6 1 0 0 0 0

8 7 1 0 0 0 0

9 6 2 0 0 0 0

10 5 1 0 0 0 0

11 10 2 0 0 0 0

12 11 1 0 0 0 0

13 12 2 0 0 0 0

14 13 1 0 0 0 0

15 13 1 0 0 0 0

16 15 2 0 0 0 0

16 10 1 0 0 0 0

17 16 1 0 0 0 0

18 17 1 0 0 0 0

18 2 1 0 0 0 0

19 18 1 0 0 0 0

19 5 1 0 0 0 0

M END

**Methyl 9-Bromo-3-oxo-2,3,4,5-tetrahydro-2,6-methanobenzo[b]azocine-6(1H)-carboxylate (4d)**

Compound identifier (MolFile):

SMILES: O=C1CCC2(C(OC)=O)C3=CC=C(Br)C=C3NC1C2

MOL Text:

Actelion Java MolfileCreator 1.0

19 21 0 0 0 0 0 0 0 0999 V2000

4.0981	2.5981	-0.0000 O	0 0 0 0 0 0 0 0 0 0 0 0

3.5981	1.7321	-0.0000 C	0 0 0 0 0 0 0 0 0 0 0 0

2.5981	1.7321	-0.0000 C	0 0 0 0 0 0 0 0 0 0 0 0

2.0981	0.8660	-0.0000 C	0 0 0 0 0 0 0 0 0 0 0 0

2.5981	-0.0000	-0.0000 C	0 0 0 0 0 0 0 0 0 0 0 0

1.7321	-0.5000	-0.0000 C	0 0 0 0 0 0 0 0 0 0 0 0

0.8660	-0.0000	-0.0000 O	0 0 0 0 0 0 0 0 0 0 0 0

0.0000	-0.5000	-0.0000 C	0 0 0 0 0 0 0 0 0 0 0 0

1.7321	-1.5000	-0.0000 O	0 0 0 0 0 0 0 0 0 0 0 0

3.2647	-1.1547	-0.0000 C	0 0 0 0 0 0 0 0 0 0 0 0

2.7647	-2.0207	-0.0000 C	0 0 0 0 0 0 0 0 0 0 0 0

3.2647	-2.8868	-0.0000 C	0 0 0 0 0 0 0 0 0 0 0 0

4.2647	-2.8868	-0.0000 C	0 0 0 0 0 0 0 0 0 0 0 0

4.7647	-3.7528	-0.0000 Br 0 0 0 0 0 0 0 0 0 0 0 0

4.7647	-2.0207	-0.0000 C	0 0 0 0 0 0 0 0 0 0 0 0

4.2647	-1.1547	-0.0000 C	0 0 0 0 0 0 0 0 0 0 0 0

4.7647	-0.2887	-0.0000 N	0 0 0 0 0 0 0 0 0 0 0 0

4.0981	0.8660	-0.0000 C	0 0 0 0 0 0 0 0 0 0 0 0

3.5981	-0.0000	-0.0000 C	0 0 0 0 0 0 0 0 0 0 0 0

2 1 2 0 0 0 0

3 2 1 0 0 0 0

4 3 1 0 0 0 0

5 4 1 0 0 0 0

6 5 1 0 0 0 0

7 6 1 0 0 0 0

8 7 1 0 0 0 0

9 6 2 0 0 0 0

10 5 1 0 0 0 0

11 10 2 0 0 0 0

12 11 1 0 0 0 0

13 12 2 0 0 0 0

14 13 1 0 0 0 0

15 13 1 0 0 0 0

16 15 2 0 0 0 0

16 10 1 0 0 0 0

17 16 1 0 0 0 0

18 17 1 0 0 0 0

18 2 1 0 0 0 0

19 18 1 0 0 0 0

19 5 1 0 0 0 0

M END

**Methyl 8-Fluoro-3-oxo-2,3,4,5-tetrahydro-2,6-methanobenzo[b]azocine-6(1H)-carboxylate (4e)**

Compound identifier (MolFile):

SMILES: O=C1CCC2(C(OC)=O)C3=CC(F)=CC=C3NC1C2

MOL Text:

Actelion Java MolfileCreator 1.0

19 21 0 0 0 0 0 0 0 0999 V2000

4.0981	2.5981	-0.0000 O	0 0 0 0 0 0 0 0 0 0 0 0

3.5981	1.7321	-0.0000 C	0 0 0 0 0 0 0 0 0 0 0 0

2.5981	1.7321	-0.0000 C	0 0 0 0 0 0 0 0 0 0 0 0

2.0981	0.8660	-0.0000 C	0 0 0 0 0 0 0 0 0 0 0 0

2.5981	-0.0000	-0.0000 C	0 0 0 0 0 0 0 0 0 0 0 0

1.7321	-0.5000	-0.0000 C	0 0 0 0 0 0 0 0 0 0 0 0

0.8660	-0.0000	-0.0000 O	0 0 0 0 0 0 0 0 0 0 0 0

0.0000	-0.5000	-0.0000 C	0 0 0 0 0 0 0 0 0 0 0 0

1.7321	-1.5000	-0.0000 O	0 0 0 0 0 0 0 0 0 0 0 0

3.2647	-1.1547	-0.0000 C	0 0 0 0 0 0 0 0 0 0 0 0

2.7647	-2.0207	-0.0000 C	0 0 0 0 0 0 0 0 0 0 0 0

3.2647	-2.8868	-0.0000 C	0 0 0 0 0 0 0 0 0 0 0 0

2.7647	-3.7528	-0.0000 F	0 0 0 0 0 0 0 0 0 0 0 0

4.2647	-2.8868	-0.0000 C	0 0 0 0 0 0 0 0 0 0 0 0

4.7647	-2.0207	-0.0000 C	0 0 0 0 0 0 0 0 0 0 0 0

4.2647	-1.1547	-0.0000 C	0 0 0 0 0 0 0 0 0 0 0 0

4.7647	-0.2887	-0.0000 N	0 0 0 0 0 0 0 0 0 0 0 0

4.0981	0.8660	-0.0000 C	0 0 0 0 0 0 0 0 0 0 0 0

3.5981	-0.0000	-0.0000 C	0 0 0 0 0 0 0 0 0 0 0 0

2 1 2 0 0 0 0

3 2 1 0 0 0 0

4 3 1 0 0 0 0

5 4 1 0 0 0 0

6 5 1 0 0 0 0

7 6 1 0 0 0 0

8 7 1 0 0 0 0

9 6 2 0 0 0 0

10 5 1 0 0 0 0

11 10 2 0 0 0 0

12 11 1 0 0 0 0

13 12 1 0 0 0 0

14 12 2 0 0 0 0

15 14 1 0 0 0 0

16 15 2 0 0 0 0

16 10 1 0 0 0 0

17 16 1 0 0 0 0

18 17 1 0 0 0 0

18 2 1 0 0 0 0

19 18 1 0 0 0 0

19 5 1 0 0 0 0

M END

**Methyl 10-Fluoro-3-oxo-2,3,4,5-tetrahydro-2,6-methanobenzo[b]azocine-6(1H)-carboxylate (4f)**

Compound identifier (MolFile):

SMILES: O=C1CCC2(C(OC)=O)C3=CC=CC(F)=C3NC1C2

MOL Text:

Actelion Java MolfileCreator 1.0

19 21 0 0 0 0 0 0 0 0999 V2000

4.0981	2.5981	-0.0000 O	0 0 0 0 0 0 0 0 0 0 0 0

3.5981	1.7321	-0.0000 C	0 0 0 0 0 0 0 0 0 0 0 0

2.5981	1.7321	-0.0000 C	0 0 0 0 0 0 0 0 0 0 0 0

2.0981	0.8660	-0.0000 C	0 0 0 0 0 0 0 0 0 0 0 0

2.5981	-0.0000	-0.0000 C	0 0 0 0 0 0 0 0 0 0 0 0

1.7321	-0.5000	-0.0000 C	0 0 0 0 0 0 0 0 0 0 0 0

0.8660	-0.0000	-0.0000 O	0 0 0 0 0 0 0 0 0 0 0 0

0.0000	-0.5000	-0.0000 C	0 0 0 0 0 0 0 0 0 0 0 0

1.7321	-1.5000	-0.0000 O	0 0 0 0 0 0 0 0 0 0 0 0

3.2647	-1.1547	-0.0000 C	0 0 0 0 0 0 0 0 0 0 0 0

2.7647	-2.0207	-0.0000 C	0 0 0 0 0 0 0 0 0 0 0 0

3.2647	-2.8868	-0.0000 C	0 0 0 0 0 0 0 0 0 0 0 0

4.2647	-2.8868	-0.0000 C	0 0 0 0 0 0 0 0 0 0 0 0

4.7647	-2.0207	-0.0000 C	0 0 0 0 0 0 0 0 0 0 0 0

5.7647	-2.0207	-0.0000 F	0 0 0 0 0 0 0 0 0 0 0 0

4.2647	-1.1547	-0.0000 C	0 0 0 0 0 0 0 0 0 0 0 0

4.7647	-0.2887	-0.0000 N	0 0 0 0 0 0 0 0 0 0 0 0

4.0981	0.8660	-0.0000 C	0 0 0 0 0 0 0 0 0 0 0 0

3.5981	-0.0000	-0.0000 C	0 0 0 0 0 0 0 0 0 0 0 0

2 1 2 0 0 0 0

3 2 1 0 0 0 0

4 3 1 0 0 0 0

5 4 1 0 0 0 0

6 5 1 0 0 0 0

7 6 1 0 0 0 0

8 7 1 0 0 0 0

9 6 2 0 0 0 0

10 5 1 0 0 0 0

11 10 2 0 0 0 0

12 11 1 0 0 0 0

13 12 2 0 0 0 0

14 13 1 0 0 0 0

15 14 1 0 0 0 0

16 14 2 0 0 0 0

16 10 1 0 0 0 0

17 16 1 0 0 0 0

18 17 1 0 0 0 0

18 2 1 0 0 0 0

19 18 1 0 0 0 0

19 5 1 0 0 0 0

M END

**Methyl 9-Methoxy-3-oxo-2,3,4,5-tetrahydro-2,6-methanobenzo[b]azocine-6(1H)-carboxylate (4g)**

Compound identifier (MolFile):

SMILES: O=C1CCC2(C(OC)=O)C3=CC=C(OC)C=C3NC1C2

MOL Text:

Actelion Java MolfileCreator 1.0

20 22 0 0 0 0 0 0 0 0999 V2000

4.0981	2.5981	-0.0000 O	0 0 0 0 0 0 0 0 0 0 0 0

3.5981	1.7321	-0.0000 C	0 0 0 0 0 0 0 0 0 0 0 0

2.5981	1.7321	-0.0000 C	0 0 0 0 0 0 0 0 0 0 0 0

2.0981	0.8660	-0.0000 C	0 0 0 0 0 0 0 0 0 0 0 0

2.5981	-0.0000	-0.0000 C	0 0 0 0 0 0 0 0 0 0 0 0

1.7321	-0.5000	-0.0000 C	0 0 0 0 0 0 0 0 0 0 0 0

0.8660	-0.0000	-0.0000 O	0 0 0 0 0 0 0 0 0 0 0 0

0.0000	-0.5000	-0.0000 C	0 0 0 0 0 0 0 0 0 0 0 0

1.7321	-1.5000	-0.0000 O	0 0 0 0 0 0 0 0 0 0 0 0

3.2647	-1.1547	-0.0000 C	0 0 0 0 0 0 0 0 0 0 0 0

2.7647	-2.0207	-0.0000 C	0 0 0 0 0 0 0 0 0 0 0 0

3.2647	-2.8868	-0.0000 C	0 0 0 0 0 0 0 0 0 0 0 0

4.2647	-2.8868	-0.0000 C	0 0 0 0 0 0 0 0 0 0 0 0

4.7647	-3.7528	-0.0000 O	0 0 0 0 0 0 0 0 0 0 0 0

5.7647	-3.7528	-0.0000 C	0 0 0 0 0 0 0 0 0 0 0 0

4.7647	-2.0207	-0.0000 C	0 0 0 0 0 0 0 0 0 0 0 0

4.2647	-1.1547	-0.0000 C	0 0 0 0 0 0 0 0 0 0 0 0

4.7647	-0.2887	-0.0000 N	0 0 0 0 0 0 0 0 0 0 0 0

4.0981	0.8660	-0.0000 C	0 0 0 0 0 0 0 0 0 0 0 0

3.5981	-0.0000	-0.0000 C	0 0 0 0 0 0 0 0 0 0 0 0

2 1 2 0 0 0 0

3 2 1 0 0 0 0

4 3 1 0 0 0 0

5 4 1 0 0 0 0

6 5 1 0 0 0 0

7 6 1 0 0 0 0

8 7 1 0 0 0 0

9 6 2 0 0 0 0

10 5 1 0 0 0 0

11 10 2 0 0 0 0

12 11 1 0 0 0 0

13 12 2 0 0 0 0

14 13 1 0 0 0 0

15 14 1 0 0 0 0

16 13 1 0 0 0 0

17 16 2 0 0 0 0

17 10 1 0 0 0 0

18 17 1 0 0 0 0

19 18 1 0 0 0 0

19 2 1 0 0 0 0

20 19 1 0 0 0 0

20 5 1 0 0 0 0

M END

**Methyl 3-Oxo-2,3-dihydro-2,6-methanobenzo[b]azocine-6(1H)-carboxylate (4h)**

Compound identifier (MolFile):

SMILES: O=C1C=CC2(C(OC)=O)C3=CC=CC=C3NC1C2

MOL Text:

Actelion Java MolfileCreator 1.0

18 20 0 0 0 0 0 0 0 0999 V2000

4.0981	2.5981	-0.0000 O	0 0 0 0 0 0 0 0 0 0 0 0

3.5981	1.7321	-0.0000 C	0 0 0 0 0 0 0 0 0 0 0 0

2.5981	1.7321	-0.0000 C	0 0 0 0 0 0 0 0 0 0 0 0

2.0981	0.8660	-0.0000 C	0 0 0 0 0 0 0 0 0 0 0 0

2.5981	-0.0000	-0.0000 C	0 0 0 0 0 0 0 0 0 0 0 0

1.7321	-0.5000	-0.0000 C	0 0 0 0 0 0 0 0 0 0 0 0

0.8660	-0.0000	-0.0000 O	0 0 0 0 0 0 0 0 0 0 0 0

0.0000	-0.5000	-0.0000 C	0 0 0 0 0 0 0 0 0 0 0 0

1.7321	-1.5000	-0.0000 O	0 0 0 0 0 0 0 0 0 0 0 0

3.2647	-1.1547	-0.0000 C	0 0 0 0 0 0 0 0 0 0 0 0

2.7647	-2.0207	-0.0000 C	0 0 0 0 0 0 0 0 0 0 0 0

3.2647	-2.8868	-0.0000 C	0 0 0 0 0 0 0 0 0 0 0 0

4.2647	-2.8868	-0.0000 C	0 0 0 0 0 0 0 0 0 0 0 0

4.7647	-2.0207	-0.0000 C	0 0 0 0 0 0 0 0 0 0 0 0

4.2647	-1.1547	-0.0000 C	0 0 0 0 0 0 0 0 0 0 0 0

4.7647	-0.2887	-0.0000 N	0 0 0 0 0 0 0 0 0 0 0 0

4.0981	0.8660	-0.0000 C	0 0 0 0 0 0 0 0 0 0 0 0

3.5981	-0.0000	-0.0000 C	0 0 0 0 0 0 0 0 0 0 0 0

2 1 2 0 0 0 0

3 2 1 0 0 0 0

4 3 2 0 0 0 0

5 4 1 0 0 0 0

6 5 1 0 0 0 0

7 6 1 0 0 0 0

8 7 1 0 0 0 0

9 6 2 0 0 0 0

10 5 1 0 0 0 0

11 10 2 0 0 0 0

12 11 1 0 0 0 0

13 12 2 0 0 0 0

14 13 1 0 0 0 0

15 14 2 0 0 0 0

15 10 1 0 0 0 0

16 15 1 0 0 0 0

17 16 1 0 0 0 0

17 2 1 0 0 0 0

18 17 1 0 0 0 0

18 5 1 0 0 0 0

M END

**1-(tert-butyl) 6-methyl 3-((tert-butoxycarbonyl)oxy)-2,5-dihydro-2,6-methanobenzo[b]azocine-1,6-dicarboxylate (5)**

Compound identifier (MolFile):

SMILES: COC(=O)C13CCC(OC(=O)OC(C)(C)C)C(C1)N(C(=O)OC(C)(C)C)c2ccccc23

MOL Text:

Actelion Java MolfileCreator 1.0

32 34 0 0 0 0 0 0 0 0999 V2000

7.6962	-2.3660	-0.0000 C	0 0 0 0 0 0 0 0 0 0 0 0

6.6962	-2.3660	-0.0000 O	0 0 0 0 0 0 0 0 0 0 0 0

6.1962	-1.5000	-0.0000 C	0 0 0 0 0 0 0 0 0 0 0 0

6.6962	-0.6340	-0.0000 O	0 0 0 0 0 0 0 0 0 0 0 0

5.1962	-1.5000	-0.0000 C	0 0 0 0 0 0 0 0 0 0 0 0

5.1962	-0.5000	-0.0000 C	0 0 0 0 0 0 0 0 0 0 0 0

4.3301	0.0000	-0.0000 C	0 0 0 0 0 0 0 0 0 0 0 0

3.4641	-0.5000	-0.0000 C	0 0 0 0 0 0 0 0 0 0 0 0

2.5981	-0.0000	-0.0000 O	0 0 0 0 0 0 0 0 0 0 0 0

1.7321	-0.5000	-0.0000 C	0 0 0 0 0 0 0 0 0 0 0 0

1.7321	-1.5000	-0.0000 O	0 0 0 0 0 0 0 0 0 0 0 0

0.8660	-0.0000	-0.0000 O	0 0 0 0 0 0 0 0 0 0 0 0

0.0000	-0.5000	-0.0000 C	0 0 0 0 0 0 0 0 0 0 0 0

0.5000	-1.3660	-0.0000 C	0 0 0 0 0 0 0 0 0 0 0 0

-0.8660	-1.0000	-0.0000 C	0 0 0 0 0 0 0 0 0 0 0 0

-0.5000	0.3660	-0.0000 C	0 0 0 0 0 0 0 0 0 0 0 0

3.4641	-1.5000	-0.0000 C	0 0 0 0 0 0 0 0 0 0 0 0

4.3301	-2.0000	-0.0000 C	0 0 0 0 0 0 0 0 0 0 0 0

3.4641	-2.8333	-0.0000 N	0 0 0 0 0 0 0 0 0 0 0 0

2.5981	-3.3333	-0.0000 C	0 0 0 0 0 0 0 0 0 0 0 0

2.5981	-4.3333	-0.0000 O	0 0 0 0 0 0 0 0 0 0 0 0

1.7321	-2.8333	-0.0000 O	0 0 0 0 0 0 0 0 0 0 0 0

0.8660	-3.3333	-0.0000 C	0 0 0 0 0 0 0 0 0 0 0 0

1.3660	-4.1994	-0.0000 C	0 0 0 0 0 0 0 0 0 0 0 0

-0.0000	-3.8333	-0.0000 C	0 0 0 0 0 0 0 0 0 0 0 0

0.3660	-2.4673	-0.0000 C	0 0 0 0 0 0 0 0 0 0 0 0

4.3301	-3.3333	-0.0000 C	0 0 0 0 0 0 0 0 0 0 0 0

4.3301	-4.3333	-0.0000 C	0 0 0 0 0 0 0 0 0 0 0 0

5.1962	-4.8333	-0.0000 C	0 0 0 0 0 0 0 0 0 0 0 0

6.0622	-4.3333	-0.0000 C	0 0 0 0 0 0 0 0 0 0 0 0

6.0622	-3.3333	-0.0000 C	0 0 0 0 0 0 0 0 0 0 0 0

5.1962	-2.8333	-0.0000 C	0 0 0 0 0 0 0 0 0 0 0 0

2 1 1 0 0 0 0

3 2 1 0 0 0 0

4 3 2 0 0 0 0

5 3 1 0 0 0 0

6 5 1 0 0 0 0

7 6 1 0 0 0 0

8 7 1 0 0 0 0

9 8 1 0 0 0 0

10 9 1 0 0 0 0

11 10 2 0 0 0 0

12 10 1 0 0 0 0

13 12 1 0 0 0 0

14 13 1 0 0 0 0

15 13 1 0 0 0 0

16 13 1 0 0 0 0

17 8 1 0 0 0 0

18 17 1 0 0 0 0

18 5 1 0 0 0 0

19 17 1 0 0 0 0

20 19 1 0 0 0 0

21 20 2 0 0 0 0

22 20 1 0 0 0 0

23 22 1 0 0 0 0

24 23 1 0 0 0 0

25 23 1 0 0 0 0

26 23 1 0 0 0 0

27 19 1 0 0 0 0

28 27 2 0 0 0 0

29 28 1 0 0 0 0

30 29 2 0 0 0 0

31 30 1 0 0 0 0

32 31 2 0 0 0 0

32 27 1 0 0 0 0

32 5 1 0 0 0 0

M END

**Methyl 1',2',4',5'-tetrahydro-6'H-spiro[[1,3]dioxolane-2,3'-[2,6]methanobenzo[b]azocine]-6'-carboxylate** (**6**)

Compound identifier (MolFile):

SMILES: COC(=O)C24CCC1(OCCO1)C(C2)Nc3ccccc34

MOL Text:

Actelion Java MolfileCreator 1.0

21 24 0 0 0 0 0 0 0 0999 V2000

2.5981	-0.0000	-0.0000 C	0 0 0 0 0 0 0 0 0 0 0 0

1.7321	-0.5000	-0.0000 O	0 0 0 0 0 0 0 0 0 0 0 0

0.8660	-0.0000	-0.0000 C	0 0 0 0 0 0 0 0 0 0 0 0

0.8660	1.0000	-0.0000 O	0 0 0 0 0 0 0 0 0 0 0 0

0.0000	-0.5000	-0.0000 C	0 0 0 0 0 0 0 0 0 0 0 0

-0.5000	0.3660	-0.0000 C	0 0 0 0 0 0 0 0 0 0 0 0

-1.5000	0.3660	-0.0000 C	0 0 0 0 0 0 0 0 0 0 0 0

-2.0000	-0.5000	-0.0000 C	0 0 0 0 0 0 0 0 0 0 0 0

-2.5878	0.3090	-0.0000 O	0 0 0 0 0 0 0 0 0 0 0 0

-3.5388	0.0000	-0.0000 C	0 0 0 0 0 0 0 0 0 0 0 0

-3.5388	-1.0000	-0.0000 C	0 0 0 0 0 0 0 0 0 0 0 0

-2.5878	-1.3090	-0.0000 O	0 0 0 0 0 0 0 0 0 0 0 0

-1.5000	-1.3660	-0.0000 C	0 0 0 0 0 0 0 0 0 0 0 0

-0.5000	-1.3660	-0.0000 C	0 0 0 0 0 0 0 0 0 0 0 0

-0.8333	-2.5207	-0.0000 N	0 0 0 0 0 0 0 0 0 0 0 0

0.1667	-2.5207	-0.0000 C	0 0 0 0 0 0 0 0 0 0 0 0

0.6667	-3.3868	-0.0000 C	0 0 0 0 0 0 0 0 0 0 0 0

1.6667	-3.3868	-0.0000 C	0 0 0 0 0 0 0 0 0 0 0 0

2.1667	-2.5207	-0.0000 C	0 0 0 0 0 0 0 0 0 0 0 0

1.6667	-1.6547	-0.0000 C	0 0 0 0 0 0 0 0 0 0 0 0

0.6667	-1.6547	-0.0000 C	0 0 0 0 0 0 0 0 0 0 0 0

2 1 1 0 0 0 0

3 2 1 0 0 0 0

4 3 2 0 0 0 0

5 3 1 0 0 0 0

6 5 1 0 0 0 0

7 6 1 0 0 0 0

8 7 1 0 0 0 0

9 8 1 0 0 0 0

10 9 1 0 0 0 0

11 10 1 0 0 0 0

12 11 1 0 0 0 0

12 8 1 0 0 0 0

13 8 1 0 0 0 0

14 13 1 0 0 0 0

14 5 1 0 0 0 0

15 13 1 0 0 0 0

16 15 1 0 0 0 0

17 16 2 0 0 0 0

18 17 1 0 0 0 0

19 18 2 0 0 0 0

20 19 1 0 0 0 0

21 20 2 0 0 0 0

21 16 1 0 0 0 0

21 5 1 0 0 0 0

M END

**1'-(tert-butyl) 6'-methyl 4',5'-dihydro-2'H-spiro[[1,3]dioxolane-2,3'-[2,6]methanobenzo[b]azocine]-1',6'-dicarboxylate (7)**

Compound identifier (MolFile):

SMILES: COC(=O)C24CCC1(OCCO1)C(C2)N(C(=O)OC(C)(C)C)c3ccccc34

MOL Text:

Actelion Java MolfileCreator 1.0

28 31 0 0 0 0 0 0 0 0999 V2000

2.5981	-0.0000	-0.0000 C	0 0 0 0 0 0 0 0 0 0 0 0

1.7321	-0.5000	-0.0000 O	0 0 0 0 0 0 0 0 0 0 0 0

0.8660	-0.0000	-0.0000 C	0 0 0 0 0 0 0 0 0 0 0 0

0.8660	1.0000	-0.0000 O	0 0 0 0 0 0 0 0 0 0 0 0

0.0000	-0.5000	-0.0000 C	0 0 0 0 0 0 0 0 0 0 0 0

-0.5000	0.3660	-0.0000 C	0 0 0 0 0 0 0 0 0 0 0 0

-1.5000	0.3660	-0.0000 C	0 0 0 0 0 0 0 0 0 0 0 0

-2.0000	-0.5000	-0.0000 C	0 0 0 0 0 0 0 0 0 0 0 0

-2.5878	0.3090	-0.0000 O	0 0 0 0 0 0 0 0 0 0 0 0

-3.5388	0.0000	-0.0000 C	0 0 0 0 0 0 0 0 0 0 0 0

-3.5388	-1.0000	-0.0000 C	0 0 0 0 0 0 0 0 0 0 0 0

-2.5878	-1.3090	-0.0000 O	0 0 0 0 0 0 0 0 0 0 0 0

-1.5000	-1.3660	-0.0000 C	0 0 0 0 0 0 0 0 0 0 0 0

-0.5000	-1.3660	-0.0000 C	0 0 0 0 0 0 0 0 0 0 0 0

-0.8333	-2.5207	-0.0000 N	0 0 0 0 0 0 0 0 0 0 0 0

-1.3333	-3.3868	-0.0000 C	0 0 0 0 0 0 0 0 0 0 0 0

-0.8333	-4.2528	-0.0000 O	0 0 0 0 0 0 0 0 0 0 0 0

-2.3333	-3.3868	-0.0000 O	0 0 0 0 0 0 0 0 0 0 0 0

-2.8333	-4.2528	-0.0000 C	0 0 0 0 0 0 0 0 0 0 0 0

-1.9673	-4.7528	-0.0000 C	0 0 0 0 0 0 0 0 0 0 0 0

-3.3333	-5.1188	-0.0000 C	0 0 0 0 0 0 0 0 0 0 0 0

-3.6994	-3.7528	-0.0000 C	0 0 0 0 0 0 0 0 0 0 0 0

0.1667	-2.5207	-0.0000 C	0 0 0 0 0 0 0 0 0 0 0 0

0.6667	-3.3868	-0.0000 C	0 0 0 0 0 0 0 0 0 0 0 0

1.6667	-3.3868	-0.0000 C	0 0 0 0 0 0 0 0 0 0 0 0

2.1667	-2.5207	-0.0000 C	0 0 0 0 0 0 0 0 0 0 0 0

1.6667	-1.6547	-0.0000 C	0 0 0 0 0 0 0 0 0 0 0 0

0.6667	-1.6547	-0.0000 C	0 0 0 0 0 0 0 0 0 0 0 0

2 1 1 0 0 0 0

3 2 1 0 0 0 0

4 3 2 0 0 0 0

5 3 1 0 0 0 0

6 5 1 0 0 0 0

7 6 1 0 0 0 0

8 7 1 0 0 0 0

9 8 1 0 0 0 0

10 9 1 0 0 0 0

11 10 1 0 0 0 0

12 11 1 0 0 0 0

12 8 1 0 0 0 0

13 8 1 0 0 0 0

14 13 1 0 0 0 0

14 5 1 0 0 0 0

15 13 1 0 0 0 0

16 15 1 0 0 0 0

17 16 2 0 0 0 0

18 16 1 0 0 0 0

19 18 1 0 0 0 0

20 19 1 0 0 0 0

21 19 1 0 0 0 0

22 19 1 0 0 0 0

23 15 1 0 0 0 0

24 23 2 0 0 0 0

25 24 1 0 0 0 0

26 25 2 0 0 0 0

27 26 1 0 0 0 0

28 27 2 0 0 0 0

28 23 1 0 0 0 0

28 5 1 0 0 0 0

M END
